# New Ru(III) NAMI/NAMI‑A Analogue Complexes with Selective Anticancer Activity

**DOI:** 10.1021/acs.jmedchem.5c03490

**Published:** 2026-07-23

**Authors:** Manel Estruch-Blasco, Jose Manuel Calderón-Montaño, Eleuterio Álvarez, Antonio Sánchez-Coronilla, Miguel López-Lázaro, Manuel Pernia Leal

**Affiliations:** † Departamento de Química Orgánica y Farmacéutica, Facultad de Farmacia, 16778 Universidad de Sevilla, c/Profesor García González, 2, Sevilla 41012, Spain; ‡ Departamento de Farmacología, Facultad de Farmacia, Universidad de Sevilla, c/Profesor García González, 2, Sevilla 41012, Spain; § Instituto de Investigaciones Químicas (IIQ), CSIC-Universidad de Sevilla, avda. Américo Vespucio 49, Sevilla 41092, Spain; ∥ Departamento de Química Física, Facultad de Farmacia, Universidad de Sevilla, c/Profesor García González, 2, Sevilla 41012, Spain

**Keywords:** ruthenium(III), NAMI-A, cancer, pyridine, selective

## Abstract

NAMI-A (ImH)­[*trans*-RuCl_4_(dmso-*S*)­(Im)], where dmso-*S* = sulfur-bonded dimethyl sulfoxide and Im = imidazole emerged as a promising alternative to approved Pt­(II) anticancer drugs, offering high antimetastatic activity and low toxicity. However, its development was discontinued due to insufficient activity against primary tumors. In this work, we synthesized 12 new pyridine NAMI derivatives and evaluated their anticancer activity against three cancer cell lines: lung adenocarcinoma A549, melanoma MeWo and bladder cancer T24, and compared with the nonmalignant keratinocyte HaCaT cells. Among them, two complexes exhibited potent cytotoxicity and selectivity while retaining the characteristic antimetastatic activity of the NAMI scaffold. The Ru­(III) complex (CHO-PyH)­[*trans*-Ru­(CHO-Py)­Cl_4_ (dmso-*S*)], where CHO-Py = pyridine-3-aldehyde, displayed an IC_50_ of 34 μM and a selectivity index of 7.60 against A549 cells. Additionally, (NCS-PyH)­[*trans*-RuCl_4_(dmso-*S*)­(NCS-Py)], where NCS-Py = 3-(isothiocyanatomethyl)­pyridine, showed an IC_50_ of 240 nM and a selectivity index of 5.2 against T24 cells.

## Graphical abstract

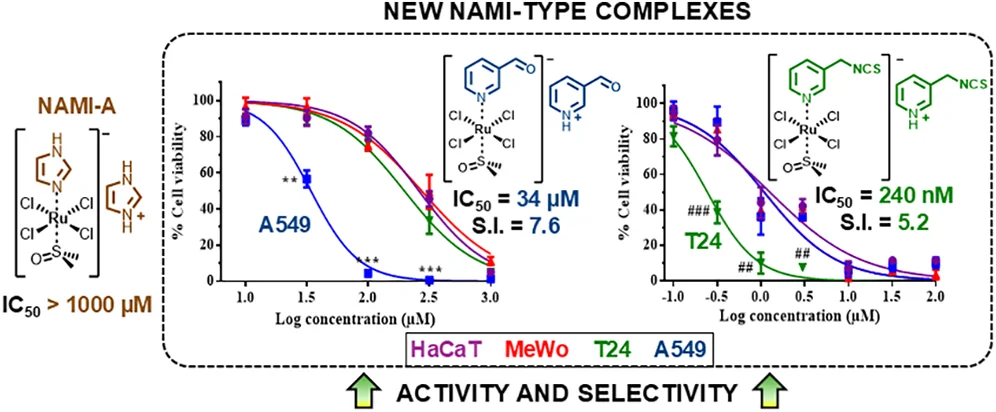

## Introduction

Since the discovery of cisplatin, and its approval for clinical use in 1978, Pt­(II) complexes have been widely used as anticancer agents against a variety of solid tumors worldwide.^1^ Despite their undeniable success, these antineoplastic compounds present severe side effects arising from their high reactivity and low selectivity.^2^ Additionally, some tumors have intrinsic or acquired resistance to these metallodrugs, increasing the need for alternatives.^3^ Consequently, new coordination compounds with other transition metals are being studied. Among them, Ru has emerged as an attractive option. It presents a wider range of geometries and coordination sphere compared to the square planar Pt­(II) complexes,^4^ and potentially a different spectrum of action and noncross resistance due to their distinct mechanism of action.^5^

Significantly, two sets of structurally similar Ru­(III) complexes, with different pharmacological profiles: KP1339/KP1019 and NAMI/NAMI-A, have gained attention in cancer treatment (Figure 1). KP1339 or BOLD-100 (Na­[*trans*-RuCl_4_(Ind)_2_] where Ind = indazole) and KP1019 (IndH)­[*trans*-RuCl_4_(Ind)_2_], demonstrated cytotoxicity against colon cancer cells and are active against platinum-resistant cancers.^6^ Notably, BOLD-100 is currently under clinical evaluation (NCT01415297,^7^ NCT04421820,^8^ NCT07027423 ^9^). On the other hand, NAMI (Na­[*trans*-RuCl_4_(dmso-*S*)­(Im)]) where dmso-*S* = sulfur-bonded dimethyl sulfoxide and Im = imidazole) and NAMI-A (ImH)­[*trans*-RuCl_4_(dmso-*S*)­(Im)], have not shown cytotoxic activity against several tumor cell lines, with the exception of leukemia cells.^10^ However, it has displayed antimetastatic activity, preventing the formation and growth of metastases in several solid tumor mouse models.^11^

**Figure 1:**
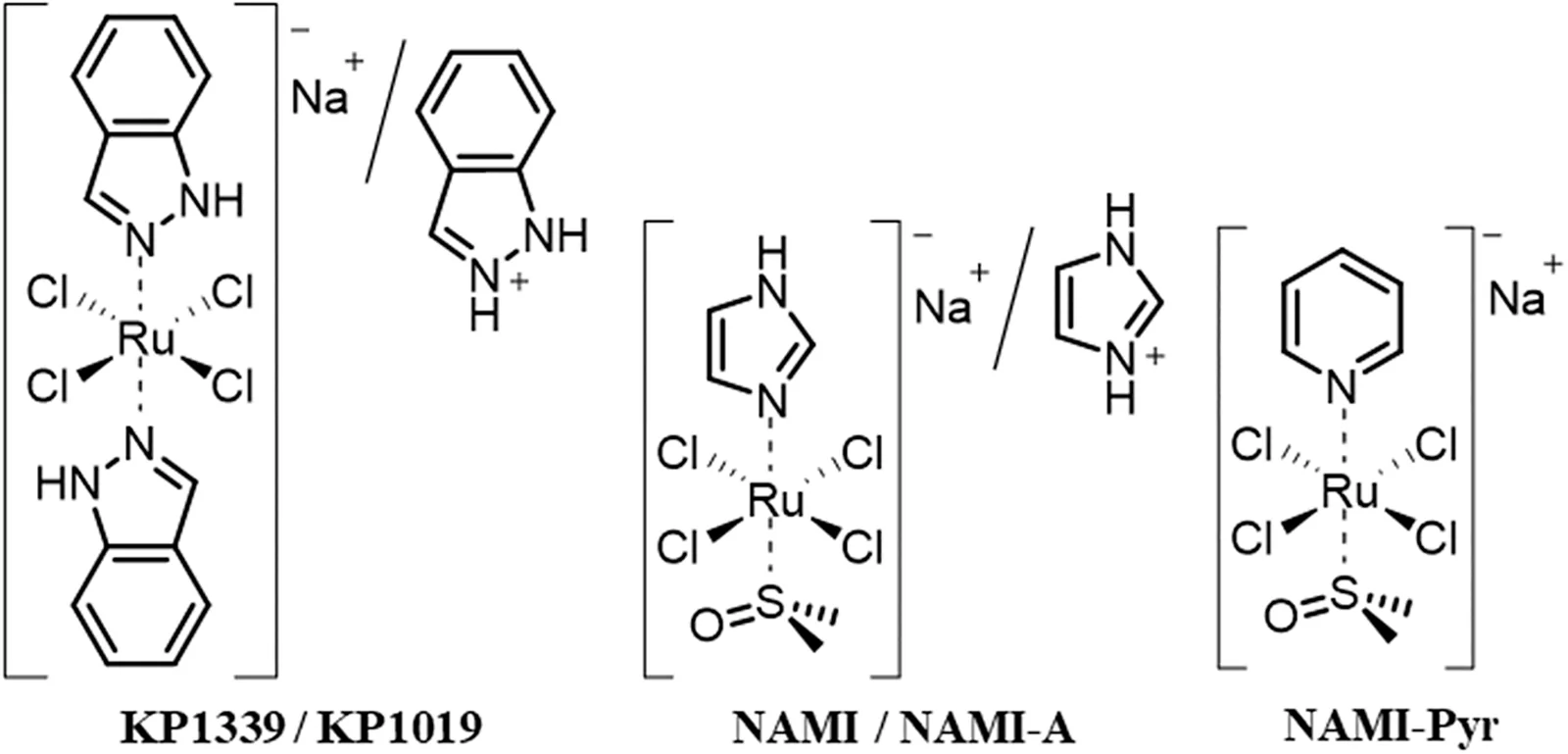
Structures of the Ru­(III) complexes KP1339, Na­[*trans*-RuCl_4_(Ind)_2_], and KP1019, (IndH)­[*trans*-RuCl_4_(Ind)_2_], on the left; NAMI, Na­[*trans*-RuCl_4_(dmso-*S*)­(Im)], and NAMI-A, (ImH)­[*trans*-RuCl_4_(dmso-*S*)­(Im)], in the middle and NAMI-Pyr, Na­[*trans*-RuCl_4_(dmso-*S*)­(Py)], on the right.

NAMI-A was the first ruthenium-based drug to be tested in humans and has reached a phase I/II clinical study in combination with gemcitabine for nonsmall cell lung cancer. However, it was classified as “*insufficiently effective for further use*”, as “*the efficacy of the treatment was lower than expected for gemcitabine alone*”.^12^ Nonetheless, new analogues with higher cytotoxicity are being prepared to overcome this limitation following two different strategies. The first one is the attachment to the heterocyclic ligand of different antiproliferative drugs, such as lonidamine (IC_50_ = 0.5 ± 0.1 μM against A549), bexarotene (IC_50_ = 66.9 ± 13.5 μM against A549) or ethacrynic acid (IC_50_ = 27 ± 2 μM against A2780cisR) and other scaffolds with intrinsic anticancer effect, as diazenecarboxamides, 4-anilinoquinazoline (IC_50_ > 100 μM against MCF-7) or ferrocene (IC_50_ = 69–35 μM against SW480).^13–17^ The second strategy involves attaching scaffolds that enable the formation of macrostructures, with the goal of protecting the compound from hydrolysis, enhance its distribution and increase cell uptake and cytotoxicity.^18–23^

Although the exact mechanism of action is not fully understood yet, these Ru­(III) complexes can be considered prodrugs that are activated by aquation.^24^ And also it has been demonstrated in clinical trials that the Ru complexes mainly interact with transport proteins, with 90% coupled to albumin.^25^ Several studies have shown that the association starts with noncovalent interactions at the hydrophobic or positively charged pockets of proteins. Subsequently, covalent bindings to cysteine and histidine side chains can occur, preventing speciation and oligomerization.^26–29^ That first noncovalent interaction is determined by the nature of the axial heterocycle, which was named as the “*crucial ligand*”. This behavior was displayed in structure–activity relationship (SAR) studies, where changes in this axial ligand resulted in significant variations of the complex-protein interaction. And also pharmacological properties, including cytotoxicity.^30^ Taking this into account, we hypothesized that complex-protein interactions should also depend on the functional group present in the crucial ligand, and possibly, the biological activity.

Even though Na­[*trans*-RuCl_4_(dmso-*S*)­(Py)] (where Py = pyridine), a pyridine analogue of NAMI known as NAMI-Pyr or AziRu (Figure 1), displayed biological activities similar to those of NAMI,^17,19,20,31^ and a few examples of analogues with simple pyridines as axial ligands have been reported.^5,32^ We considered pyridine derivatives bearing interesting functional groups, over imidazole, to be more appropriate for the heterocyclic ligand.^33^ As pyridine is a common and versatile scaffold for drug discovery, especially the ones with known biological activities, such as aldehydes or isothiocyanates.^34–36^

Therefore, in this work we expanded the family of NAMI-Pyr analogue complexes by preparing and characterizing a series of 10 novel compounds featuring pyridines substituted at the 3 or 4 position. The cytotoxic activity of these analogues was evaluated against three human cancer cell lines (A549 lung adenocarcinoma, MeWo melanoma and T24 bladder cancer) and a human nonmalignant cell line (HaCaT keratinocytes). The NAMI-A analogues of the two compounds with best cytotoxic and selectivity properties were also prepared and tested. Moreover, we studied whether these Ru complexes maintained the properties of the NAMI family.

## Results and Discussion

### Synthesis of NAMI-Pyr Analogues

The preparation of Ru­(III) complexes was focused on NAMI-type complexes due to the mild synthesis conditions required compared to the KP complexes, thus allowing the use of a range of functional group possibilities.

First, ligands **L4**, **L6**, **L8** and **L9** were prepared (Schemes S1–S4). Further synthetic details are described in the experimental section. Structure and purity of the synthesized ligands were confirmed by proton nuclear magnetic resonance (^1^H NMR). **L1**, **L2**, **L3** and **L7** are commercially available and were used as received.

Using the ligands **L1**–**L4** and **L6**–**L9** and the Ru­(III) precursor **Ru–P2**, we synthesized the corresponding new NAMI-Pyr analogues **Ru1**–**Ru4** and **Ru6**–**Ru9** (Figure S1). We observed that **Ru–P2** did not yield the desired complex **Ru5** and **Ru10** if a nucleophilic amino group was present in the ligand. To overcome this limitation, these two Ru­(III) complexes were obtained after the *tert*-butyloxycarbonyl (BOC) deprotection of complexes **Ru4** and **Ru9**, respectively (Scheme S6).

It is noteworthy that few complexes are described with amino groups in their structure. They are typically limited to amines conjugated to the heterocycle,^28,29^ which decreases their nucleophilicity. Other strategies involve changing the Ru precursor^37^ or using harsh conditions^38^ that could decompose less stable ligands. In our case, **Ru5** and **Ru10** were rapidly obtained as the corresponding zwitterionic salts after aqueous HCl deprotection of **Ru4** and **Ru9**, respectively. This method enables the use of **Ru–P2** as a common precursor.

The complexes were isolated as yellow to dark orange powders in excellent to quantitative yields (Figure 2). Their purity was assessed through high pressure liquid chromatography (HPLC) and the structures were confirmed by ^1^H NMR, low- and high-resolution mass spectrometry (see Supporting Information), and single crystal X-ray diffraction (SC-XRD).

**Figure 2:**
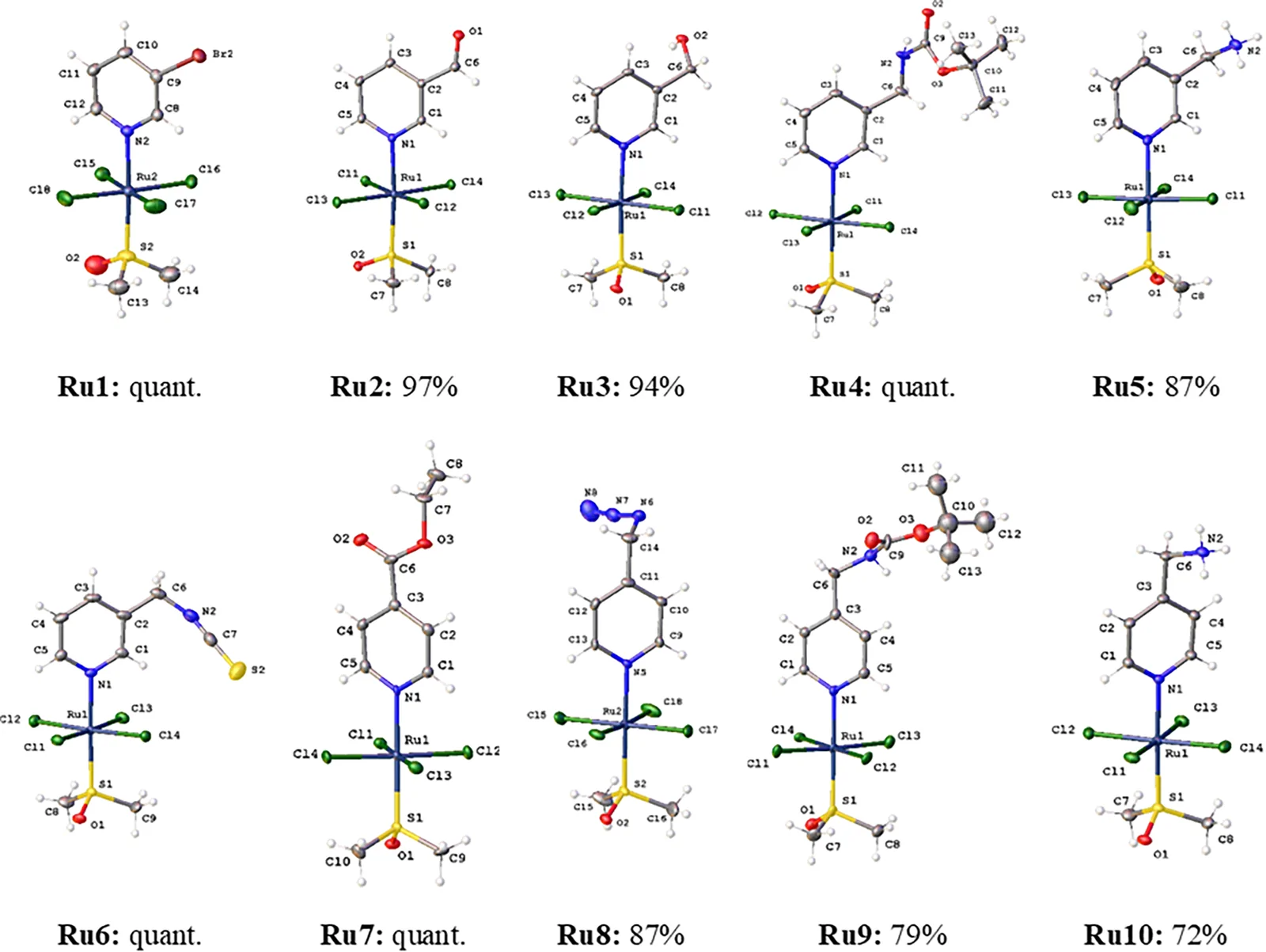
X-ray crystal structures of NAMI-Pyr analogues: **Ru1** to **Ru10**, showing only the anionic part of the sodium salt (except for **Ru5** and **Ru10** which are zwitterions). The sodium cation (if present) as well as other coordinating and crystallizing solvent molecules for each complex are omitted for clarity. Isolated yields are indicated next to their corresponding code.

^1^H NMR spectra of all prepared complexes were recorded. The paramagnetic nature of the Ru­(III) metal (4d^5^, *S* = 1/2) made the specific assignment of the coordinated pyridines signals difficult, that appeared as broad signals (see Supporting Information). Nevertheless, a broad signal around −12 ppm, that corresponds to the coordinated DMSO and is characteristic of this family of complexes was also observed in all spectra, thereby confirming the obtention of NAMI-Pyr analogues.^26^

Negative ionization mass spectrometry was used to confirm the successful preparation of the complexes (see Supporting Information). Their mass analysis revealed characteristic peaks common to the NAMI-Pyr analogue complexes. For instance, in the case of **Ru4** (Figure 3), the base peak at *m*/*z* 243.78 corresponds to the RuCl_4_ central anion with the typical Ru and Cl isotope distribution. The peak at *m*/*z* 321.79, with intensities ranging from 2% to 6%, corresponds to the central anion with the DMSO ligand. Additionally, the peaks corresponding to the complex anion were observed and confirmed with the expected calculated masses. Interestingly, some peaks with the characteristic isotope distributions were observed at higher *m*/*z* than the corresponding molecular complex anion peak. These peaks are in accordance with the observed crystalline polymeric structures.

**Figure 3:**
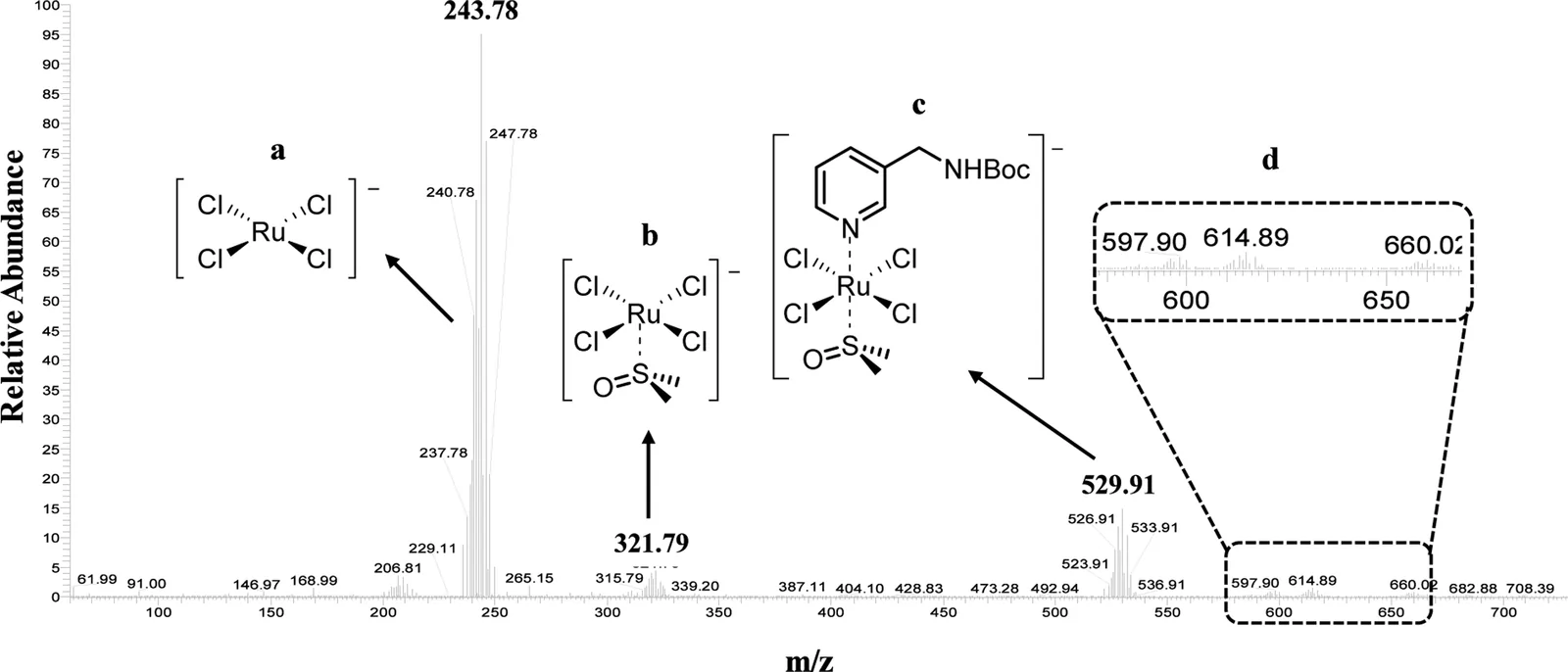
Low resolution mass spectra of **Ru4**. (a) Central RuCl_4_ anion. (b) Central anion with DMSO ligand. (c) Ru4 anion. (d) Crystalline polymeric peaks.

### Structural Analysis of NAMI-Pyr Analogues by Single-Crystal X-ray Diffraction

As evidence supporting the successful synthesis of the NAMI-Pyr analogues, suitable crystals of each complex were obtained using the crystallization methods described in the experimental section. Through these crystals, the structures of complexes **Ru1** to **Ru10** were determined by SC-XRD. Figure 2 illustrates the anionic moieties of the corresponding salts of NAMI-Pyr analogues containing the ruthenium metal.

From the analysis of these crystal structures, the coordination geometry around the ruthenium atom is observed to be octahedral, as expected. In all ten complexes, the average Ru–N bond length for the coordinated pyridine-substituted ligands is 2.114(5) Å. In all cases, the DMSO ligand is coordinated via the sulfur atom with an average Ru–S bond length of 2.292(8) Å. Finally, for all complexes, the average Ru–Cl bond distance is 2.352(8) Å. All these bond distances between the atoms and the central Ru atom are similar to the corresponding values reported for related Ru­(III) complexes with pyridines.^28,39–42^

The crystal structures of these ten ruthenium salts (**Ru1** to **Ru10**) are quite diverse. A summary of each structure is provided in the Supporting Information.

### Evaluation of Selective Cytotoxic Activity of NAMI-Pyr Analogues against Human Cancer Cells and Nonmalignant Cells

In order to test the anticancer activity of our Ru­(III) complexes, we evaluated them in vitro against human cancer cells and human nonmalignant cells. Cancer cell lines used were A549 (lung cancer), MeWo (melanoma), and T24 (bladder cancer), representing some of the most common cancers. To evaluate the selectivity of our compounds, we used human noncancerous HaCaT cells, a cell line derived from adult tissue that has a growth rate similar to that of cancer cells. Most of the anticancer agents currently used in clinics do not have sufficient anticancer selectivity, affecting cells with high proliferation rates, whether cancer cells or normal cells. All cell lines were exposed to several concentrations of our Ru­(III) complexes for 72 h before measuring cell viability with the resazurin assay. Carboplatin, an anticancer drug used in clinics, and NAMI-A were studied under the same experimental conditions. The half-maximal inhibitory concentration (IC_50_) value and the selective index (S.I.) of each compound are collected in Table 1. Figures 4 and S12 display the concentration–response curves for each compound, facilitating comprehension of the cytotoxic profile at a wide range of concentrations.

**Table 1:** IC_50_ Values (μM) of Ru­(III) Complexes and Carboplatin against Human Non-Malignant Cells and Human Cancer Cells^a^

	HaCaT (nonmalignant keratinocyte)	A549 (lung adenocarcinoma)		MeWo (melanoma)		T24 (bladder cancer)	
compound	IC_50_ (95% CI)	IC_50_ (95% CI)	S.I.	IC_50_ (95% CI)	S.I.	IC_50_ (95% CI)	S.I.
**Ru1**	700 (610–790)	560 (490–630)	1.2	710 (610–820)	1.0	480 (440–520)	1.5
**Ru2**	420 (360–480)	90 (80–100)	4.8	300 (230–380)	1.4	310 (220–450)	1.3
**Ru3**	760 (650–890)	610 (530–700)	1.2	750 (650–870)	1.00	500 (470–540)	1.50
**Ru4**	980 (880–1080)	680 (620–740)	1.4	1090 (920–1280)	0.9	640 (580–710)	1.5
**Ru5**	>1000	>1000	N.D.	>1000	N.D.	450 (370–540)	N.D.
**Ru6**	2.7 (2.1–3.4)	2.7 (2.3–3.3)	1.0	3.5 (2.8–4.4)	0.8	1.0 (0.8–1.2)	2.80
**Ru7**	720 (650–800)	480 (400–580)	1.5	700 (610–820)	1.0	420 (350–520)	1.7
**Ru8**	830 (770–890)	630 (500–780)	1.3	930 (800–1080)	0.9	610 (560–660)	1.4
**Ru9**	810 (730–890)	480 (430–530)	1.7	950 (810–1120)	0.9	450 (410–490)	1.8
**Ru10**	650 (440–970)	>1000	N.D.	560 (380–840)	1.2	310 (230–420)	2.1
NAMI-A	1100 (400 to >3000)	>3000	N.D.	2600 (1000 to >3000)	0.4	1700 (300 to >3000)	0.7
carboplatin	29 (25–34)	36 (29–45)	0.80	80 (60–110)	0.36	21 (19–23)	1.40

a Cells were exposed to the drugs for 72 h and then cell viability was determined with the resazurin assay. Selectivity index (S.I.) values were calculated as the IC_50_ value in the nonmalignant cells divided by the IC_50_ value in the cancer cells. Data are presented as the means and their corresponding 95% confidence intervals (95% CI). All data were obtained from at least three independent experiments (see Experimental Section for details). N.D. = Not determined.

**Figure 4:**
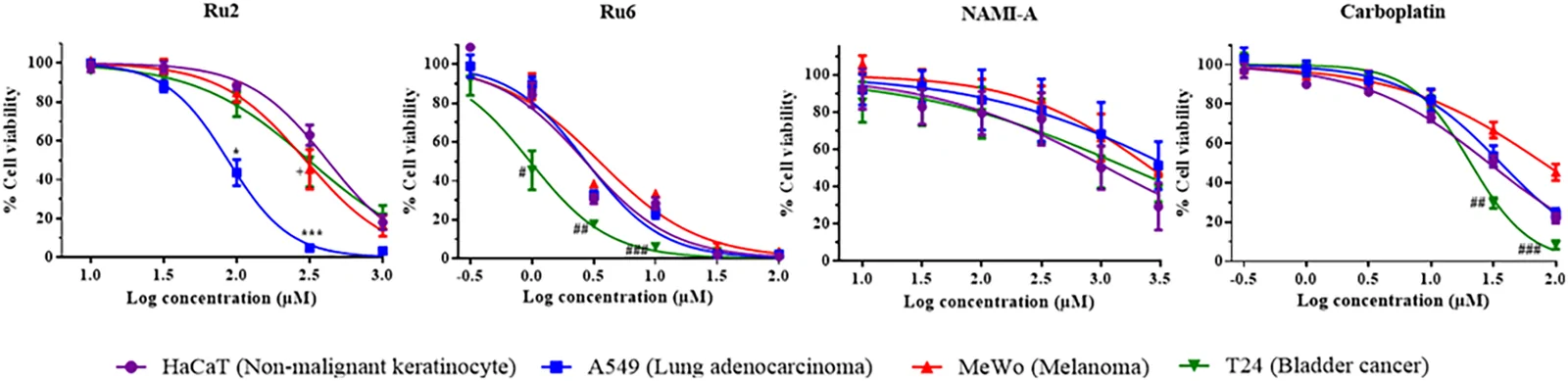
Evaluation of the selective anticancer activity of **Ru2**, **Ru6**, NAMI-A and carboplatin on human cancer cells (A549 in blue, MeWo in red and T24 in green) and human nonmalignant cells (HaCaT in purple). Cells were exposed to several concentrations of compounds for 72 h. After treatment, cell viability was determined with the resazurin assay. Data represent mean ± SEM from at least three independent experiments. Statistical analysis was calculated using the paired *t*-test; * indicates *p* < 0.05, ** indicates *p* < 0.01, ** indicates *p* < 0.001 (similar representations for marks + or #).

NAMI-A, as expected, showed very low cytotoxic activity (Table 1 and Figure 4). Cell viability exceeded 30% even at the highest concentration tested (3 mM). Although our Ru­(III) complexes showed higher activity than NAMI-A, most of them also showed very low cytotoxicity, with IC_50_ values between 400 and 1000 μM. Compounds **Ru1**, **Ru3**, **Ru7** and **Ru8** were the least cytotoxic and selective (Table 1 and Figure S12). The deprotection of the aminoBOC complexes (**Ru5** and **Ru10** versus **Ru4** and **Ru9**) modestly increased the selective cytotoxicity against T24 bladder cancer cells, but decreased the cytotoxic activity against A549 lung cancer cells (Table 1 and Figure S12). Interestingly, the derivative of 3-pyridinecarboxaldehyde (**Ru2**) was approximately five times more cytotoxic against lung cancer cells (A549) than against noncancerous cells (HaCaT), indicating a specific cell line selectivity that was not observed for the other two cancer lines. Although **Ru2** was less active than carboplatin against A549 cells (IC_50_ values 90 and 36 μM, respectively), this Ru­(III) complex presented a better selective profile (Table 1 and Figure 4). In fact, carboplatin only showed modest selective activity against bladder cancer (T24) under our experimental conditions. 3-(isothiocyanatomethyl)­pyridine derivative (**Ru6**) showed the highest cytotoxic activity with IC_50_ values between 1.0 and 3.5 μM, being 10 times more cytotoxic than carboplatin (Table 1 and Figure 4). However, **Ru6** showed only modest selectivity against the bladder cancer cell line, which were twice as sensitive as noncancerous cells.

### Synthesis of NAMI-A Analogues

Encouraged by the significant results of the NAMI-Pyr analogues **Ru2** and **Ru6**, their NAMI-A counterparts were prepared. After reacting **L2** or **L6** with the precursor **Ru–P1**, **Ru11** and **Ru12** were obtained, respectively, as orange solids in excellent yields (Figure 5). Further synthetic details are described in the experimental section. The purity of the complexes was measured with HPLC and elemental analysis (EA), and they were characterized by ^1^H NMR, low- and high-resolution mass spectrometry and SC-XRD. For these NAMI-A analogues, their ^1^H NMR spectra were similar to their sodium counterparts, with the signals of the protonated pyridines appearing at the expected chemical shifts. And similarly to the previously described Ru­(III) complexes, the mass spectra of **Ru11** and **Ru12** exhibited the same peaks as those of complexes **Ru2** and **Ru6**, respectively, since they share the same central anion.

**Figure 5:**
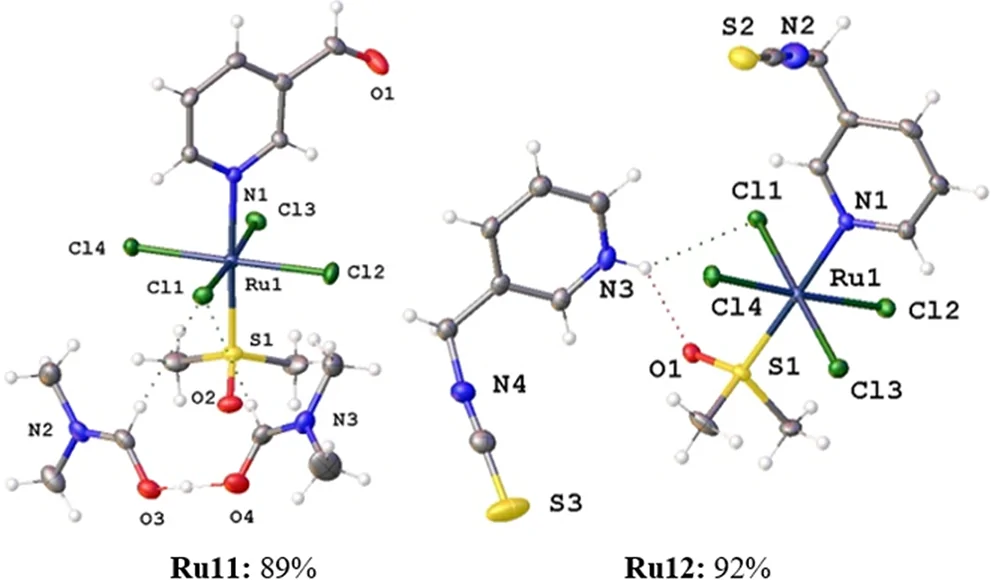
ORTEP view with thermal ellipsoids depicted at the 30% probability level structure of the new NAMI-A analogues **Ru11** and **Ru12**. Isolated yields are indicated next to their corresponding code.

### Structural Analysis of NAMI-A Analogues Single-Crystal X-ray Diffraction

Figure 5 illustrates the crystallized structures of the complexes **Ru11** and **Ru12**. Based also on the structural analysis by single-crystal X-ray diffraction, the coordination geometry around the ruthenium atom, the type of ligands involved, and their average bond lengths in the NAMI-A complexes are shown to be completely equivalent to those described for the NAMI-Pyr complexes mentioned above.

Although the same method was used to prepare the NAMI-A analogues **Ru11** and **Ru12**, the crystal structure of **Ru11** does not show the expected protonated ligand **L2** as counterion but rather a protonated (DMF) dimer (Figures 5 and S13). Albeit uncommon, this counterion has been described in other complexes.^43^ The use of DMF as solvent in the crystallization process to obtain suitable crystals for SC-XRD induces the exchange of the counterion ligand **L2** for the protonated DMF dimer. This is confirmed in the ^1^H NMR spectra of freshly prepared **Ru11**, which is similar to the one from **Ru2** but with additional sharper peaks from the protonated **L2**. Besides, no typical DMF signals around 3 and 8 ppm were observed in the **Ru11** spectra. On the other hand, **Ru12** was crystallized using acetone as solvent, yielding the expected crystal structure.

This result indicates that the desired complex **Ru11** was obtained. DMF was also observed in the crystal structure of **Ru5** (Figure S10) and **Ru10** (Figure S11), as it was necessary for the crystallization process.

### Evaluation of Selective Cytotoxic Activity of NAMI-A Analogues and Their Ligands against Human Cancer Cells and Nonmalignant Cells

We evaluated the cytotoxicity of **Ru11** and **Ru12** against cancer cells and noncancerous cells under the same experimental conditions described above. Gemcitabine, a clinical anticancer drug, was used as a positive control. The results are collected in Table 2 and Figure 6A. Both NAMI-A analogues showed higher activity than their NAMI-Pyr counterparts (**Ru2** and **Ru6**). To further evaluate whether the selective cytotoxic activity of these complexes arose from the presence of the pyridine-based ligands, additional investigations were carried out.

**Table 2:** IC_50_ Values of Complexes **Ru11** (μM), **Ru12** (nM) and Gemcitabine (nM) against Human Non-Malignant Cells and Human Cancer Cells^a^

	HaCaT (nonmalignant keratinocyte)	A549 (lung adenocarcinoma)		MeWo (melanoma)		T24 (bladder cancer)	
compound	IC_50_ (95% CI)	IC_50_ (95% CI)	S.I.	IC_50_ (95% CI)	S.I.	IC_50_ (95% CI)	S.I.
**Ru11**	260 (220–310)	34 (31–37)	7.60	270 (210–350)	0.95	200 (160–240)	1.31
**Ru12**	1.3 (0.9–1.8)	1.1 (0.7–1.6)	1.2	1.1 (0.8–1.4)	1.2	0.24 (0.20–0.29)	5.2
gemcitabine (nM)	10 (8–12)	2.5 (2.1–2.9)	4.1	4 (3–7)	2.3	1.3 (1.0–1.6)	8.0

a Cells were exposed to the drugs for 72 h, and then cell viability was determined with the resazurin assay. Selectivity index (S.I.) values were calculated as the IC_50_ value in the nonmalignant cells divided by the IC_50_ value in the cancer cells obtained in each independent experiment. Data are presented as the means and their corresponding 95% confidence intervals (95% CI). All data were obtained from at least three independent experiments (see Experimental Section for details).

**Figure 6:**
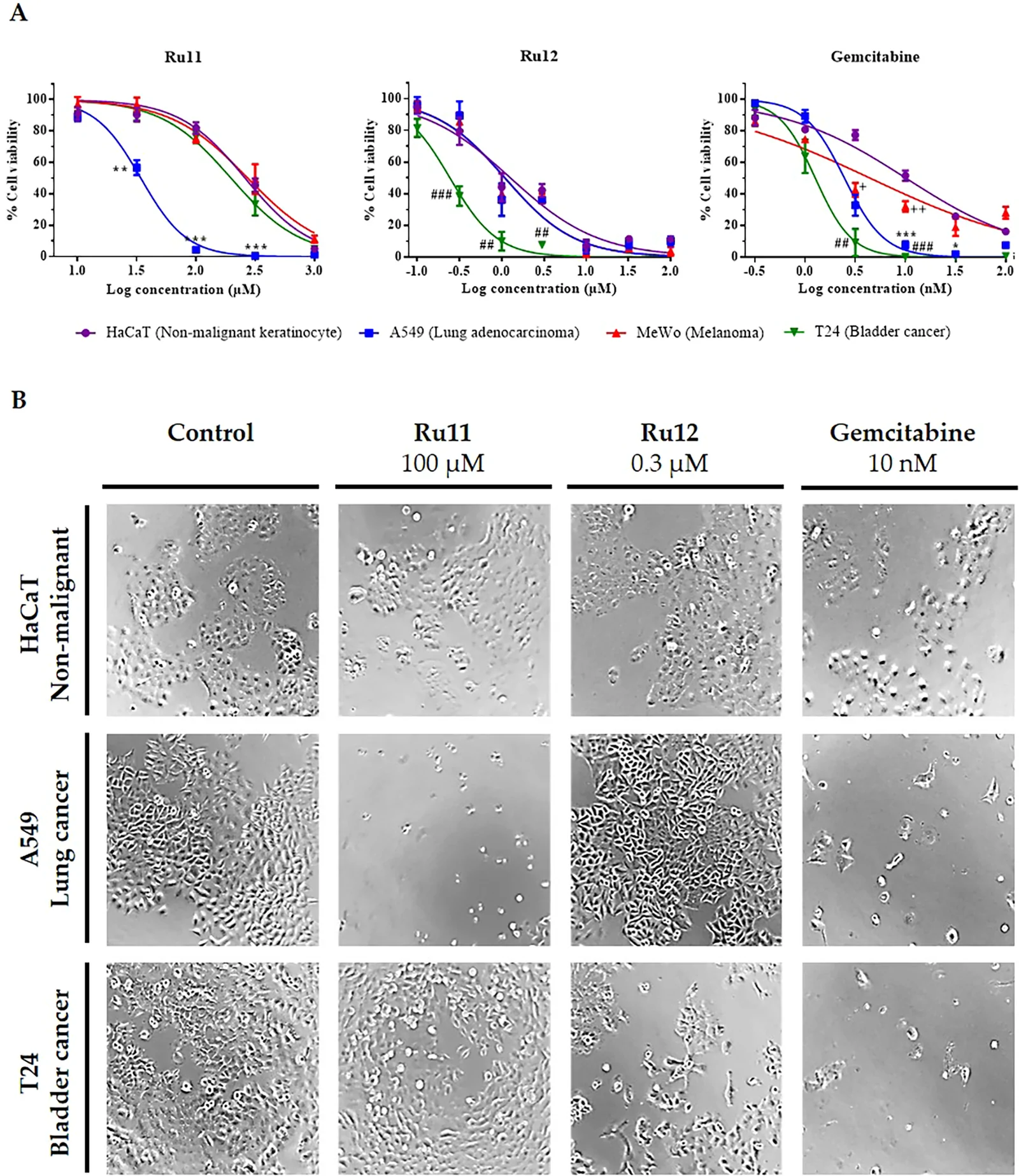
Evaluation of the selective anticancer activity of **Ru11**, **Ru12** and gemcitabine on human cancer cells (A549, MeWo and T24) and human nonmalignant cells (HaCaT). Cells were exposed to several concentrations of compounds for 72 h. After treatment, cell viability was determined with the resazurin assay. (A) Percentage of cell viability. Data represent mean ± SEM from at least three independent experiments. Statistical analysis was calculated using the paired *t*-test; * indicates *p* < 0.05, ** indicates *p* < 0.01, ** indicates *p* < 0.001 (similar representations for marks + or #). (B) Representative photographs of HaCaT, A549 and T24 cells in the absence (control) or presence of **Ru11** (100 μM), **Ru12** (0.3 μM) and gemcitabine (10 nM). Images were taken using a Huawei P9 lite Leica camera adapted to an inverted microscope (magnification 10×).

Isothiocyanates are known for their chemopreventive and anticancer effect, among other biological activities.^35^ The role of isothiocyanates in bladder cancer has been the subject of numerous investigations.^44^ For instance, some of us have previously reported that several carbohydrate-based analogues of the natural isothiocyanate iberin are selective for the T24 bladder cancer cell line.^45^ Cytotoxicity of the isothiocyanatomethyl ligand **L6** has already been evaluated against eight different human cancer cell lines, with an average IC_50_ of 2.5 ± 1.0 μM,^34^ which is in accordance with the results that we obtained for **Ru6**. And noticeably, **Ru12** exhibits twice the activity of **Ru6** against the lung cancer (A549) and melanoma cells (MeWo), which indicates that the ligand **L6** is probably the responsible for the cytotoxicity, as it is present in one equivalent in the NAMI-Pyr analogue and two in NAMI-A.

Regarding the aldehydes, it is well established that they display a variety of biological activities depending on their structure. They can act as signaling molecules, and form covalent bonds with amine or thiol residues of biomolecules such as DNA and enzymes.^36^ Specifically, the cytotoxic effect of benzaldehyde, a bioisostere of **L2**, has already been described in several cancer cell lines, with mean IC_50_ values of 19, 2.1, 6.4, and 0.43 mM against human normal oral cells, human oral squamous cell carcinoma, human glioblastoma and human myelogenous leukemia respectively.^46^ Therefore, to confirm the cytotoxic activity of **L2** and **L6**, we evaluated their effects under the same cell lines and conditions (Table S13 and Figure S15). **L6** showed a cytotoxicity profile similar to **Ru6** when tested under the same conditions (Table S13 and Figure S15). The same trends exhibited **Ru11** against the melanoma (MeWo) and bladder cancer cells (T24), which almost doubled the activity of **Ru2** and **L2** (Table S13 and Figure S15).

Curiously, **Ru11** was 2.5 times more cytotoxic against lung cancer cells (A549) than **Ru2**. This increase in activity was accompanied by an increase in selectivity, being 7.6 times more cytotoxic to lung cancer cells than to nonmalignant cells. It is interesting to note that 95% cell inhibition was observed for lung cancer cells at a dose of 100 μM, whereas only 10% cell inhibition was observed for the noncancerous cells (Figure 6B). Interestingly, while maintaining a relatively good cytotoxicity (IC_50_ of KP1339 = 126.4 ± 5.8 μM against A549 in similar conditions^47^) **Ru11** showed a higher selective index against A549 cells than gemcitabine and carboplatin. **Ru12** showed higher activity against all cell lines than its NAMI-Pyr analogue **Ru6**, maintaining greater selectivity against bladder cancer cells. In fact, T24 cells were approximately five times more sensitive to **Ru12** than noncancerous cells HaCaT. It is noteworthy that at concentrations close to the IC_50_ value for T24 cells (0.2 μM), approximately 85% cell viability was maintained in noncancerous cells (Figure 6B). Although not as potent as gemcitabine, **Ru12** showed an IC_50_ value against T24 cells in the nM range (240 nM), which is lower than cisplatin (IC_50_ 23.1 ± 1.4 μM for a 48 h exposure against T24 ^48^) and almost a 7000-fold increase in activity compared to NAMI-A.

### UV–Vis Studies

Activation by aquation of NAMI-type complexes is a well-studied process where the chlorines or DMSO are exchanged by hydroxyl groups.^49^ The new labile OH are more reactive,^50^ and it is thought that depending on the hydrolysis degree of NAMI-A, the oxo species can preferentially interact with different biologically relevant molecules.^51^ Since complexes **Ru2**, **Ru6**, **Ru11** and **Ru12** showed enhanced in vitro anticancer activity compared to the parent compounds, we investigated whether this difference could be attributed to a distinct activation.^52^ Thus, we first monitored spectrometrically the hydrolysis process, as each ligand exchange produces a modification in the UV–vis absorbance spectrum. Therefore, the UV–vis spectra of the most cytotoxic complexes were analyzed in phosphate buffered saline solution (PBS) at 37 °C over time (Figures S16 and S17). Starting with the NAMI-Pyr derivatives **Ru2** and **Ru6**, freshly dissolved complexes exhibited two bands peaking at ca. 220 and 240 nm, which along with the shoulder that peaks at 290 nm, are in the expected range for the high energy π → π* electronic transitions of Ru­(III) complexes.^53^ The broader band peaking at ca. 400 nm is typical of ligand-to-metal charge transfer (LMCT) (Figure S16A,C). After the first 20 min of incubation in PBS, the absorbance of these three bands decreased, and a new band appeared at ca. 350 nm, accompanied with an isosbestic point at ca. 370 nm, indicative of the aquation (Figure S16A,C). With longer incubation times, there was a further decrease in the energetic π → π* transition band (Figure S17). Additionally, a hypsochromic shift of the LMCT band peaking at 310 nm was observed, with a second isosbestic point at ca. 340 nm (Figure S16B,D). **Ru11** and **Ru12** exhibited a similar UV–vis hydrolysis pattern as their sodium analogues, maintaining the characteristic bands peaking at the same wavelengths. The only appreciable differences in these UV–vis spectra were a broader band that peaks at ca. 240 nm, and a slower decrease in the π → π* transition band (Figure S16E–H). These results suggest two consecutive ligand exchanges, consistent with the behavior of other NAMI-type complexes already described.^22,28,29,51,54–56^

### Cellular Uptake

Cytotoxic Ru­(III) complexes such as KP1339,^57^ or the Ru­(II) complexes TLD1433 ^58^ and RDC11,^59^ exert their biological activity intracellularly, therefore, their efficacy is closely linked to cellular uptake efficiency. In contrast, NAMI-A binds to proteins of the extracellular matrix and cell membrane, and exhibits low cellular uptake except at high concentrations.^11,60,61^ Interestingly, the cytotoxicity of NAMI-Pyr complexes can be significantly enhanced by their incorporation into liposome-like macrostructures, due to the improved cellular internalization.^19–23^ Therefore, Ru uptake was quantified by ICP–MS for the most cytotoxic complexes **Ru2**, **Ru6**, **Ru11** and **Ru12**, and compared to the noncytotoxic **Ru3** and NAMI-A as controls (Table S14) for the A549 and T24 cell lines.

Although all values were close to the detection limit, higher uptake was detected in T24 cells, with **Ru2** showing the greatest uptake among the tested complexes. Nevertheless, with less than 0.1% of the administered Ru detected, the uptake profile was consistent with these of NAMI-type complexes. Given the marginal cellular accumulation, and the fact that the antiproliferative activities of **Ru2** and **Ru6** are in the same range as **L2** and **L6** respectively, we conclude that the cytotoxic effects of **Ru2**, **Ru6**, **Ru11** and **Ru12** likely originate from the substituted pyridine.

### Assessing the Contribution of ROS to the Selective Cytotoxicity of Ru­(III) Complexes

Evidence supports a crucial role for reactive oxygen species (ROS) in mediating the cytotoxic mechanism of several clinically useful and emerging anticancer drugs.^62^ On that account, we evaluated whether ROS generation was involved in the cytotoxicity of our complexes. We selected four representative compounds for this purpose: **Ru3** and **Ru5** with low cytotoxicity, and **Ru2** and **Ru6** with high cytotoxic and selective activity. A549 and T24 cells were exposed to these compounds in the presence or absence of the antioxidants catalase and *N*-acetylcysteine (NAC) (Figures 7 and S18).

**Figure 7:**
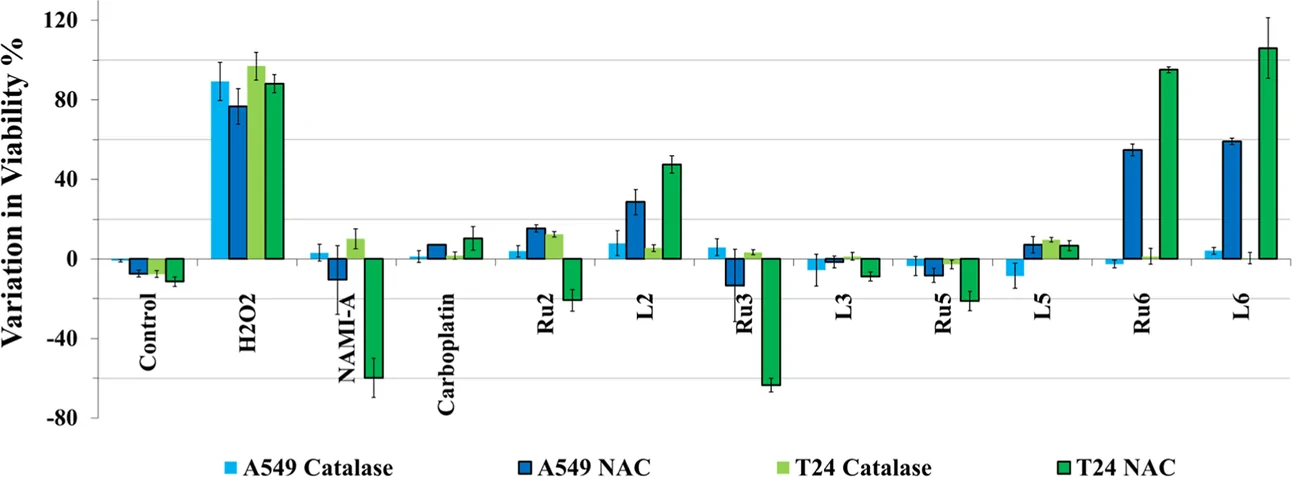
Assessment of the contribution of ROS generation to the cytotoxicity of the ruthenium complexes. A549 cells (blue) were treated with **Ru2** (100 μM), **L2** (100 μM), **Ru3** (1000 μM), **L3** (1000 μM), **Ru5** (1000 μM), **L5** (1000 μM), **Ru6** (10 μM), **L6** (10 μM), NAMI-A (1000 μM), H_2_O_2_ (100 μM), or carboplatin (100 μM) and T24 cells (green) were treated with **Ru2** (1000 μM), **L2** (1000 μM), **Ru3** (1000 μM), **L3** (1000 μM), **Ru5** (1000 μM), **L5** (1000 μM), **Ru6** (3.2 μM), **L6** (3.2 μM), NAMI-A (1000 μM), H_2_O_2_ (100 μM), or carboplatin (100 μM),for 72 h in the absence or presence of catalase (light) or *N*-acetylcysteine (NAC, dark). Data represent mean ± SEM from at least three independent experiments.

Incubation with either antioxidants reduced the cytotoxicity induced by H_2_O_2_ (positive control). However, the cytotoxic effects of the Ru­(III) complexes and their corresponding ligands were not mitigated by catalase. In contrast, the effects of NAC were compound dependent. For NAMI-A, **Ru2**, **Ru3** and **Ru5**, the cytotoxic effect on T24 cells was enhanced in the presence of NAC, at the highest concentration of complex tested. Given that the activity of the free ligands **L3** and **L5** was unaltered by NAC, this enhancement can be attributed to the reduction of Ru­(III) to the more active Ru­(II) species within the reducing environment generated by NAC.^11^ The variation in viability of the complexes was less pronounced in the A549 cell line, consistent with the lower Ru internalization observed for this cell line. Nonetheless, similar results were observed in A549 cells, except for **Ru2**, **L2** and **Ru11** (Figure S18), which showed increased viability in the presence of NAC. This difference could be related to the selectivity exhibited by the complex and ligand toward this cell line.

A549 cells are known to overexpress aldehyde dehydrogenase (ALDH) enzymes,^63,64^ which may contribute to the distinct response observed for the aldehyde containing compounds **L2**, **Ru2** and **Ru11**. ALDH enzymes constitute a NAD­(P)^+^-dependent intracellular superfamily of 19 human isoforms that catalyze the oxidation of endogenous and exogenous aldehydes to their corresponding carboxylic acids. Given the structural similarity of **L2** to aromatic aldehydes, which are known substrates for various ALDH isoforms, we performed exploratory molecular docking studies to investigate potential interactions. However, these preliminary calculations were inconclusive and do not provide definitive mechanistic evidence (see Docking Studies section in the Supporting Information for additional details).^63,64^ The protective effect of NAC observed for **L2** and **Ru11** may be attributed to its ability to reduce the oxidative stress, or to direct chemical scavenging of the electrophilic aldehyde moiety of **L2** by the nucleophilic thiol group of NAC forming an inert adduct (Figure S18). On the other hand, preincubation with NAC completely abolished the cytotoxic effect of **Ru6** and its corresponding ligand **L6** in both cancer cell lines. The isothiocyanate moiety of **L6**, as in other isothiocyanates with anticancer activity, could induce cell death by directly conjugating with and depleting the cellular pool of glutathione (GSH).^65^ The high reactivity of isothiocyanate groups toward thiols could lead to an abrupt intracellular glutathione depletion, disrupting their redox balance and the ability to quickly scavenge newly produced ROS. The loss of this critical antioxidant leads to a significant accumulation of ROS and lethal oxidative stress.^62^ NAC prevents this by acting as a GSH substitute, scavenging the ligand **L6** directly and thereby preserving the intracellular glutathione levels required for cell survival. Similar findings were obtained for **Ru12** (Figure S18). According to recent results obtained from some of us, T24 cells are more sensitive than A549, MeWo and HaCaT to isothiocyanates and changes in ROS levels.^45^ In fact, under our experimental conditions with the antioxidants, the viability of T24 cells treated with H_2_O_2_ increased more than the viability of A549 cells.

### Effect of Ru­(III) Complexes on Cell Migration

As previously mentioned, NAMI-A has not exhibited cytotoxicity against most cancer cell lines but has demonstrated notable antimetastatic activity.^11^ Targeting metastasis as a therapeutic approach is crucial, as it remains the leading cause of cancer-related mortality.^66^ And tumor cell migration is an important step in this process. Among our Ru­(III) complexes tested, **Ru2** and **Ru6** (and their NAMI-A counterparts) showed the highest cytotoxicity. Since **L2** and **L6** are likely responsible for their cytotoxic effects, we assessed whether **Ru2** and **Ru6** also displayed antimetastatic activity, as expected for this family of compounds. In addition, we evaluated two other complexes that exhibited low cytotoxicity (**Ru3** and **Ru5**).

To investigate the inhibitory effects on cell migration, a wound healing assay was performed (Figure 8).^67^ Bladder cancer cells (T24) were treated with the maximum nontoxic concentrations for 24 h, based on the previously obtained cell viability curves: NAMI-A (100 μM), **Ru2** (20 μM), **Ru3** (100 μM), **Ru5** (100 μM) and **Ru6** (0.3 μM). The same concentrations were used to evaluate the activity of the corresponding ligands (Figure S25). All complexes tested, with the exception of **Ru2**, showed reduced wound closure compared to untreated cells. In contrast, cells treated with the ligands showed wound closure comparable to untreated cells, except for **L2** (Figure S25). These results indicate that the inhibition of cell migration is due to the Ru­(III) complexes rather than their free ligands. The increase in migration of cells treated with 20 μM of **Ru2** or **L2** could be related to a nonlethal accumulation of toxic aldehydes. These aldehydes can cause DNA damage and chromosomal instability, which in turn increases the migration capacity of cancer cells.^68^ Noteworthy, this effect occurs only at low doses. At higher concentrations of **Ru2** or **L2**, aldehyde accumulation is expected to be cytotoxic, as observed in Tables 1, 2 and S13 and Figures 4, 6 and S15.

**Figure 8:**
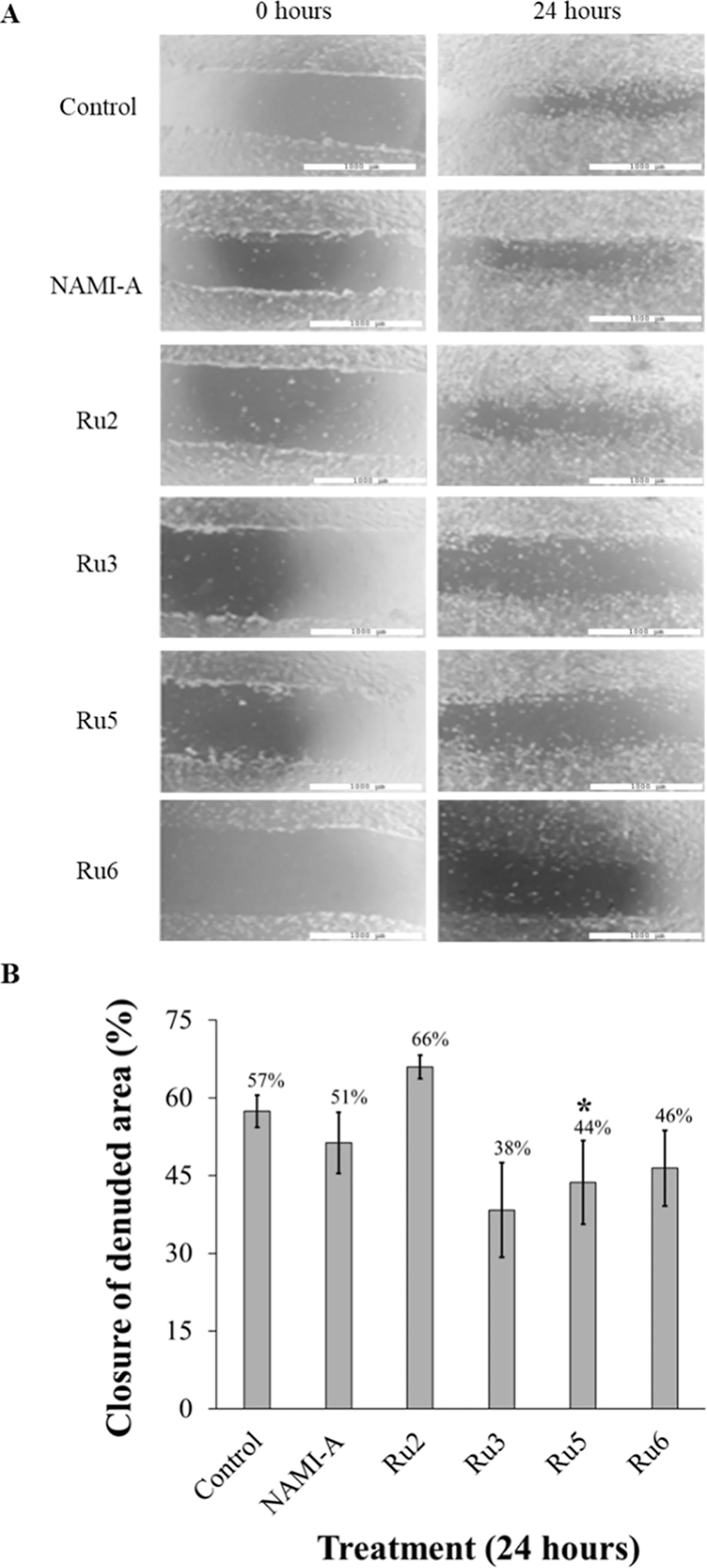
Effects of NAMI-A, **Ru2**, **Ru3**, **Ru5** and **Ru6** on migration of bladder cancer cells. T24 cells were seeded in a 6-well plate and incubated overnight. A wound area was generated, and cells were left untreated or treated for 24 h with NAMI-A (100 μM), **Ru2** (20 μM), **Ru3** (100 μM), **Ru5** (100 μM) and **Ru6** (0.3 μM). (A) Representative photographs at 4× magnification. (B) Quantification of the wound area. The closure of denuded area (%) was calculated as the ratio of the remaining wound area at 24 h to the original wound area created at 0 h. The data represent the means ± SEM of two independent experiments.

On the other hand, **Ru3** and **Ru5** were the most active, exhibiting the strongest inhibition of wound closure. This effect could be attributed to the higher nontoxic concentrations used for these compounds, which would result in a higher concentration of Ru. Therefore, our findings suggest that these new complexes, could have antimetastatic properties similar to NAMI-A.

## Conclusions

Ru­(III) complexes have emerged as promising alternatives to the conventional Pt­(II) chemotherapeutic agents, especially NAMI-A. However, its development was halted after phase II clinical studies determined “insufficiently active for further use”. This limitation motivated this work, leading to the synthesis and characterization of 12 new NAMI/NAMI-A-type complexes featuring modified pyridines at positions 3 or 4 as the heterocyclic ligand. Moreover, their cytotoxic activity was assessed in vitro against 4 different cell lines. Notably, complexes **Ru2** and **Ru11**, both featuring an aldehyde at position 3 of the pyridine, stood out with good activity and selectivity against A549 cancer cells. **Ru11**, in particular, with an IC_50_ of 34 μM and S.I. of 7.6, exhibited a higher selective index than gemcitabine and carboplatin, with lower IC_50_ than KP1339 against the same cell line. Conversely, **Ru6** and **Ru12**, bearing an isothiocyanatomethyl group at position 3, showed high cytotoxic activity against T24 cancer cells. **Ru12** showed an IC_5_ 240 nM and S.I. of 5.2, exhibiting 85 times more activity than carboplatin and being more cytotoxic than cisplatin under similar conditions.

These Ru­(III) compounds retained the characteristic hydrolytic behavior and low cellular uptake observed for the NAMI-type family. Therefore, the enhanced cytotoxicity is strongly influenced by the reactivity of the pyridines **L2** and **L6**, which exert antitumor activity through ROS-mediated toxicity.

Importantly, as the NAMI core structure was preserved, and supported by the wound healing migration assays, the results indicate that antimetastatic properties are largely retained for most complexes, with exception of **Ru2**. An increased migration was observed for the aldehydes containing **L2** and **Ru2** at the tested noncytotoxic dose. In summary, these findings identify that the new complexes **Ru2**, **Ru6**, **Ru11** and **Ru12** emerge as the most promising candidates, combining enhanced cytotoxicity with preserved NAMI-type behavior, including low uptake and antimetastatic activity for most complexes. Their ligand-driven biological effects and selective activity against specific cancer lines highlight their therapeutic potential. We hope that these findings pave the way for the reclassification of this family of complexes as “sufficiently active for further use” and worthy of renewed attention in anticancer drug development.

## Experimental Section

### Materials

Chemicals and solvents were used as received from commercial sources. Anhydrous HPLC grade solvents were used. Thin layer chromatography was carried out on silica gel 60 F254 analytical plates from Merck. Reactions were monitored with UV lamp (254 nm) and revealed with solutions of phosphomolybdic acid or ninhydrin 5% in EtOH. Merck 230–400 mesh silica gel was used for column chromatography and eluents are indicated as volume-to-volume ratio of solvents. Proton nuclear magnetic resonance spectra (^1^H NMR) were obtained with a BRUKER AVANCE III 300 MHz spectrometer. Chemical shifts (δ) are reported in ppm and referred to solvent residual protons coupling constants (*J*) are reported in hertz. Spectra of the complexes were recorded with a spectral window of 40 ppm centered at −5 ppm, 8 μs 90° pulse and 128 scans. Low resolution mass spectra (LRMS) and high resolution mass spectra (HRMS) were recorded with a Thermo Scientific Orbitrap Elite with electrospray ionization in negative ion mode (ESI^–^). UV–vis spectra were recorded using a Shimadzu UV-1280 UV–vis spectrophotometer by using disposable brand UV-Cuvette micro (12.5 × 12.5 × 45 mm). Milli-Q water (18.2 MΩ·cm, Merck Milli-Q Advantage, Darmstadt, Germany) was used for HPLC measurements. Purity of the final compounds was determined by analytical HPLC. Analyses were performed either on a Thermo Scientific Dionex UltiMate 3000 UHPLC-system equipped with a Waters Acquity UPLC BEH C18 1.7 μm 3.0 × 50 mm column using a 5–95% (v/v) acetonitrile/water gradient over 5 min at a flow rate of 0.6 mL/min or on a Shimadzu LC-2030C Plus 3D RoHS-Prominence-I HPLC system equipped with a Phenomenex Luna C18 5 μm 250 × 4.6 mm column using a 5–95% (v/v) acetonitrile/water gradient over 10 min at a flow rate of 1.0 mL/min. Acetonitrile and Milli-Q water used as eluents contained 0.1% (v/v) of trifluoroacetic acid, and elution was monitored at 220 nm. Detailed chromatographic conditions and chromatograms are provided in the Supporting Information. HPLC analysis indicated a purity of ≥95% for all complexes used for biological evaluation. Elemental analysis (C, H, N, weight %) data was obtained using an Elemental LECO TruSpec CHNS micro analyzer at the Centro de Investigaciones, Tecnología e Innovación (CITIUS) of the University of Sevilla. Inductively coupled plasma mass spectrometry (ICP–MS) was used to determine Ru concentration using a PerkinElmer ICP–MS NexION 300D with universal at Servicios Centrales de Apoyo a la Investigación (SCAI) of the University of Málaga.

### Synthetic Methods

#### *tert*-Butyl (Pyridin-3-ylmethyl)­carbamate (**L4**)

Di-*tert*-butyldicarbonate (480 mg, 2.2 mmol), K_2_CO_3_ (207 mg, 3 mmol), 10 mL acetone and pyridin-3-ylmethanamine (203 μL, 2 mmol) were stirred overnight at room temperature (Scheme S1). The solvent was removed under vacuum and the crude was distributed between saturated aqueous solution of NH_4_Cl and CH_2_Cl_2_. The biphasic mixture was separated, and the aqueous phase washed twice with CH_2_Cl_2_. The combined organic phases were dried over Na_2_SO_4_, evaporated, and purified by column chromatography hexane/EtOAc from 1:2 to 1:3. Yellow liquid (291 mg, 70%); *R*_f_ = 0.3 (hexane/EtOAc 1:2). ^1^H NMR (300 MHz, CDCl_3_): δ (ppm) 8.56 (d, *J* = 1.6 Hz, 1H), 8.52 (dd, *J* = 4.9, 1.4 Hz, 1H), 7.68 (d, *J* = 7.7 Hz, 1H), 7.30 (dd, *J* = 7.8, 4.9 Hz, 1H), 5.01 (s, 1H), 4.34 (d, *J* = 5.9 Hz, 2H), 1.45 (s, 9H).

#### 3-(Isothiocyanatomethyl)­pyridine (**L6**)

**L6** was prepared following a procedure already reported.^69^ Briefly, a solution of 3-(aminomethyl)­pyridine (102 μL, 1 mmol) and triethylamine (505 μL, 4 mmol) in dry CH_2_Cl_2_ (5 mL) was cooled to 0 °C under Ar atmosphere. Thiophosgene (85 μL, 1.1 mmol) dissolved in dry CH_2_Cl_2_ (1.7 mL) was then added dropwise, under vigorous stirring, into the first solution (Scheme S2). The dark mixture was left stirring at room temperature until the amine was completely consumed (around 30 min). The solvent was evaporated, and the crude purified by column chromatography CH_2_Cl_2_/CH_3_OH 30:1 to obtain **L6** as a yellow oil (134 mg, 89%). *R*_f_ = 0.75 (CH_2_Cl_2_/CH_3_OH 15:1). ^1^H NMR (300 MHz, CDCl_3_): δ (ppm) 8.61 (dd, *J* = 4.8, 1.3 Hz, 1H), 8.58 (d, *J* = 2.0 Hz, 1H), 7.70 (ddd, *J* = 7.9, 2.2, 1.7 Hz, 1H), 7.35 (dd, *J* = 7.9, 4.9 Hz, 1H), 4.75 (s, 2H).

#### 4-(Azidomethyl)­pyridine (**L8**)

4-(Bromomethyl)­pyridine hydrobromide (258 mg, 1 mmol) and K_2_CO_3_ (140 mg, 1 mmol) were dissolved in 2.5 mL of *N*,*N*-dimethylformamide (DMF). After stirring for 15 min, sodium azide (98 mg, 1.5 mmol) was added and the mixture was stirred for 3 h (Scheme S3). After completion, a saturated NaHCO_3_ solution was poured slowly, followed by diethyl ether (Et_2_O). The biphasic mixture was separated, and the aqueous layer was extracted once with Et_2_O. The combined organic phases were washed twice with distilled water, and finally 3 mL of HCl (1 M) were added. The new biphasic mixture was evaporated completely to obtain the corresponding hydrochloride **L8** as a yellowish solid (155 mg, 91%). *R*_f_ = 0.65 (hexane/EtOAc, 1:1).^1^H NMR (300 MHz, D_2_O): δ (ppm) 8.79 (d, *J* = 6.7 Hz, 2H), 8.09 (d, *J* = 6.1 Hz, 2H), 4.92 (s, 2H).

The pyridine **L8** must be deprotonated before being used as a ligand. Therefore, the corresponding hydrochloride and an excess of K_2_CO_3_ were stirred in CH_2_Cl_2_. After 5 min, the insoluble salts were filtered out, and almost all solvent was evaporated.

#### *tert*-Butyl (Pyridin-4-ylmethyl)­carbamate (**L9**)

Following the procedure described for **L4** but using pyridin-4-ylmethanamine as the starting material (Scheme S4). Yellow wax (195 mg, 47%); *R*_f_ = 0.3 (hexane/EtOAc 1:2). ^1^H NMR (300 MHz, CD_3_OD): δ (ppm) 8.46 (d, *J* = 6.1 Hz, 2H), 7.34 (d, *J* = 6.0 Hz, 2H), 4.28 (s, 2H), 1.46 (s, 9H).

#### (DMSO)_2_H­[*trans*-RuCl_4_(dmso-*S*)_2_] **(Ru–P1)**

A slightly modified procedure reported by Alessio was followed (Scheme S5).^70^ Commercially hydrated RuCl_3_ (1.5 g 5.74 mmol) was stirred at 80 °C with 7 mL of DMSO and 1 mL of 35% aqueous HCl for ca. 20 min, until it was completely dissolved. The obtained dark brown solution was then stirred at 105 °C for 15 min. The dark orange solution was left to cool to room temperature before adding 30 mL of acetone. Dark red crystals appeared after cooling the crude at 4 °C overnight. The crystals were filtered, washed with acetone and Et_2_O, and vacuum-dried to obtain **Ru–P1** (2.45 g, 76%).

#### Na­[*trans*-RuCl_4_(dmso-*S*)_2_] **(Ru–P2)**

The synthesis of **Ru–P2** was carried out following a protocol described by Alessio (Scheme S5).^70^ Briefly, upon addition of a solution of NaCl (315 mg, 5.39 mmol) in H_2_O (0.9 mL) to a solution of **Ru–P1** (2 g, 3.59 mmol) in a mixture of EtOH (90 mL) and H_2_O (1.25 mL), orange crystals immediately appeared. **Ru–P2** was filtered, washed with EtOH and Et_2_O, and vacuum-dried (1.2 g, 80%).

#### (ImH)­[*trans*-RuCl_4_(dmso-*S*) (Im)] **NAMI-A**

The synthesis was carried out following a procedure already described.^71^ Briefly, to a suspension of **Ru–P1** (100 mg, 0.18 mmol) in acetone (1 mL) was added a solution of imidazole (49 mg, 0.72 mmol) in acetone (1 mL). The mixture was stirred at room temperature overnight. The brick red solid that formed was filtered, washed with acetone and Et_2_O, and dried to obtain the desired product (72 mg, 87%). *R*_f_ = 0.4 (CH_2_Cl_2_/CH_3_OH 5:1). ^1^H NMR (300 MHz, DMSO-*d*_6_): δ (ppm) 9.09 (s, 1H), 7.74 (s, 2H), −2.31 (br s), −13.23 (br s). LRMS (ESI^–^) *m*/*z* (%): 389.83 (M^–^, 2), 321.79 (M^–^–Im, 3), 243.78 (M^–^–Im–DMSO, 100).

### General Protocol for the Synthesis of NAMI-Pyr Analogues

A solution of the corresponding ligand (0.26 mmol) in acetone (2 mL) was added dropwise to a suspension of **Ru–P2** (100 mg, 0.237 mmol) in acetone (4 mL) (Figure S1). The mixture was stirred at room temperature until the ruthenium precursor was completely consumed. The clear orange solution was evaporated to ∼1 mL, and the product was precipitated with Et_2_O. The fine powder was filtered, washed with Et_2_O, and vacuum-dried.

#### Na­[*trans*-Ru­(BrPy)­Cl_4_(dmso-*S*)] **(Ru1)**

Orange solid (117 mg, quant.). *R*_f_ = 0.4 (CH_2_Cl_2_/CH_3_OH 5:1). ^1^H NMR (300 MHz, DMSO-*d*_6_): δ (ppm) −0.51 (br s), −1.58 (br s), −12.64 (br s). LRMS (ESI^–^) *m*/*z* (%): 478.74 (M^–^, 9), 321.79 (M^–^–BrPy, 3), 243.78 (M^–^–BrPy–DMSO, 100). HRMS (ESI^–^): calcd for C_7_H_10_ON_4_^79^BrCl_4_RuS, 476.7470; found, 476.7448 (−4.5958 ppm), calcd for C_7_H_10_ON_4_^81^BrCl_4_RuS, 478.7449; found, 478.7435 (−2.9022 ppm).

#### Na­[*trans*-Ru­(CHO-Py)­Cl_4_ (dmso-*S*)] **(Ru2)**

Bright orange solid (104 mg, 97%). *R*_f_ = 0.35 (CH_2_Cl_2_/CH_3_OH 5:1). ^1^H NMR (300 MHz, DMSO-*d*_6_): δ (ppm) 6.75 (br s), 6.41 (br s), −1.13 (br s), −12.58 (br s). LRMS (ESI^–^) *m*/*z* (%): 428.83 (M^–^, 8), 321.79 (M^–^–CHOPy, 4), 243.78 (M^–^–CHOPy–DMSO, 100). HRMS (ESI^–^): calcd for C_8_H_11_O_2_NCl_4_RuS, 426.8314; found, 426.8295 (−4.2705 ppm).

#### Na­[*trans*-RuCl_4_(dmso-*S*)­(OH-Py)] **(Ru3)**

Orange solid (101 mg, 94%). *R*_f_ = 0.3 (CH_2_Cl_2_/CH_3_OH 5:1). ^1^H NMR (300 MHz, DMSO-*d*_6_): δ (ppm) 5.57 (br s), 3.43 (br s), 2.02 (br s), −1.98 (br s), −12.63 (br s). LRMS (ESI^–^) *m*/*z* (%): 455.85 (M^–^, 5), 321.79 (M^–^–OHPy, 2), 243.78 (M^–^–OHPy–DMSO, 100). HRMS (ESI^–^): calcd for C_8_H_13_O_2_NCl_4_RuS, 428.8470; found, 428.8451 (−4.4050 ppm).

#### Na­[*trans*-RuCl_4_(dmso-*S*)­(3-NBOC-Py)] **(Ru4)**

Orange solid (130 mg, quant.). *R*_f_ = 0.3 (CH_2_Cl_2_/CH_3_OH 5:1). ^1^H NMR (300 MHz, DMSO-*d*_6_): δ (ppm) 5.58 (br s), 1.28 (br s), −1.98 (br s), −12.87 (br s). LRMS (ESI^–^) *m*/*z* (%): 529.91 (M^–^, 16), 321.79 (M^–^–3-NBOCPy, 5), 243.78 (M^–^–3-NBOCPy–DMSO, 100). HRMS (ESI^–^): calcd for C_13_H_22_O_3_N_2_Cl_4_RuS, 527.9154; found, 527.9138 (−3.0103 ppm).

#### Na­[*trans*-RuCl_4_(dmso-*S*)­(NCS-Py)] **(Ru6)**

Dark yellow solid (117 mg, quant.). *R*_f_ = 0.6 (CH_2_Cl_2_/CH_3_OH 5:1). ^1^H NMR (300 MHz, DMSO-*d*_6_): δ (ppm) 6.09 (br s), −1.75 (br s), −12.55 (br s). LRMS (ESI^–^) *m*/*z* (%): 471.82 (M^–^, 5), 321.79 (M^–^–NCSPy, 4), 243.78 (M–NCSPy–DMSO, 100). HRMS (ESI^–^): calcd for C_9_H_12_ON_2_Cl_4_RuS_2_, 469.8194; found, 469.8176 (−3.9191 ppm).

#### Na­[*trans*-RuCl_4_(CO_2_Et)­(dmso-*S*)] **(Ru7)**

Dark yellow solid (117 mg, quant.). *R*_f_ = 0.5 (CH_2_Cl_2_/CH_3_OH 5:1). ^1^H NMR (300 MHz, DMSO-*d*_6_): δ (ppm) 2.93 (br s), 0.36 (br s), −1.08 (br s), −12.40 (br s). LRMS (ESI^–^) *m*/*z* (%): 472.85 (M^–^, 6), 321.79 (M^–^–CO_2_EtPy, 4), 243.78 (M^–^–CO_2_EtPy–DMSO, 100). HRMS (ESI^–^): calcd for C_10_H_15_O_3_NCl_4_RuS, 470.8576; found, 470.8557 (−3.9415 ppm).

#### Na­[*trans*-RuCl_4_(dmso-*S*)­(N_3_–Py)] **(Ru8)**

Dark orange solid (99 mg, 87%). *R*_f_ = 0.5 (CH_2_Cl_2_/CH_3_OH 5:1). ^1^H NMR (300 MHz, DMSO-*d*_6_): δ (ppm) 6.85 (br s), −1.84 (br s), −12.70 (br s). LRMS (ESI^–^) *m*/*z* (%): 455.85 (M^–^, 5), 321.79 (M^–^–N_3_Py, 4), 243.78 (M^–^–N_3_Py–DMSO, 100). HRMS (ESI^–^): calcd for C_8_H_12_ON_4_Cl_4_RuS, 453.8535; found, 453.8518 (−3.8253 ppm).

#### Na­[*trans*-RuCl_4_(dmso-*S*)­(4-NBOC-Py)] **(Ru9)**

Dark orange solid (104 mg, 79%). *R*_f_ = 0.3 (CH_2_Cl_2_/CH_3_OH 5:1). ^1^H NMR (300 MHz, DMSO-*d*_6_): δ (ppm) 5.52 (br s), 3.29 (br s), 0.82 (br s), −1.23 (br s), −1.81 (br s), −12.64 (br s). LRMS (ESI^–^) *m*/*z* (%): 529.91 (M^–^, 10), 321.79 (M^–^–4-NBOCPy, 5), 243.78 (M^–^–4-NBOCPy–DMSO, 100). HRMS (ESI^–^): calcd for C_13_H_22_O_3_N_2_Cl_4_RuS, 527.9143; found, 527.9141 (−0.4598 ppm).

### General Protocol for the Amine Deprotection

To a solution of the corresponding complex (65 mg, 0.118 mmol) in acetone (4 mL) 90 μL of HCl (35%) were added (Scheme S6), and the orange solution became cloudy almost instantly. The mixture was left stirring at room temperature overnight. The product was filtered, washed with acetone and Et_2_O, and vacuum-dried.

#### [*trans*-RuCl_4_(dmso-*S*)­(3-NH_3_Py)] **(Ru5)**

Pale orange solid (44 mg, 87%). *R*_f_ = 0.1 (CH_2_Cl_2_/CH_3_OH 5:1). ^1^H NMR (300 MHz, DMSO-*d*_6_): δ (ppm) 6.35 (br s), 1.59 (br s), −1.69 (br s), −12.54 (br s). LRMS (ESI^–^) *m*/*z* (%): 429.86 (M^–^, 39), 321.79 (M^–^–NH_2_Py, 6), 243.78 (M^–^–NH_2_Py–DMSO, 83). HRMS (ESI^–^): calcd for C_8_H_14_ON_2_Cl_4_RuS, 427.8630; found, 427.8614 (−3.6182 ppm).

#### [*trans*-RuCl_4_(dmso-*S*)­(4-NH_3_Py)] **(Ru10)**

Orange solid (36 mg, 72%). *R*_f_ = 0.1 (CH_2_Cl_2_/CH_3_OH 5:1). ^1^H NMR (300 MHz, DMSO-*d*_6_): δ (ppm) 6.22 (br s), −0.79 (br s), −1.45 (br s), −12.71 (br s). LRMS (ESI^–^) *m*/*z* (%): 429.86 (M^–^, 39), 321.79 (M^–^–NH_2_Py, 6), 243.78 (M^–^–NH_2_Py–DMSO, 100). HRMS (ESI^–^): calcd for C_8_H_14_ON_2_Cl_4_RuS, 427.8619; found, 427.8617 (−0.3690 ppm).

### General Protocol for the Synthesis of NAMI-A Analogues

To a suspension of **Ru–P1** (100 mg, 0.18 mmol) in acetone (3 mL) was added a solution of the corresponding ligand (0.54 mmol) in acetone (3 mL) (Scheme S7). The mixture was stirred at room temperature overnight. Et_2_O was added and the reaction mixture was kept at −20 °C to facilitate the product precipitation. The solid was filtered, washed with Et_2_O and vacuum-dried.

#### (CHO-PyH)­[*trans*-Ru­(CHO-Py)­Cl_4_ (dmso-*S*)] **(Ru11)**

Pale yellow solid (86 mg, 89%). *R*_f_ = 0.4 (CH_2_Cl_2_/CH_3_OH 5:1). ^1^H NMR (300 MHz, DMSO-*d*_6_): δ (ppm) 10.17 (s, 1H), 9.20 (s, 1H), 8.95 (s, 1H), 8.48 (s, 1H), 7.83 (s, 1H), 6.73 (br s), 6.42 (br s), −1.13 (br s), −12.55 (br s). LRMS (ESI^–^) *m*/*z* (%): 428.83 (M^–^, 15), 321.79 (M^–^–CHOPy, 5), 243.78 (M^–^–CHOPy–DMSO, 100). HRMS (ESI^–^): calcd for C_8_H_11_O_2_NCl_4_RuS, 426.8314; found, 426.8299 (−3.3945 ppm). EA for C_14_H_17_C_l4_N_2_O_3_RuS·2H_2_O theoretical 29.38% C, 3.70% H, 4.90% N. Experimental 29.38% C, 3.42% H, 4.81% N.

#### (NCS-PyH)­[*trans*-RuCl_4_(dmso-*S*)­(NCS-Py)] **(Ru12)**

Pale orange solid (103 mg, 92%). *R*_f_ = 0.55 (CH_2_Cl_2_/CH_3_OH 5:1). ^1^H NMR (300 MHz, DMSO-*d*_6_): δ (ppm) 8.87 (d, *J* = 18.0 Hz, 2H), 8.42 (s, 1H), 7.95 (s, 1H), 6.80 (br s), 6.09 (br s), 5.16 (s, 2H), −1.75 (br s), −12.51 (br s). LRMS (ESI^–^) *m*/*z* (%): 471.82 (M^–^, 20), 321.79 (M^–^–NCSPy, 7), 243.78 (M^–^–NCSPy–DMSO, 100). HRMS (ESI^–^): calcd for C_9_H_12_ON_2_Cl_4_RuS_2_, 469.8194; found, 469.8178 (−3.4875 ppm). EA for C_16_H_19_C_l4_N_4_ORuS_3_·H_2_O theoretical 30.01% C, 3.31% H, 8.75% N. Experimental 30.12% C, 3.29% H, 8.65% N.

### Crystallization

Unless otherwise indicated, suitable crystals for X-ray diffraction were obtained as follows. The complex was dissolved in acetone, Et_2_O was added until mild turbidity was observed, and the solution was cooled at −20 °C for at least 48 h. Crystals for complexes **Ru6**, **Ru10** and **Ru11** were obtained following the same procedure but using DMF instead of acetone.

### Single-Crystal X-ray Diffraction Materials and Methods

A summary of the crystallographic data and the structure refinement results for compounds **Ru1** to **Ru12** is given in the Supporting Information Tables S1–S12. Crystals of a suitable size for X-ray diffraction analysis were coated with dry perfluoropolyether and mounted on glass fibers and fixed in a cold nitrogen stream (*T* = 193 K) to the goniometer head. Data collection was carried out on a Bruker-AXS, D8 QUEST ECO, PHOTON II area detector diffractometer, using monochromatic radiation λ­(Mo Kα) = 0.71073 Å, by means of ω and φ scans with a width of 0.50°. The data were reduced (SAINT^72^) and corrected for absorption effects by the multiscan method (SADABS^73^). The structures were solved by intrinsic phasing modification of direct methods (SHELXT^74^) and refined against all *F* ^2^ data by full-matrix least-squares techniques (SHELXL-2018/3 ^75^) minimizing *w*[*F*_o_^2^ – *F*_c_^2^]^2^. All non-hydrogen atoms were refined anisotropically. The hydrogen atoms were included from calculated positions and refined riding on their respective carbon atoms with isotropic displacement parameters. The corresponding crystallographic data were deposited with the Cambridge Crystallographic Data Centre as supplementary publications. CCDC 2371342 (**Ru1**), CCDC 2371343 (**Ru2**), CCDC 2371344 (**Ru3**), CCDC 2371345 (**Ru4**), CCDC 2371346 (**Ru5**), CCDC 2371347 (**Ru6**), CCDC 2371348 (**Ru7**), CCDC 2371349 (**Ru8**), CCDC 2371350 (**Ru9**), CCDC 2371351 (**Ru10**), CCDC 2371352 (**Ru11**) and CCDC 2371353 (**Ru12**), contain the supplementary crystallographic data for this paper. The data can be obtained free of charge via: https://www.ccdc.cam.ac.uk/structures/.

### Cell Lines

A549 (human nonsmall cell lung cancer cells), T24 (human bladder cancer cells), MeWo (human BRAF wild-type melanoma cells) and HaCaT cells (human keratinocytes^76^) were purchased from Cell Lines Service (CLS). Cells were maintained in Dulbecco’s modified Eagle’s medium (DMEM) that contained l-glutamine and high glucose levels. The medium was supplemented with 100 U/mL penicillin, 100 μg/mL streptomycin and 10% fetal bovine serum. All cells were cultured at 37 °C in a humidified atmosphere of 5% CO_2_. All cell culture reagents were obtained from Biowest and Thermo Fisher Scientific.

### Cell Viability Assay

Cell viability was determined with the resazurin assay, a quantitative colorimetric method. This assay is based on the reduction by living cells of the oxidized blue reagent resazurin to a pink resorufin product. Only viable cells with active metabolism can reduce resazurin, therefore, the amount of resorufin produced is proportional to the number of viable cells. In this work, exponentially growing cells (3000–5000 cells per well) were seeded in 96-well plates and allowed to grow for 24 h. The cells were then exposed to several concentrations of the new Ru­(III) complexes or the anticancer drugs carboplatin and gemcitabine. Specifically, the complexes were first fully dissolved in DMSO, then diluted in the culture medium, and promptly added to the cells. For the determination of the contribution of ROS to the selective cytotoxicity of the complexes, antioxidants were added 1 h before compounds. After a 72 h treatment period, the medium was removed and the cells were washed once with phosphate buffered saline. Next, 150 μL of resazurin (final concentration: 20 μg/mL) was added to each well and, after a 5 h incubation period at 37 °C and 5% CO_2_, optical densities were measured at 540 and 620 nm using a multiwell plate spectrophotometer reader. Cell viability was calculated as a percentage in relation to controls (untreated cells). Dose–response curves were analyzed in GraphPad Prism using nonlinear regression. IC_50_ values are presented as the means and their corresponding 95% confidence intervals (95% CI). Variation in viability % was calculated as the difference in the % of cell viability between cells treated in the presence of antioxidants and cells treated in the absence of antioxidants. Data are from at least three independent experiments and are expressed as the means ± standard error of the mean (SEM).

For statistical analysis, the cytotoxicity of a particular concentration of the compound was compared between nonmalignant HaCaT cells and a particular cancer cell line. The *t*-test (paired, two-tailed) was used. A *p* value >0.05 is not considered statistically significant and is not represented by any symbol. A *p* value <0.05 is considered statistically significant and is indicated with an asterisk (*), a *p* value <0.01 is indicated with a double asterisk (**), and a *p* value <0.001 is indicated with a triple asterisk (***).

The selective index values (S.I.) were calculated to analyze the selective anticancer activity of the compounds. S.I. values were obtained by dividing the IC_50_ values in nonmalignant HaCaT cells by those of cancer cells.

### UV–Vis Studies

20 mL of a 0.1 mM solution of the corresponding complex in PBS was placed in a water bath at 37 °C. 700 μL of the solution were taken, and the absorbance from 200 to 600 nm was measured at the indicated times, with the scan speed selected at medium.

### Cellular Uptake

Cells were seeded at a density of 150,000 cells/well in 12-well plates and incubated 24 h at nontoxic complex concentrations: **Ru2** (20 μM), **Ru3** (20 μM), **Ru6** (0.3 μM), **Ru11** (20 μM), **Ru12** (0.3 μM) and NAMI-A (20 μM). Subsequently, the cells were exposed to the complexes for 24 h. Following treatment, the cells were detached using trypsin–EDTA for 6 min. After neutralizing the trypsin with culture medium, the cells were counted for each sample. The cell suspension was then centrifuged at 300*g* for 4 min at 4 °C; the supernatant was discarded, and the cell pellet was washed once with PBS, followed by another centrifugation step under the same conditions. Finally, the cell pellets were digested in 1 mL 10% solution of HNO_3_ in Milli-Q H_2_O overnight, and the Ru content of the solution quantified by ICP–MS.

### Scratch Wound Healing Assay

The efficacy of Ru­(III) NAMI-type complexes on cell migration was assessed using the wound healing assay. T24 cells were seeded in a 6-well plate (800,000 cells/well) and incubated overnight. After aspirating the medium, the wounds were performed in the monolayer cells using a 200 μL sterile micropipette tip. Wells were washed two times with sterile PBS to remove cell debris and then cultured in serum-free media with or without tested compounds. Images of cell migration were taken at 0 and 24 h by an inverted microscope, and the area of migration was measured with ImageJ software. The closure of denuded area (%) was calculated as the ratio of the remaining wound area at 24 h to the original wound area created at 0 h. The data represent the means ± SEM of two independent experiments.

## Supplementary Material







## Notes

Additional scheme, X-ray crystallographic data of complexes, anticancer activities, UV–vis hydrolysis spectra of complexes **Ru2**, **Ru6**, **Ru11** and **Ru12**, cellular uptake studies, docking studies, ROS studies, cell migration of ligands ^1^H NMR spectra for the prepared ligands and complexes, mass spectra and HPLC purity analysis (PDF)
Global Cif with all 12 structures in one (CIF)
SMILES and IC_50_ spreadsheet (CSV)

## Abbreviations

A549: human nonsmall cell lung cancer cells
ALDH: aldehyde dehydrogenase
BOC: *tert*-butyloxycarbonyl
DMF: *N*,*N*-dimethylformamide
DMSO: dimethyl sulfoxide
dmso-*S*: sulfur-bonded dimethyl sulfoxide
EA: elemental analysis
ESI^–^: electron spray ionization in negative ion mode
HaCaT: human keratinocytes
HPLC: high pressure liquid chromatography
^1^H NMR: proton nuclear magnetic resonance
HRMS: high resolution mass spectrometry
IC_50_: half-maximal inhibitory concentration
KP1339: Na­[*trans*-RuCl_4_(Ind)_2_]
LMCT: ligand-to-metal charge transfer
LRMS: low resolution mass spectrometry
MeWo: human BRAF wild-type melanoma cell
NAC: *N*-acetylcysteine
NAMI: Na­[*trans*-RuCl_4_(dmso-*S*) (Im)]
NAMI-A: (ImH)­[*trans*-RuCl_4_(dmso-*S*) (Im)]
PBS: phosphate buffered saline solution
ROS: reactive oxygen species
Ru–P1: (DMSO)_2_H­[*trans*-RuCl_4_(dmso-*S*)_2_]
Ru–P2: Na­[*trans*-RuCl_4_(dmso-*S*)_2_]
S.I.: selectivity index
SAR: structure–activity relationship
SC-XRD: single crystal X-ray diffraction
T24: human bladder cancer cells
