# Supplementary material for: New Ru(III) NAMI/NAMI‑A Analogue Complexes with Selective Anticancer Activity
Source: J Med Chem. 2026 Jul 23;69(15):17881–95. doi: 10.1021/acs.jmedchem.5c03490 (PMC13492274; doi:10.1021/acs.jmedchem.5c03490)
Supplement: Supplementary file 1 [file jm5c03490_si_001.pdf]

Supporting Information

New Ru(III) NAMI/NAMI-A analogue complexes with selective anticancer activity.

Manel Estruch-Blasco,[a] Jose Manuel Calderón-Montaño,[b] Eleuterio Álvarez,[c] Antonio Sánchez-Coronilla,[d] Miguel López-Lázaro,[b] and Manuel Pernia Leal.[a,].*

[a]Departamento de Química Orgánica y Farmacéutica, Facultad de Farmacia, Universidad de Sevilla, c/Profesor García González, 2, 41012, Sevilla, Spain.

[b]Departamento de Farmacología, Facultad de Farmacia, Universidad de Sevilla, c/Profesor García González, 2, 41012, Sevilla, Spain.

[c]Instituto de Investigaciones Químicas (IIQ), CSIC-Universidad de Sevilla, avda. Américo Vespucio 49, 41092, Sevilla, Spain.

[d]Departamento de Química Física, Facultad de Farmacia, Universidad de Sevilla, c/Profesor García González, 2, 41012, Sevilla, Spain.

*Corresponding author. Email: mpernia@us.es

Table of Contents

Ligand synthetic schemes 3
Complex synthesis 4
SC-XRD data of complexes Ru1-Ru10. 6
Anticancer activity evaluation of complexes Ru1, Ru3-Ru5, Ru7-Ru10. 29
SC-XRD data of complexes Ru11 and Ru12. 30
Anticancer activity evaluation of complexes Ru11 and Ru12. 35
UV-Vis hydrolysis spectra of complexes Ru2, Ru6, Ru11 and Ru12. 37
Ru uptake 39
ROS 40
Docking studies 41
Effect of ligands in cell migration. 55
^{1}H NMR characterization of ligands: 57
^{1}H NMR characterization of complexes: 61
LRMS and HRMS of complexes (ESI^{-}). 80
HPLC purity analysis of complexes. 93

Ligand synthetic schemes

Scheme S1. Synthesis of *tert*-butyl (pyridin-3-ylmethyl)carbamate (**L4**)

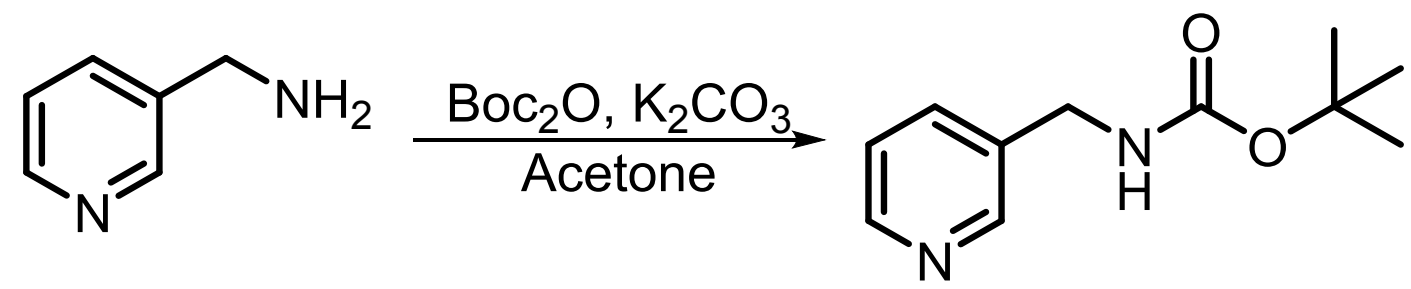

L4

Scheme S2. Synthesis of 3-(isothiocyanatomethyl)pyridine (**L6**)

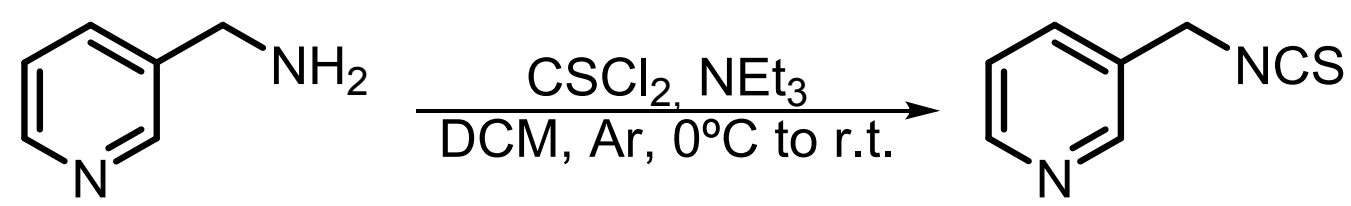

L6

Scheme S3. Synthesis of 4-(azidomethyl)pyridine (**L8**)

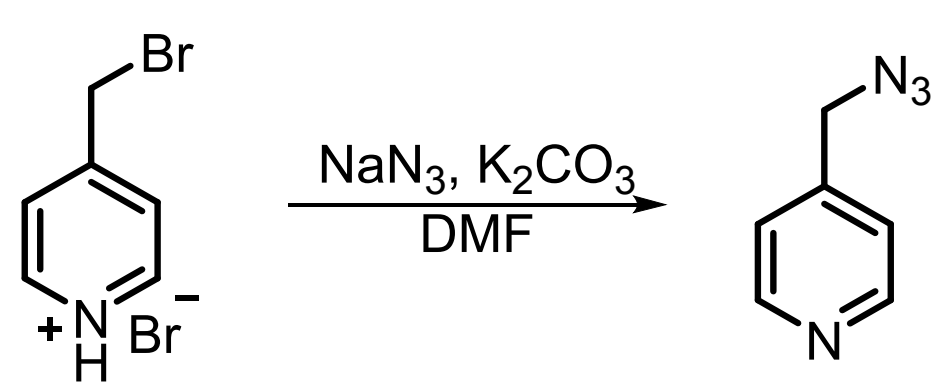

L8

Scheme S4. Synthesis of *tert*-butyl (pyridin-4-ylmethyl)carbamate (**L9**)

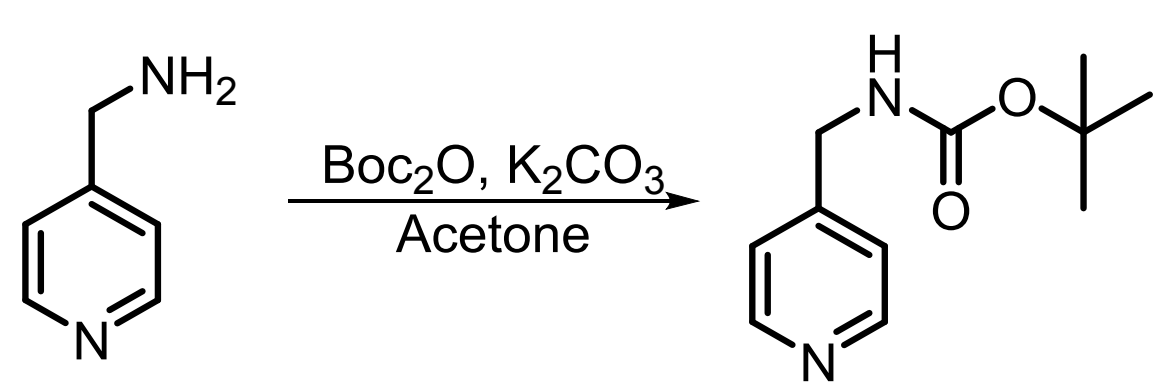

L9

Complex synthesis

Scheme S5. Synthesis of precursors Ru-P1 and Ru-P2. **a)** 1. 80 ˚C, 20 min. 2. 105 ˚C, 15 min. **b)** EtOH/H_2O.

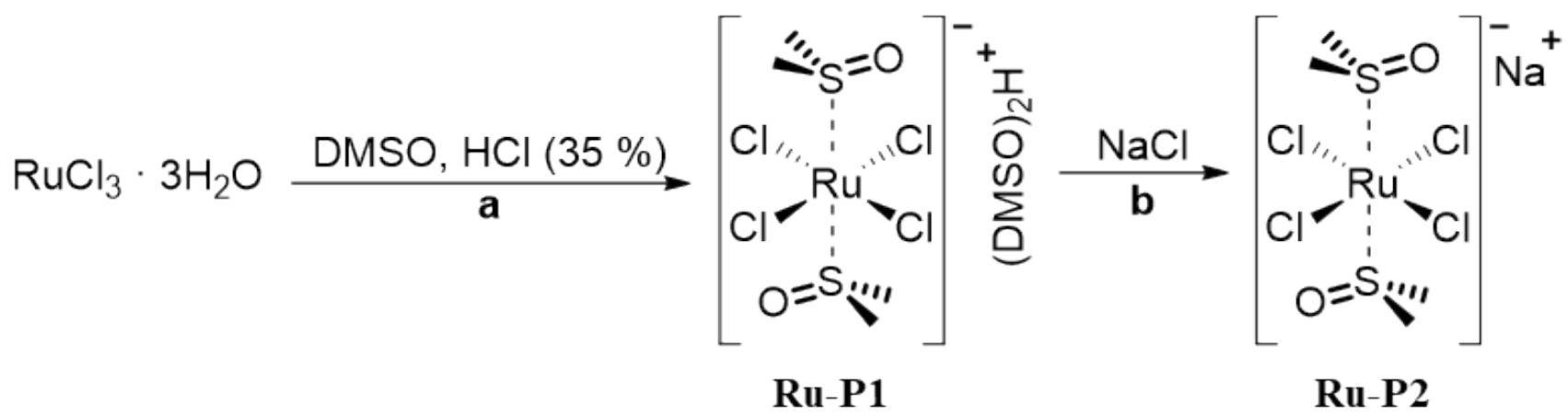

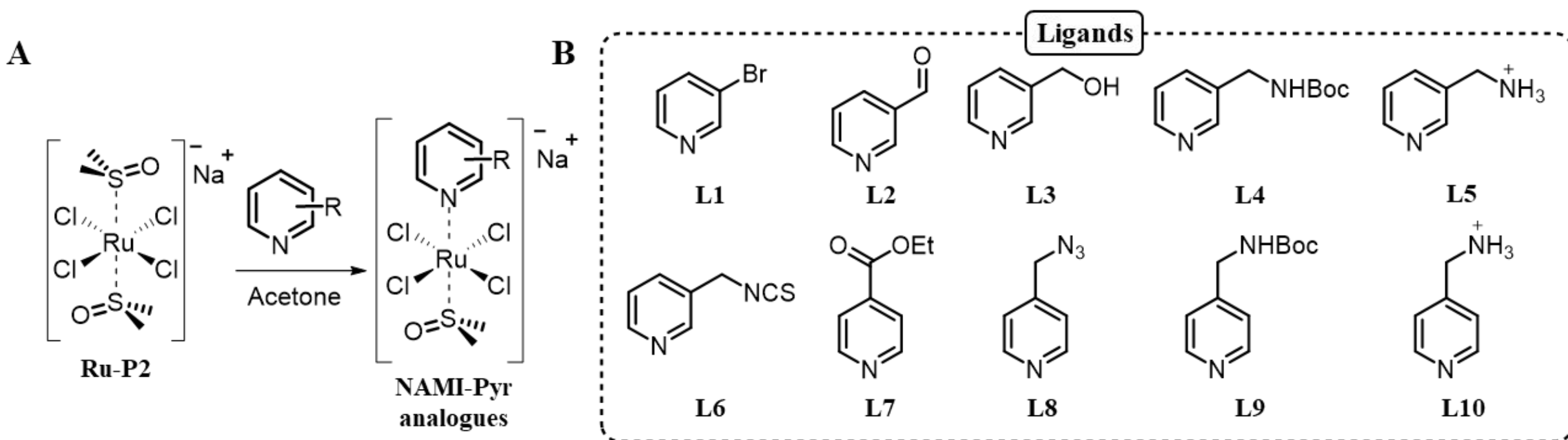

Figure S1. **(A)** Synthetic scheme of the new NAMI analogues **R1-Ru4** and **Ru6-Ru9**. **(B)** Structures of the pyridines used as the heterocyclic ligand. Ligands are named as L followed by the number of the complex in which they were used.

Scheme S6. BOC deprotection of complexes **Ru4** and **Ru9** to obtain **Ru5** and **Ru10**.

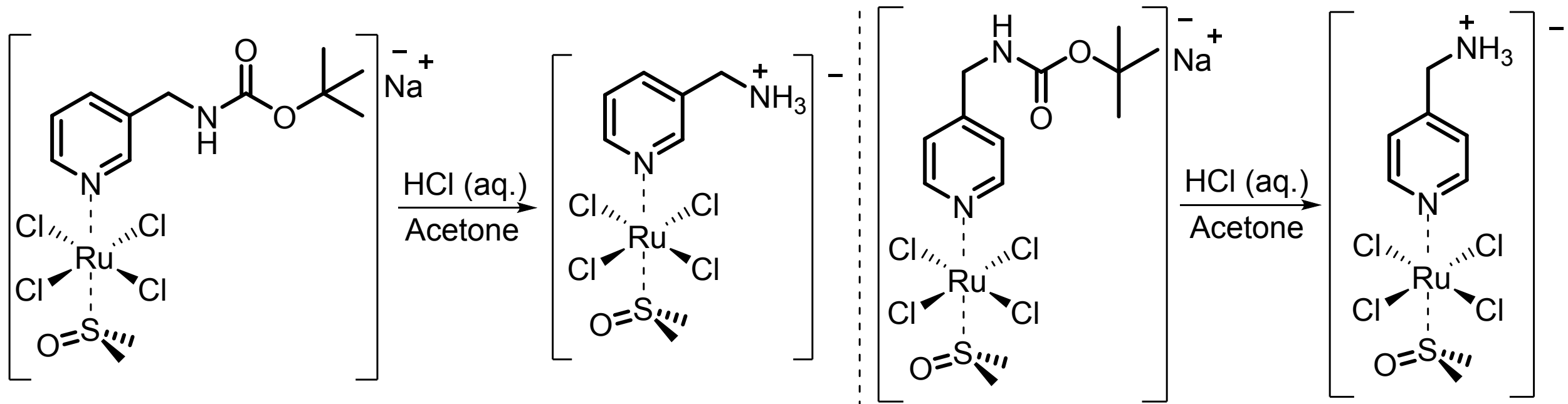

Ru4 **Ru5** **Ru9** **Ru10**

Scheme S7. Synthetic scheme of the new NAMI-A analogues Ru11 and Ru12.

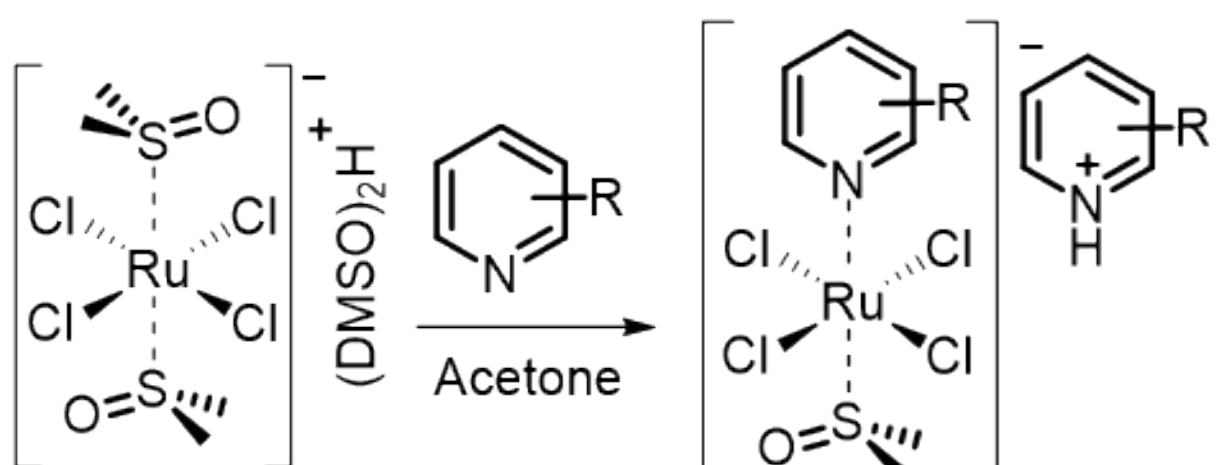

Ru-P1

NAMI-A analogues

SC-XRD data of complexes Ru1-Ru10.

The crystal structure of **Ru1** consists of two independent anionic Ru complexes, each exhibiting the previously described octahedral coordination. These complexes are accompanied by a trihydrated sodium cation and a protonated DMSO molecule. All ions are interconnected through intermolecular hydrogen bonds. In broader detail, one of the two Ru(III) complexes forms chlorine bridges with a sodium atom through two of its chlorine ligands (d[Ru-Cl] = 2.357 and 2.360 Å), resulting in a somewhat distorted octahedral geometry around the sodium atom. The sodium atom also coordinates with the oxygen atom of the DMSO in the same complex and three water molecules. Two of these water molecules form hydrogen bonds with a chlorine atom and the DMSO oxygen of the other Ru complex. To balance the charges of the Ru complex anions, a protonated DMSO molecule is present, forming hydrogen bonds with the remaining coordination water molecule attached to the sodium atom (Figure S2).

The **Ru2**, **Ru4**, and **Ru9** complexes form 2D and 1D polymeric structures involving links formed by pairs of octahedral Ru anions coordinated in a head-to-tail manner to pairs of sodium cations. These sodium cations exhibit two oxygen bridges from the DMSO ligands (tail) belonging to two different Ru anions. Each of these Ru anions also coordinates through two of its chlorine ligands to a single sodium atom in the pair. The octahedral coordination of each Na atom is completed by binding to the carbonyl group (head) present on the pyridine ring of another Ru complex and also to an acetone molecule (Figures S3, S4 and S5).

The crystal structures of **Ru3**, **Ru7**, and **Ru8** exhibit tetranuclear complexes formed by a pair of octahedral Ru anions coordinated to two sodium atoms through two oxygen bridges from each of their DMSO ligands. Furthermore, the distorted octahedral coordination of each Na atom in **Ru3** is completed by two chlorine ligands from each of the Ru anions, by the oxygen of an acetone molecule, and by the hydroxyl group of another free 3-pyridinemethanol molecule. In contrast, the distorted octahedral coordination of each Na atom in **Ru8** is completed by a single chlorine ligand from each of the Ru anions, a water molecule, and an oxygen atom from another free DMSO molecule. Finally, the distorted tetrahedral coordination of each Na atom in **Ru7** is completed by a single chlorine ligand from each of the Ru anions and two water molecules coordinating each sodium atom (Figures S6, S7 and S8).

The crystal structure of **Ru6** also exhibits tetranuclear complexes formed by a pair of octahedral Ru anions, similar to the previous structures. However, it differs in that each Ru anion is coordinated to a single sodium atom through the oxygen atom of its DMSO ligand, in addition to a single chlorine ligand. The most notable difference is that three acetone molecules form oxygen bridges with both sodium atoms. Additionally, to complete their distorted octahedral coordination, each sodium atom is terminally coordinated with the oxygen of another acetone molecule (Figure S9).

The crystal structures of the zwitterionic complexes **Ru5** and **Ru10** appear simpler, each comprising a single molecule of the octahedral complex of Ru(III) featuring an ammonium group in its pyridine ring coordinated to Ru, thereby compensating for the four chloride atoms of

the complex. Additionally, both zwitterions in their respective structures are accompanied by two crystallization *N,N*-dimethylformamide (DMF) molecules that form intra- and intermolecular hydrogen bonds with both the ammonium groups and the chloride atoms coordinated to the Ru atom (Figures S10, S11).

The **Ru1** crystal exhibits positional disorder in the protonated DMSO cation at two positions, with refined occupancy coefficients of 76:24. In contrast, the **Ru8** crystal displays substitutional disorder in the acetone/DMSO molecules coordinated to a sodium atom, with refined occupancy coefficients of 77.9:22.1. Consequently, it was necessary to apply geometric restraints (SADI, SIMU, etc.) during the structure refinement to ensure a sensible geometry. The crystal of the **Ru7** complex appears to be twinned, so it was resolved and refined as a two-component twin with a refined domain ratio of 72:28. A search for solvent-accessible voids in the **Ru1**, **Ru5**, and **Ru9** structures using SQUEEZE[1] revealed potential solvent volumes. For **Ru1**, a volume of 1027 Å^3 (361 electrons) was identified. For Ru5, a volume of 218 Å^3 (45 electrons) was found. For **Ru9**, two volumes of 45 Å^3 (2 electrons) and 42 Å^3 (1 electron) were detected. The solvent content in these voids could not be identified or refined, even under the most stringent restrictions. The corresponding CIF data represent SQUEEZE treated structures with the solvent molecules handling as a diffuse contribution to the overall scattering, without specific atom position and excluded from the structural model. The SQUEEZE results were appended to the CIF.

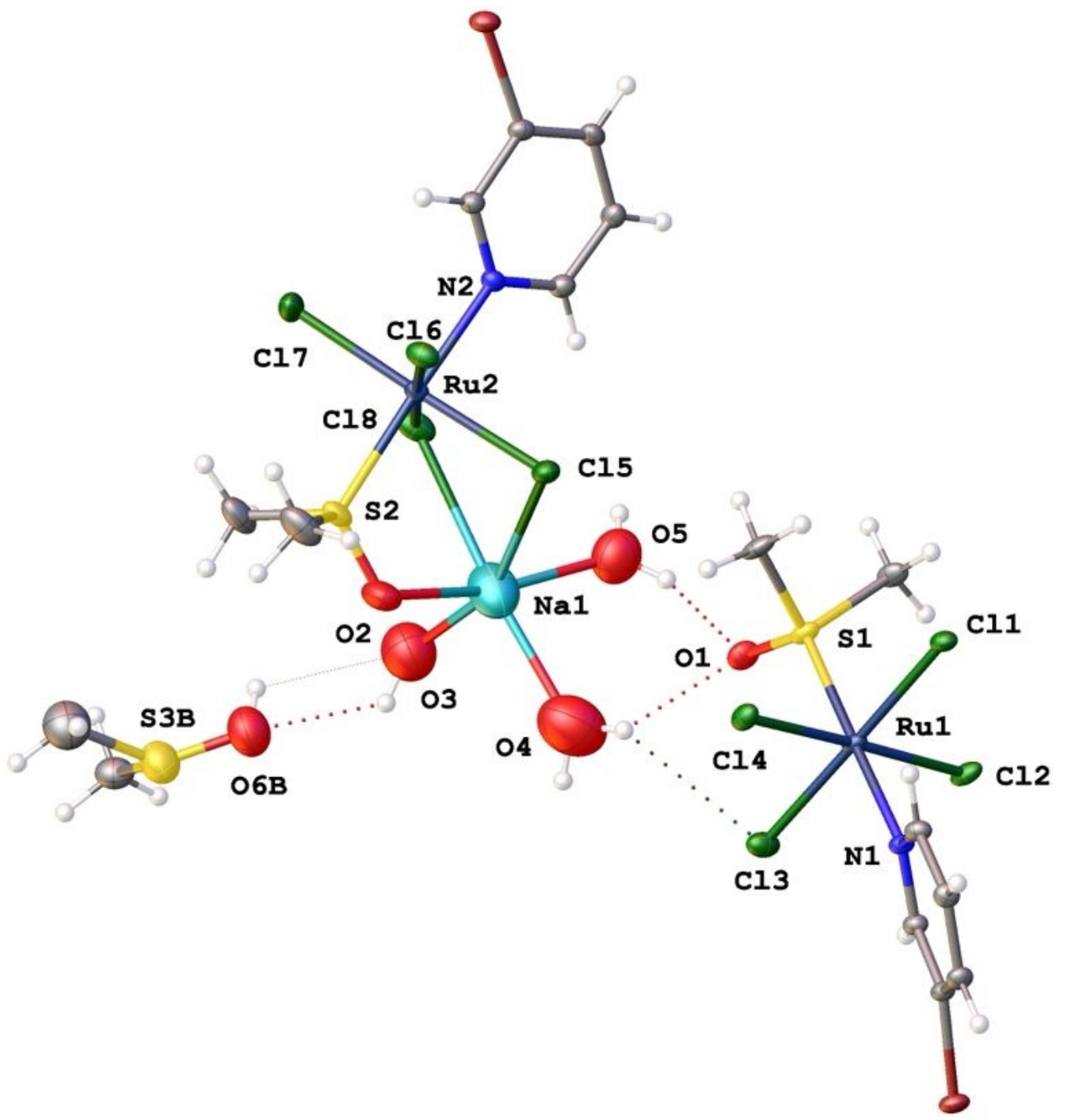

Figure S2. ORTEP view of **Ru1** with thermal ellipsoids depicted at the 30% probability level.

Table S1. Crystal data and structure refinement for **Ru1.**

Empirical formula	$C_{16}H_{33}Br_2Cl_8N_2NaO_6Ru_2S_3$
Formula weight	1114.17
Temperature/K	193.00
Crystal system	Monoclinic
Space group	$P2_1/c$
a/Å	16.5799(5)
b/Å	8.3436(2)
c/Å	33.9387(8)
α/°	90
β/°	98.3290(10)
γ/°	90
Volume/Å^3	4645.4(2)
Z	4

ρ_{calc}g/cm^3	1.593
μ/mm^{-1}	3.001
F(000)	2176.0
Crystal size/mm^3	0.4 × 0.3 × 0.1
Radiation	MoKα (λ = 0.71073)
2Θ range for data collection/°	4.852 to 50.5
Index ranges	-19 ≤ h ≤ 19, -10 ≤ k ≤ 9, -40 ≤ l ≤ 37
Reflections collected	53999
Independent reflections	8363 [R_{int} = 0.0268, R_{sigma} = 0.0190]
Data/restraints/parameters	8363/182/401
Goodness-of-fit on F^2	1.042
Final R indexes [I>=2σ (I)]	R_1 = 0.0420, wR_2 = 0.1143
Final R indexes [all data]	R_1 = 0.0458, wR_2 = 0.1167
Largest diff. peak/hole / e Å^{-3}	1.14/-1.27

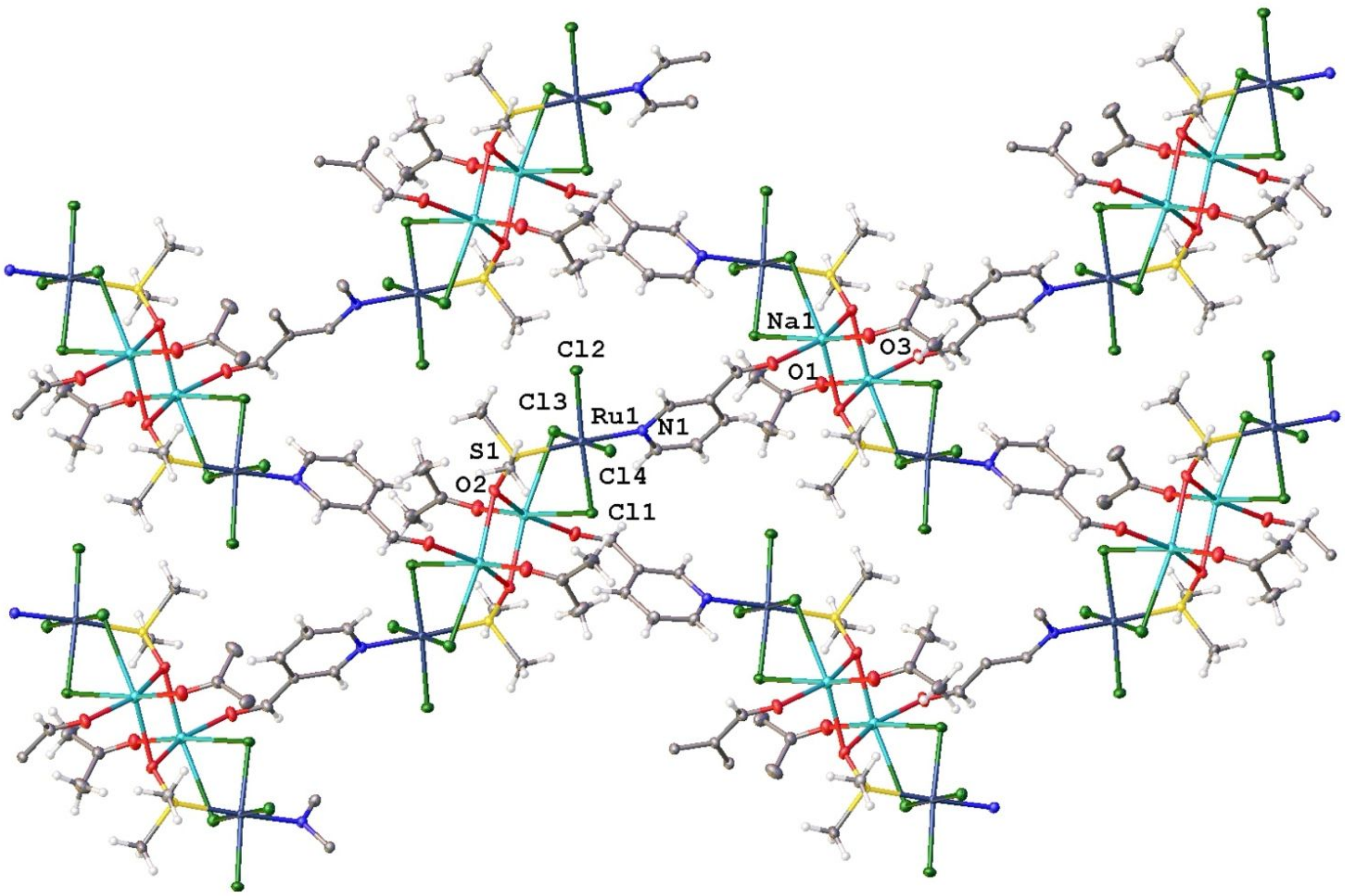

Figure S3. ORTEP view of **Ru2** with thermal ellipsoids depicted at the 30% probability level.

Table S2. Crystal data and structure refinement for **Ru2.**

Empirical formula	$C_{11}H_{17}Cl_4NNaO_3RuS$
Formula weight	509.17
Temperature/K	193.00
Crystal system	Monoclinic
Space group	$P2_1/c$
a/Å	11.254(2)
b/Å	11.0160(19)
c/Å	16.037(3)
α/°	90
β/°	110.393(6)
γ/°	90
Volume/Å^3	1863.5(6)

Z	4
ρ_{calc}g/cm^3	1.815
μ/mm^{-1}	1.557
F(000)	1012.0
Crystal size/mm^3	0.35 × 0.2 × 0.1
Radiation	MoKα (λ = 0.71073)
2Θ range for data collection/°	4.584 to 55.494
Index ranges	-12 ≤ h ≤ 14, -14 ≤ k ≤ 14, -21 ≤ l ≤ 21
Reflections collected	20067
Independent reflections	4325 [R_{int} = 0.0291, R_{sigma} = 0.0271]
Data/restraints/parameters	4325/0/203
Goodness-of-fit on F^2	0.988
Final R indexes [I>=2σ (I)]	R_1 = 0.0294, wR_2 = 0.0935
Final R indexes [all data]	R_1 = 0.0298, wR_2 = 0.0938
Largest diff. peak/hole / e Å^{-3}	0.92/-0.81

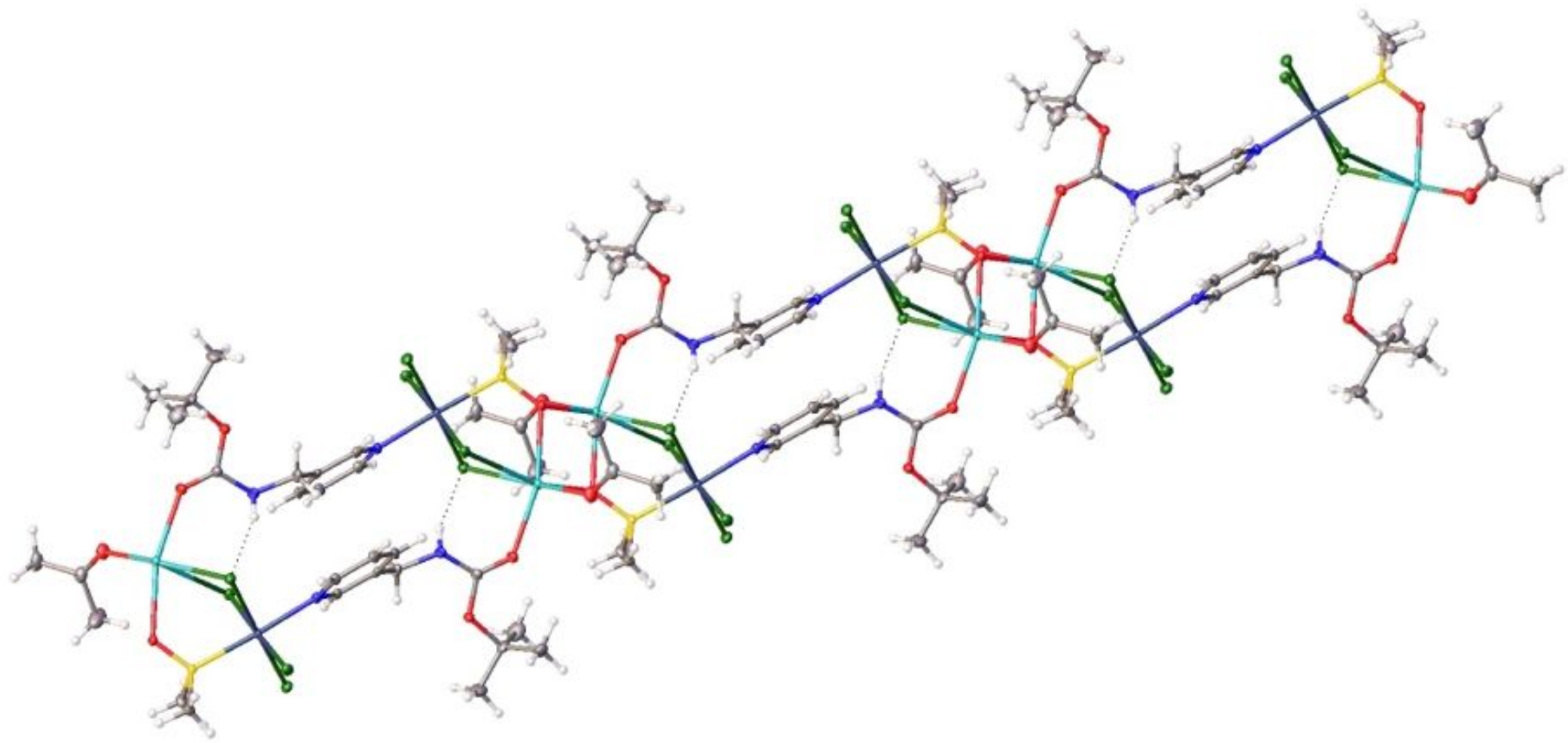

Figure S4. ORTEP view of **Ru4** with thermal ellipsoids depicted at the 30% probability level.

Table S3. Crystal data and structure refinement for **Ru4.**

Empirical formula	$C_{16}H_{28}Cl_4N_2NaO_4RuS$
Formula weight	610.32
Temperature/K	193.00
Crystal system	Triclinic
Space group	P-1
a/Å	9.5990(3)
b/Å	11.6112(3)
c/Å	12.4809(4)
α/°	101.9030(10)
β/°	90.7430(10)
γ/°	113.1780(10)
Volume/Å^3	1244.42(7)
Z	2
ρ_{calc}g/cm^3	1.629
μ/mm^{-1}	1.185
F(000)	618.0
Crystal size/mm^3	0.02 × 0.012 × 0.008

Radiation	MoKα (λ = 0.71073)
2Θ range for data collection/°	3.92 to 50.5
Index ranges	$-11 \leq h \leq 11$, $-13 \leq k \leq 13$, $-14 \leq l \leq 14$
Reflections collected	39556
Independent reflections	4514 [R_{int} = 0.0623, R_{sigma} = 0.0294]
Data/restraints/parameters	4514/0/269
Goodness-of-fit on F^2	1.077
Final R indexes [I>=2σ (I)]	R_1 = 0.0264, wR_2 = 0.0563
Final R indexes [all data]	R_1 = 0.0311, wR_2 = 0.0580
Largest diff. peak/hole / e $Å^{-3}$	0.65/-0.53

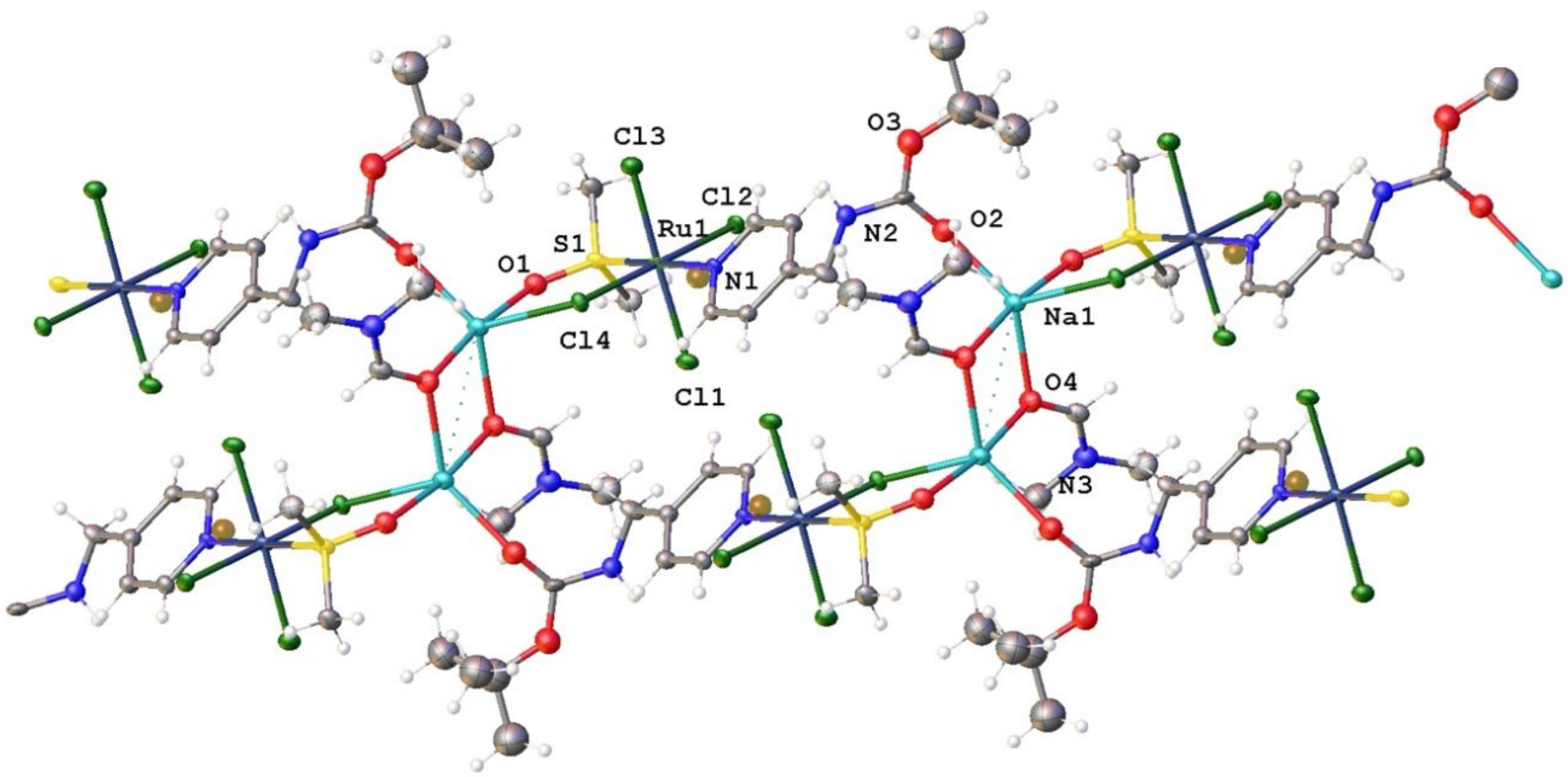

Figure S5. ORTEP view of **Ru9** with thermal ellipsoids depicted at the 30% probability level.

Table S4. Crystal data and structure refinement for **Ru9**.

Empirical formula	$C_{16}H_{29}Cl_4N_3NaO_4RuS$
Formula weight	625.34
Temperature/K	193.00
Crystal system	Triclinic
Space group	P-1
a/Å	7.6233(12)
b/Å	12.5505(19)
c/Å	15.476(2)
α/°	68.257(5)
β/°	85.450(6)
γ/°	76.421(6)
Volume/Å^3	1336.9(4)
Z	2
ρ_{calc}g/cm^3	1.553
μ/mm^{-1}	1.106
F(000)	634.0
Crystal size/mm^3	0.1 × 0.08 × 0.05

Radiation	MoKα (λ = 0.71073)
2Θ range for data collection/°	5.498 to 50.496
Index ranges	$-9 \leq h \leq 7$, $-15 \leq k \leq 15$, $-18 \leq l \leq 18$
Reflections collected	11624
Independent reflections	4094 [R_{int} = 0.0755, R_{sigma} = 0.1034]
Data/restraints/parameters	4094/227/278
Goodness-of-fit on F^2	1.096
Final R indexes [I>=2σ (I)]	R_1 = 0.1097, wR_2 = 0.2960
Final R indexes [all data]	R_1 = 0.1631, wR_2 = 0.3227
Largest diff. peak/hole / e Å^{-3}	2.55/-1.53

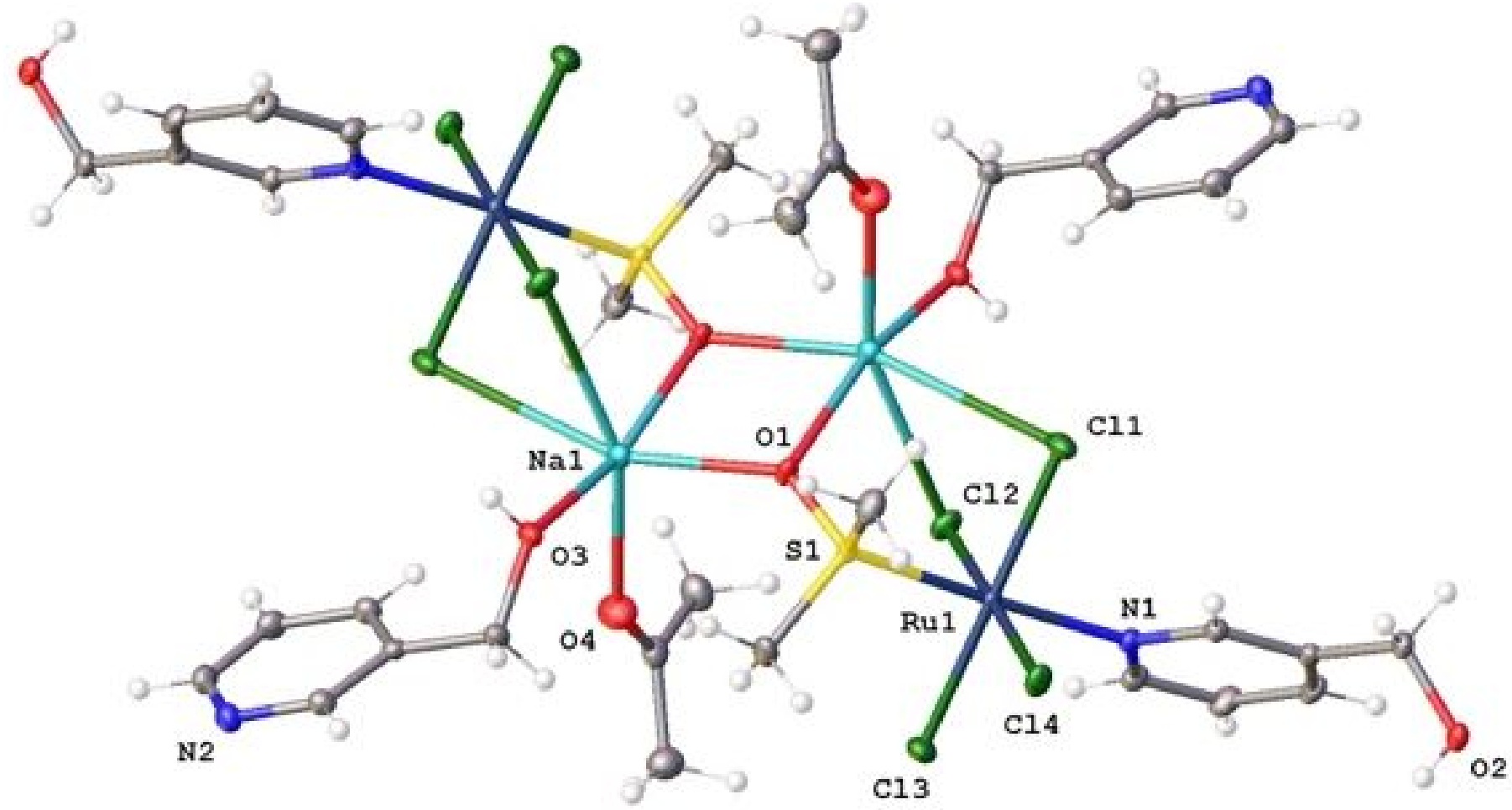

Figure S6. ORTEP view of **Ru3** with thermal ellipsoids depicted at the 30% probability level.

Table S5. Crystal data and structure refinement for **Ru3.**

Empirical formula	$C_{34}H_{52}Cl_8N_4Na_2O_8Ru_2S_2$
Formula weight	1240.63
Temperature/K	193.00
Crystal system	Triclinic
Space group	P-1
a/Å	8.2033(6)
b/Å	11.1802(7)
c/Å	14.0550(10)
α/°	105.487(2)
β/°	99.845(3)
γ/°	91.155(3)
Volume/Å^3	1221.01(15)
Z	1
ρ_{calc}g/cm^3	1.687
μ/mm^{-1}	1.209
F(000)	626.0
Crystal size/mm^3	0.25 × 0.15 × 0.05
Radiation	MoKα (λ = 0.71073)

2Θ range for data collection/°	5.052 to 50.492
Index ranges	-9 ≤ h ≤ 9, -13 ≤ k ≤ 13, -16 ≤ l ≤ 16
Reflections collected	40670
Independent reflections	4420 [R_{int} = 0.0303, R_{sigma} = 0.0185]
Data/restraints/parameters	4420/0/276
Goodness-of-fit on F^2	1.066
Final R indexes [I>=2σ (I)]	R_1 = 0.0184, wR_2 = 0.0444
Final R indexes [all data]	R_1 = 0.0202, wR_2 = 0.0451
Largest diff. peak/hole / e Å^{-3}	0.68/-0.70

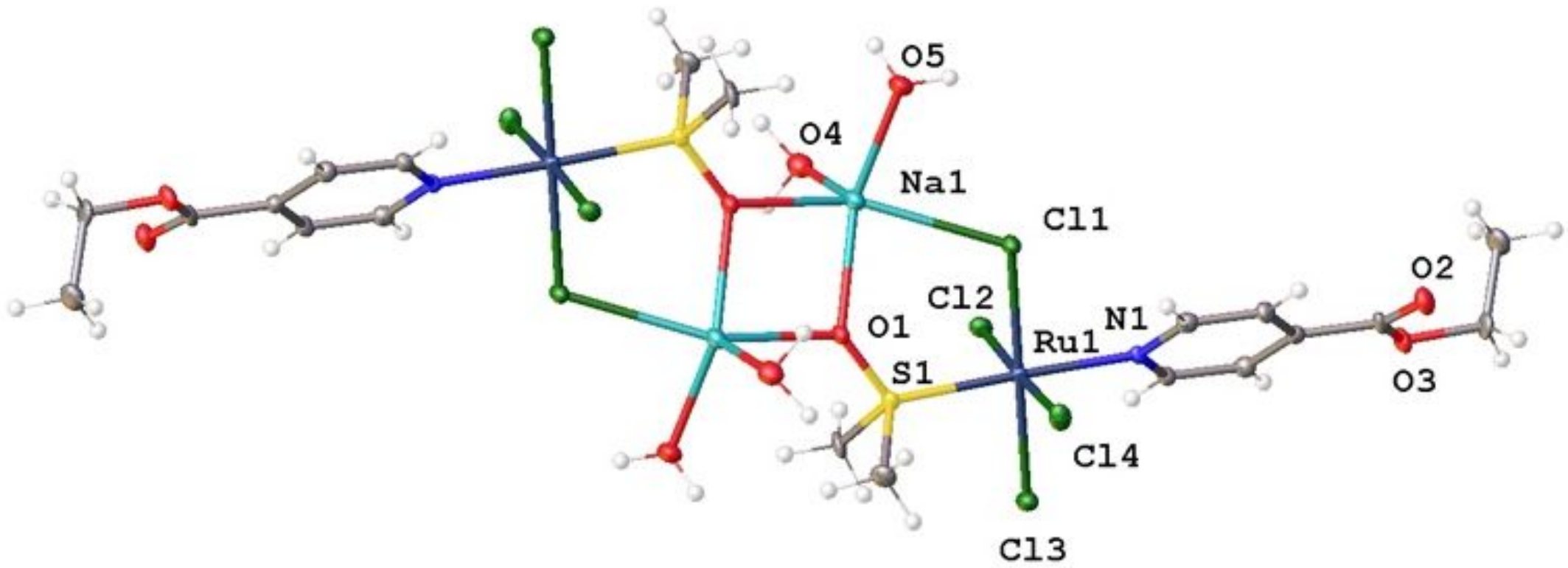

Figure S7. ORTEP view of **Ru7** with thermal ellipsoids depicted at the 30% probability level.

Table S6. Crystal data and structure refinement for **Ru7.**

Empirical formula	$C_{20}H_{38}Cl_8N_2Na_2O_{10}Ru_2S_2$
Formula weight	1062.36
Temperature/K	193.00
Crystal system	Triclinic
Space group	P-1
a/Å	7.8612(6)
b/Å	10.2250(8)
c/Å	12.1815(9)
α/°	104.677(3)
β/°	90.052(3)
γ/°	96.685(3)
Volume/Å^3	940.30(12)
Z	1
ρ_{calc}g/cm^3	1.876
μ/mm^{-1}	1.555
F(000)	530.0
Crystal size/mm^3	0.2 × 0.1 × 0.05
Radiation	MoKα (λ = 0.71073)
2Θ range for data collection/°	4.148 to 50.498

Index ranges	$-9 \le h \le 9$, $-12 \le k \le 11$, $0 \le l \le 14$
Reflections collected	3415
Independent reflections	3415 [R_{int} = ?, R_{sigma} = 0.0487]
Data/restraints/parameters	3415/51/214
Goodness-of-fit on F^2	1.047
Final R indexes [I>=2σ (I)]	R_1 = 0.0441, wR_2 = 0.0989
Final R indexes [all data]	R_1 = 0.0649, wR_2 = 0.1062
Largest diff. peak/hole / e $Å^{-3}$	0.74/-0.84

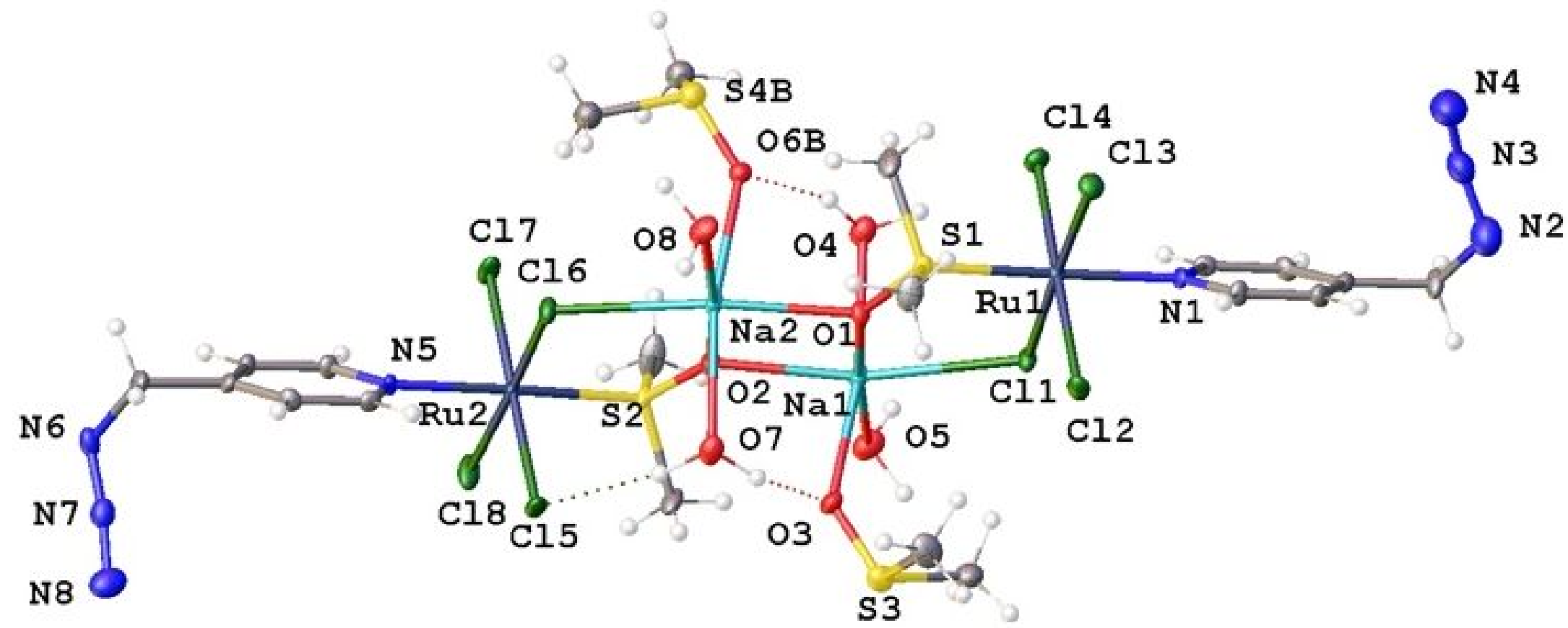

Figure S8. ORTEP view of **Ru8** with thermal ellipsoids depicted at the 30% probability level.

Table S7. Crystal data and structure refinement for **Ru8.**

Empirical formula	$C_{20.77}H_{44}Cl_8N_8Na_2O_8Ru_2S_{3.23}$
Formula weight	1169.05
Temperature/K	193.00
Crystal system	Monoclinic
Space group	$P2_1/n$
a/Å	19.911(3)
b/Å	7.9923(13)
c/Å	28.056(4)
α/°	90
β/°	101.027(6)
γ/°	90
Volume/Å^3	4382.3(12)
Z	4
ρ_{calc}g/cm^3	1.772
μ/mm^{-1}	1.400
F(000)	2345.0
Crystal size/mm^3	0.12 × 0.08 × 0.05
Radiation	MoKα (λ = 0.71073)
2Θ range for data collection/°	4.168 to 50.498

Index ranges	$-23 \le h \le 22$, $-9 \le k \le 9$, $-33 \le l \le 33$
Reflections collected	76061
Independent reflections	7871 [R_{int} = 0.0732, R_{sigma} = 0.0382]
Data/restraints/parameters	7871/257/516
Goodness-of-fit on F^2	1.134
Final R indexes [I>=2σ (I)]	R_1 = 0.0943, wR_2 = 0.2245
Final R indexes [all data]	R_1 = 0.1083, wR_2 = 0.2314
Largest diff. peak/hole / e $Å^{-3}$	7.39/-1.44

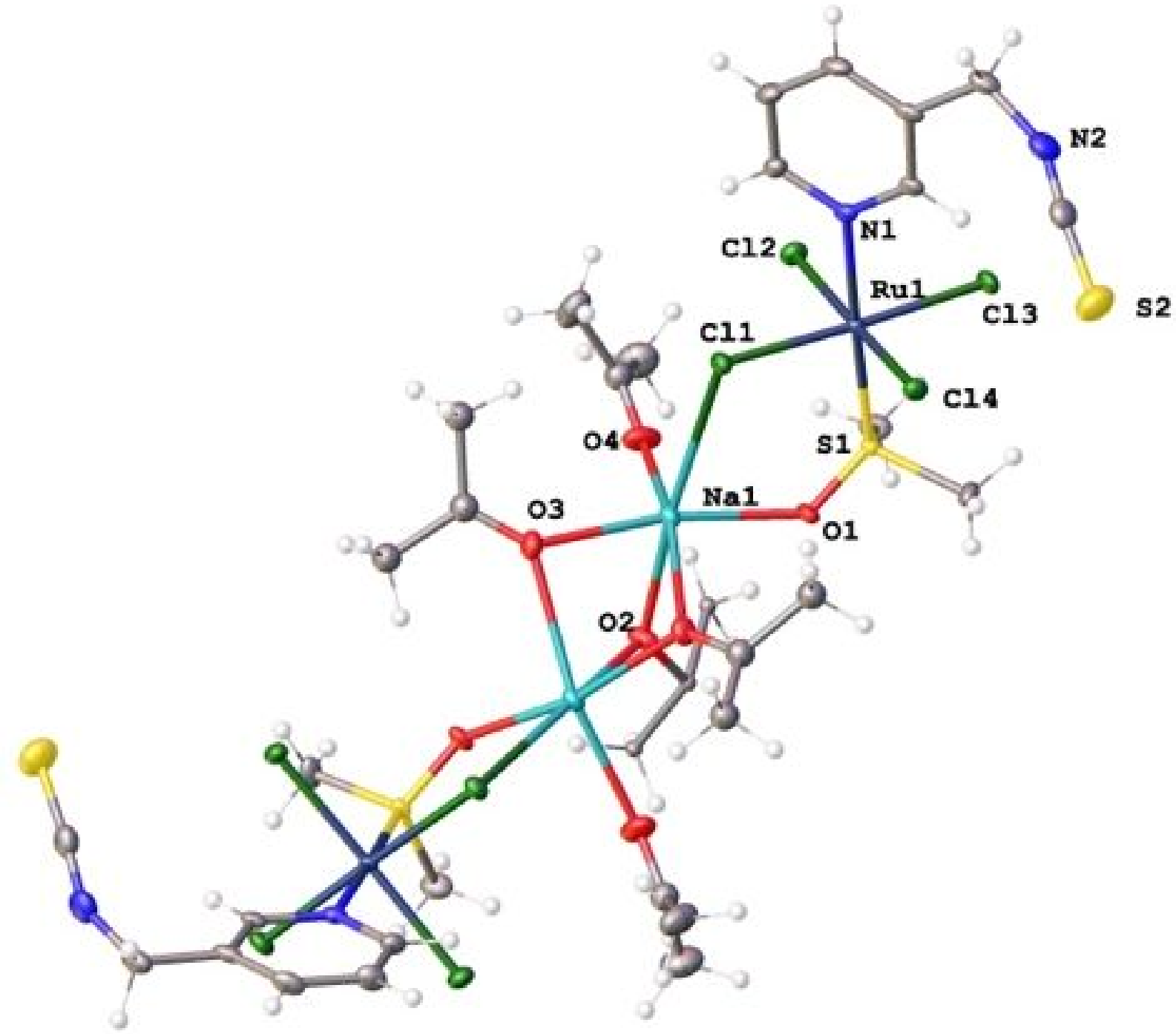

Figure S9. ORTEP view of **Ru6** with thermal ellipsoids depicted at the 30% probability level.

Table S8. Crystal data and structure refinement for **Ru6.**

Empirical formula	$C_{33}H_{54}Cl_8N_4Na_2O_7Ru_2S_4$
Formula weight	1278.76
Temperature/K	193.00
Crystal system	Monoclinic
Space group	C2/c
a/Å	34.728(3)
b/Å	7.7522(7)
c/Å	24.430(2)
α/°	90
β/°	127.018(3)
γ/°	90
Volume/Å^3	5251.2(8)
Z	4
ρ_{calc}g/cm^3	1.617

μ/mm^{-1}	1.202
F(000)	2584.0
Crystal size/mm^3	0.3 × 0.1 × 0.08
Radiation	MoKα (λ = 0.71073)
2Θ range for data collection/°	5.456 to 50.492
Index ranges	-41 ≤ h ≤ 41, -9 ≤ k ≤ 9, -29 ≤ l ≤ 29
Reflections collected	71146
Independent reflections	4741 [R_{int} = 0.0372, R_{sigma} = 0.0158]
Data/restraints/parameters	4741/189/327
Goodness-of-fit on F^2	1.124
Final R indexes [I>=2σ (I)]	R_1 = 0.0440, wR_2 = 0.1165
Final R indexes [all data]	R_1 = 0.0451, wR_2 = 0.1170
Largest diff. peak/hole / e Å^{-3}	2.97/-1.14

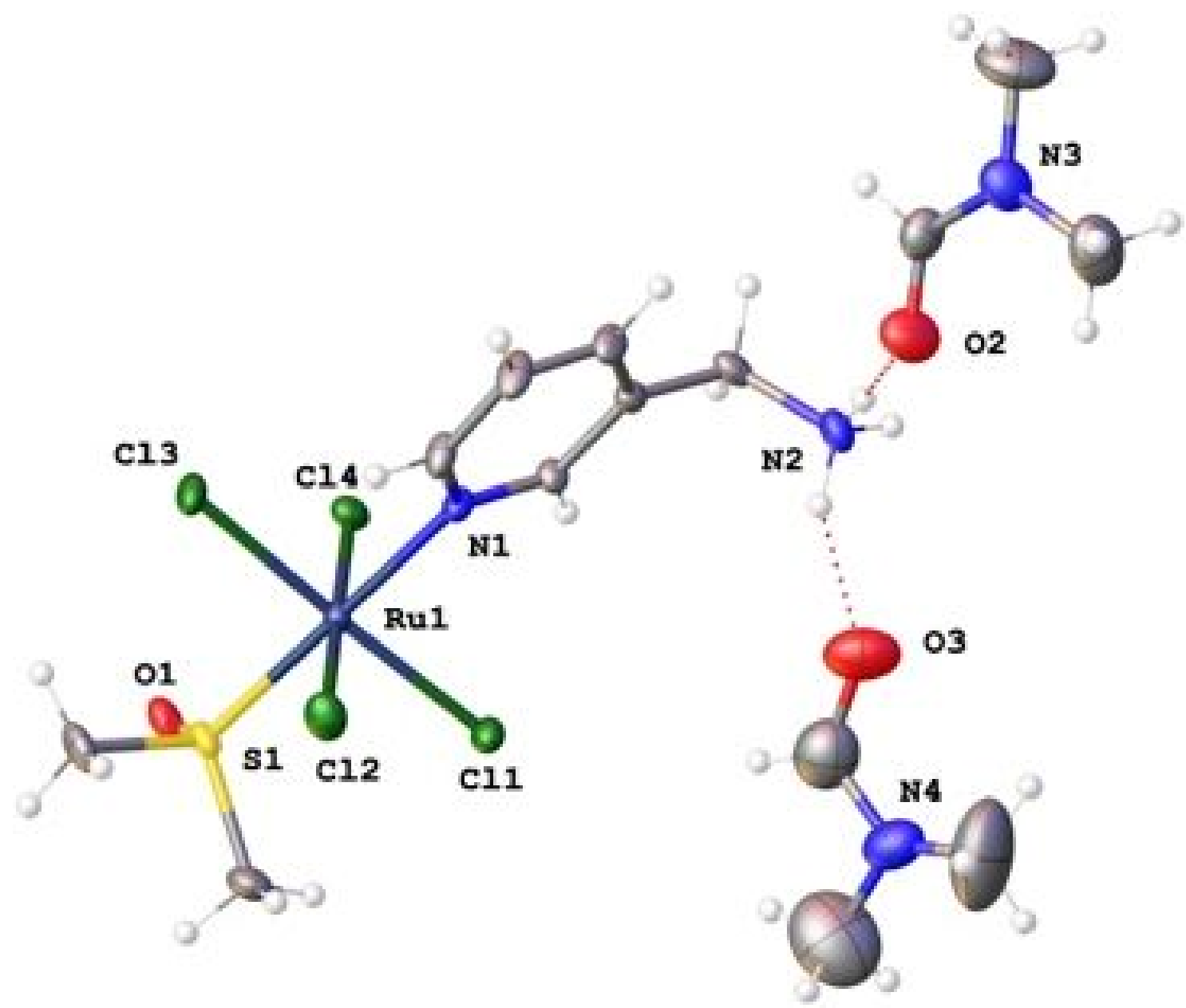

Figure S10. ORTEP view of zwitterionic **Ru5** with thermal ellipsoids depicted at the 30% probability level.

Table S9. Crystal data and structure refinement for **Ru5.**

Empirical formula	$C_{14}H_{26}Cl_4N_4O_3RuS$
Formula weight	573.32
Temperature/K	193.00
Crystal system	Triclinic
Space group	P-1
a/Å	8.5166(5)
b/Å	11.8522(6)
c/Å	13.6489(7)
α/°	80.909(2)
β/°	77.513(2)
γ/°	89.246(2)
Volume/Å^3	1327.92(12)
Z	2

ρ_{calc}g/cm^3	1.434
μ/mm^{-1}	1.090
F(000)	580.0
Crystal size/mm^3	0.3 × 0.1 × 0.05
Radiation	MoKα (λ = 0.71073)
2Θ range for data collection/°	4.276 to 50.494
Index ranges	-10 ≤ h ≤ 10, -14 ≤ k ≤ 14, -16 ≤ l ≤ 16
Reflections collected	25839
Independent reflections	4782 [R_{int} = 0.0344, R_{sigma} = 0.0257]
Data/restraints/parameters	4782/48/250
Goodness-of-fit on F^2	1.029
Final R indexes [I>=2σ (I)]	R_1 = 0.0413, wR_2 = 0.1118
Final R indexes [all data]	R_1 = 0.0476, wR_2 = 0.1155
Largest diff. peak/hole / e Å^{-3}	1.46/-0.48

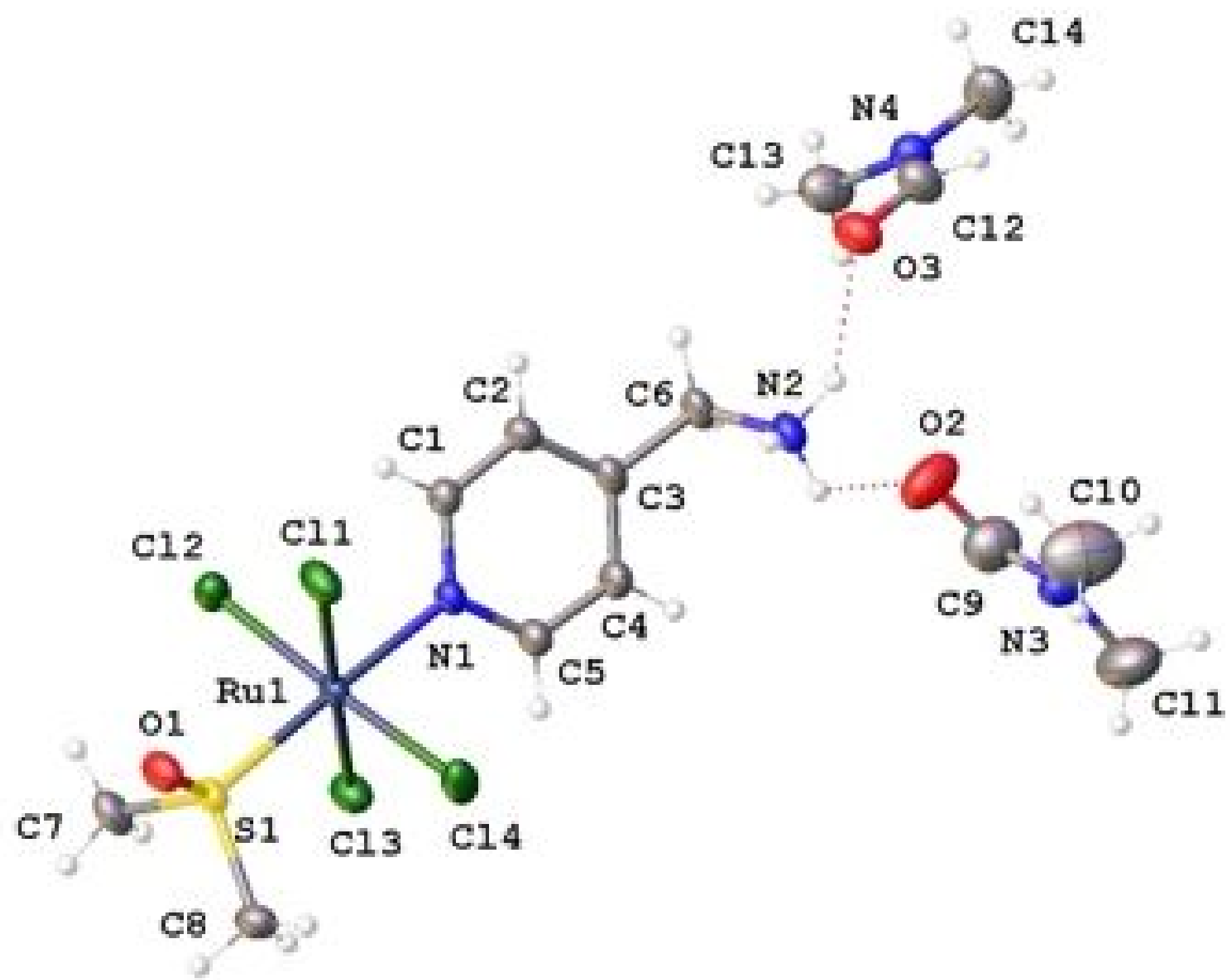

Figure S11. ORTEP view of zwitterionic **Ru10** with thermal ellipsoids depicted at the 30%

probability level.

Table S10. Crystal data and structure refinement for **Ru10.**

Empirical formula	$C_{14}H_{29}Cl_4N_4O_3RuS$
Formula weight	576.34
Temperature/K	193.00
Crystal system	Monoclinic
Space group	$P2_1/n$
a/Å	10.3314(6)
b/Å	21.8637(16)
c/Å	10.5336(8)
α/°	90
β/°	95.870(3)
γ/°	90

Volume/Å^3	2366.9(3)
Z	4
ρ_{calc}g/cm^3	1.617
μ/mm^{-1}	1.223
F(000)	1172.0
Crystal size/mm^3	0.35 × 0.25 × 0.08
Radiation	MoKα (λ = 0.71073)
2Θ range for data collection/°	4.31 to 61.078
Index ranges	-14 ≤ h ≤ 14, -30 ≤ k ≤ 31, -15 ≤ l ≤ 13
Reflections collected	52178
Independent reflections	7214 [R_{int} = 0.0411, R_{sigma} = 0.0246]
Data/restraints/parameters	7214/0/251
Goodness-of-fit on F^2	1.055
Final R indexes [I>=2σ (I)]	R_1 = 0.0364, wR_2 = 0.0844
Final R indexes [all data]	R_1 = 0.0488, wR_2 = 0.0931
Largest diff. peak/hole / e Å^{-3}	1.55/-0.60

Anticancer activity evaluation of complexes Ru1, Ru3-Ru5, Ru7-Ru10.

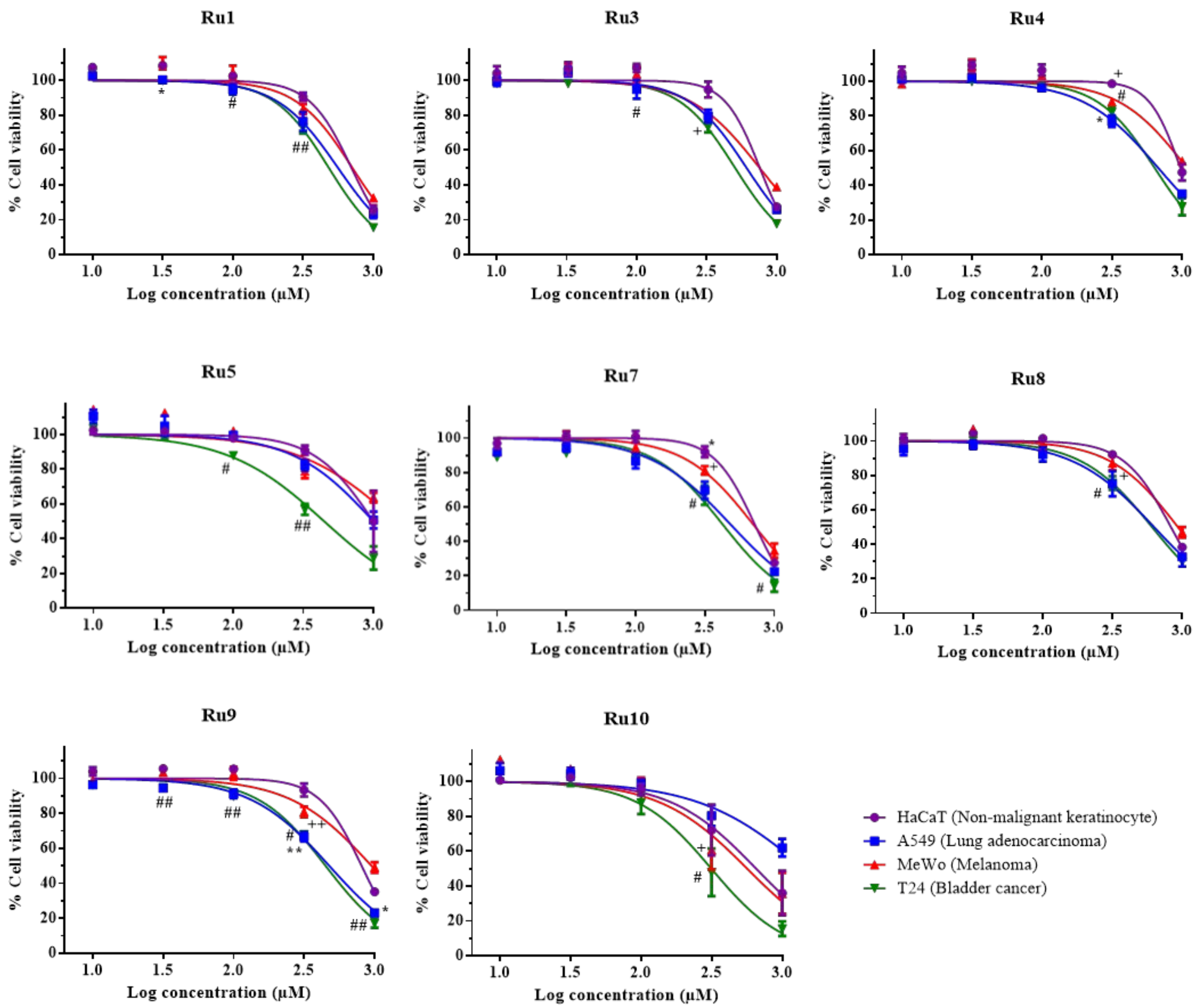

Figure S12. Evaluation of the selective anticancer activity of Ru(III) complexes on human cancer cells (A549 in blue, MeWo in red and T24 in green) and human non-malignant cells (HaCaT in purple). Cells were exposed to several concentrations of compounds for 72 hours. After treatment, cell viability was determined with the resazurin assay. Data represent mean ± SEM from at least three independent experiments. Statistical analysis was calculated using the paired

t-test; * indicates $p < 0.05$, ** indicates $p < 0.01$, ** indicates $p < 0.001$ (similar representations for marks + or #).

SC-XRD data of complexes Ru11 and Ru12.

The crystal structure of **Ru11** consists of an octahedral Ru complex and an unusual cation, specifically a strongly proton-bound DMF dimer: $[(DMF)_2(H+)]$. Single-Crystal X-ray Diffraction reveals a short distance of 2.432 Å between the two oxygen atoms of the DMF molecules. The differences in the donor-acceptor behavior of the two DMF molecules and the fact that the proton is not located midway between them are due to hydrogen bonding interactions between the formyl hydrogens and Cl1 of the Ru complex (Figure 5 and Figure S13).

Ru12 crystallizes as a single octahedral complex anion of Ru, accompanied by a pyridinium 3-isothiocyanate cation. In this structure, the proton of the pyridinium cation forms two hydrogen bonds: one with the oxygen atom of DMSO and another with a chlorine atom, both associated with the Ru complex (Figure 5 and Figure S14).

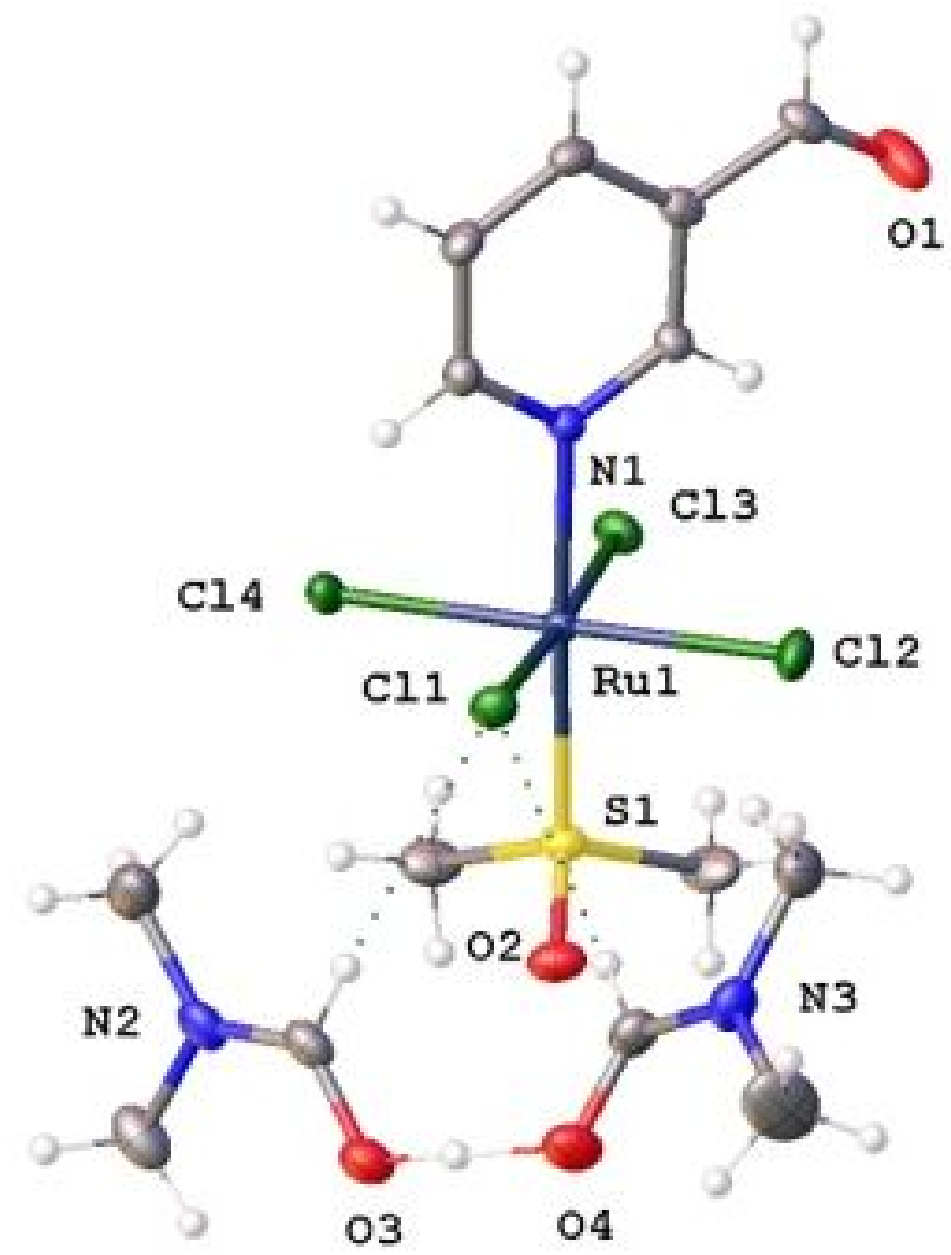

Figure S13. ORTEP view of **Ru11** with thermal ellipsoids depicted at the 30% probability level.

Table S11. Crystal data and structure refinement for **Ru11.**

Empirical formula	$C_{14}H_{26}Cl_4N_3O_4RuS$
Formula weight	575.31
Temperature/K	193.00
Crystal system	Monoclinic
Space group	$P2_1/c$
a/Å	7.7024(10)
b/Å	19.379(2)
c/Å	15.669(2)
α/°	90
β/°	97.462(5)
γ/°	90
Volume/Å^3	2319.0(5)
Z	4
ρ_{calc}g/cm^3	1.648
μ/mm^{-1}	1.251

F(000)	1164.0
Crystal size/mm³	0.15 × 0.1 × 0.05
Radiation	MoKα (λ = 0.71073)
2Θ range for data collection/°	4.954 to 50.498
Index ranges	-9 ≤ h ≤ 9, -22 ≤ k ≤ 23, -18 ≤ l ≤ 18
Reflections collected	31852
Independent reflections	4195 [R_{int} = 0.0510, R_{sigma} = 0.0278]
Data/restraints/parameters	4195/0/251
Goodness-of-fit on F^2	1.136
Final R indexes [I>=2σ (I)]	R_1 = 0.0258, wR_2 = 0.0585
Final R indexes [all data]	R_1 = 0.0284, wR_2 = 0.0595
Largest diff. peak/hole / e Å^{-3}	0.46/-0.44

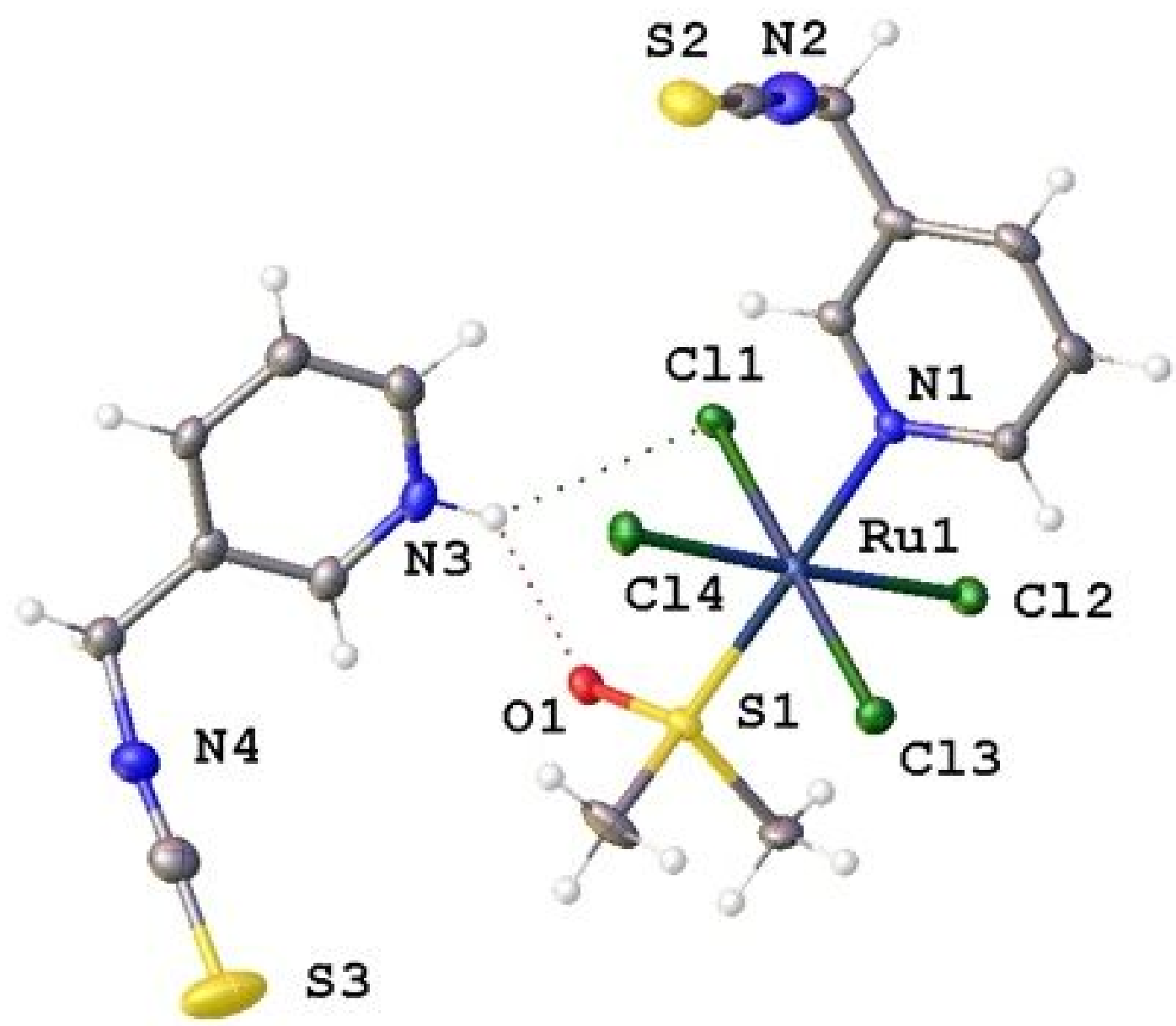

Figure S14. ORTEP view of **Ru12** with thermal ellipsoids depicted at the 30% probability level.

Table S12. Crystal data and structure refinement for **Ru12.**

Empirical formula	$C_{16}H_{19}Cl_4N_4ORuS_3$
Formula weight	622.40
Temperature/K	193.00
Crystal system	Monoclinic
Space group	$P2_1/n$
a/Å	11.5636(3)
b/Å	15.0168(3)
c/Å	14.3970(4)
α/°	90
β/°	105.1210(10)
γ/°	90
Volume/Å^3	2413.46(10)
Z	4
ρ_{calc}g/cm^3	1.713
μ/mm^{-1}	1.368
F(000)	1244.0
Crystal size/mm^3	0.35 × 0.2 × 0.12
Radiation	MoKα (λ = 0.71073)
2Θ range for data collection/°	4.04 to 50.496

Index ranges	$-13 \leq h \leq 13$, $-17 \leq k \leq 17$, $-17 \leq l \leq 17$
Reflections collected	32663
Independent reflections	4365 [R_{int} = 0.0454, R_{sigma} = 0.0277]
Data/restraints/parameters	4365/30/264
Goodness-of-fit on F^2	1.092
Final R indexes [I>=2σ (I)]	R_1 = 0.0240, wR_2 = 0.0532
Final R indexes [all data]	R_1 = 0.0268, wR_2 = 0.0541
Largest diff. peak/hole / e $Å^{-3}$	0.59/-0.41

Anticancer activity evaluation of complexes Ru11 and Ru12.

Table S13 IC_{50} values of **L2** and **L6** (μM), and gemcitabine (nM) against human non-malignant cells and human cancer cells.

Compound	HaCaT (Non-malignant keratinocyte)	A549 (Lung adenocarcinoma)		MeWo (Melanoma)		T24 (Bladder cancer)	
	IC50 (95% CI)	IC50 (95% CI)	S.I.	IC50 (95% CI)	S.I.	IC50 (95% CI)	S.I.
L2	730 (590–910)	54 (47–62)	13.5	310 (150–660)	2.3	450 (380–550)	1.6
L6	1.7 (1.4–2.2)	1.6 (1.3–1.8)	1.1	1.3 (1.1–1.5)	1.35	0.52 (0.46–0.57)	3.4

Cells were exposed to the drugs for 72 h and then cell viability was determined with the resazurin assay. Selectivity index (S.I.) values were calculated as IC_{50} value in the non-malignant cells divided by the IC_{50} value in the cancer cells obtained in each independent experiment. Data are presented as the means and their corresponding 95% confidence intervals (95% CI). All data were obtained from at least three independent experiments (see Experimental for details)

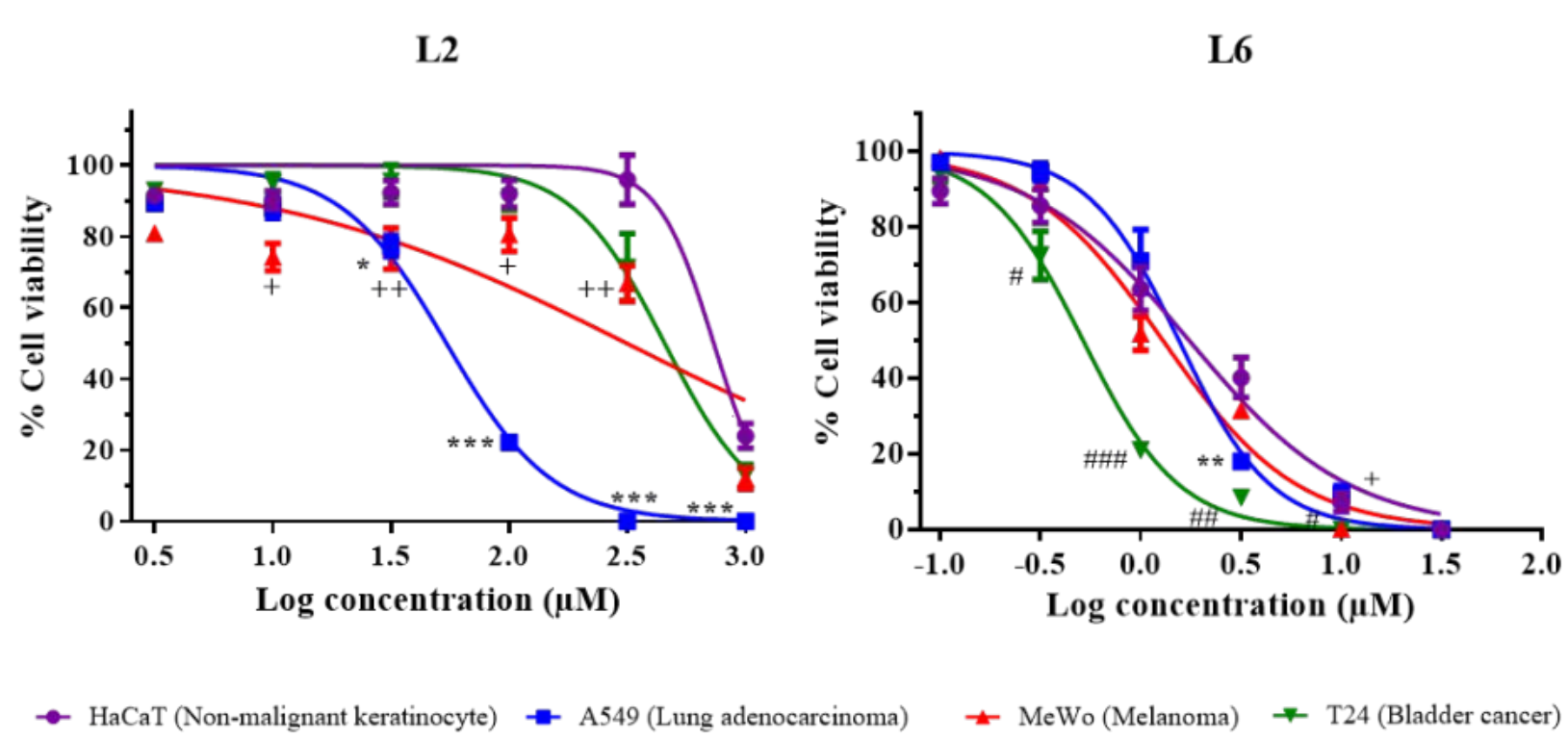

Figure S15. Evaluation of the selective anticancer activity of **L2** and **L6** on human cancer cells (A549, MeWo and T24) and human non-malignant cells (HaCaT). Cells were exposed to several concentrations of compounds for 72 hours. After treatment, cell viability was determined with the resazurin assay. Data represent mean ± SEM (μM), from at least three independent

experiment) Statistical analysis was calculated using the paired t-test; * indicates $p < 0.05$, ** indicates $p < 0.01$, ** indicates $p < 0.001$ (similar representations for marks + or #).

UV-Vis hydrolysis spectra of complexes Ru2, Ru6, Ru11 and Ru12.

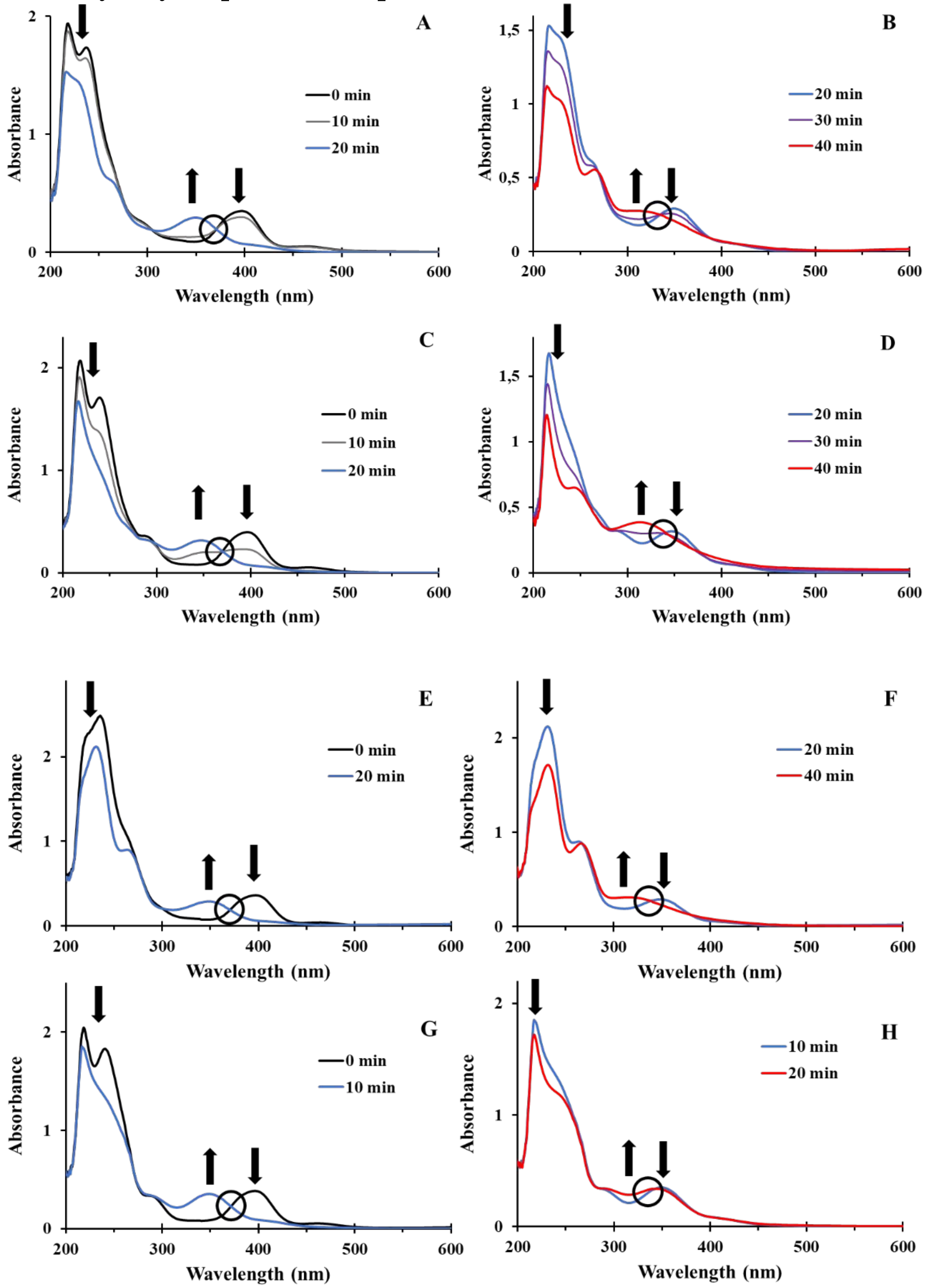

Figure S16. UV-Vis spectra of **Ru2 (A, B)**, **Ru6 (C, D)**, **Ru11 (E, F)** and **Ru12 (G, H)** incubated at 37 ˚C in PBS 0.1 mM at different times. Isosbestic points indicated with black circles.

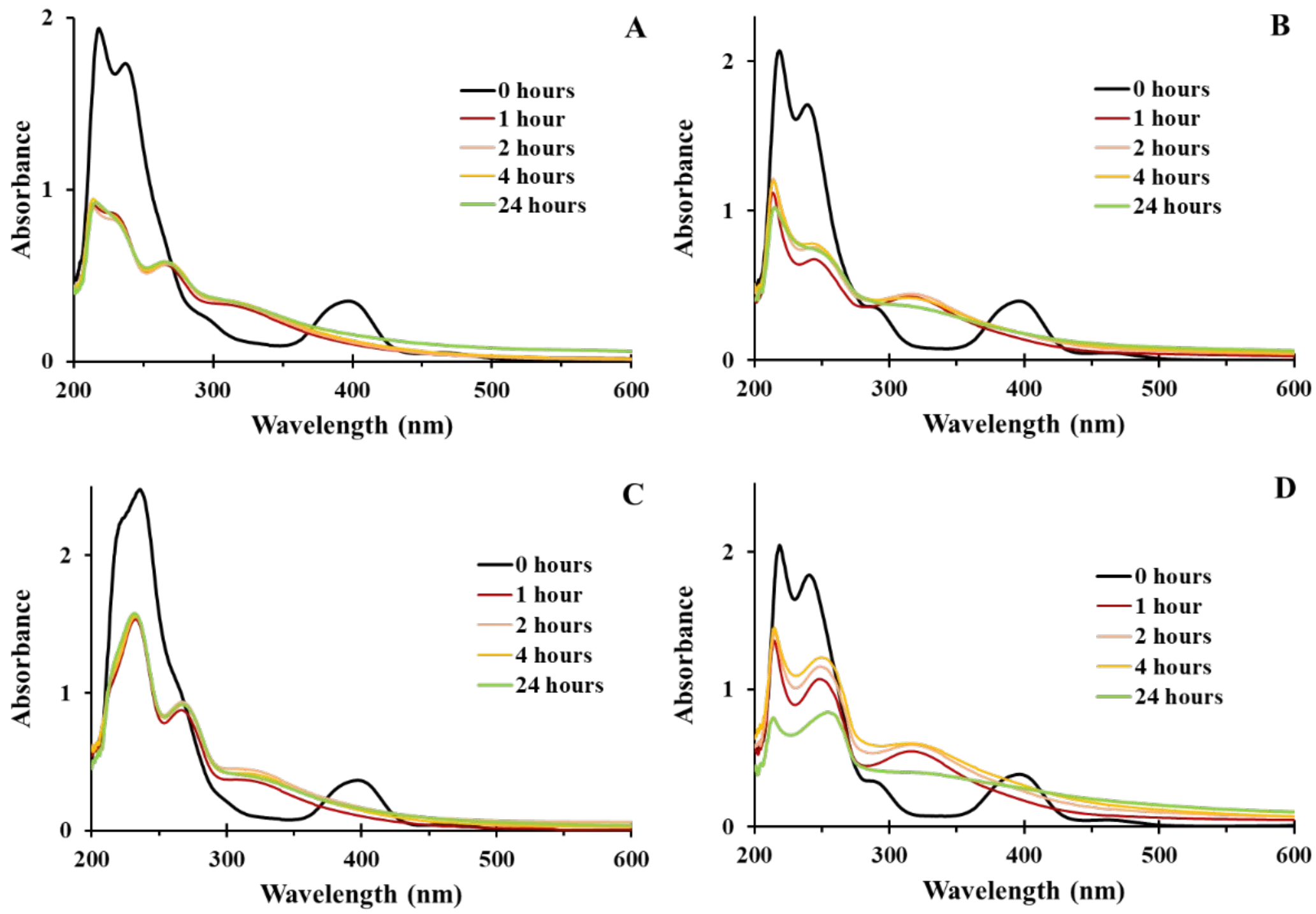

Figure S17. UV-Vis spectra of **Ru2 (A)**, **Ru6 (B)**, **Ru11 (C)** and **Ru12 (D)** incubated at 37 °C in PBS 0.1 mM at different times.

Ru uptake

Table S14 Cellular uptake of Ru complexes Ru2, Ru3, Ru6, Ru11, Ru12 and NAMI-A in A549 and T24 lines.

		Ru2	Ru3	Ru6	Ru11	Ru12	NAMI-A
**A549**	ng Ru/10^6 cells	0.402 ± 0.006	< LOD	< LOD	0.30 ± 0.03	< LOD	< LOD
	% Uptake	0.0104 ± 0.0006	< LOD	< LOD	0.0097 ± 0.0002	< LOD	< LOD
**T24**	ng Ru/10^6 cells	0.88 ± 0.03	0.44 ± 0.01	< LOD	0.6 ± 0.3	< LOD	0.54 ± 0.01
	% Uptake	0.037 ± 0.002	0.024 ± 0.001	< LOD	0.0250 ± 0.0009	< LOD	0.031 ± 0.002

Ru concentration determined by ICP-MS in A549 and T24 cells after 24 h incubation with the complexes **Ru2** (20 µM), **Ru3** (20 µM), **Ru6** (0.3 µM), **Ru11** (20 µM), **Ru12** (0.3 µM) and **NAMI-A** (20 µM). Data are presented as the mean ± standard error of the mean (SEM) of at least two independent experiments (see Experimental for details). < LOD indicates that the concentration of Ru in the sample was lower than the limit of detection (< 0.20 µg/L).

The Ru quantified in the cell pellet is presented in two different ways. As a concentration (ng Ru/10^6 cells), and % Uptake. The % Uptake represents the % of total Ru administered that was detected in the cell pellet.

ROS

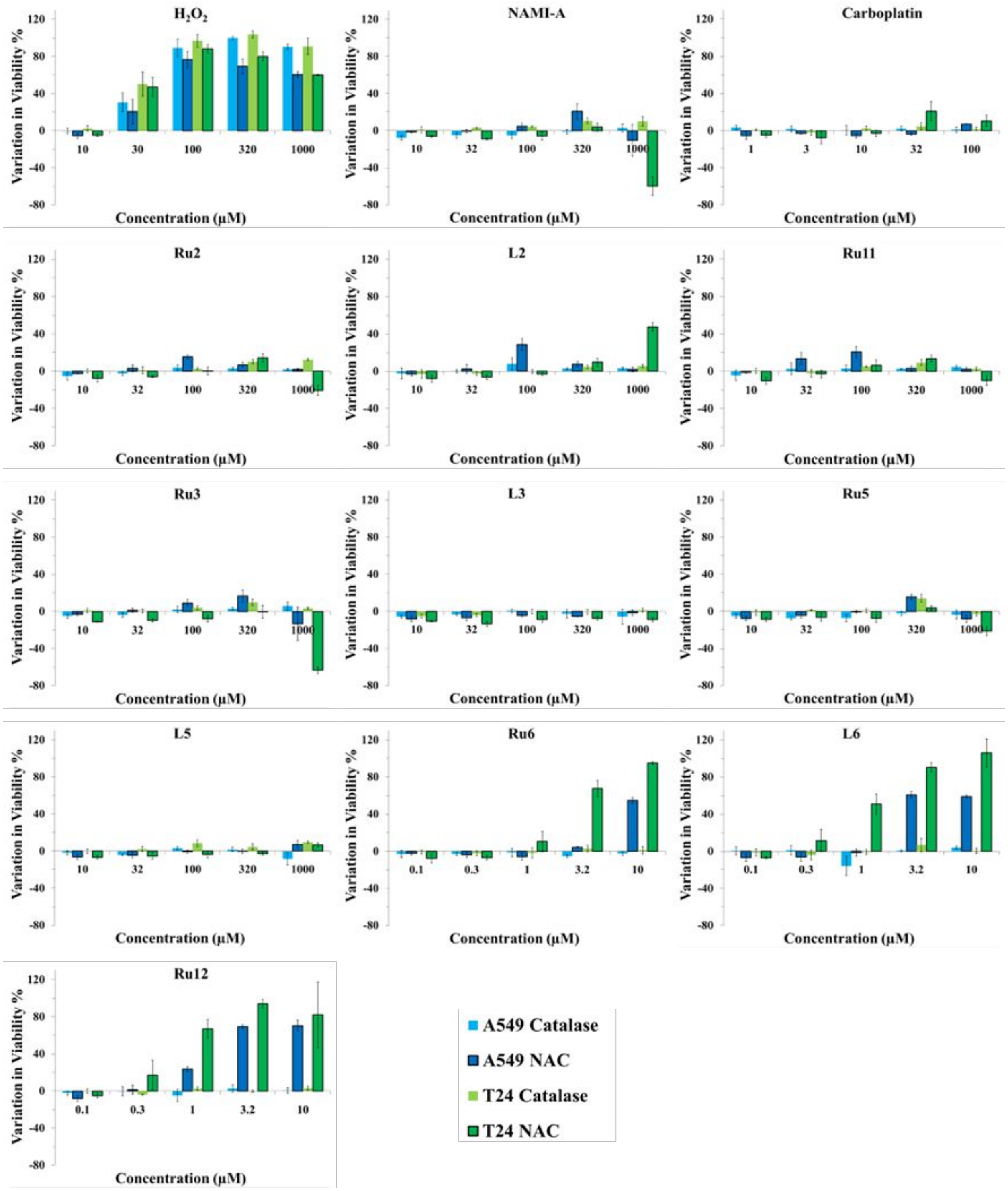

H_2O_2
NAMI-A
Carboplatin
Ru2
L2
Ru11
Ru3
L3
Ru5
L5
Ru6
L6
Ru12
Variation in Viability %
Concentration (µM)
A549 Catalase
A549 NAC
T24 Catalase
T24 NAC

Figure S18. Assessment of the contribution of ROS generation to the cytotoxicity of the ruthenium complexes. A549 (blue) and T24 (green) cells were treated at different concentrations for 72 hours in the absence or presence of catalase (light) or N-acetylcysteine (NAC, dark). Antioxidants were added 1 hour before compounds. Cell viability was determined by the resazurin assay. Data represent mean ± SEM from at least three independent experiments.

Docking studies

Autodock Vina 1.5.7 software was used for the docking between ligand and proteins.[2, 3] The structures of ALDH1A1, ALDH1A3 and ALDH3A1 proteins were obtained from the Protein Data Bank with the following PDB IDs: 4WPN, 5FHZ and 4H80 respectively.[4-9] The chemical structure of ligand was optimized with Gaussian at the B3LYP/cc-pvdz level and converted to pdb files.[10-13] Water molecules were deleted from the proteins and added both polar hydrogens and Kollman charges saved everything in their respective pdbqt file format. A grid box was built around the binding sites of the receptor proteins by making a grid box size of 40x40x40 with a grid spacing of 0.45 Å for ALDH1A1, a grid box size of 20x30x22 with a grid spacing of 1.0 Å for ALDH1A3, 21x30x18 with a grid spacing of 1.0 Å for ALDH3A1. Grid box center coordinates X; Y; Z: 37.006; 21.697; 18.786 for ALDH1A1. -0.141; 77.503; 69.471 for ALDH1A3 and 19.812; 2.926; -45.904 for ALDH3A1. The lowest binding energy (ΔG) was selected as the most favorable binding affinity site (Table S15 and S16). Interaction profiles and figures were obtained with Discovery Studio software version 24.1.0.23298.[14]

Aromatic aldehydes, such as benzaldehyde and its derivatives (e.g., N,N-diethylaminobenzaldehyde), are well known substrates and inhibitors of ALDH enzymes. Inhibition of these enzymes blocks a key cellular defense pathway, causing the accumulation of toxic aldehydes and increased oxidative stress, ultimately resulting in cytotoxicity. Given its structural similarity, **L2** could be considered a bioisostere of benzaldehyde, and a putative substrate for ALDH.

To better understand the cytotoxicity and selectivity of **L2**, **Ru2** and **Ru11** against the A549 cell line, docking studies with three ALDH isoforms, known to be overexpressed in A549 cells and related to cancer proliferation, were performed.[15-17] Calculations on these three ALDH isoforms were carried out using the most selective ligand, **L2**. For comparison, **L6**, the most active ligand against A549 (Table 1 and S13), and **L3**, which displays low cytotoxicity (Table 1) were also docked. The docking analysis of these ligands with the best fit binding affinity sites of the ALDH isoforms is depicted in table S15 and figures S19-S21.

Table S15. Docking scores of L2, L3 and L6 with ALDH1A1, ALDH1A3 and ALDH3A1.

Protein	Ligand	Docking affinity (kcal/mol)
ALDH1A1	L2	-4.6
	L6	-4.9
	L3	-4.6
ALDH1A3	L2	-4.8
	L6	-4.8
	L3	-4.7
ALDH3A1	L2	-5.2

	L6	-5.7
	L3	-5.1

Energetic results showed similar docking affinity for the three ligands, with **L6** exhibiting slightly stronger binding, followed by **L2** and lastly **L3** with the ALDH3A1 isoform. Docking affinity results of the ligands, as well as their Ru(III) complexes, cannot explain their anticancer activity and selectivity. The results obtained among the ligands, ranging from -5.7 to -4.6 kcal/mol (table S15), are relatively small and weak to be considered selective competitive inhibitors of ALDH, which typically exhibit binding affinities below -8 kcal/mol.[18-20] It was therefore not possible to correlate the theoretical binding affinities with enzyme inhibition.

Ligands L2, L3 and L6 with isoform ALDH1A1.

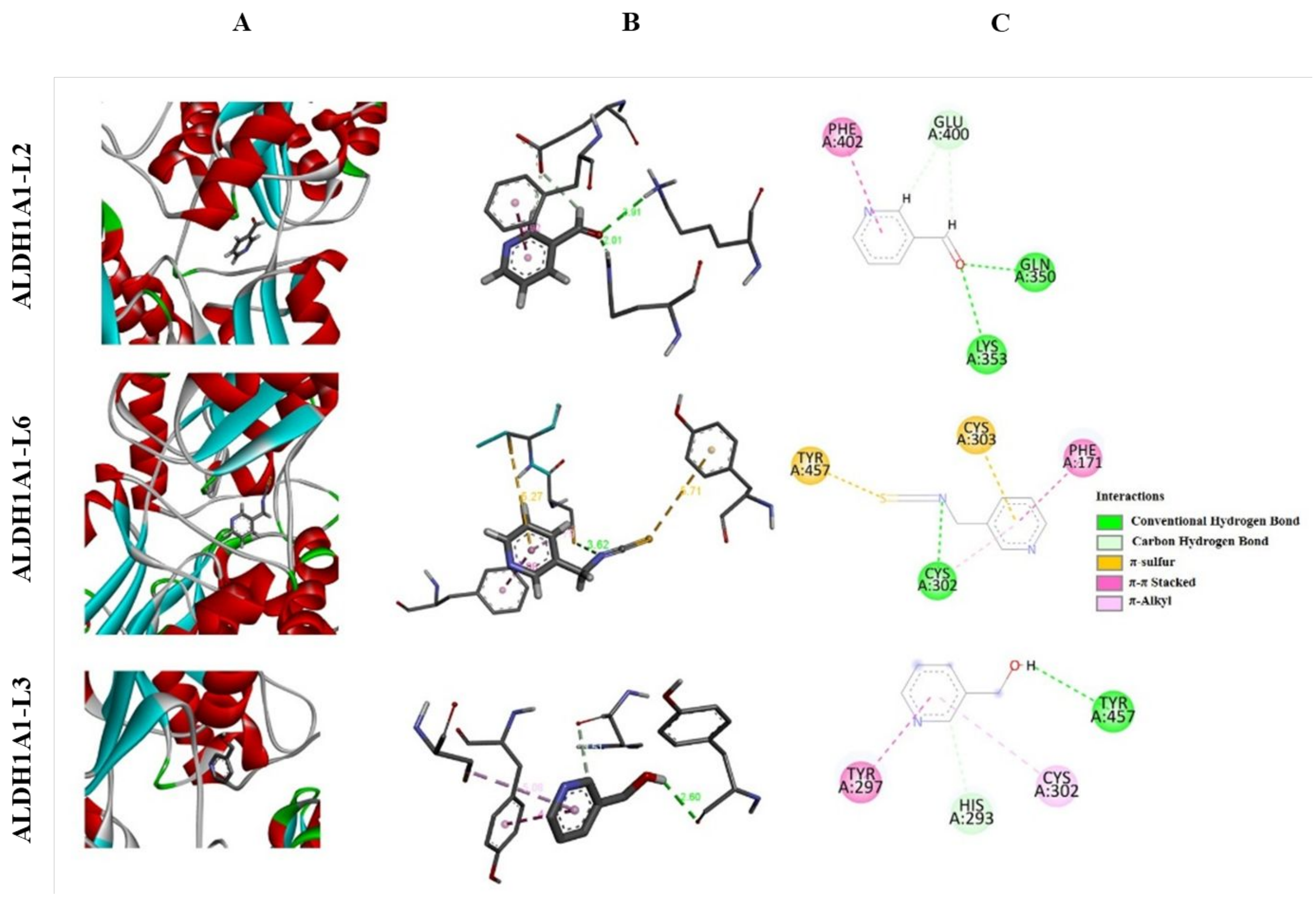

Figure S19. Interaction profile for the docking between the ligands of **Ru2** (**L2**), **Ru6** (**L6**) and **Ru3** (**L3**) with ALDH1A1 (PDB ID: 4WPN). **(A)** Binding site. **(B)** More favourable conformation between the receptor and ligand in 3D with bond distances (Å). **(C)** 2D interaction diagram.

Results from the docking between **L2**, **L6** and **L3** with ALDH1A1 are shown in Figure S19. For **L2** it is observed two conventional hydrogen bonds between the oxygen atom from aldehyde with GLN350 (2.01 Å) and LYS353 (2.91 Å) residues. It is also detected two carbon hydrogen bonds between the pyridine ring and the carboxaldehyde with GLU400 residue and a π-π interaction between the pyridine ring and PHE402 residue (3.92 Å). For **L6** there are two π-

sulfur interactions between TYR457 and CYS303 residues with isothiocyanatomethyl (5.71 Å) and pyridine (5.27 Å), respectively. It is observed a π-π, and π-alkyl interaction between the pyridine ring and PHE17, CYS3031 residues, respectively, Also, a conventional hydrogen bond is presented between the isothiocyanatomethyl group with CYS302 (3.62 Å). In the case of **L3**, the hydroxyl group and the pyridine ring interact via hydrogen bond interaction with TYR457 (2.60 Å) and HIS293 (3.51 Å) residues, respectively. The pyridine ring presents π-π, and π-alkyl interactions with TYR297 and CYS302, respectively.

Ligands L2, L3 and L6 with isoform ALDH1A3.

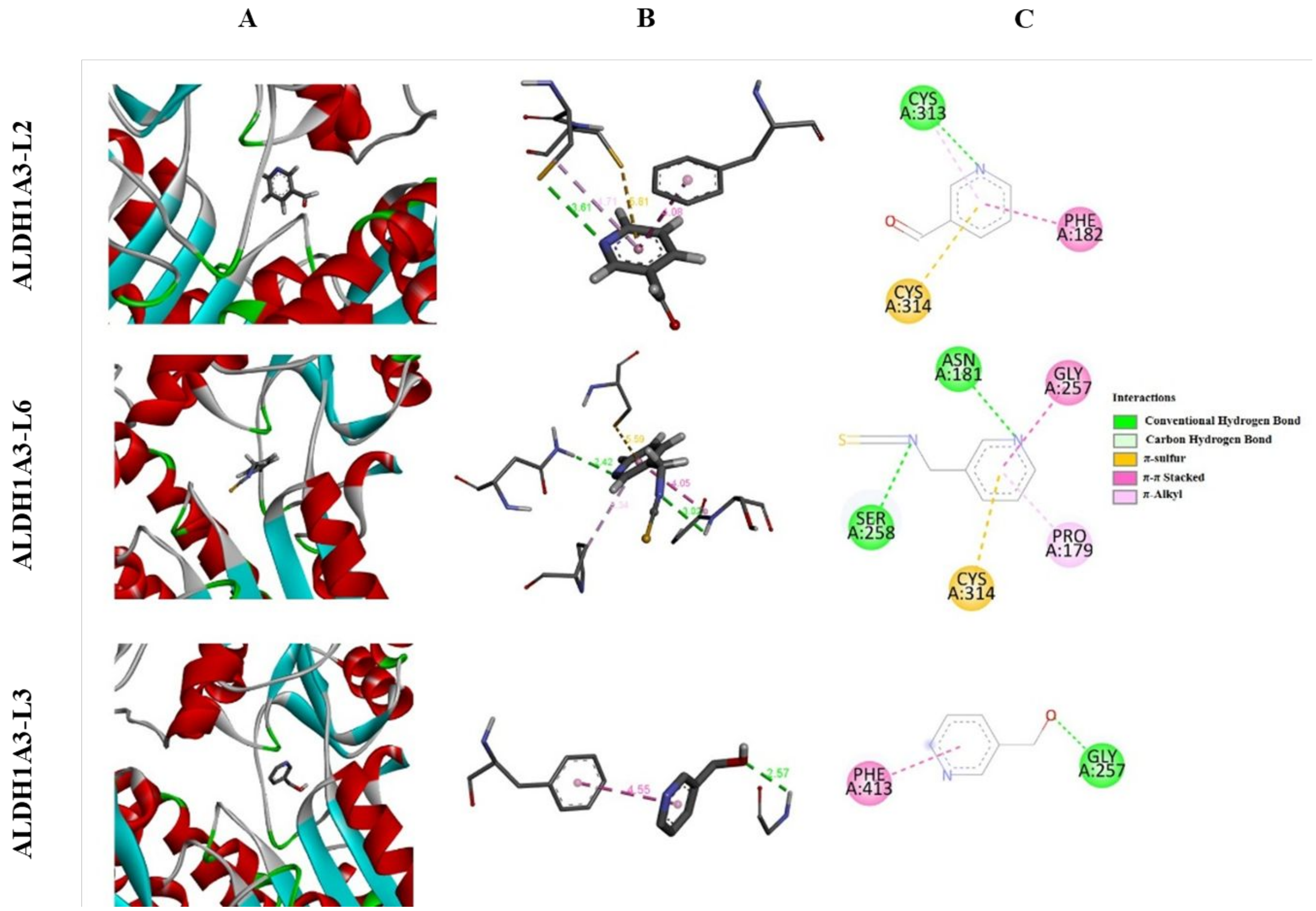

Figure S20. Interaction profile for the docking between the ligands of **Ru2** (**L2**), **Ru6** (**L6**) and **Ru3** (**L3**) with ALDH1A3 (PDB ID: 5FHZ). **(A)** Binding site. **(B)** More favourable conformation between the receptor and ligand in 3D with bond distances (Å). **(C)** 2D interaction diagram.

Figure S20 shows the interactions between **L2**, **L6** and **L3** with ALDH1A3. The pyridine ring from **L2** interacts with CYS313 (3.61 Å), PHE182 (4.08 Å) and CYS314 (5.81 Å) residues with conventional hydrogen bond, π-π and π-sulfur interactions, respectively. For **L6** the isothiocyanatomethyl and pyridine groups interact through conventional hydrogen bond with SER258 (3.02 Å) and ASN181 (3.62 Å), respectively. The pyridine ring interacts with CYS314

(5.59 Å), GLY257 (4.05 Å) and PRO179 (5.34 Å) residues via π-sulfur, π-π and π-alkyl interactions, respectively. L3 forms a conventional hydrogen bond between the oxygen atom from hydroxyl group and GLY257 (2.57 Å). The pyridine ring forms a π-π interaction with PHE413 (4.55 Å) residue.

Ligands L2, L3 and L6 with isoform ALDH3A1.

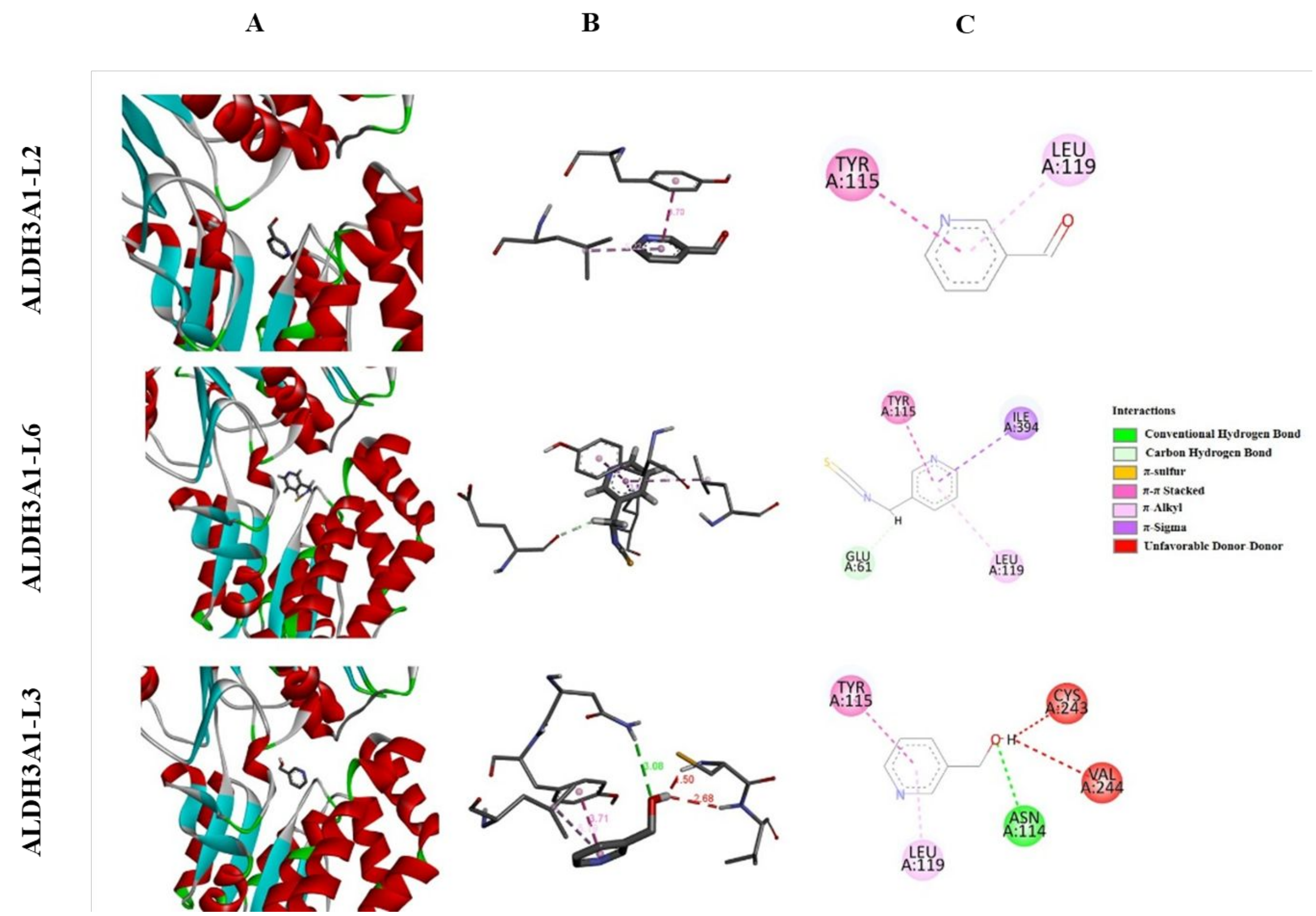

Figure S21. Interaction profile for the docking of ligands of **Ru2** (**L2**), **Ru6** (**L6**) and **Ru3** (**L3**) with ALDH3A1 (PDB ID: 4H80). **(A)** Binding site. **(B)** Most favourable receptor-ligand conformation in 3D, with bond distances (Å). **(C)** 2D interaction diagram.

Docking of **L2** with ALDH3A1 revealed π-π and π-alkyl interactions between the pyridine ring and TYR115 (3.70 Å) and LEU119 (5.22 Å), respectively. For **L6**, a conventional hydrogen bond was detected between the isothiocyanatomethyl group and the GLU61 residue (2.54 Å). Additional π-π, π-alkyl and π- σ interactions were observed between the pyridine ring and TYR115 (3.57 Å), LEU119 (5.27 Å) and ILE294 (3.97), respectively. In contrast, **L3** showed unfavorable interactions of the hydroxyl group with CYS243 and VAL244, as well as a conventional hydrogen bond with ASN114 (3.08 Å). The pyridine ring interacted with TYR115 (3.71 Å) and LEU119 (5.19 Å) through π-π and π-alkyl interactions, respectively.

Similar docking results were obtained for the corresponding Ru(III) complexes **Ru2**, **Ru3** and **Ru6** with the ALDH isoforms (Table S16 and figures S22-S24).

Complexes Ru2, Ru3 and Ru6.

Table S16. Docking scores of Ru2, Ru3 and Ru6 with ALDH1A1, ALDH1A3 and ALDH3A1.

Protein	Complex	Docking affinity (kcal/mol)
ALDH1A1	Ru2	-6.6

	Ru6	-6.7
	Ru3	-6.4
ALDH1A3	Ru2	-6,1
	Ru6	-6.1
	Ru3	-6.0
ALDH3A1	Ru2	-5.1
	Ru6	-5.6
	Ru3	-5.0

Based on the experimental results, **Ru2** and **Ru6** have been selected as the complexes with highest cytotoxic activity and compared to the non-cytotoxic **Ru3** as control. Thus, the three complexes have been docked with ALDH1A1, 1A3 and 3A1. Table S16 and Figures S22, S23, S24 shows the lowest binding energy conformation (ΔG) at the binding site selected as the most favourable binding affinity site.

Complexes **Ru2**, **Ru3** and **Ru6** with isoform ALDH1A1.

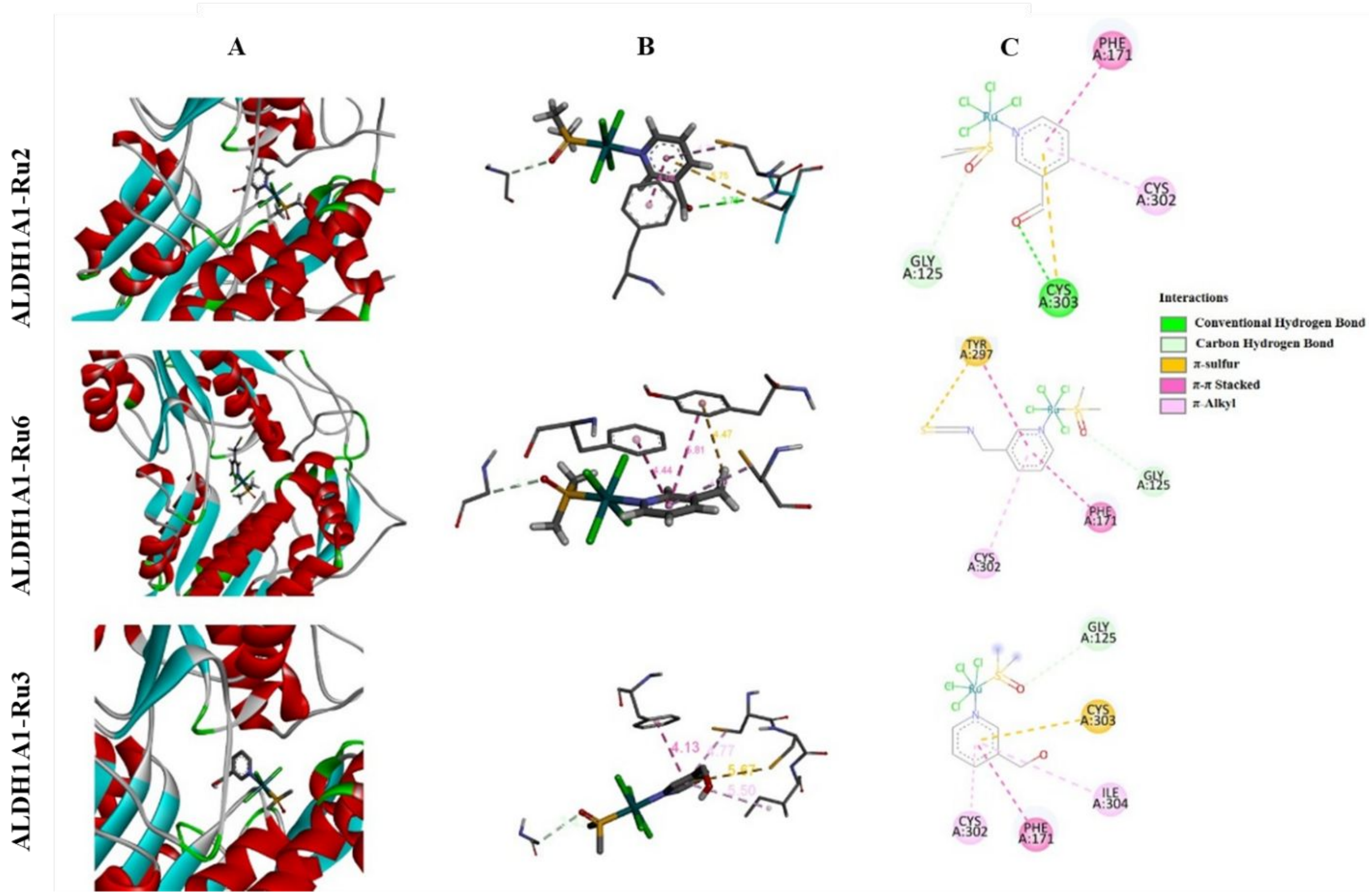

Figure S22. Interaction profile for the docking between **Ru2**, **Ru6** and **Ru3** with ALDH1A1 (PDB ID: 4WPN). **(A)** Binding site. **(B)** More favourable conformation between the receptor and ligand in 3D with bond distances (Å). **(C)** 2D interaction diagram.

Figure S22 shows the docking between **Ru2**, **Ru6** and **Ru3** with ALDH1A1. For the interaction with ALDH1A1, it is observed carbon hydrogen bond interactions between the GLY125 residue (around 3.2 to 3.4 Å) with the oxygen atom from the DMSO group of the complexes as well as π-π, and π-alkyl interactions with PHE171 and CYS302 residues, respectively (Figure S22). For **Ru2** it is observed a conventional hydrogen bond interaction between the CYS303 residue and

the oxygen atom from the carboxaldehyde. For Ru6 it is observed π-sulfur interactions between the isothiocyanatomethyl group and the TYR297 residue as well as this residue presents π-π interactions with the pyridine ring. For **Ru3** it is observed π-sulfur and π-alkyl interactions between the pyridine ring and CYS303 and ILE304 residues, respectively.

Complexes Ru2, Ru3 and Ru6 with isoform ALDH1A3.

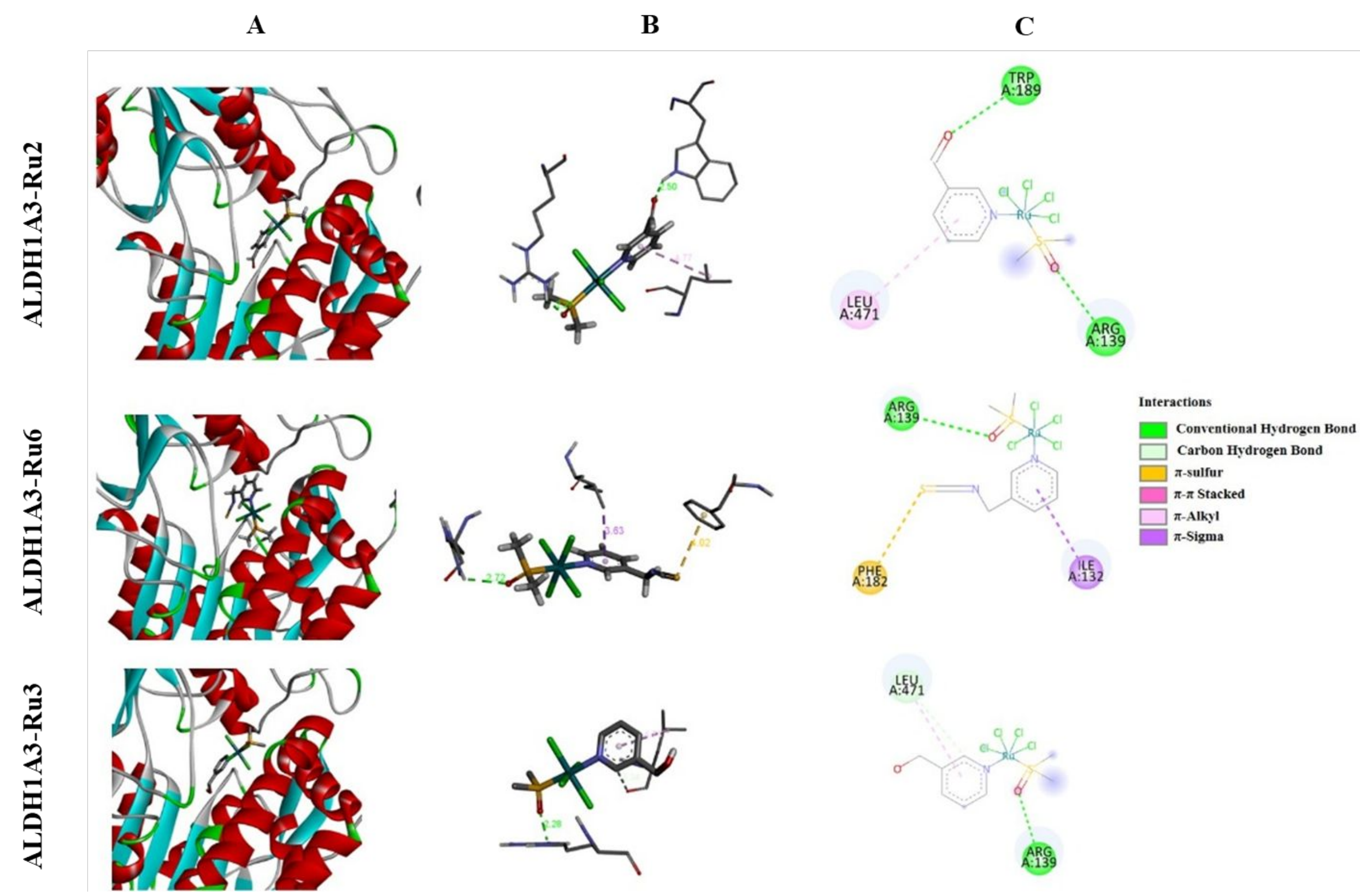

Figure S23. Interaction profile for the docking between Ru2, Ru6 and Ru3 with ALDH1A3 (PDB ID: 5FHZ). **(A)** Binding site. **(B)** More favourable conformation between the receptor and ligand in 3D with bond distances (Å). **(C)** 2D interaction diagram.

Figure S23 shows the interactions of **Ru2**, **Ru6** and **Ru3** complexes with ALDH1A3. It is observed conventional hydrogen bond interactions between the ARG139 residue with the oxygen atom from the DMSO group of the complexes. For **Ru2** it is observed a conventional hydrogen bond interaction between the TRP189 residue and the oxygen atom from the carboxaldehyde (2.50 Å), π-alkyl interaction between LEU471 and pyridine ring. For **Ru6** it is observed π-sulfur interactions between the isothiocyanatomethyl group and the PHE182 residue and π-sigma interaction between ILE132 and the pyridine ring (3.63 Å). For **Ru3** it is observed a carbon hydrogen bond with LEU471 residue and pyridine ring (3.34 Å).

Complexes Ru2, Ru3 and Ru6 with isoform ALDH3A1.

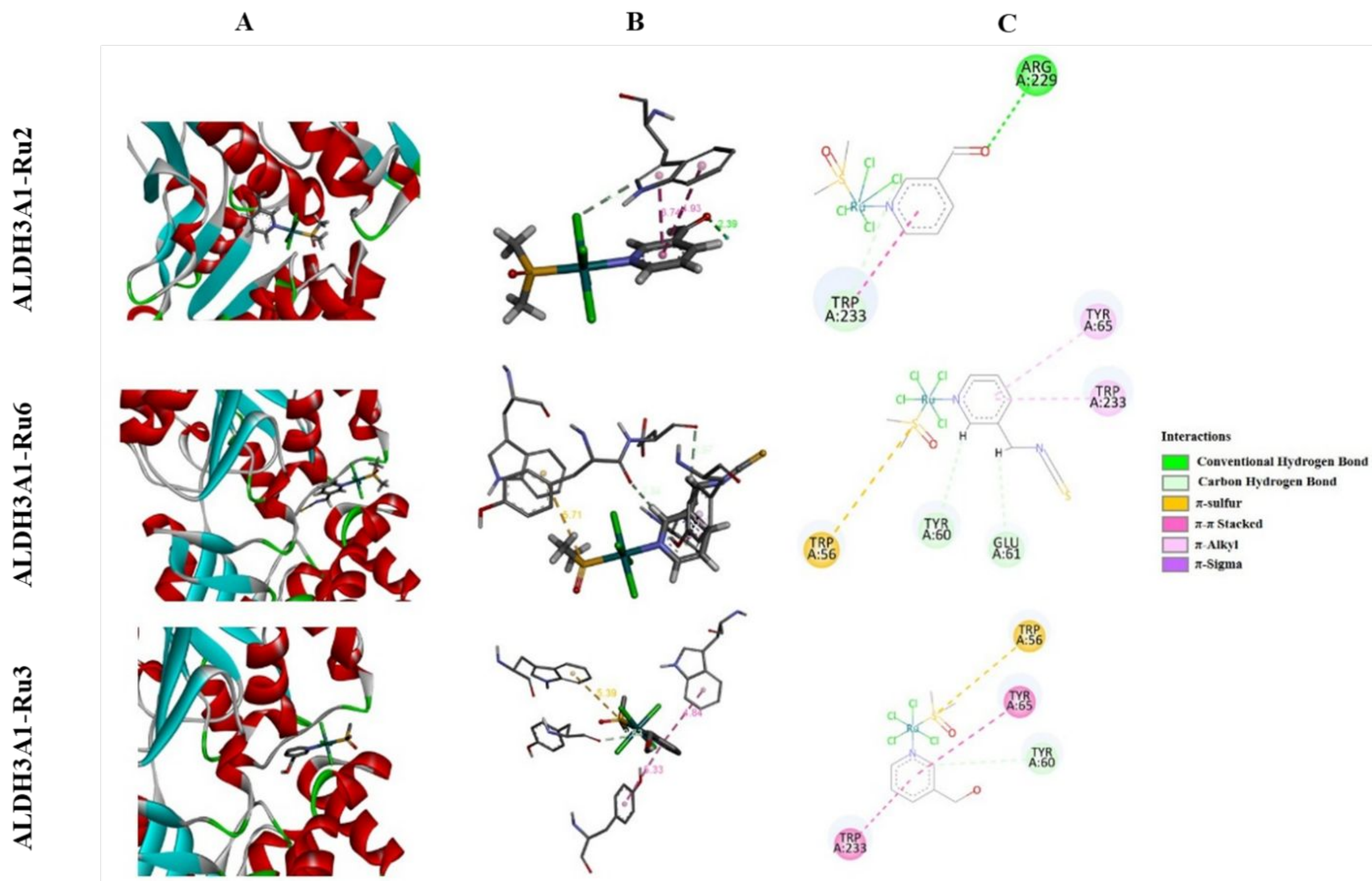

Figure S24. Interaction profile for the docking between Ru2, Ru6 and Ru3 with ALDH3A1 (PDB ID: 4H80). **(A)** Binding site. **(B)** More favourable conformation between the receptor and ligand in 3D with bond distances (Å). **(C)** 2D interaction diagram.

Figure S24 shows the results from the docking between **Ru2**, **Ru6** and **Ru3** with ALDH3A1. For **Ru2** it is observed conventional and carbon hydrogen bond interactions between the ARG229 residue with the oxygen atom from the DMSO (2.39 Å) and the TRP233 residue with the pryridine ring (3.47 Å), respectively. For **Ru6** complex it is observed two carbon hydrogen bond interactions between GLU61 and TYR60 residues, and the isothiocyanatomethyl group (2.97 Å) and pyridine ring (2.84 Å), respectively. It is also observed a π-sulfur interaction between the

TRP56 residue and DMSO group (5.71 Å) and π-alkyl interactions with TYR65 and TRP233.

For **Ru3** it is detected a carbon hydrogen bond interaction between the pyridine ring and TYR60 residue (3.53 Å), as well as π-π interactions with TYR65 (5.33 Å) and TRP233 (4.84 Å). Also, a π-sulfur interaction is observed between the DMSO group of **Ru3** and TRP56 residue (5.39 Å).

Energetic results from Table S16 show the docking affinity of **Ru2** and **Ru6** complexes are slightly favoured than **Ru3**. As a whole, the docking affinity follows the stability order of **Ru6** as the most favoured, followed by **Ru2** and **Ru3**. The interactions with ALDH1A1 are the most favoured followed by the docking with ALDH1A3 and ALDH3A1. However, similar docking affinity values were obtained for the docking between **Ru2** and **Ru6** with ALDH1A3 (-6.1 kcal/mol).

Even though the docking scores of the complexes are lower than the ones obtained for the ligands, and they follow the same trend as their anticancer activities: **Ru6** > **Ru2** > **Ru3** The results obtained do not allow a direct correlation between the cytotoxic activity of the three complexes and their binding affinities. The differences in energies among the complexes are not significant, especially between **Ru2** and **Ru3**, to be able to justify their differences in activities. Moreover, the low Ru uptake showed by the cells (Table S14) difficult the ALDH-complex interaction, due to the intracellular location of ALDH enzymes.

Effect of ligands in cell migration.

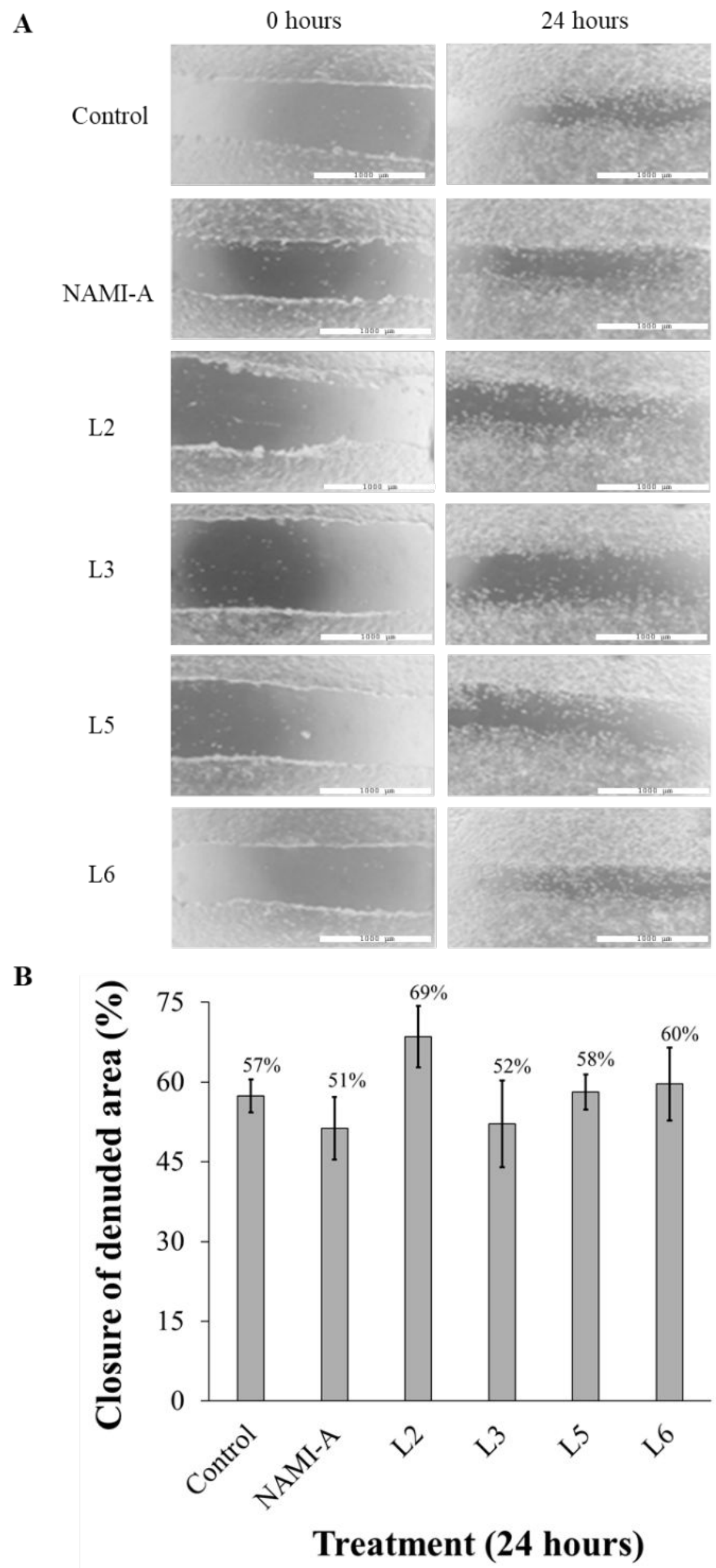

Figure S25. Effects of NAMI-A, **L2**, **L3**, **L5** and **L6** on migration of bladder cancer cells. T24 cells were seeded in a 6-well plate and incubated overnight. A wound area was generated, and cells were untreated or treated for 24 hours with NAMI-A (100 μM), **L2** (20 μM), **L3** (100 μM),

L5 (100 μM) and **L6** (0.3 μM). **(A)** Representative photographs at 4x magnification. **(B)** Quantification of the wound area. The closure of denuded area (%) was calculated as the ratio of the remaining wound area at 24 hours to the original wound area created at 0 hours. The data represent the means ± SEM of two independent experiments. The images and data for the controls and NAMI-A are the same as those in main Figure 8. The data were obtained from the same experiment but are shown in separate graphs to facilitate interpretation

^{1}H NMR characterization of ligands:

tert-butyl (pyridin-3-ylmethyl)carbamate (**L4**) ($CDCl_3$, 300 MHz):

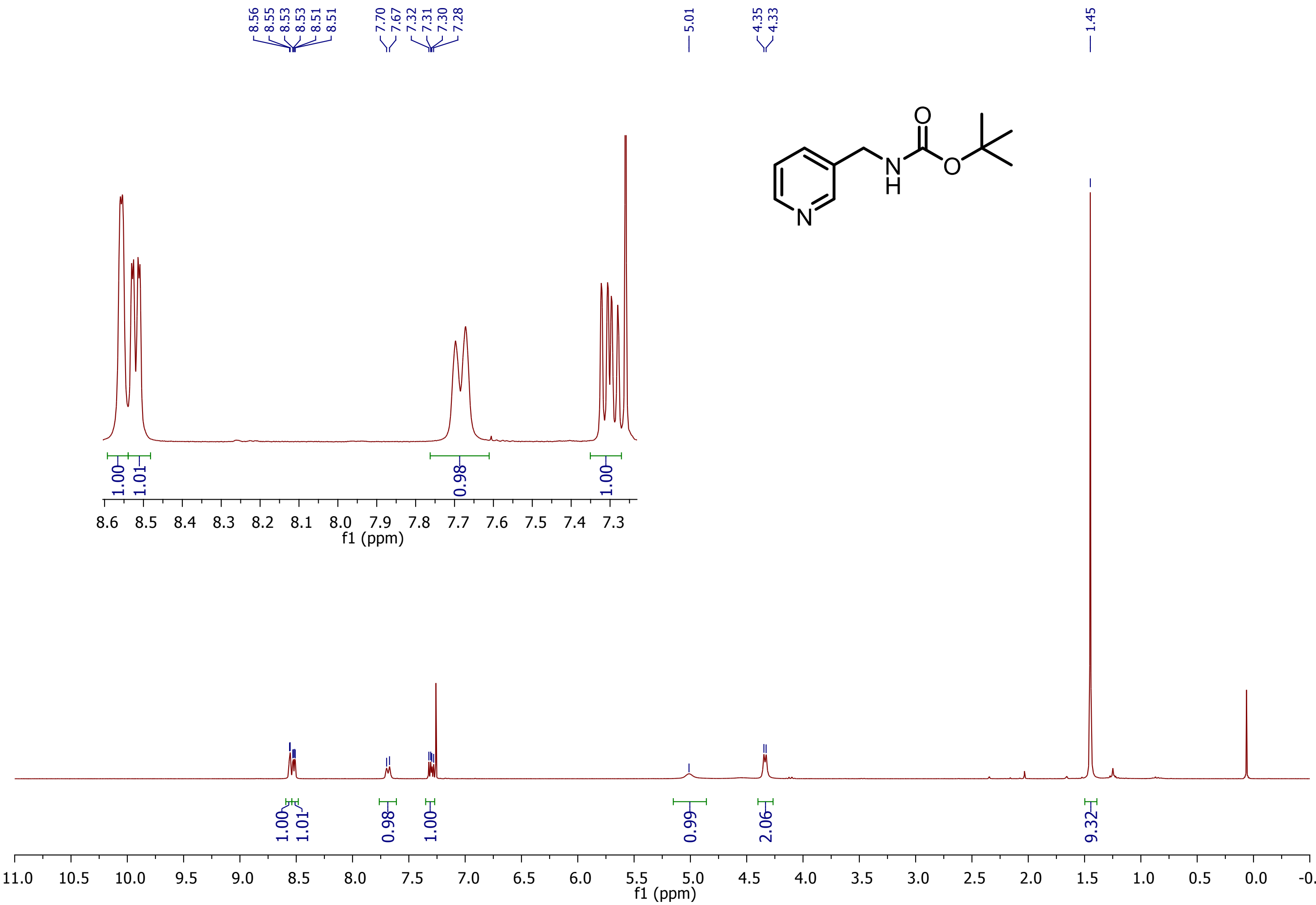

3-(isothiocyanatomethyl)pyridine (**L6**) ($CDCl_3$, 300 MHz)

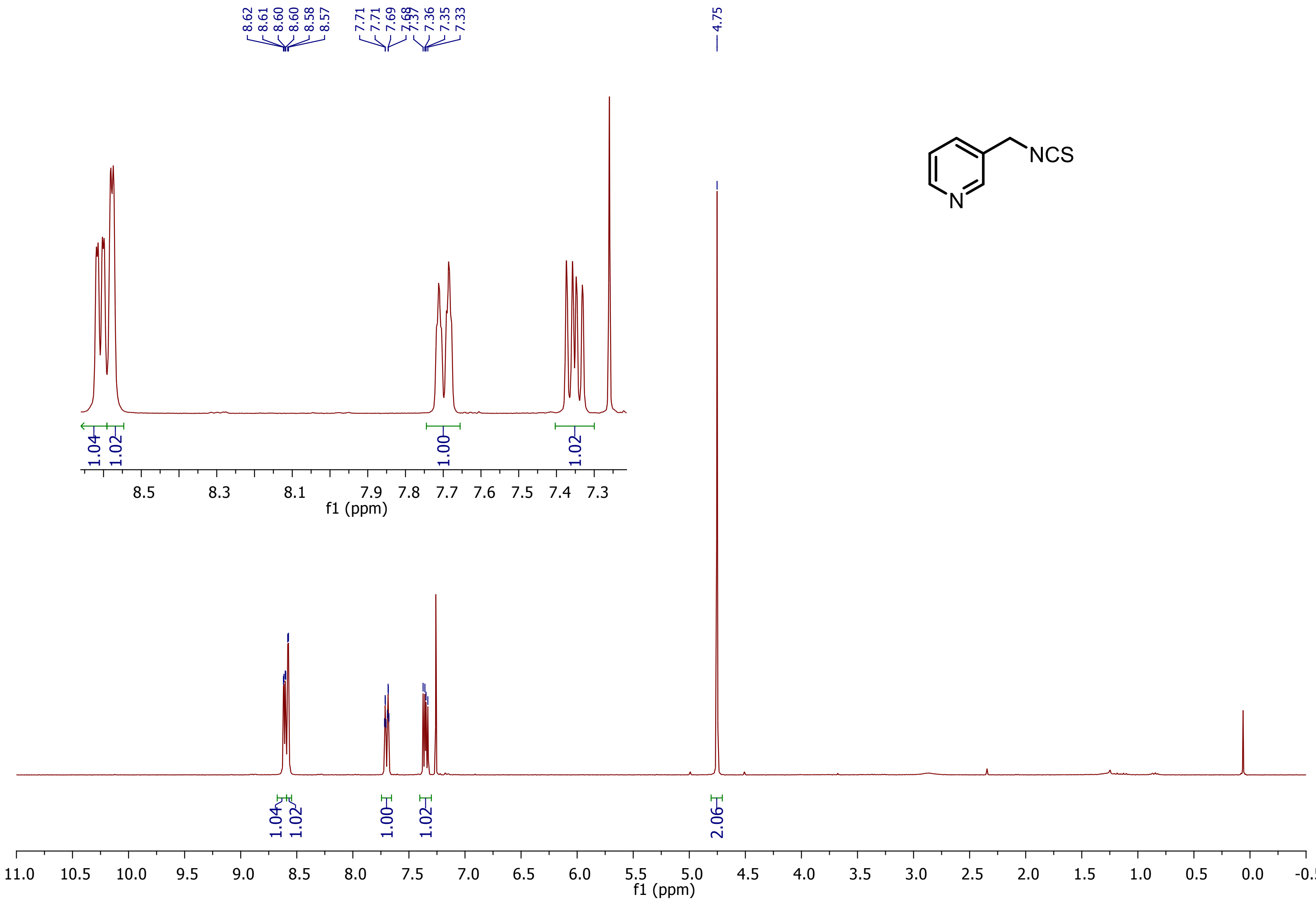

4-(azidomethyl)pyridinium chloride (**L8**) (D_2O, 300 MHz)

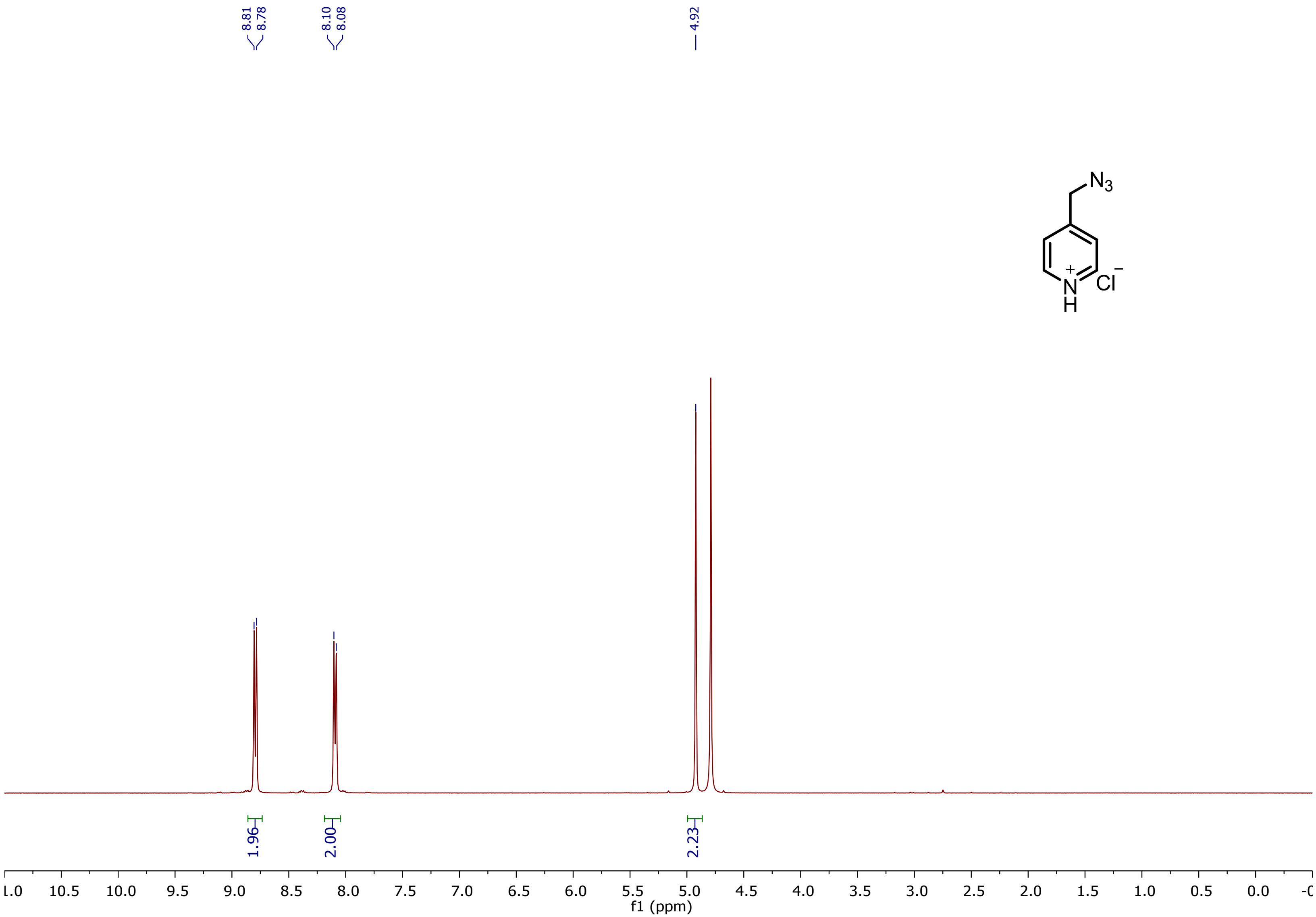

tert-butyl (pyridin-4-ylmethyl)carbamate (**L9**) (CD_3OD, 300 MHz):

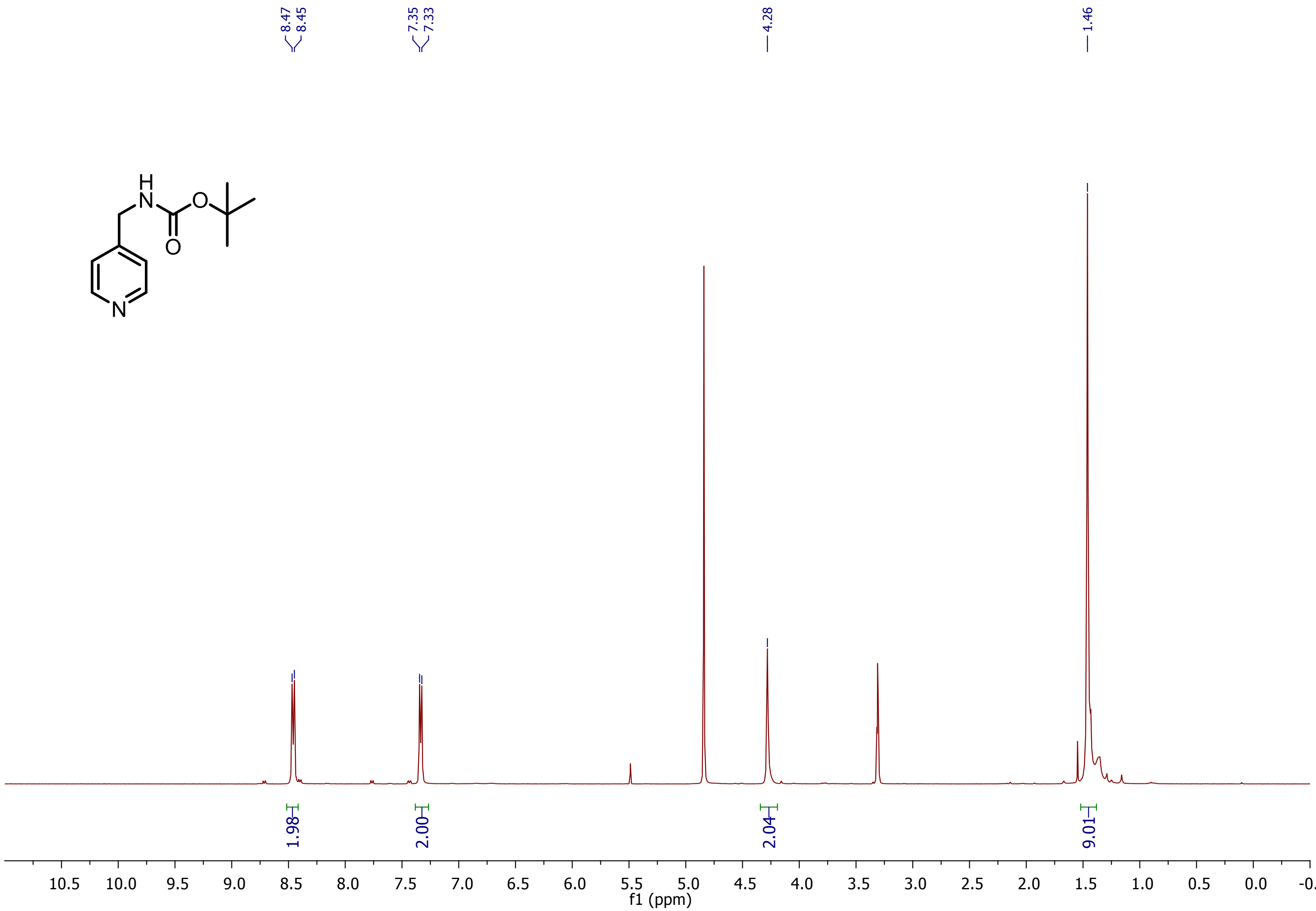

^{1}H NMR characterization of complexes:

(DMSO)$_2$H)[*trans*-RuCl$_4$(dmso-*S*)$_2$] **(Ru-P1)** (DMSO-d_6, 300 MHz):

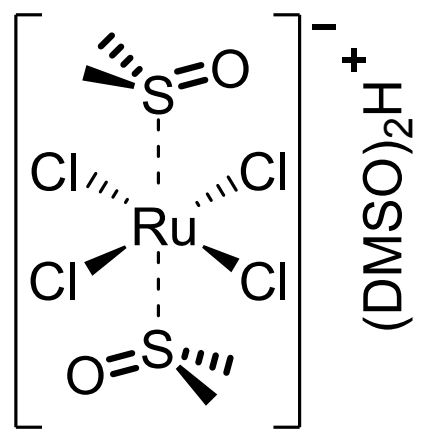

Ru-P1

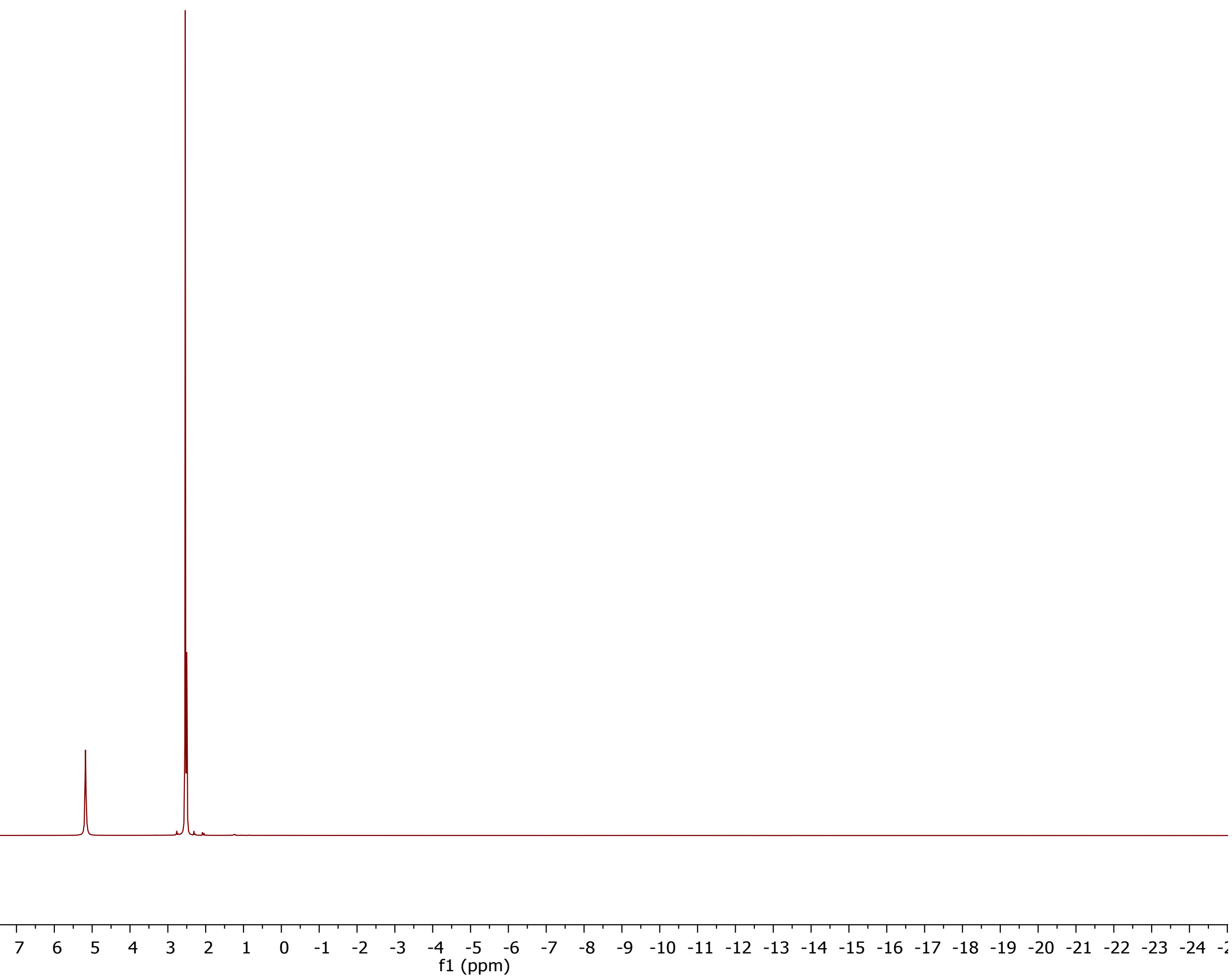

Na[*trans*-$RuCl_4$(dmso-*S*)$_2$] **(Ru-P2)** (DMSO-d_6, 300 MHz):

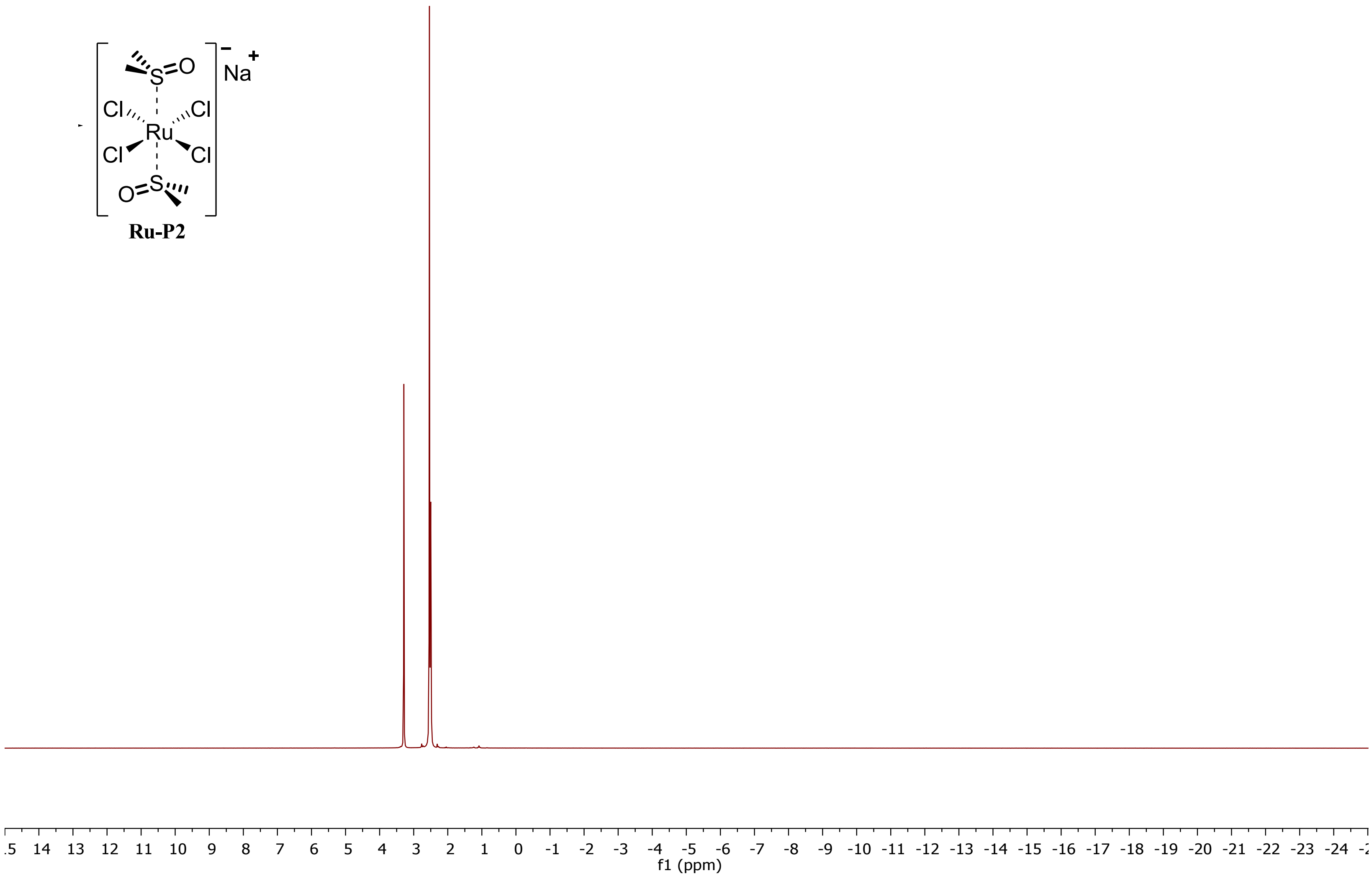

(ImH)[*trans*-RuCl$_4$(dmso-*S*) (Im)]) **NAMI-A** (DMSO-d_6, 300 MHz):

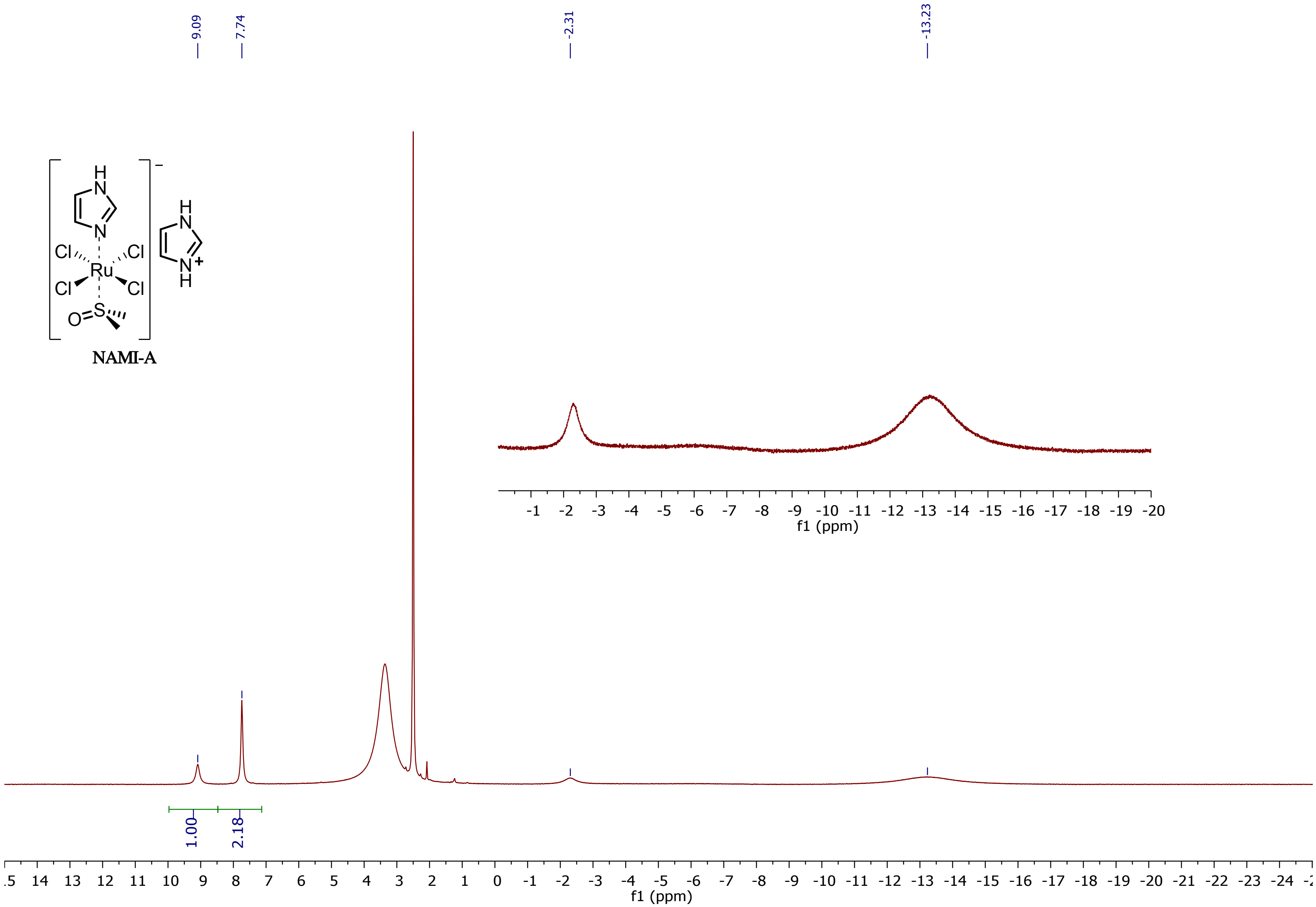

Na[*trans*-Ru(BrPy)Cl_4(dmso-*S*)] **(Ru1)** (DMSO-d_6, 300 MHz):

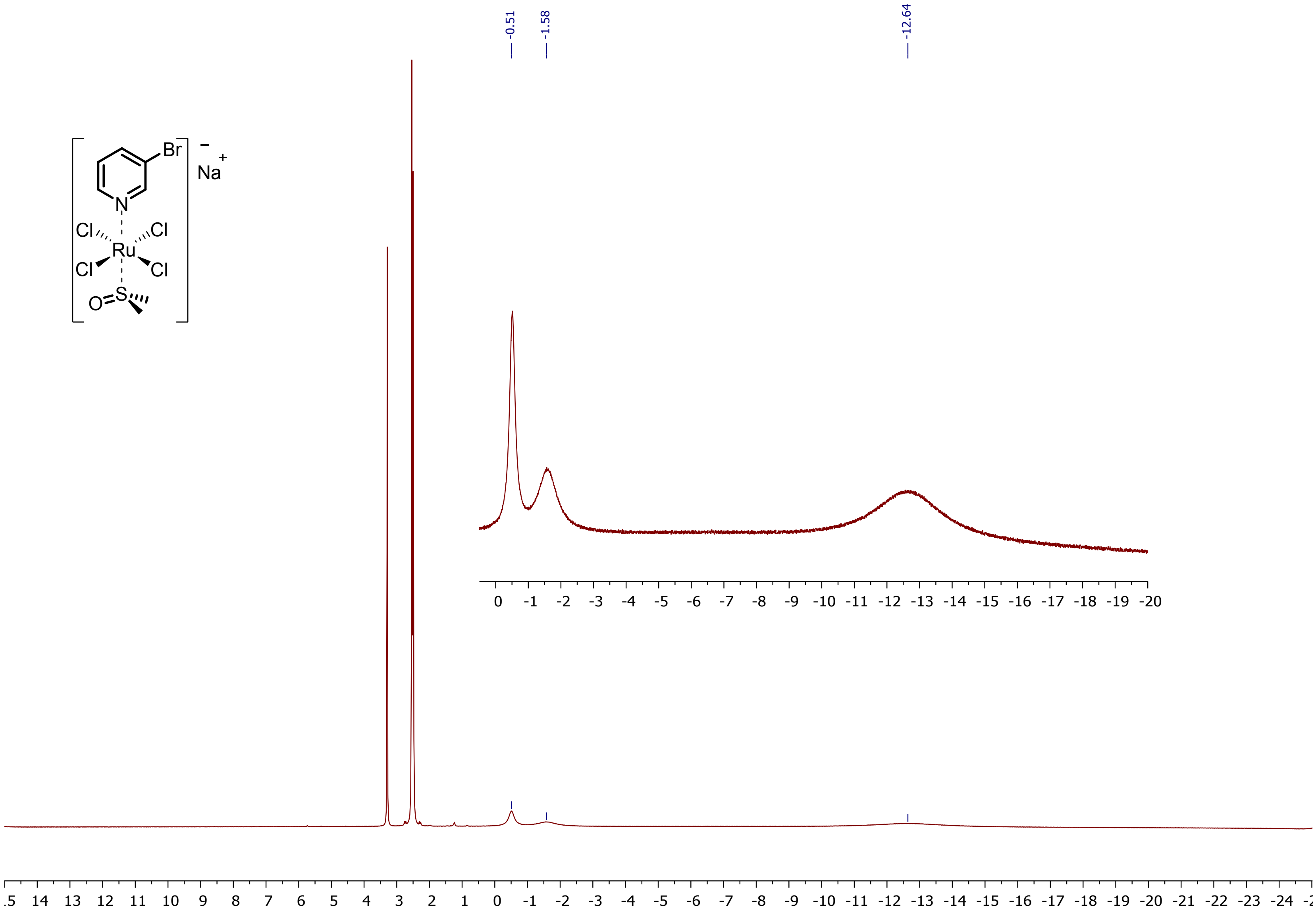

Na[*trans*-Ru(CHO-Py)Cl_4 (dmso-*S*)] **(Ru2)** (DMSO-d_6, 300 MHz):

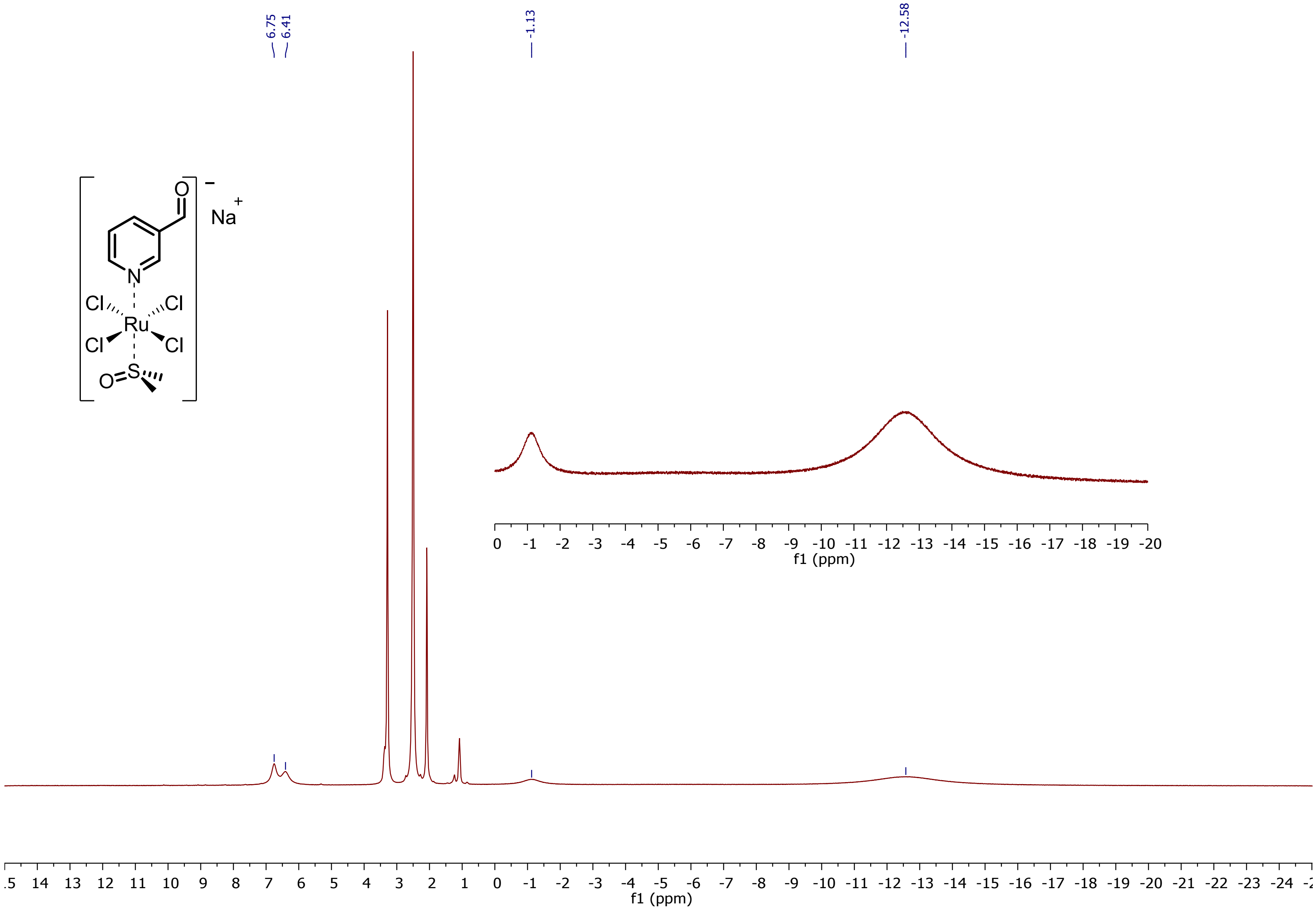
6.75
6.41
-1.13
-12.58
f1 (ppm)
f1 (ppm)
Na+

Na[*trans*-$RuCl_4$(dmso-*S*)(OH-Py)] **(Ru3)** (DMSO-d_6, 300 MHz):

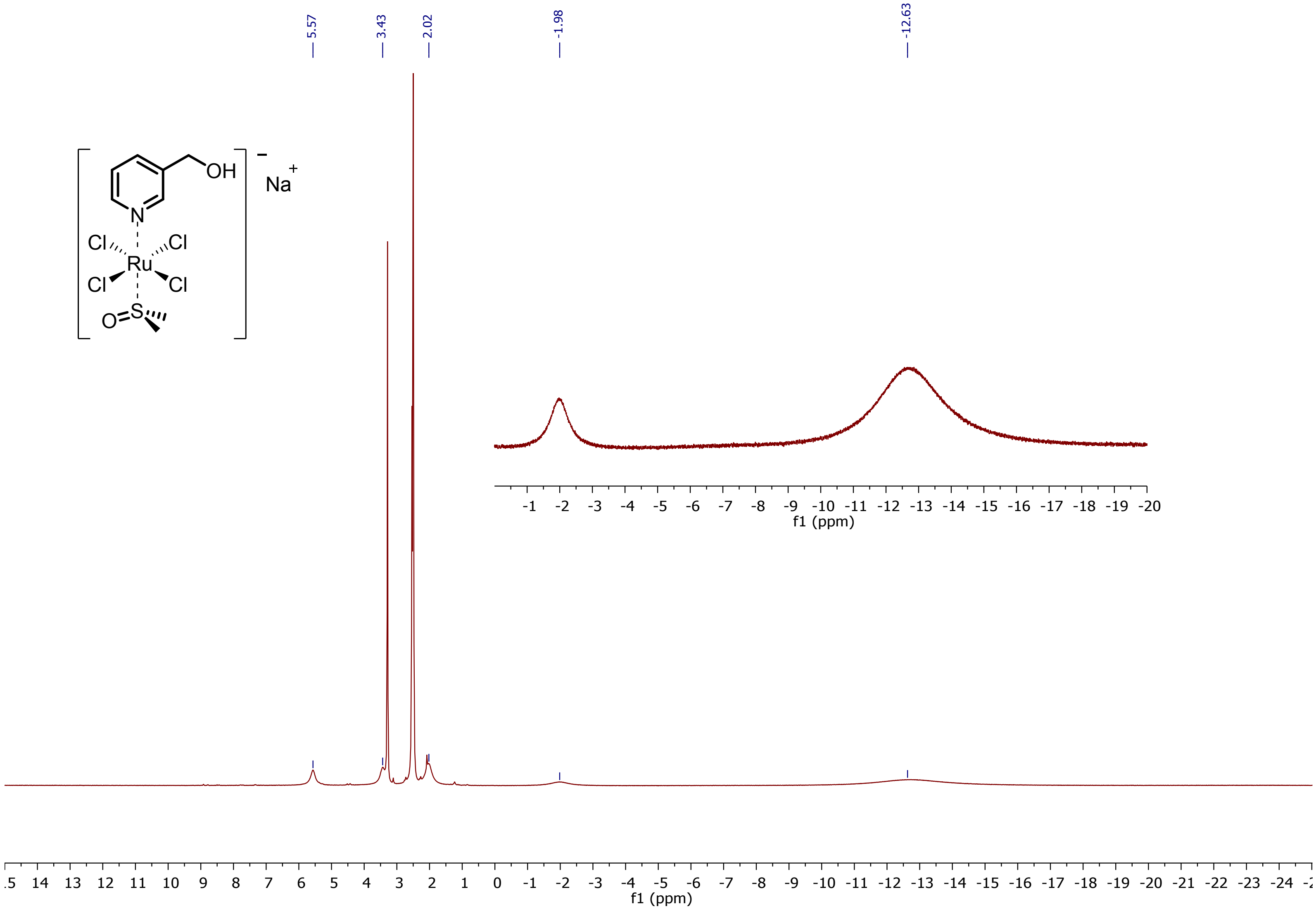

Na[*trans*-$RuCl_4$(dmso-*S*)(3-NBOC-Py)] **(Ru4)** (DMSO-d_6, 300 MHz):

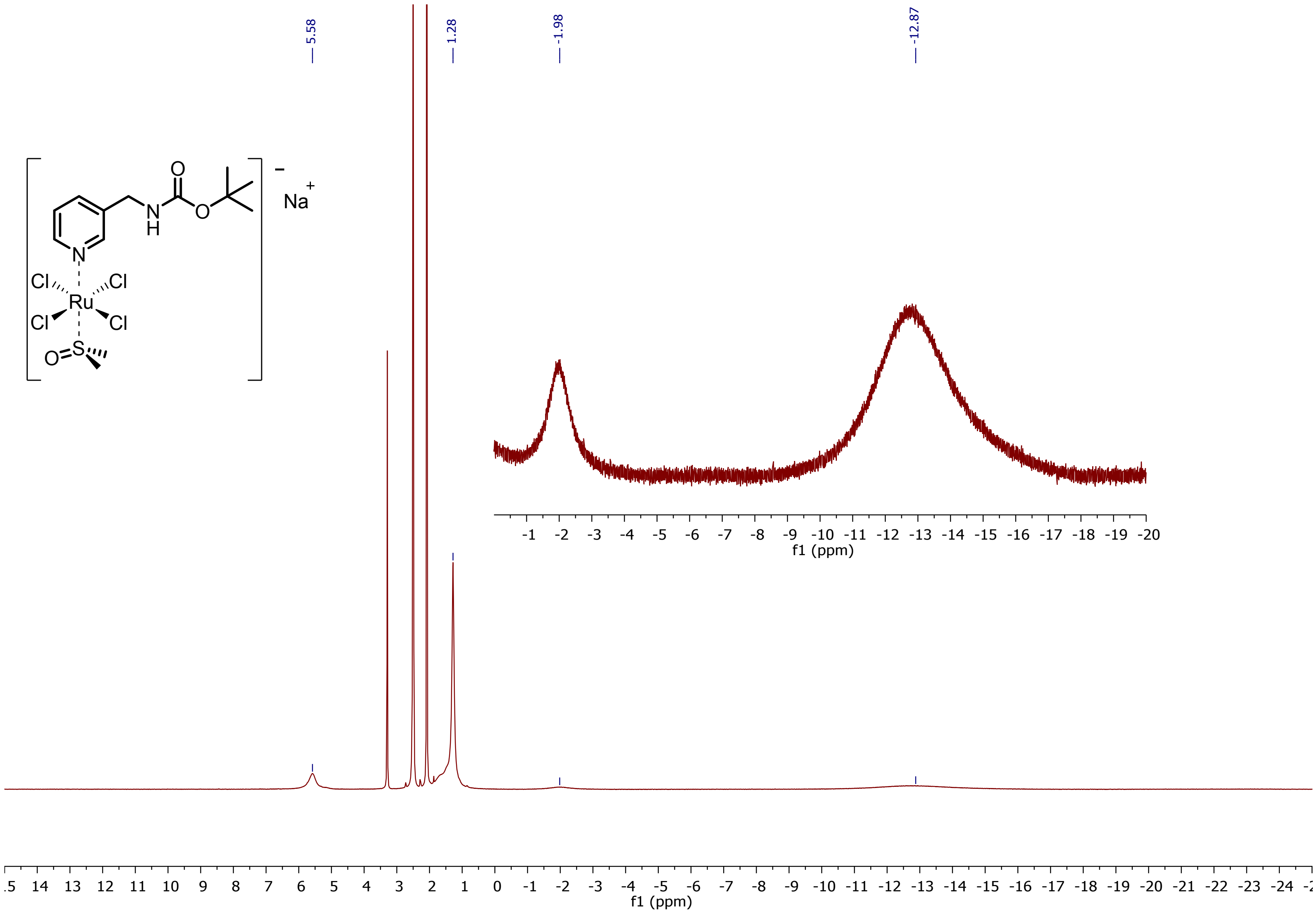

[*trans*-RuCl$_4$(dmso-*S*)(3-NH$_3$Py)] **(Ru5)** (DMSO-d_6, 300 MHz):

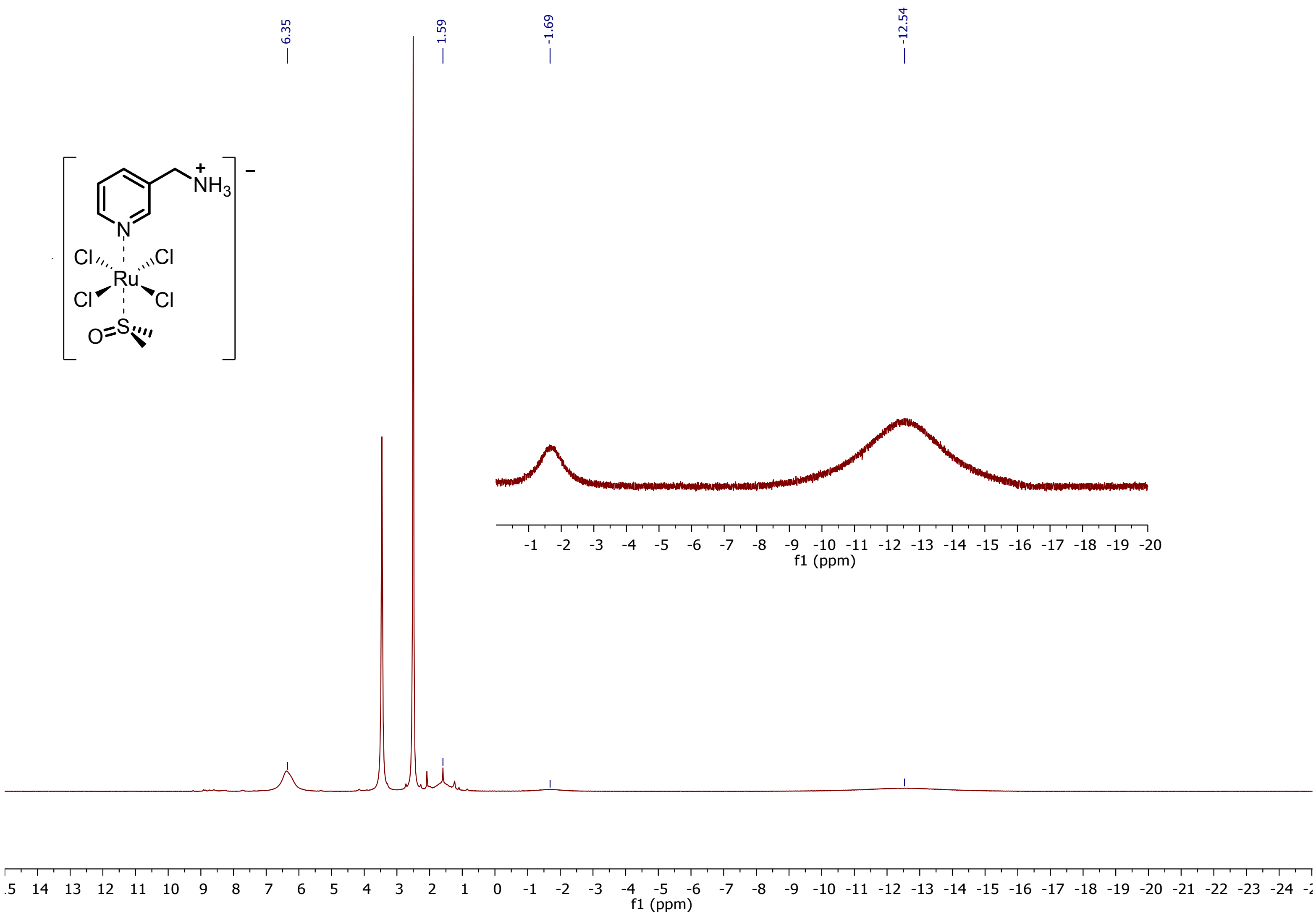

Na[*trans*-$RuCl_4$(dmso-*S*)(NCS-Py)] **(Ru6)** (DMSO-d_6, 300 MHz):

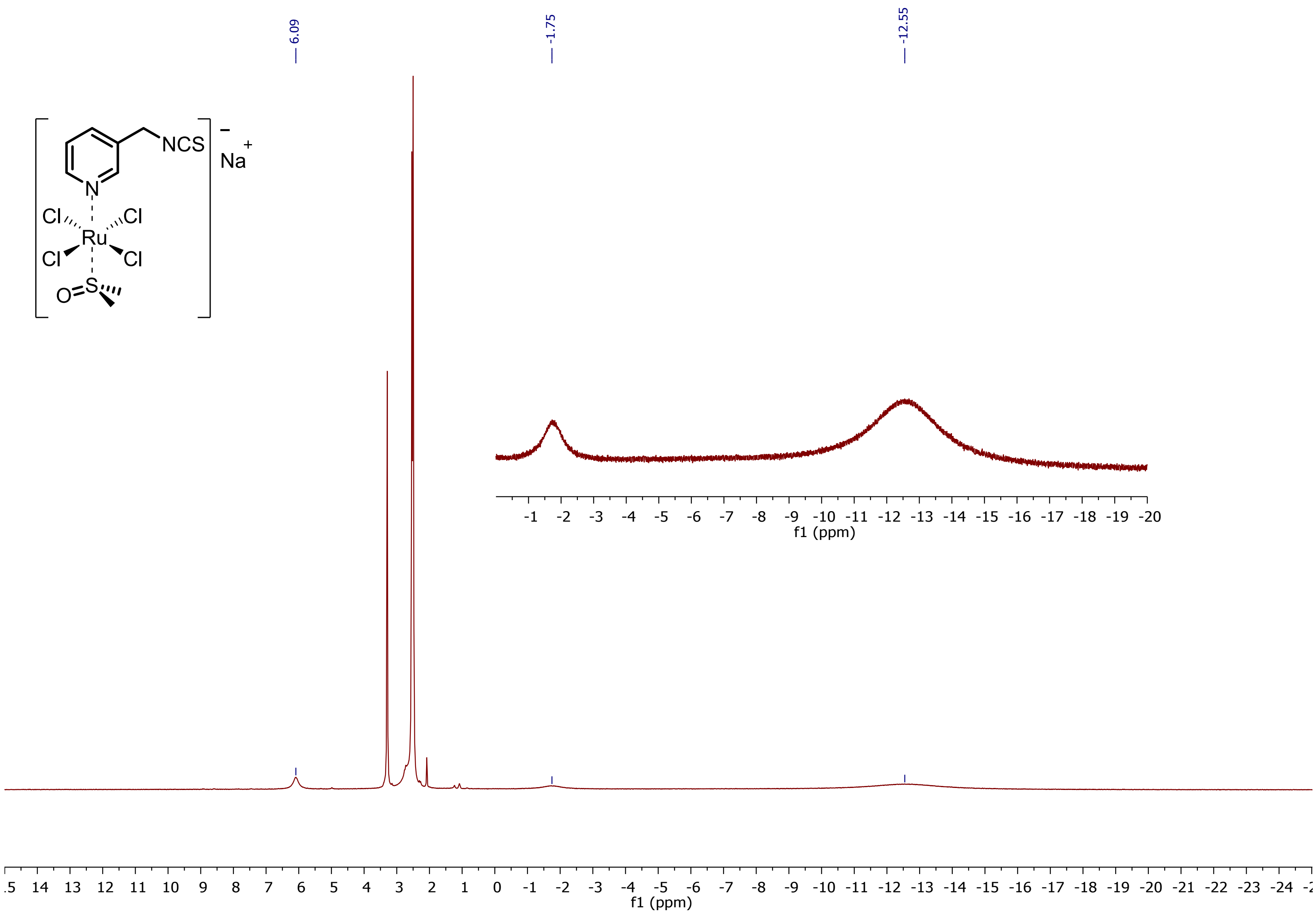

Na[*trans*-RuCl$_4$(CO$_2$Et)(dmso-*S*)] **(Ru7)** (DMSO-*d*$_6$, 300 MHz):

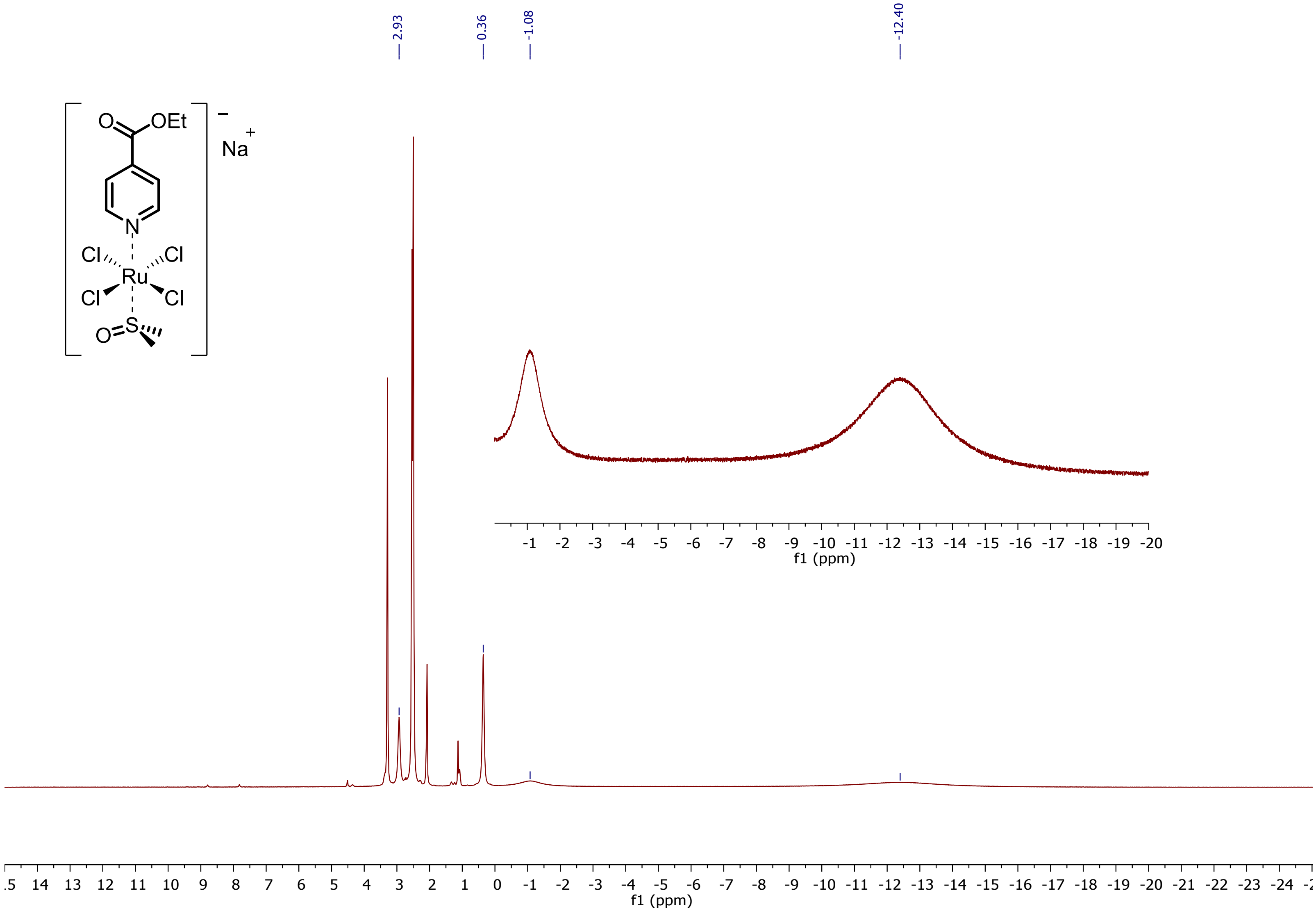

Na[*trans*-$RuCl_4$(dmso-*S*)(N_3-Py)] **(Ru8)** (DMSO-d_6, 300 MHz):

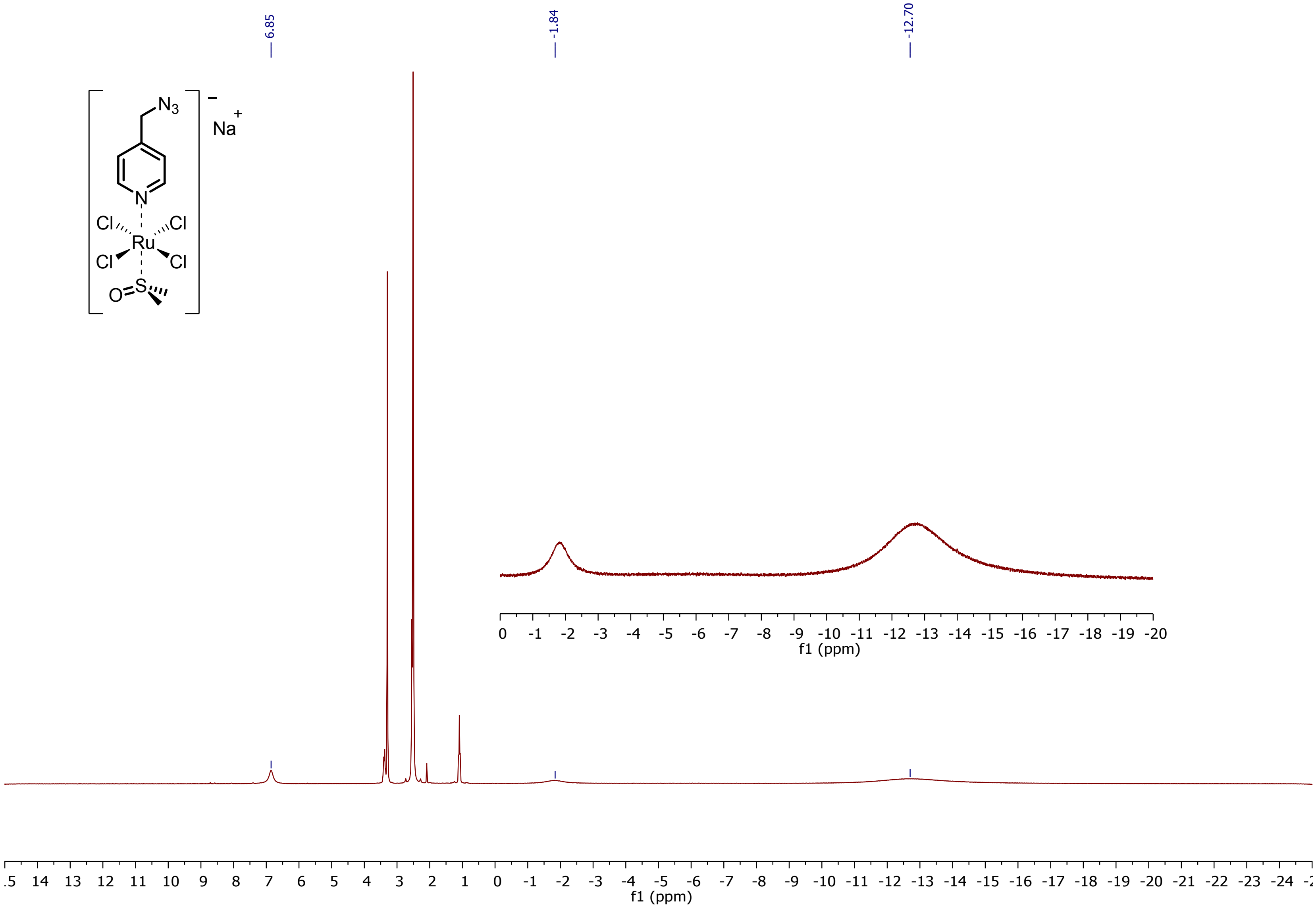
N3
Na+
N
Cl
Ru
Cl
Cl
Cl
O=S
6.85
-1.84
-12.70
0 -1 -2 -3 -4 -5 -6 -7 -8 -9 -10 -11 -12 -13 -14 -15 -16 -17 -18 -19 -20
f1 (ppm)
.5 14 13 12 11 10 9 8 7 6 5 4 3 2 1 0 -1 -2 -3 -4 -5 -6 -7 -8 -9 -10 -11 -12 -13 -14 -15 -16 -17 -18 -19 -20 -21 -22 -23 -24
f1 (ppm)

Na[*trans*-$RuCl_4$(dmso-*S*)(4-NBOC-Py)] **(Ru9)** (DMSO-d_6, 300 MHz):

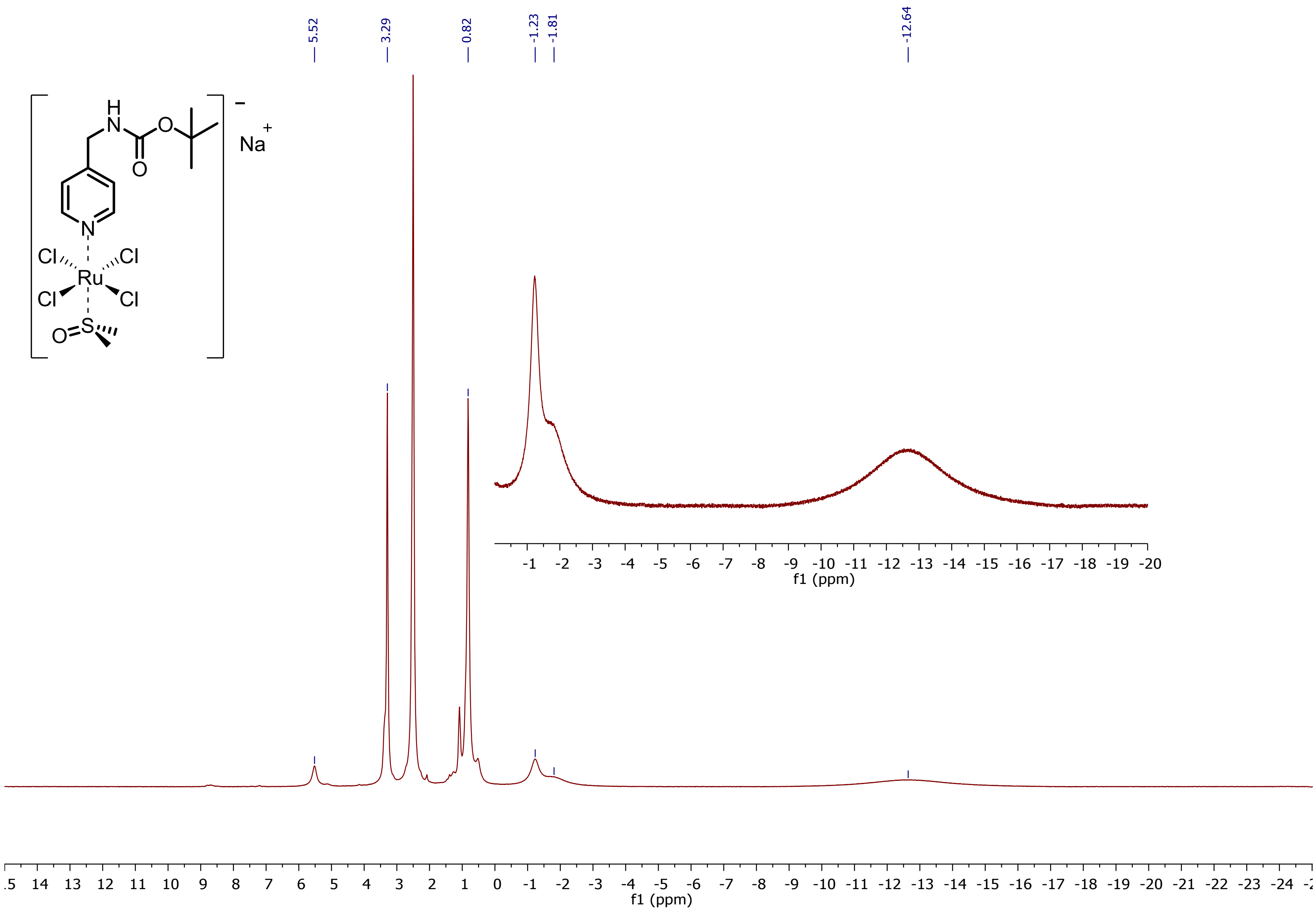

[*trans*-RuCl$_4$(dmso-*S*)(4-NH$_3$Py)] **(Ru10)** (DMSO-d_6, 300 MHz):

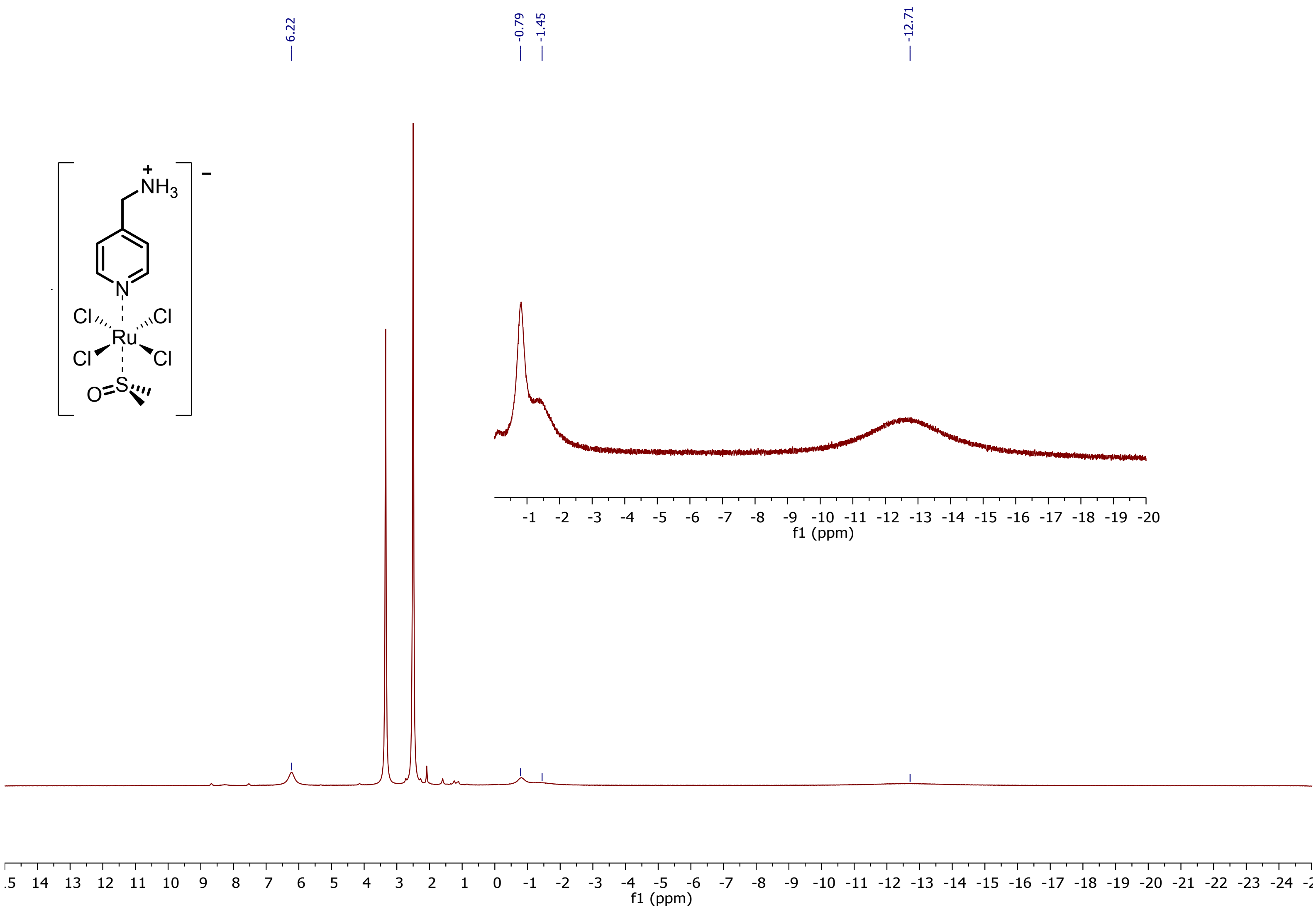

(CHO-PyH)[*trans*-Ru(CHO-Py)Cl_4 (dmso-*S*)] **(Ru11)** (DMSO-d_6, 300 MHz):

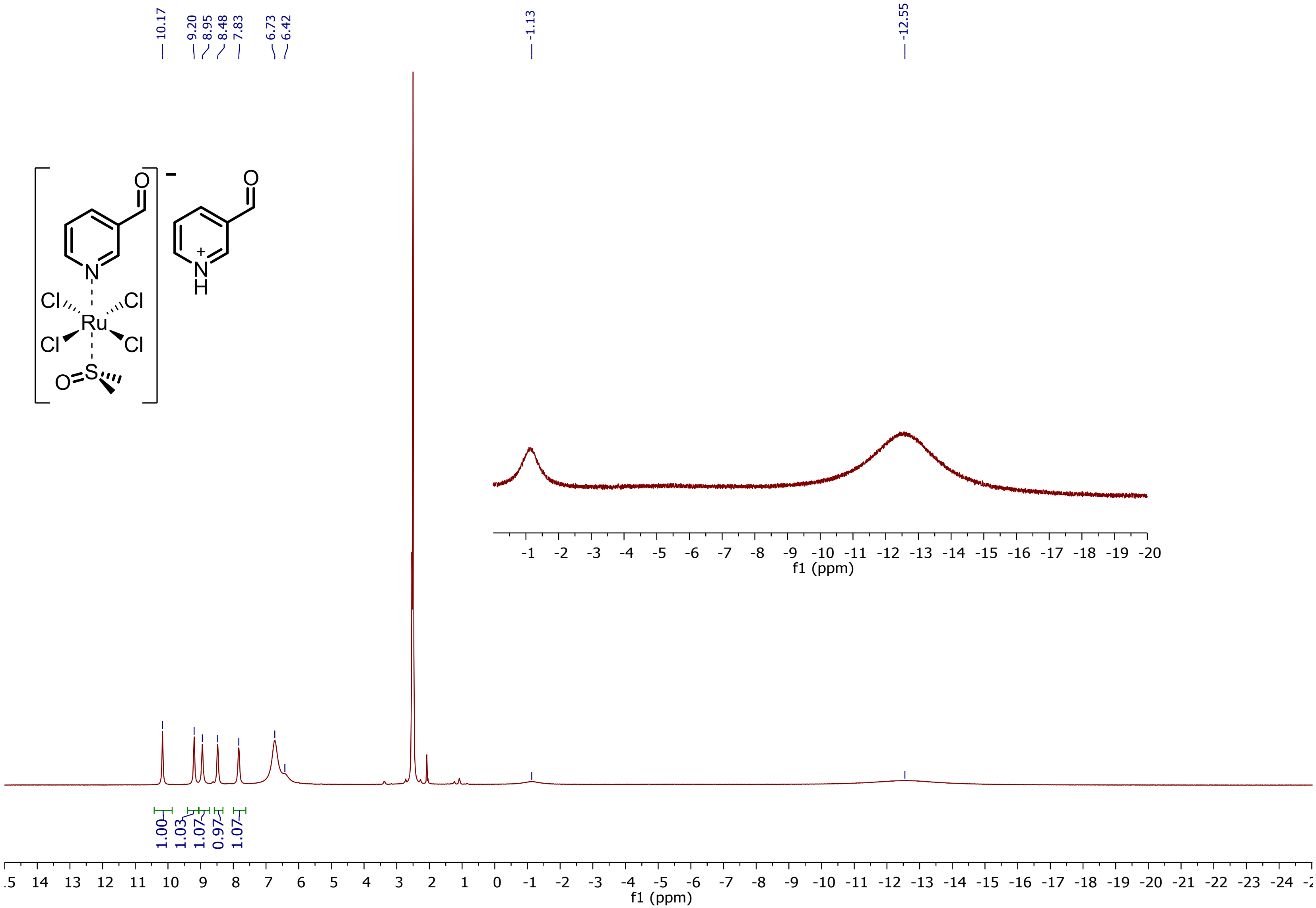
10.17
9.20
8.95
8.48
7.83
6.73
6.42
-1.13
-12.55
1.00
1.03
1.07
0.97
1.07
f1 (ppm)
f1 (ppm)

(NCS-PyH)[*trans*-RuCl$_4$(dmso-*S*)(NCS-Py)] **(Ru12)** (DMSO-d_6, 300 MHz)

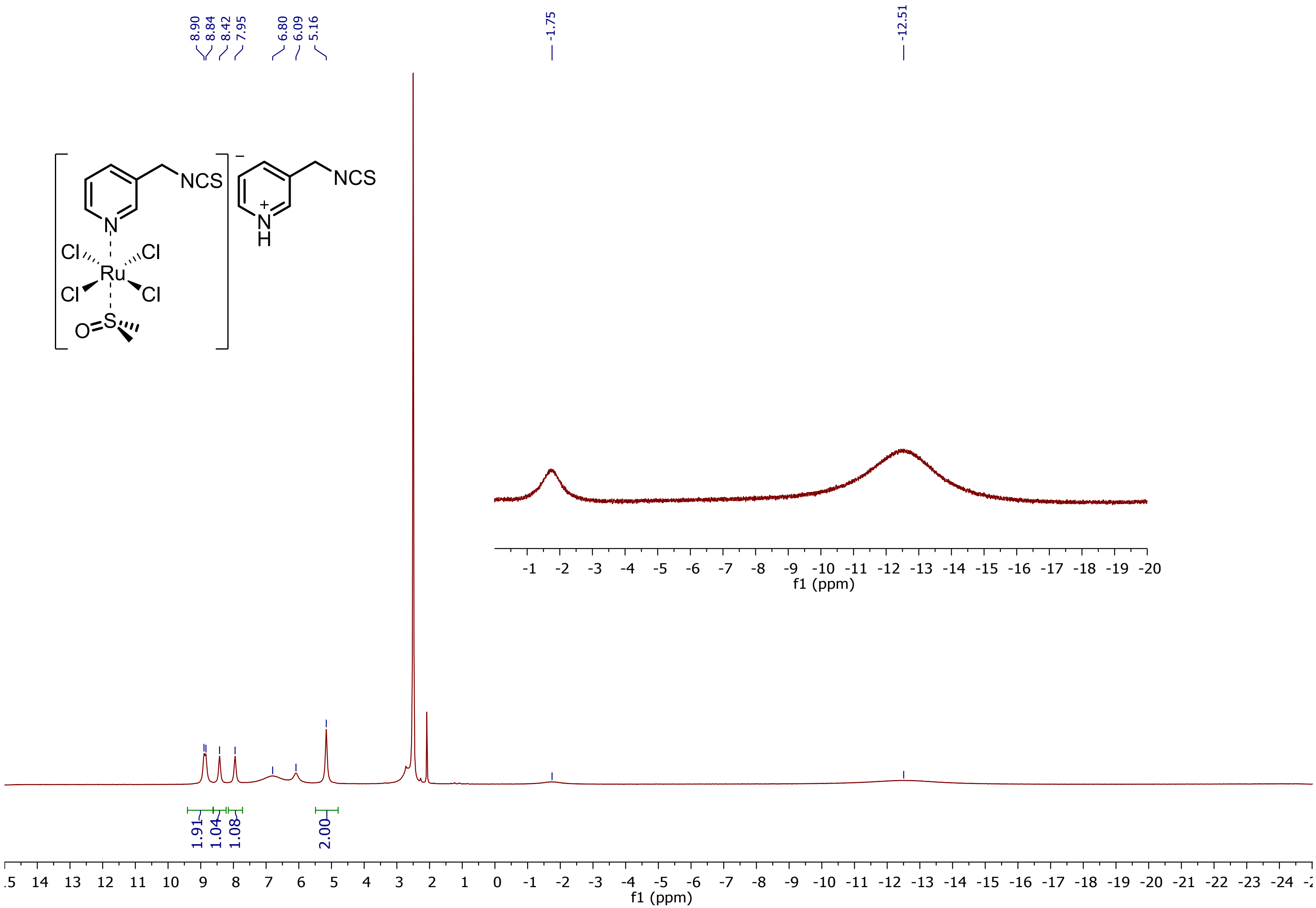

LRMS and HRMS of complexes (ESI$^-$).

Na[*trans*-Ru(BrPy)Cl_4(dmso-*S*)] (**Ru1**)

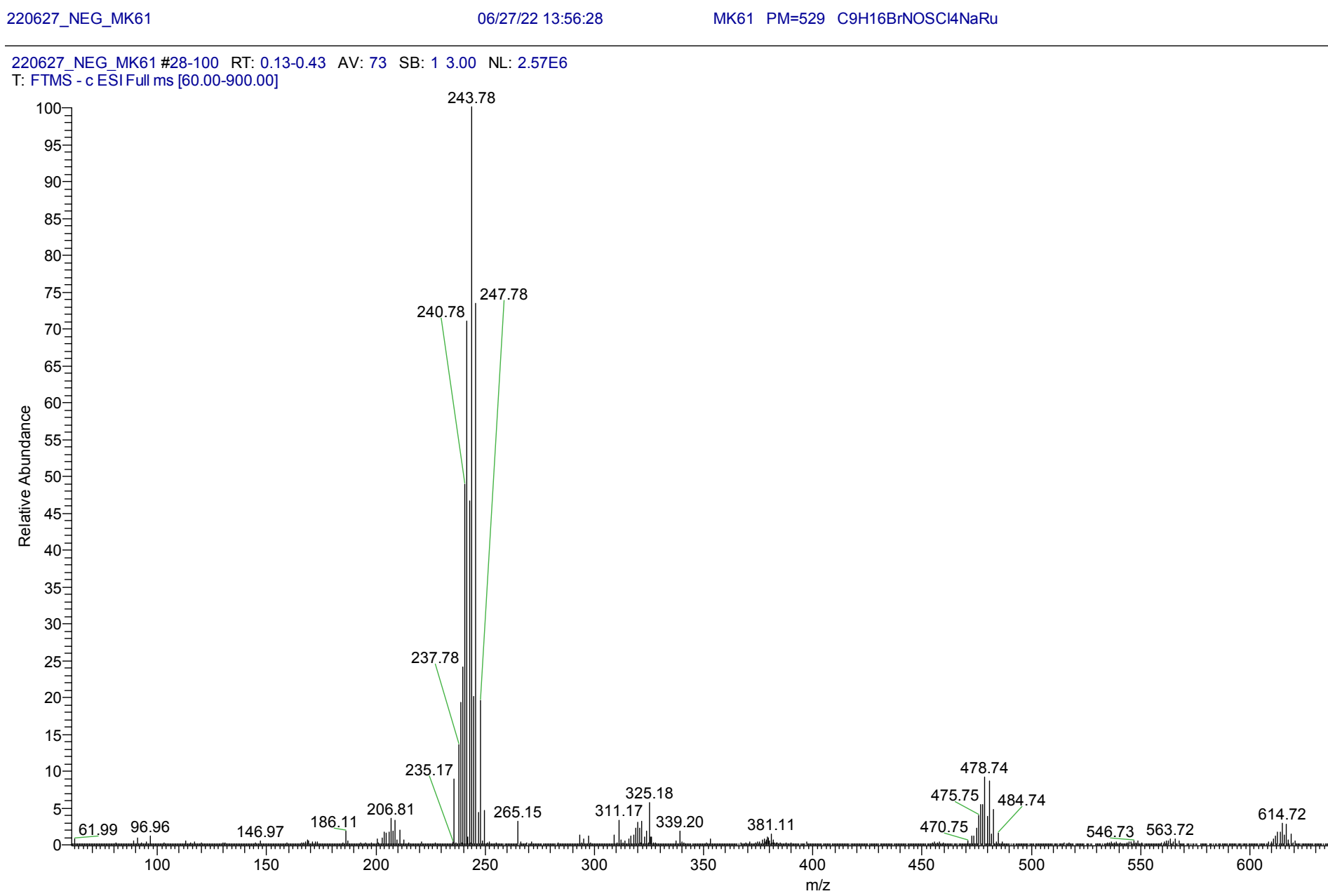

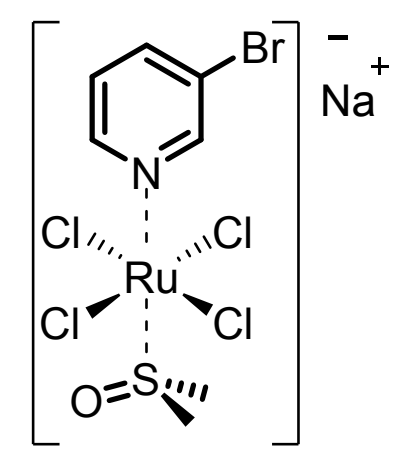

Chemical Formula:
$C_7H_{10}BrCl_4NORuS^-$
Molecular Weight:
478,9975

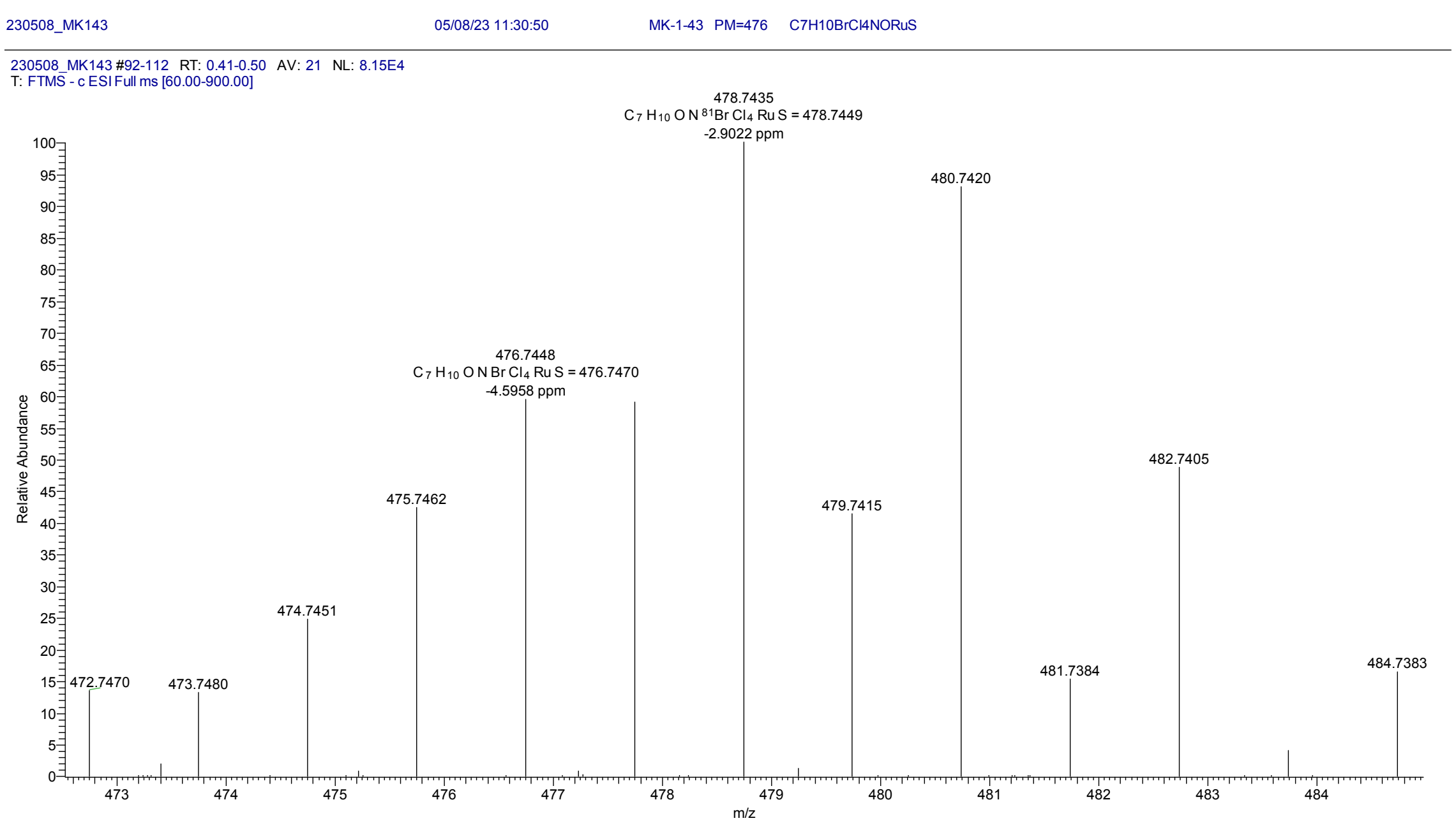

Na[*trans*-Ru(CHO-Py)Cl_4(dmso-*S*)] (**Ru2**)

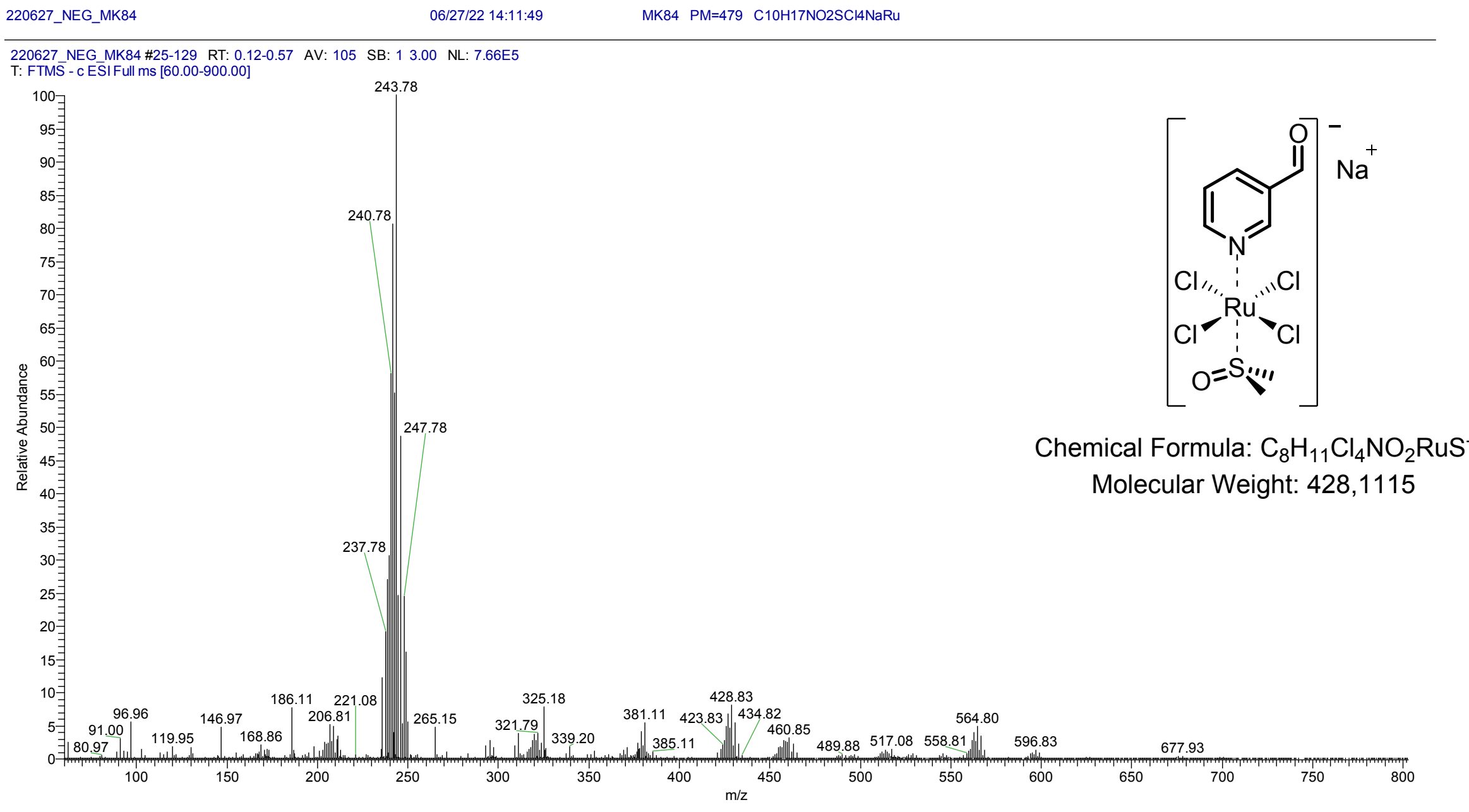

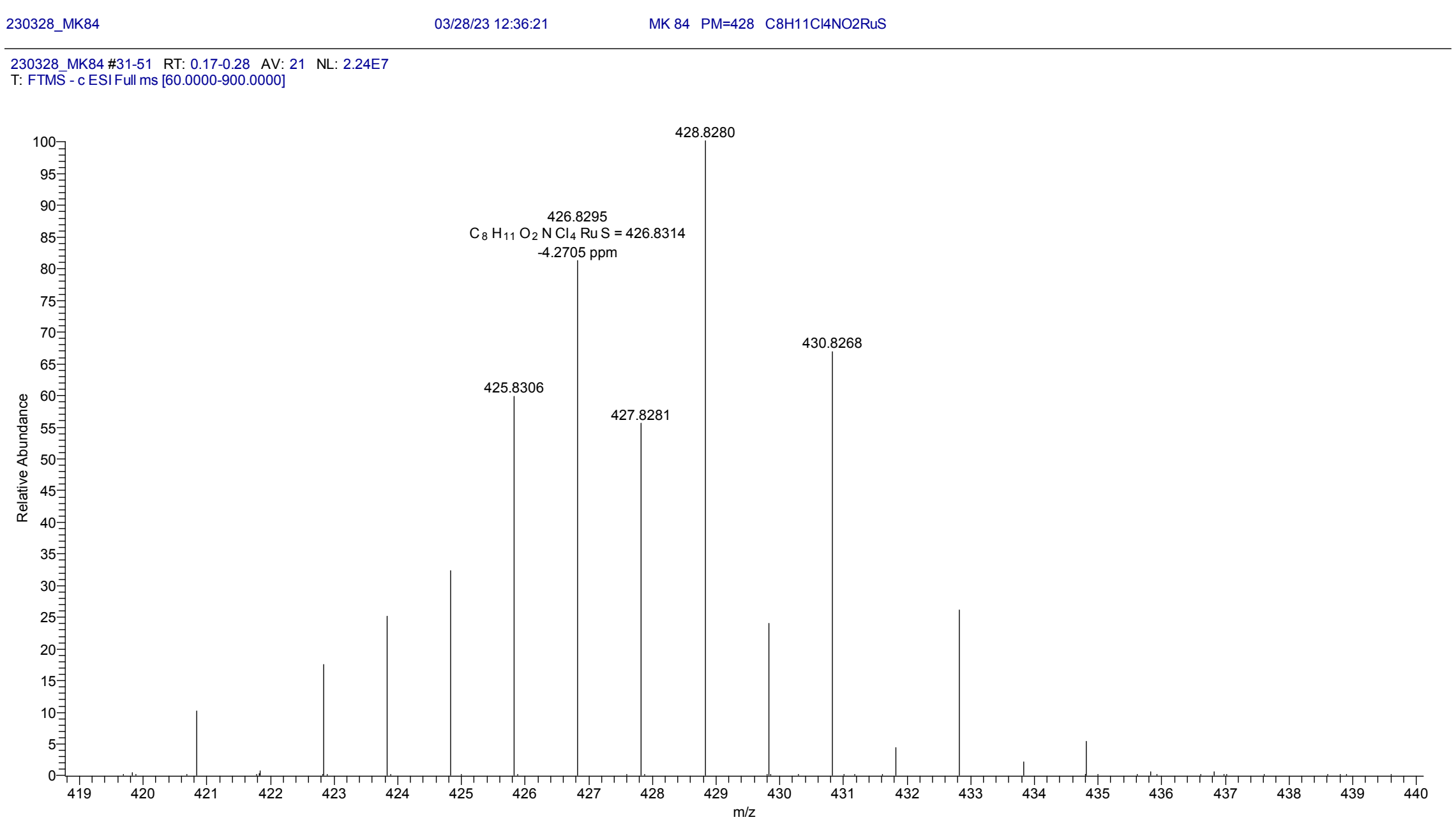

Na[*trans*-$RuCl_4$(dmso-*S*)(OH-Py)] (**Ru3**)

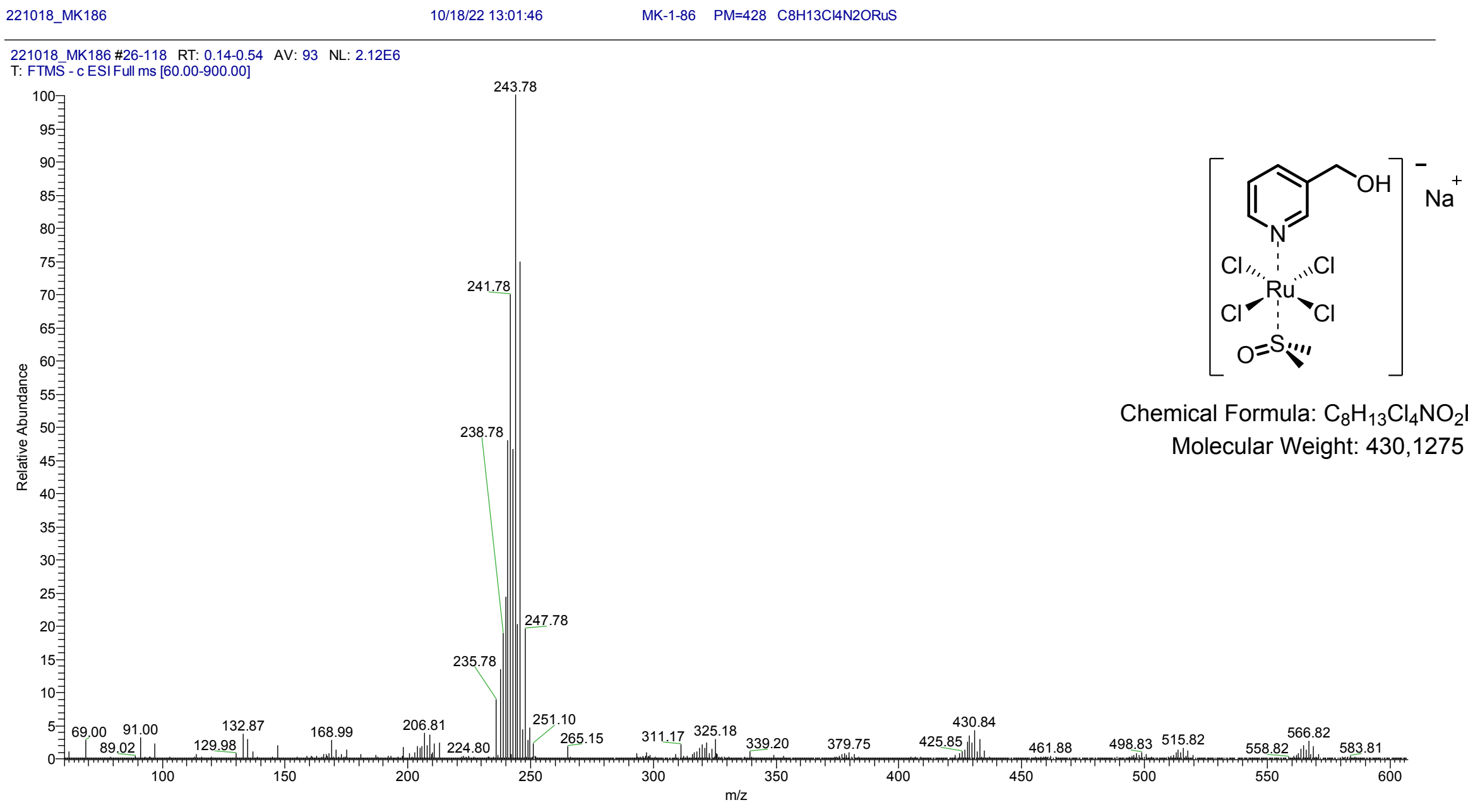

Chemical Formula: $C_8H_{13}Cl_4NO_2RuS^-$
Molecular Weight: 430,1275

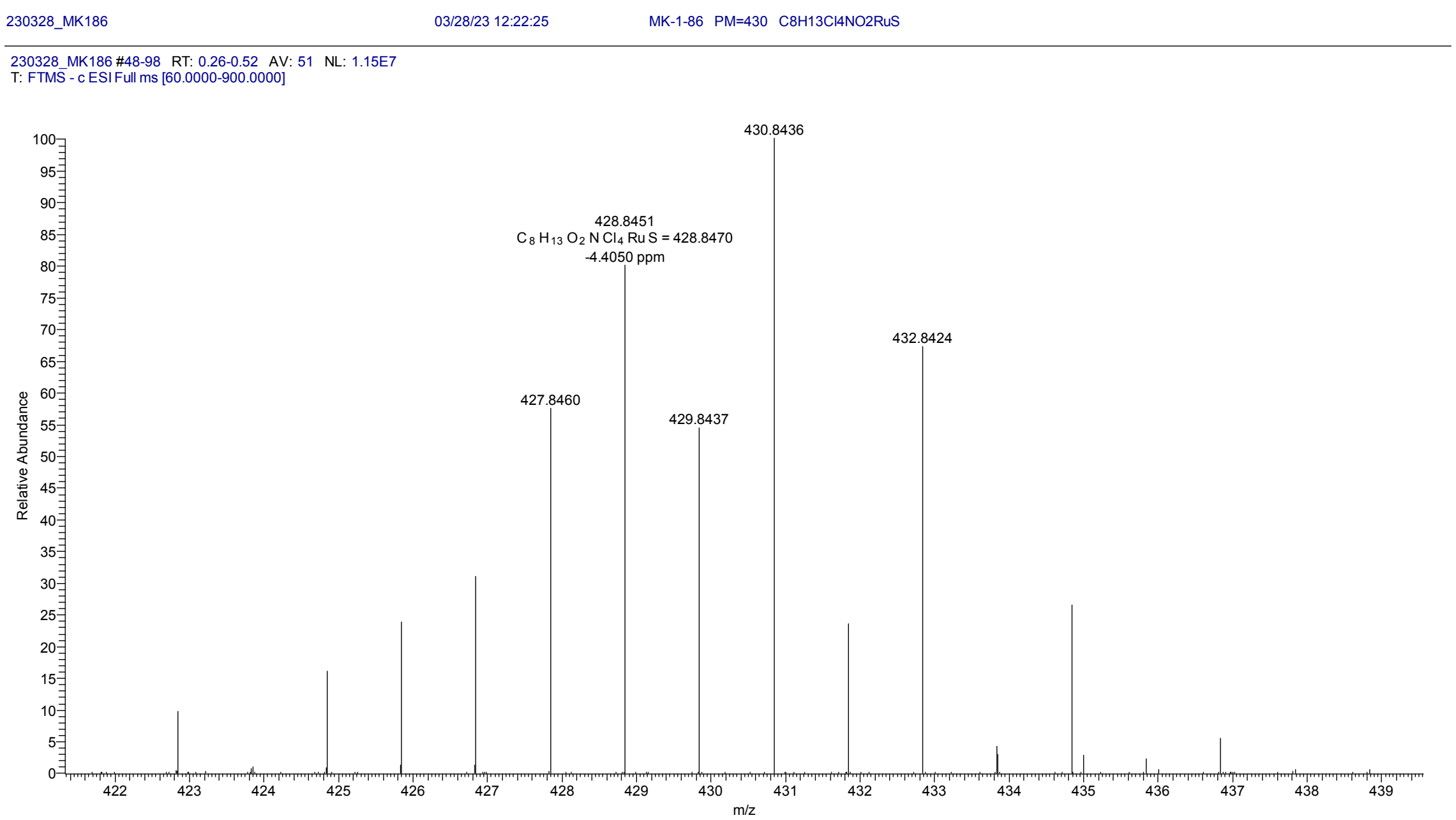

Na[*trans*-$RuCl_4$(dmso-*S*)(3-NBOC-Py)] (**Ru4**)

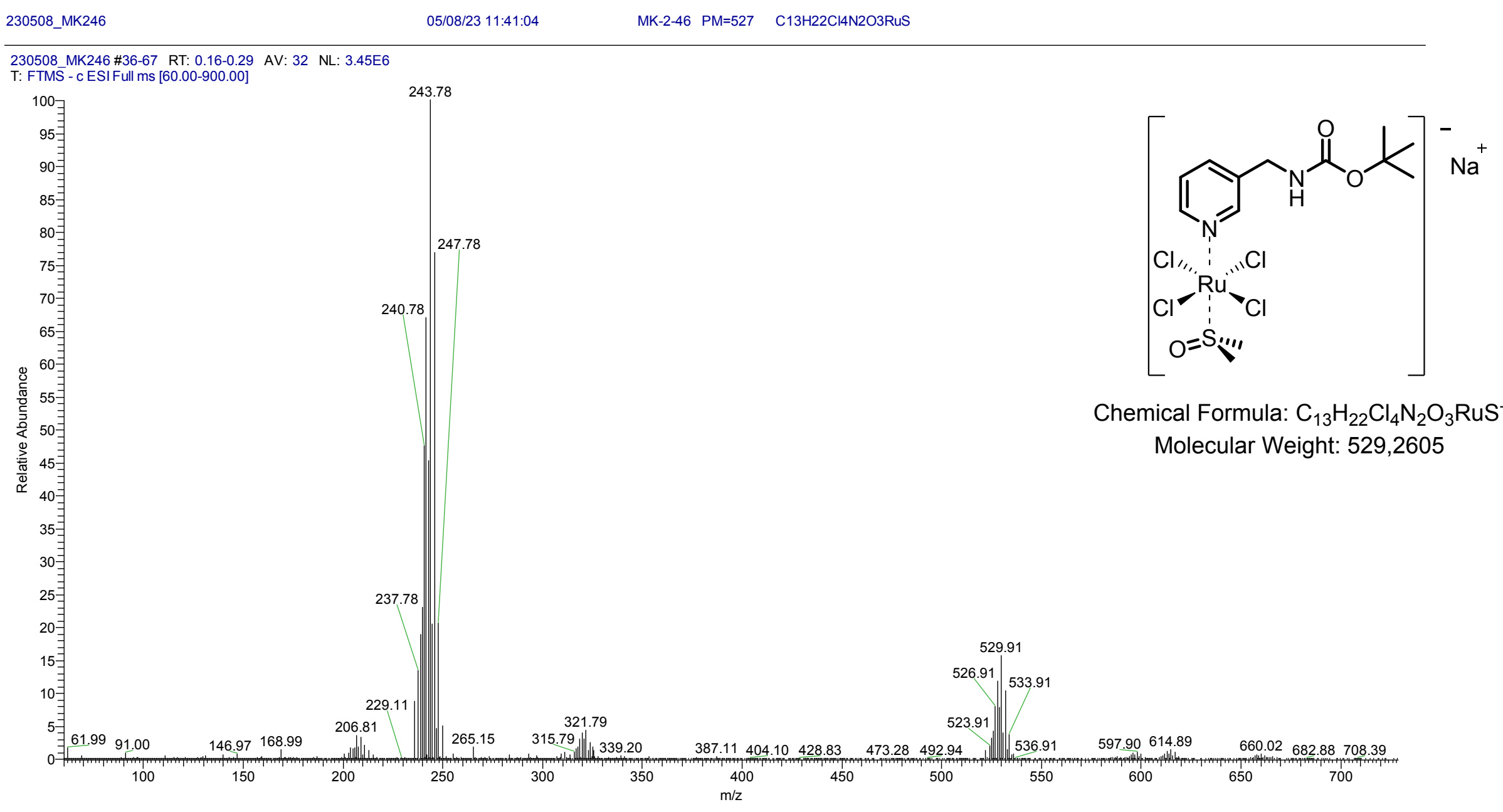

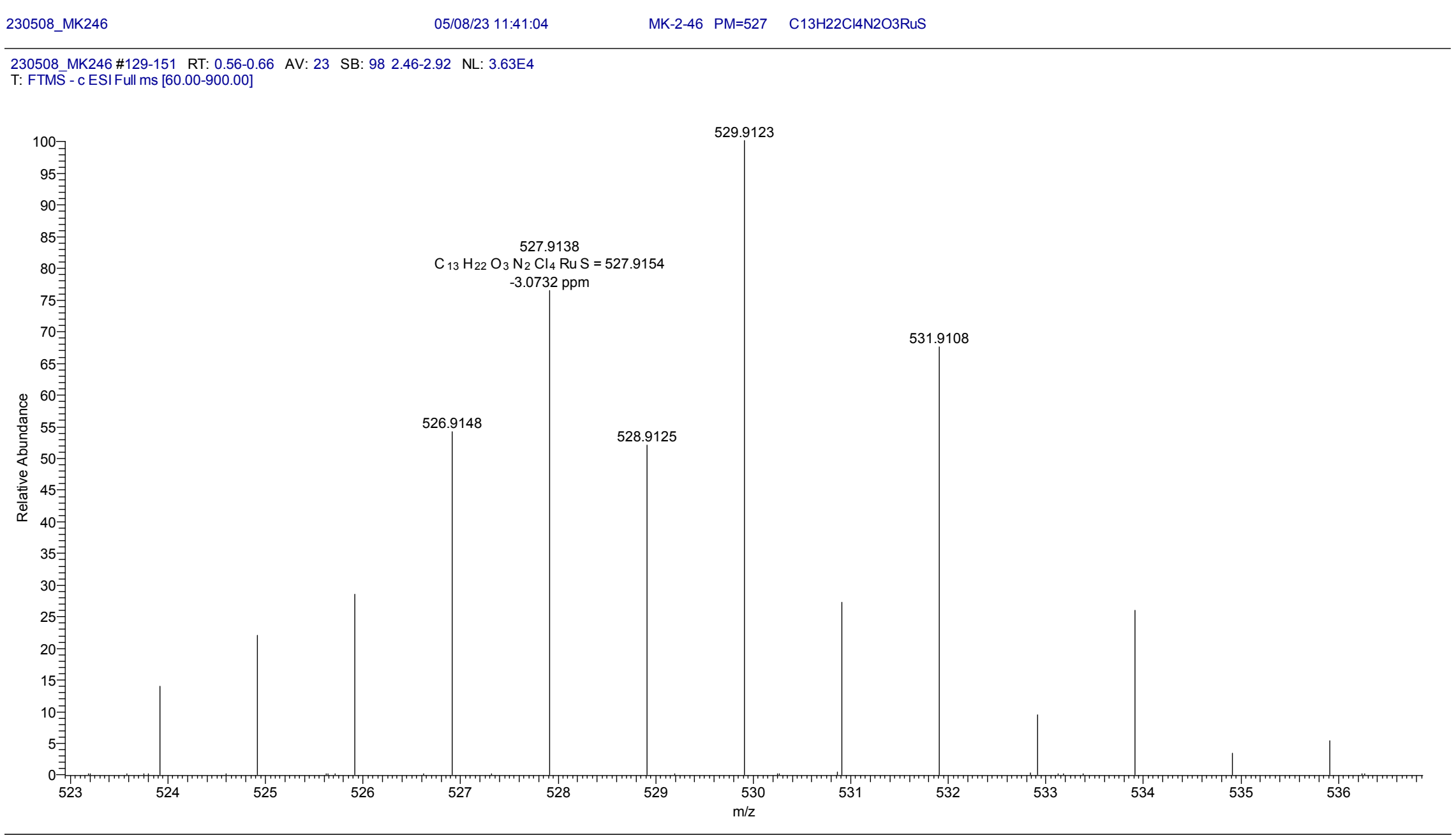

[*trans*-$RuCl_4$(dmso-*S*)(3-NH3Py)] (**Ru5**)

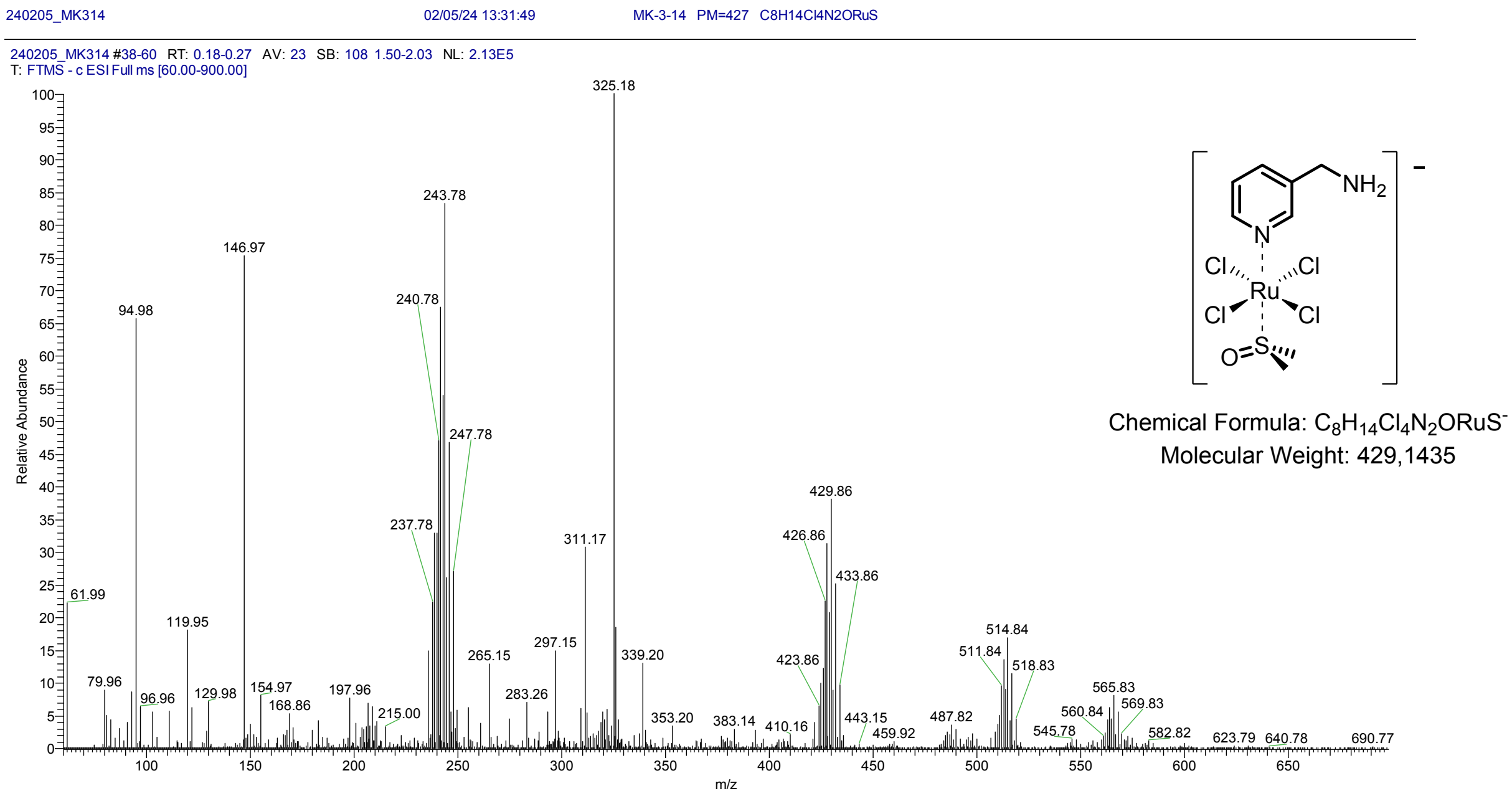

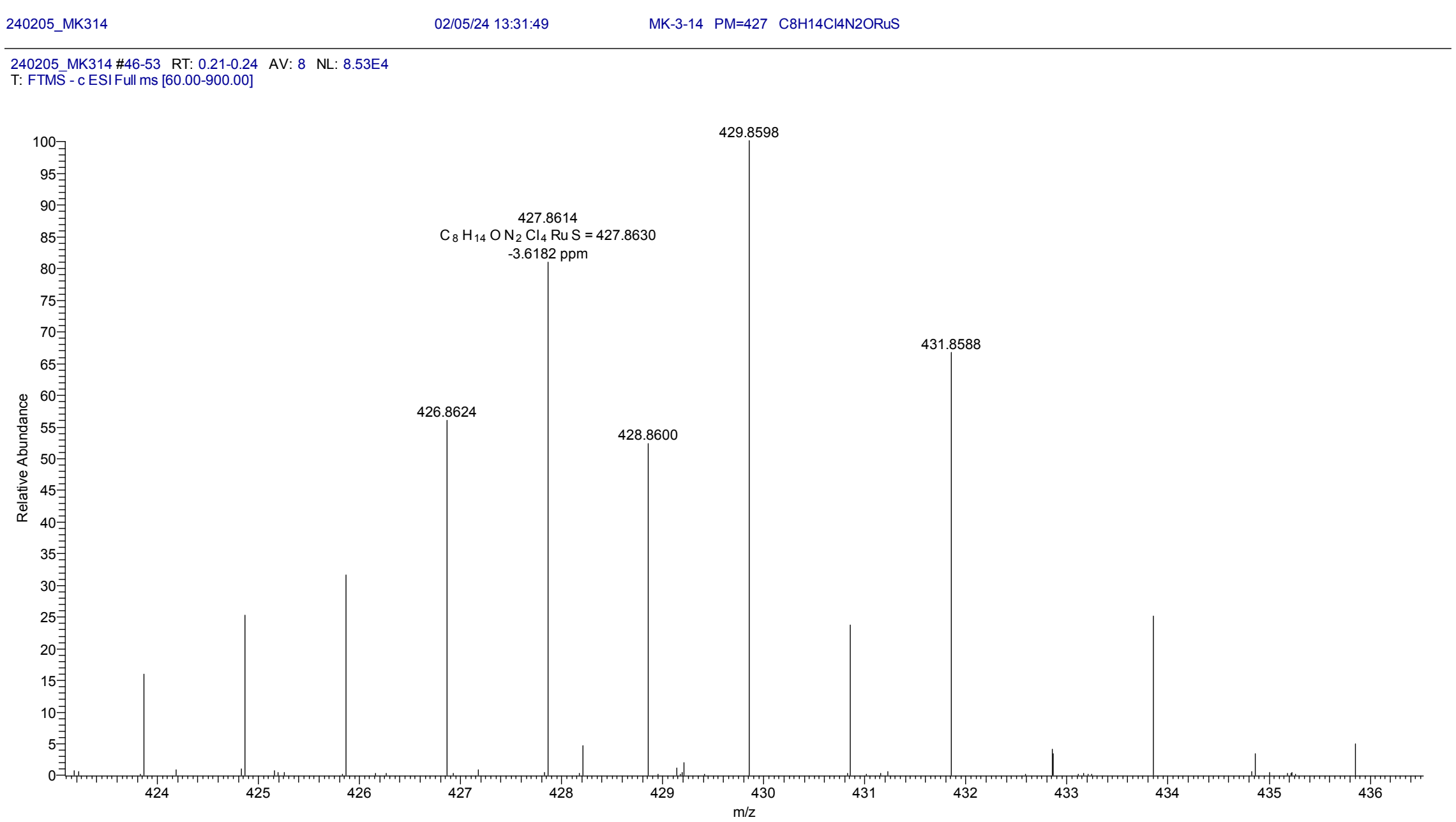

Na[*trans*-$RuCl_4$(dmso-*S*)(NCS-Py)] (**Ru6**)

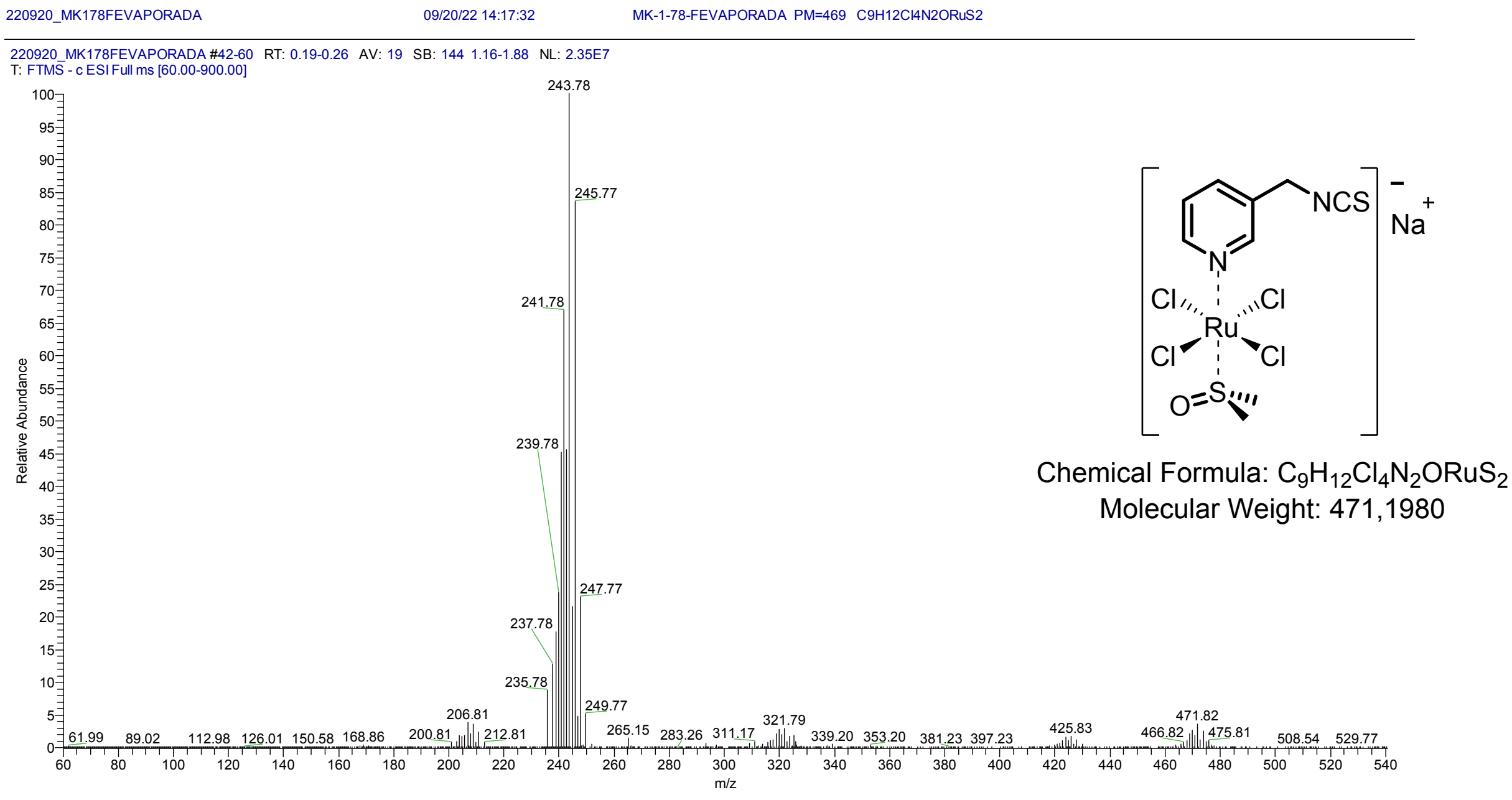

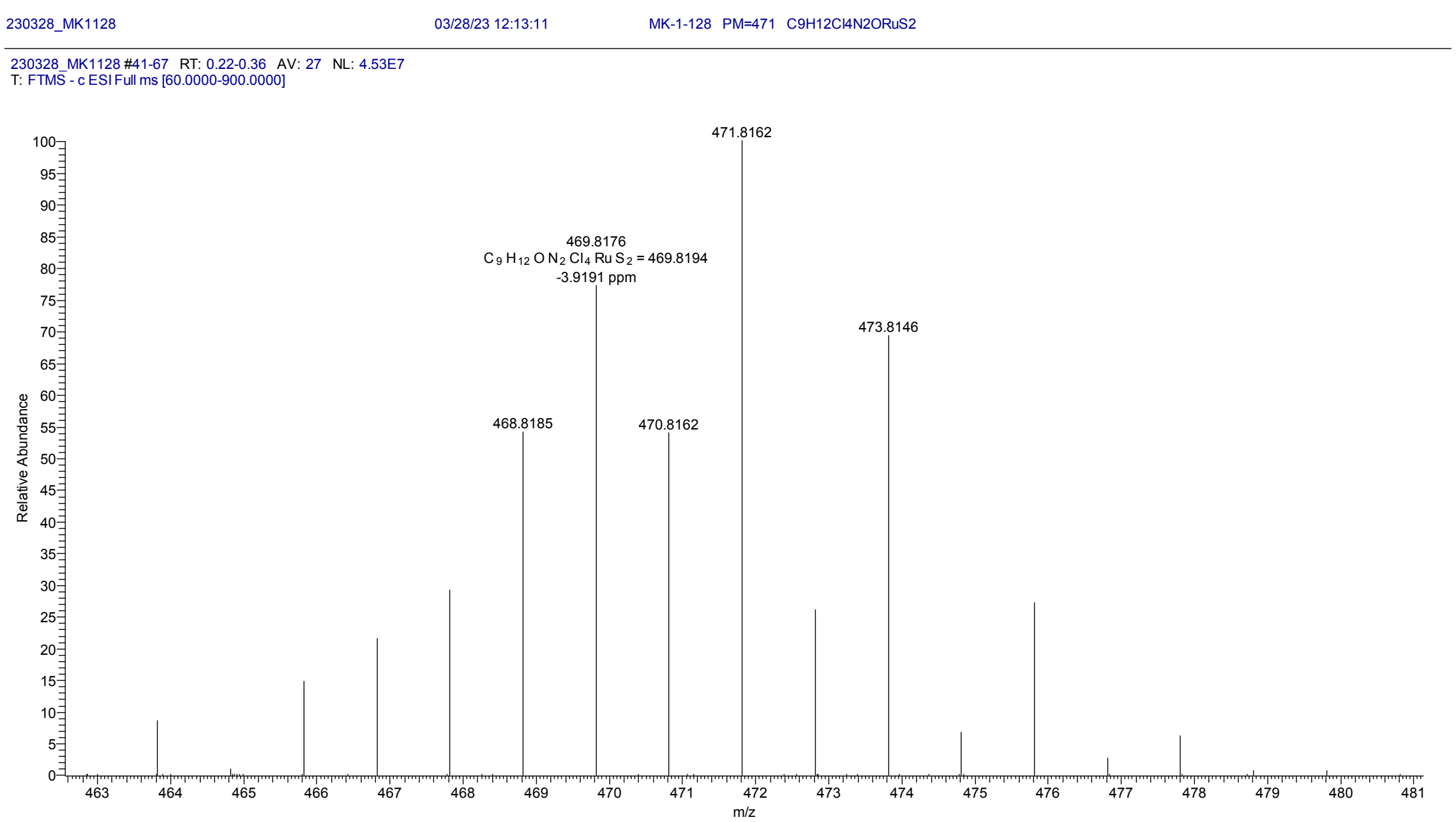

Na[*trans*-$RuCl_4$(CO_2Et)(dmso-*S*)] (**Ru7**)

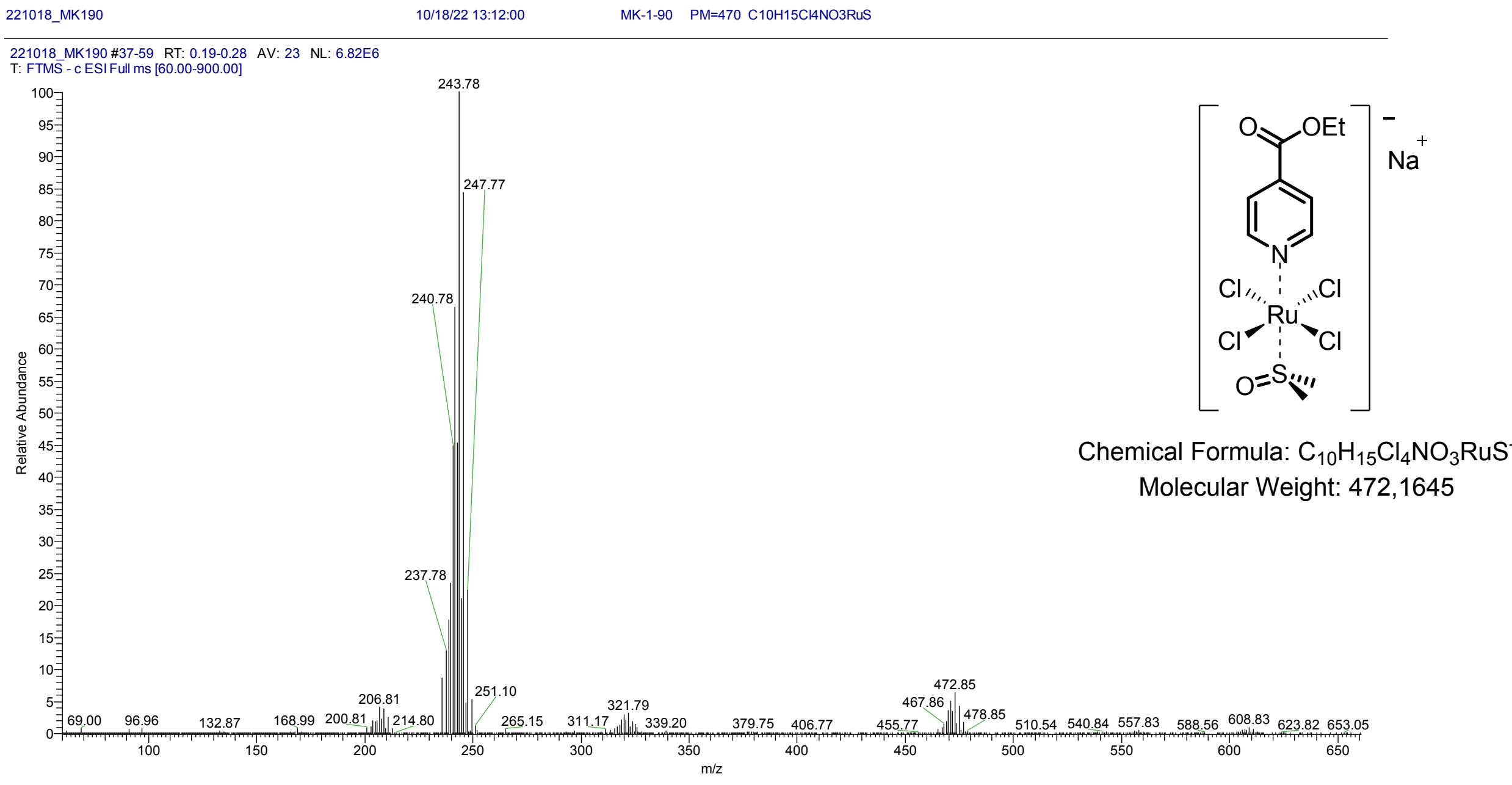

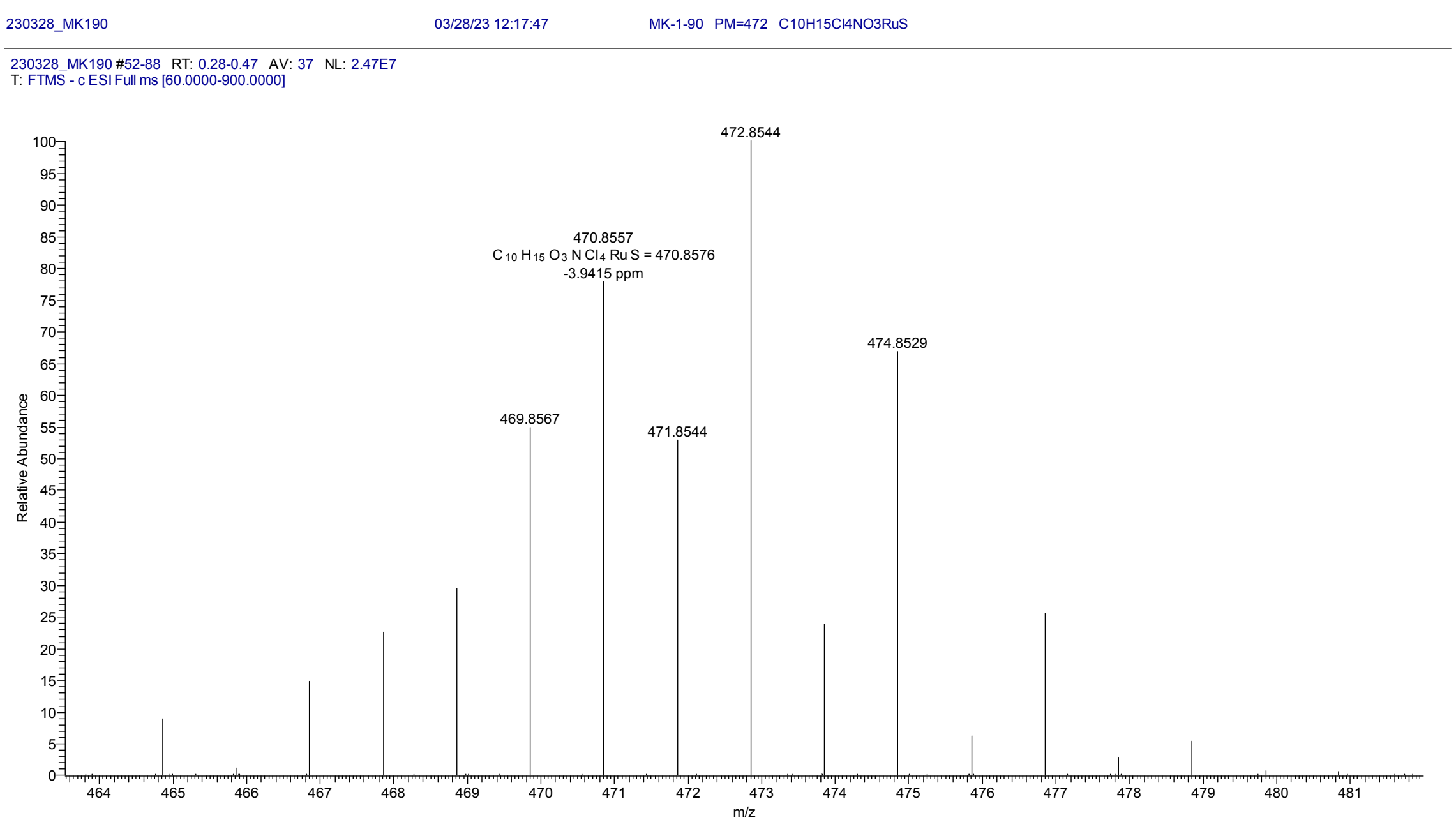

Na[*trans*-RuCl$_4$(dmso-*S*)(N$_3$-Py)] (**Ru8**)

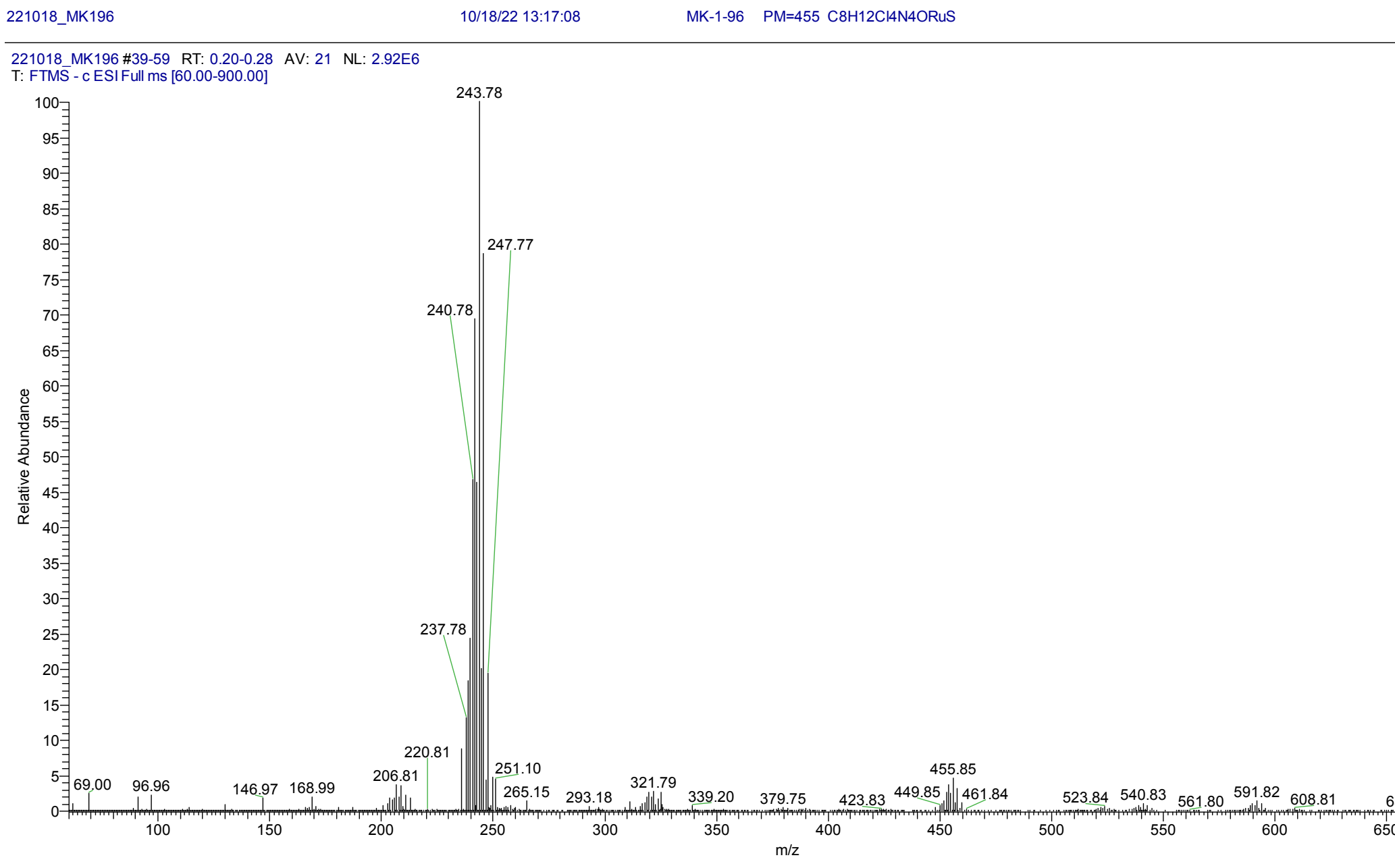

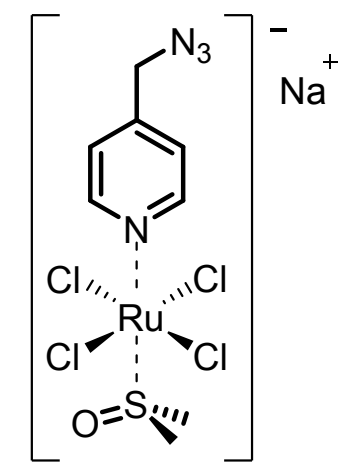

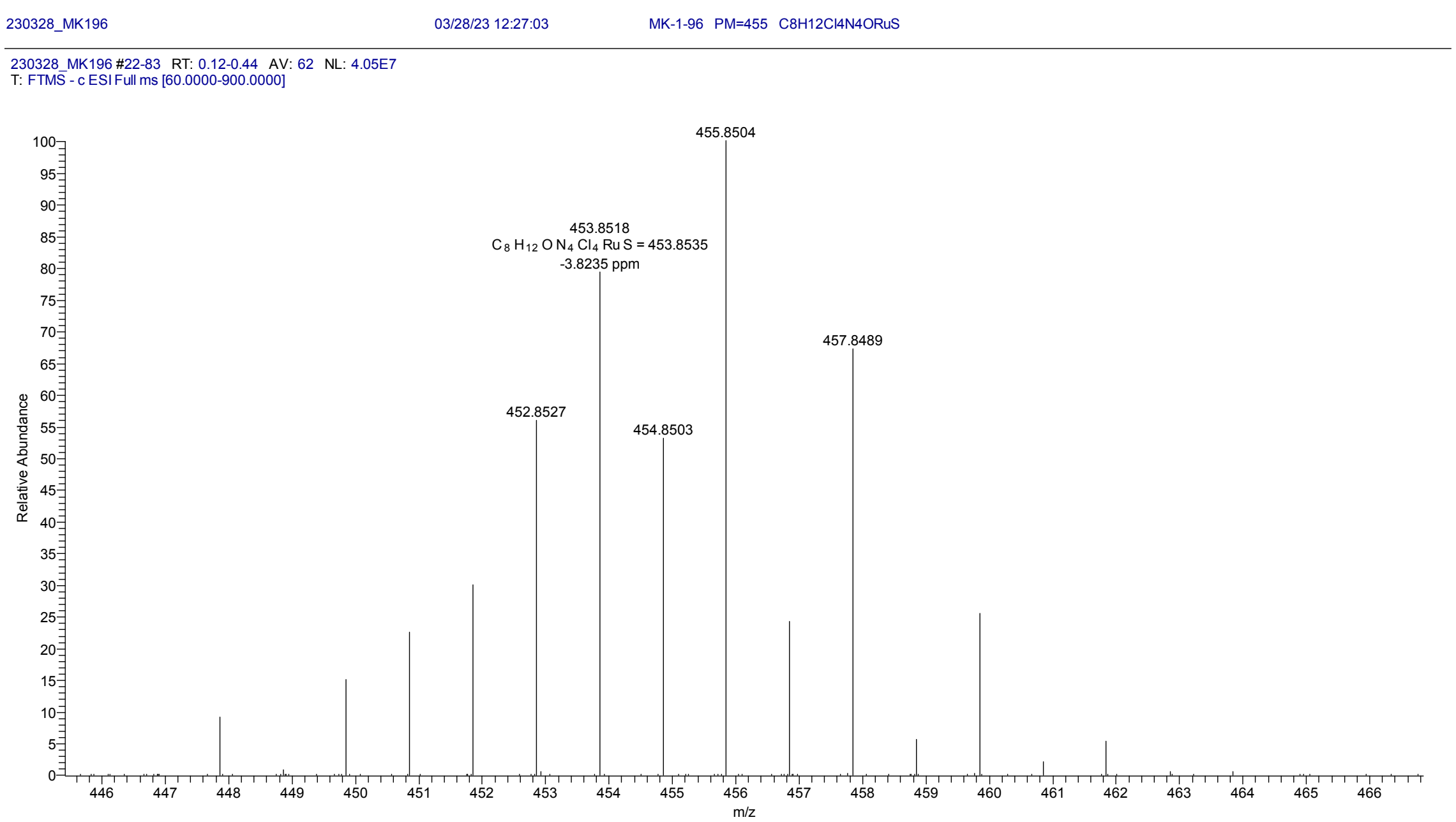

Na[*trans*-RuCl$_4$(dmso-*S*)(4-NBOC-Py)] (**Ru9**)

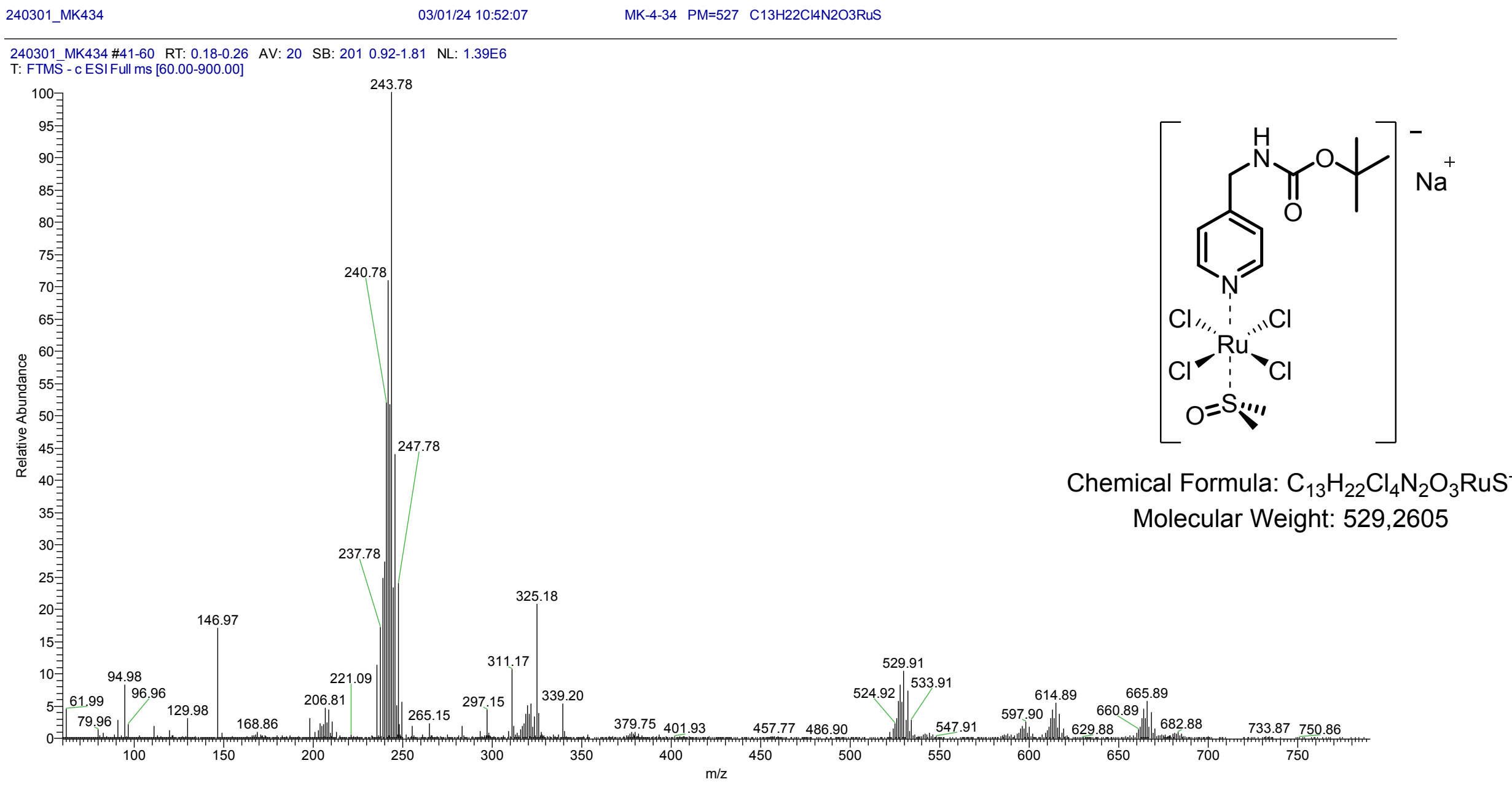

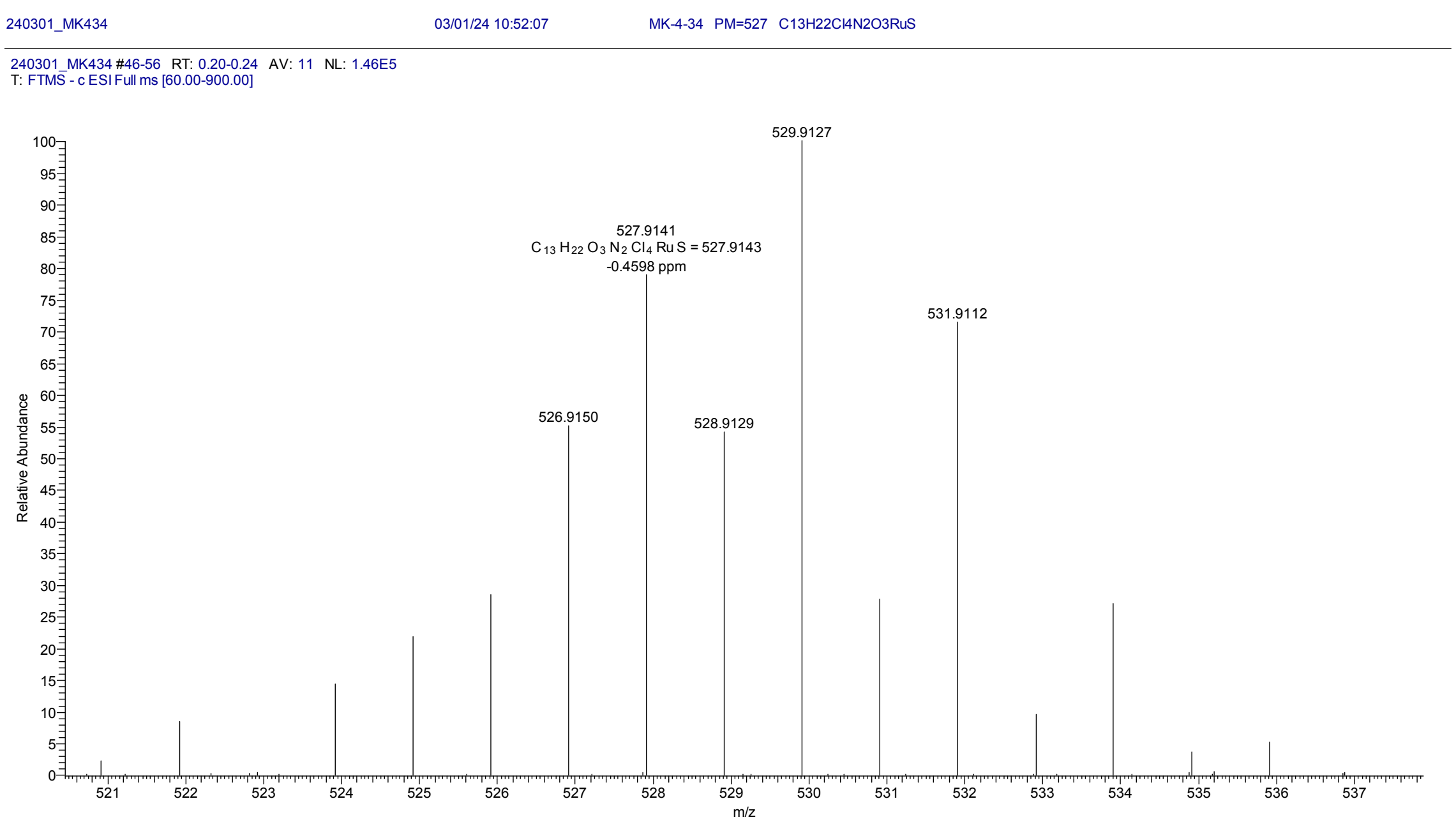

[*trans*-$RuCl_4$(dmso-*S*)(4-NH_3Py)] (**Ru10**)

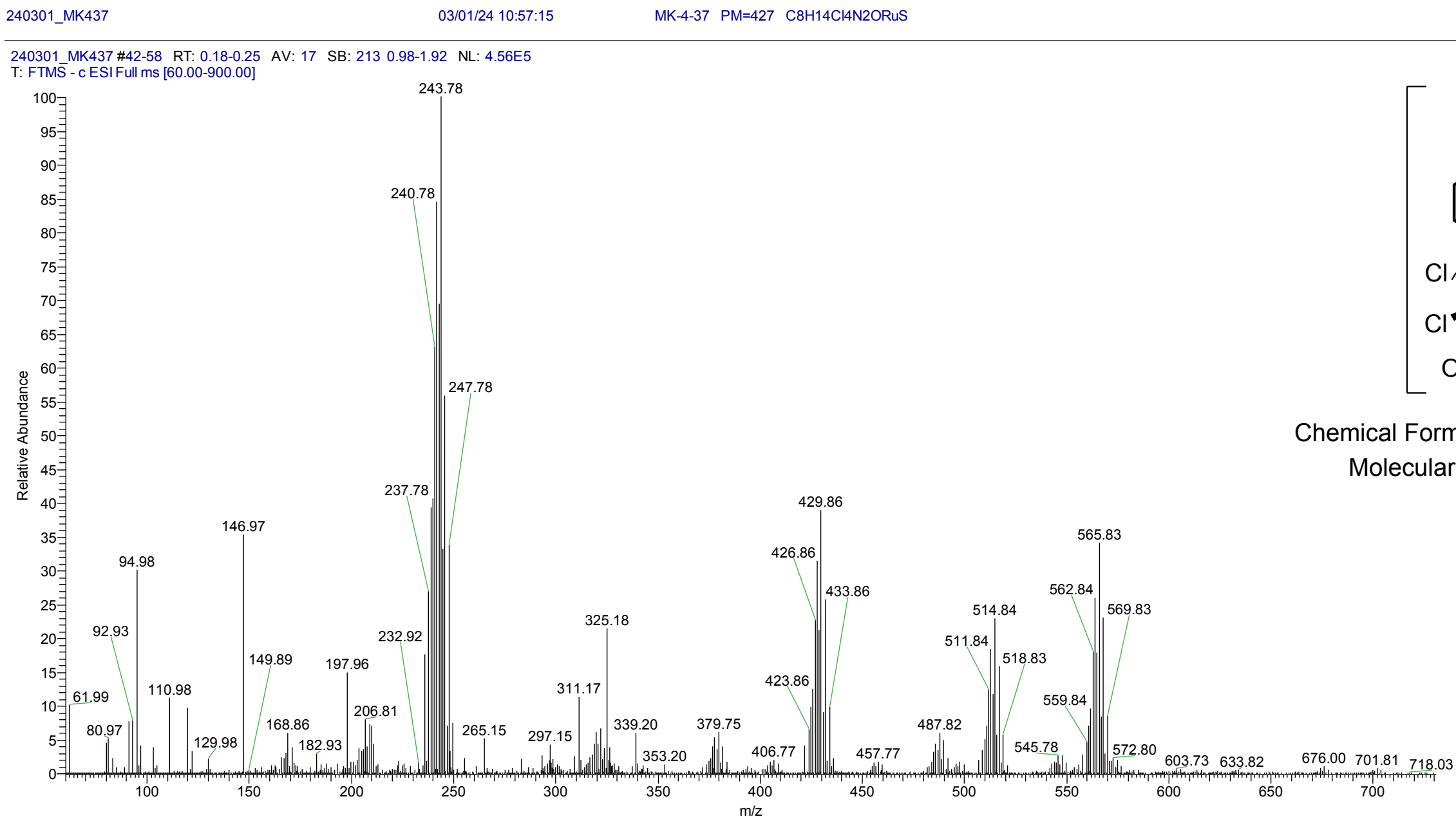

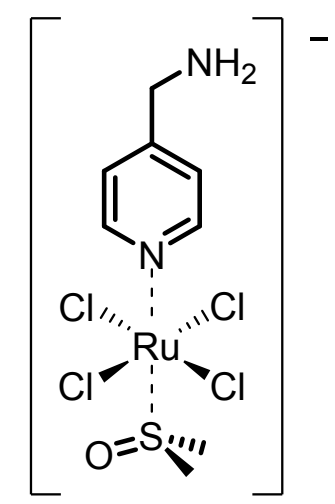

Chemical Formula: $C_8H_{14}Cl_4N_2ORuS^-$
Molecular Weight: 429,1435

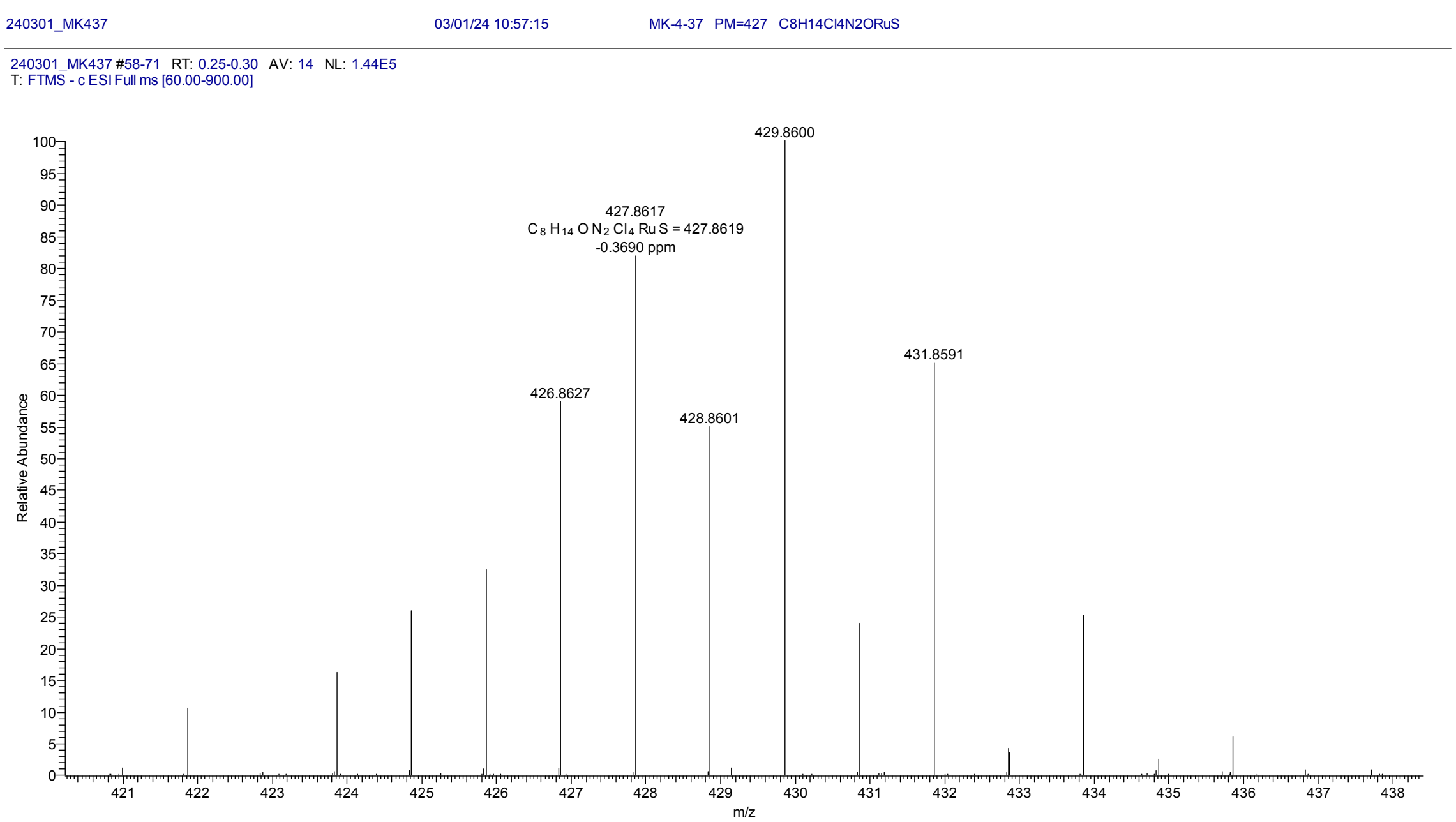

(CHO-PyH)[*trans*-Ru(CHO-Py)Cl_4 (dmso-*S*)] (**Ru11**)

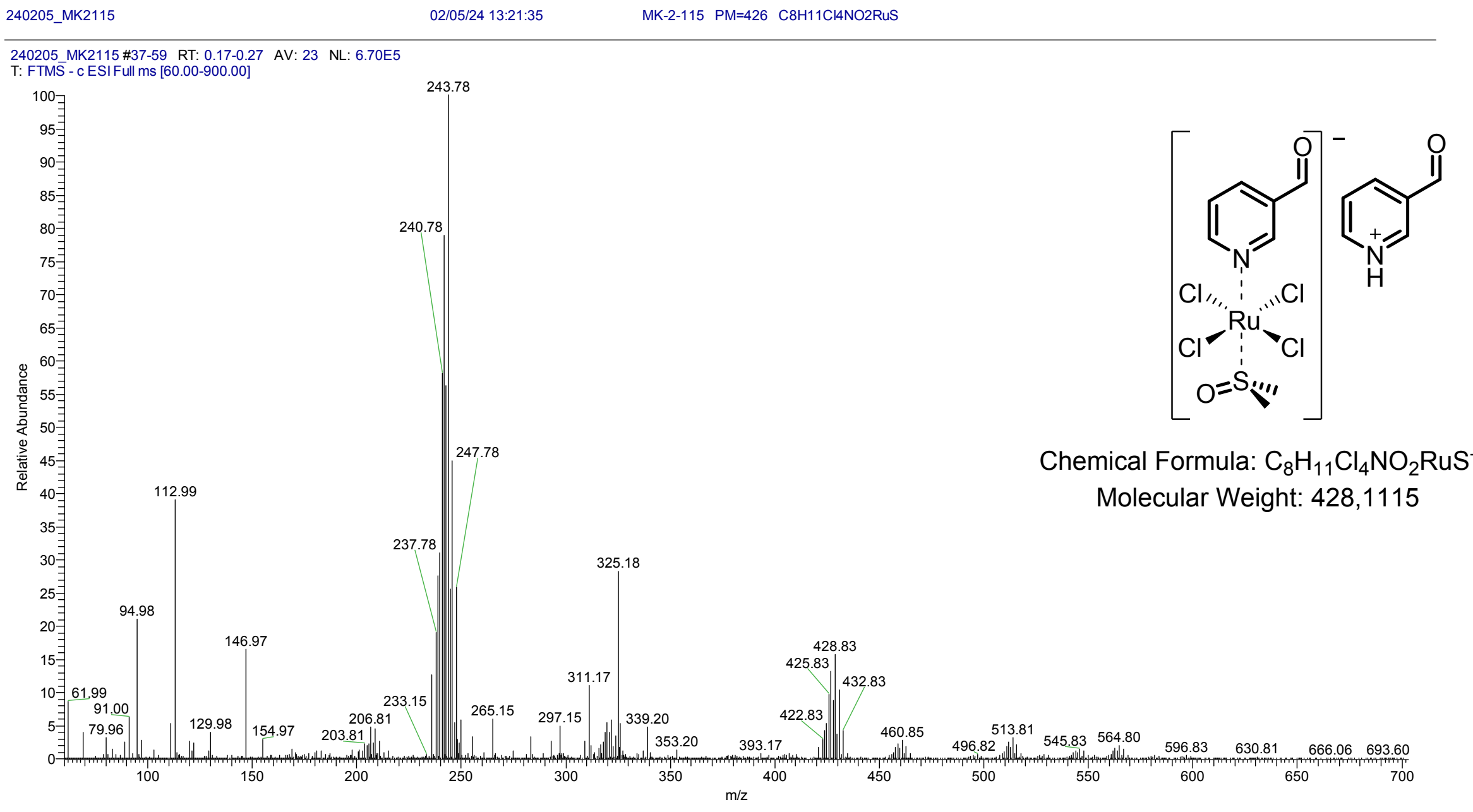

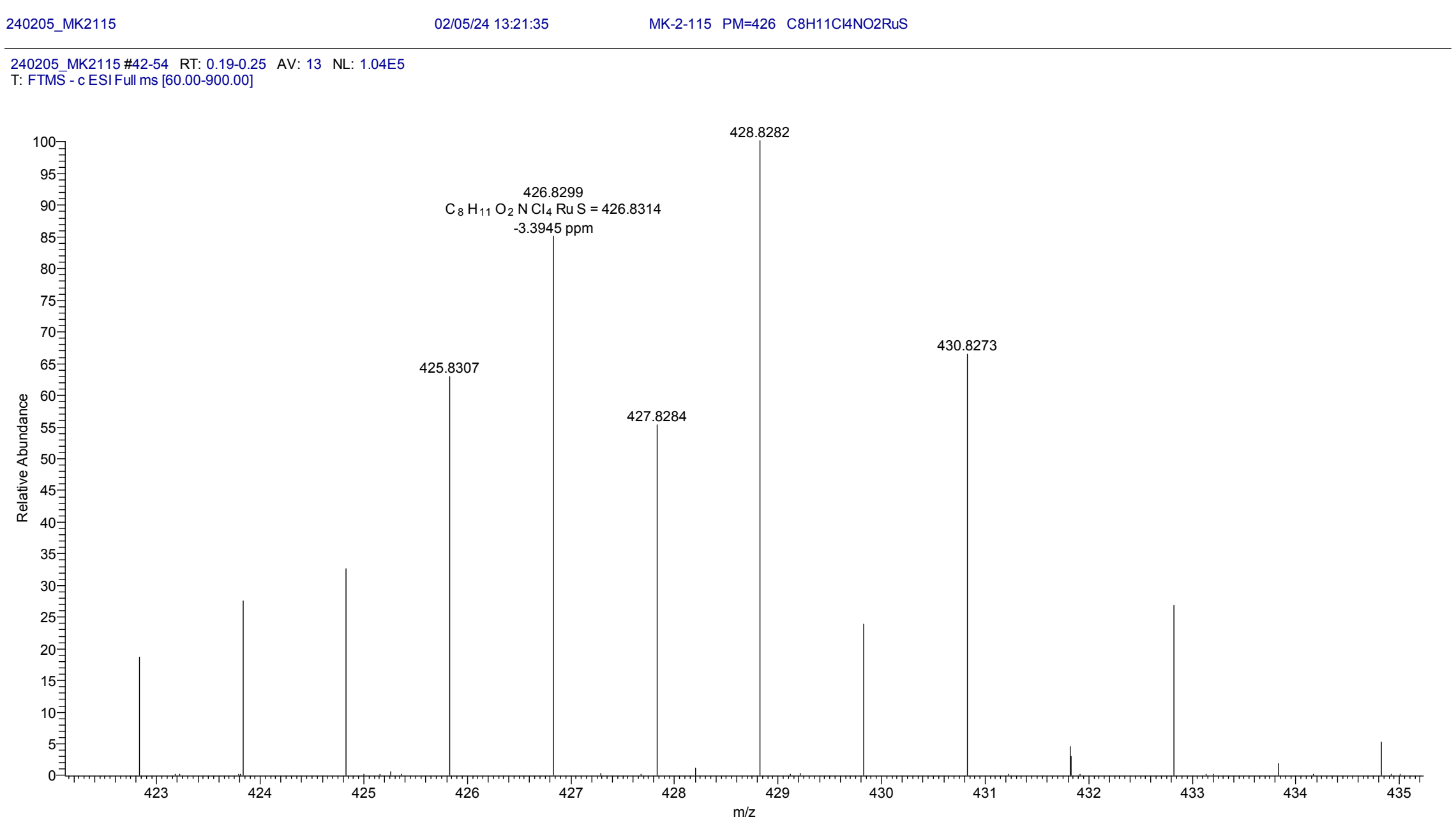

(NCS-PyH)[*trans*-RuCl$_4$(dmso-*S*)(NCS-Py)] (**Ru12**)

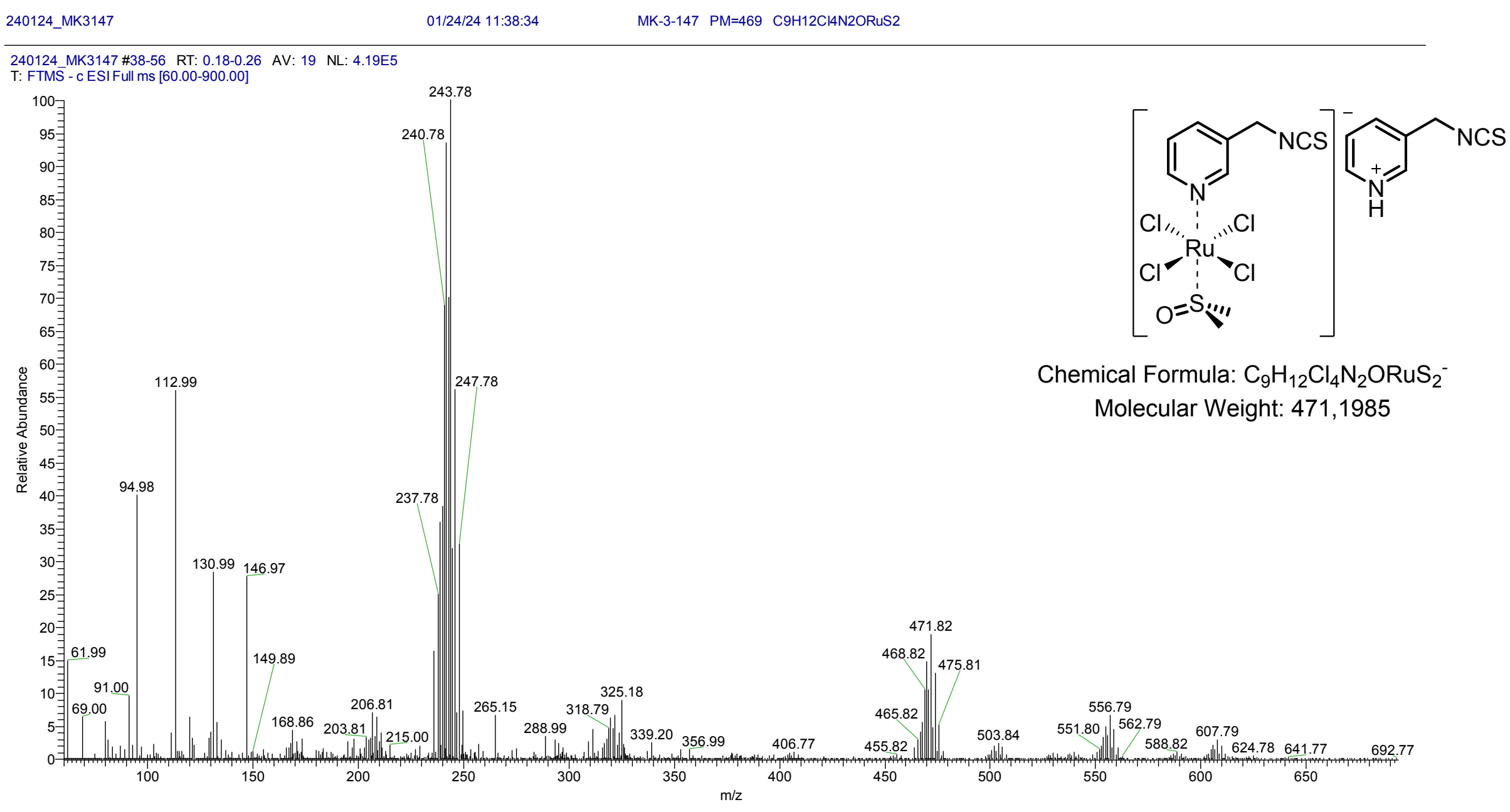

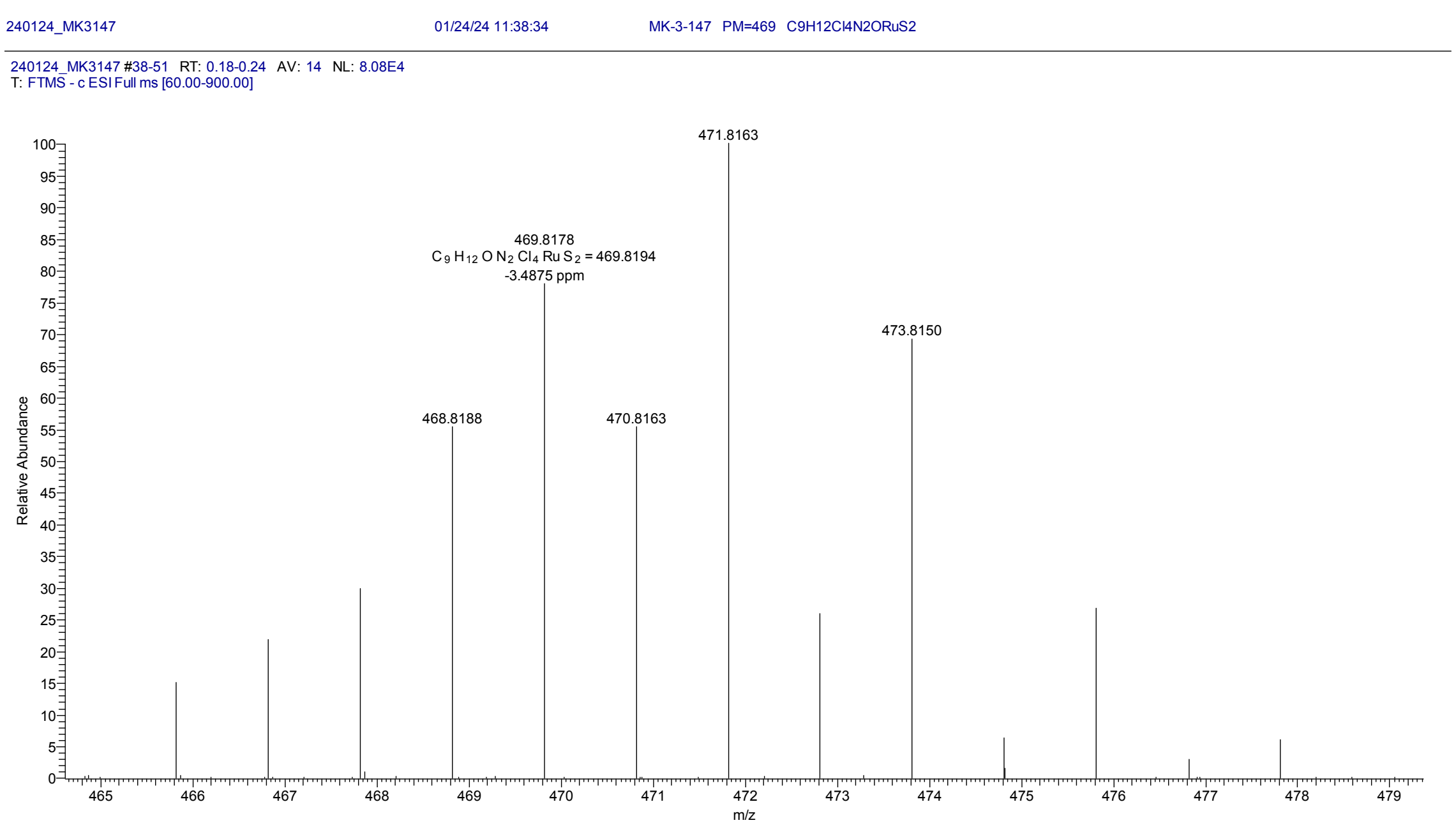

(ImH)[*trans*-$RuCl_4$(dmso-*S*) (Im)] (**NAMI-A**)

230209_MK216.raw 2/9/2023 1:36:56 PM MK-2-16 PM=387 C5H10Cl4N2ORuS

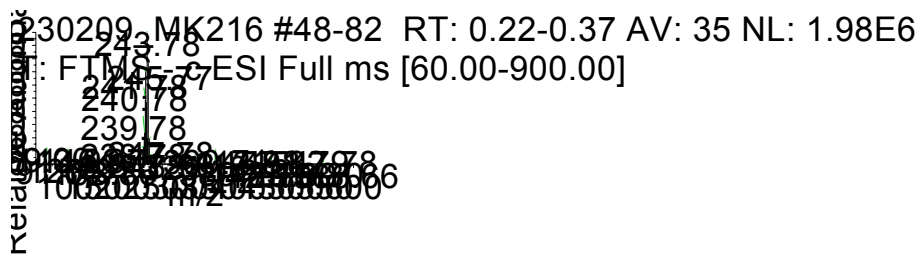

HPLC purity analysis of complexes.

Purity of the complexes **Ru1**-**Ru5** and **Ru7**-**Ru11** was analyzed on a Thermo Scientific Dionex UltiMate 3000 UHPLC-system using a Waters Acquity UPLC BEH C18 1.7 µm 3.0x50 mm column. Acetonitrile containing 0.1% (v/v) trifluoroacetic acid, and Milli-Q water containing 0.1% (v/v) trifluoroacetic acid were used as eluents. A gradient of 5-95% (v/v) of acetonitrile in water, at a flow rate of 0.6 ml/min, over 5 min was used. Absorption was monitored using a wavelength of 220 nm.

Chromatograms of **Ru6** and **Ru12** were recorded using HPLC Shimadzu LC-2030C Plus 3D RoHS-Prominence-I using a Phenomenex Luna C18 5 µm 250x4.6 mm column. Same eluents as before, with a gradient of 5-95% (v/v) of acetonitrile in water over 10 minutes, at a flow rate of 1.0 mL/min.

<u>Sample preparation.</u>

0.6 µmol of the complex were completely dissolved in 5 µL of DMSO and added into 200 µL of a 5% (v/v) mixture of acetonitrile in water, with 0.1% (v/v) of trifluoroacetic acid as additive.

<u>Chromatogram interpretation.</u>

The purity determination of the complexes through HPLC is not straightforward and several things had to be considered. Some of the complexes prepared, especially the ones containing protonated amines (**Ru5** and **Ru10**) and the NAMI-A type (**Ru11** and **Ru12**), do not present high solubility in the starting conditions for the chromatography (5 % v/v acetonitrile in H_2O). This

problem was avoided by previous complete dissolution in 5 μL of DMSO, which causes the absorbance observed in the injection peak (r.t. around 0.5 min, figure S26).

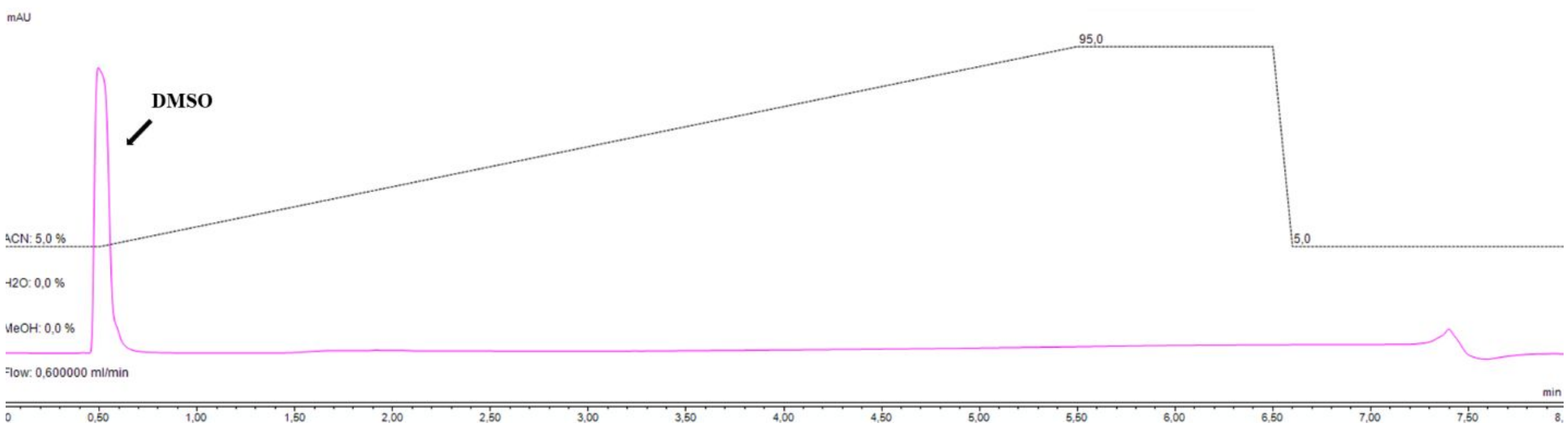

Figure S26. Blank chromatogram obtained from the addition of 5 μL of DMSO to the 200 μL 5 % v/v acetonitrile in H_2O mixture with 0.1% v/v of trifluoroacetic acid.

Besides DMSO, the complex peak always appeared accompanied by other smaller peaks, which at first glance could seem impurities, but after closer inspection of the chromatograms of samples incubated at 25 °C at different times, this hypothesis was ruled out. The area of some of these peaks was observed to decrease (Orange arrows, Figure S27), while the main peak corresponding to the product increased (Black arrow, Figure S27). Which indicated that the smaller peaks also corresponded to the complex but needed some time to converge into the main peak. This could be explained by counterion exchange in solution or dissolution of the polymeric solid structure already observed with the LRMS or the SC-XRD. And, as expected from the behavior in aqueous solution of the NAMI-type complexes, when the incubation time increased new small peaks started to appear, corresponding to the hydrolysis (Green arrows, Figure S27).

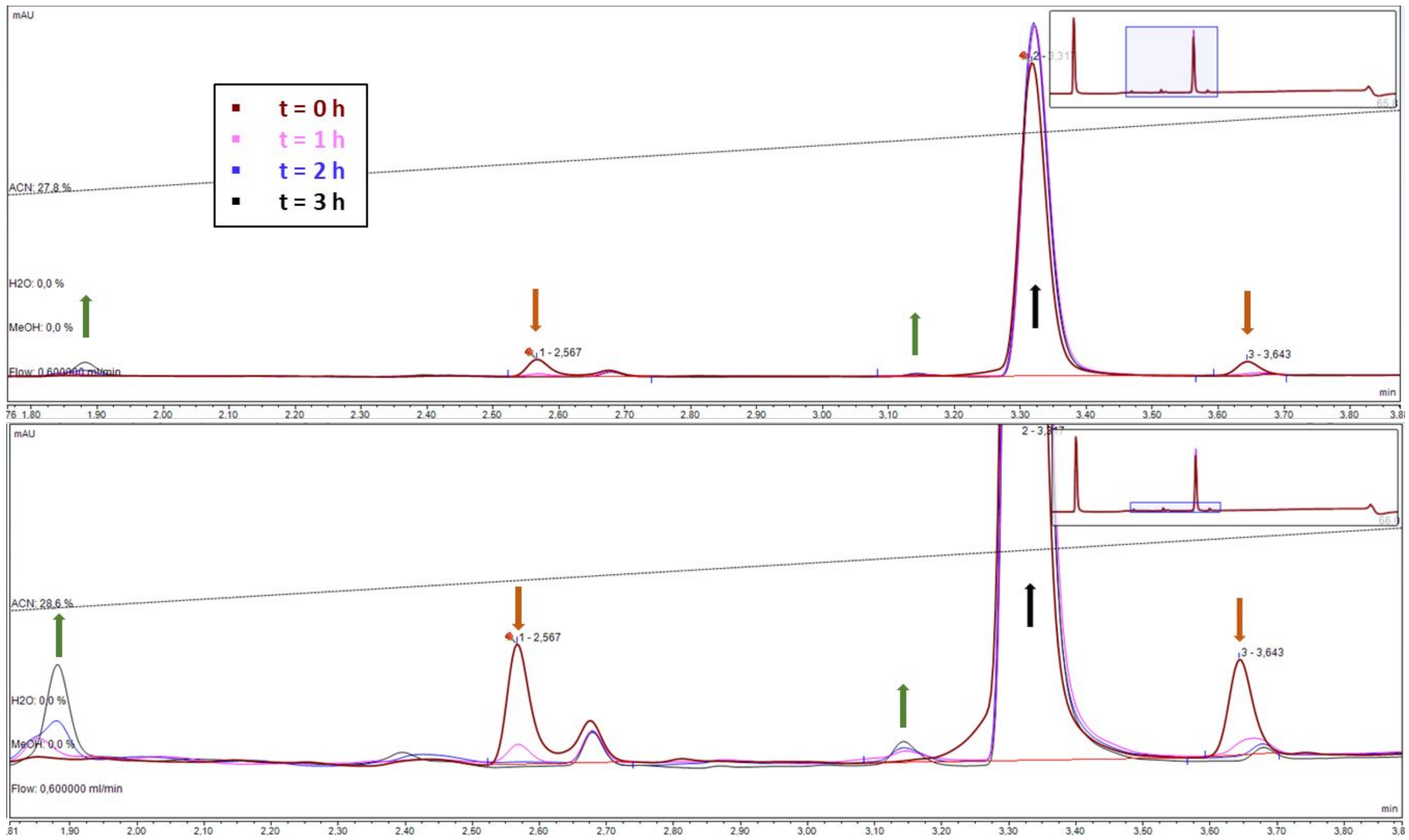

Figure S27. Magnifications of the stacked chromatograms of **Ru9** at different incubation times at 25 ˚C. In garnet red is represented the chromatogram obtained after solution. Pink was used for one hour incubation, blue for two hours and black for three. The complex peak is indicated with the black arrow. Orange arrows represent the peaks with decreasing area after incubation, and with green arrows the peaks with increasing area.

This led us to use the chromatograms obtained at t = 0, and discard all the peaks with areas that changed with time, to estimate the purity of the compounds prepared. Relative area % was used for the estimation, to simplify calculations.

Due to software limitations, chromatograms of complexes **Ru6** and **Ru12** could not be stacked, difficulting the analysis of the peaks that disappear with time. Therefore, the purity analysis was carried out by comparison of the freshly dissolved sample and one incubated for 3 hours.

In the **Ru12** chromatogram, there is a peak around 6.5 minutes that is not considered for the purity analysis. This peak corresponds to the protonated pyridine. The counterion in **Ru11** is not visible in the chromatogram because it has the same elution time as the DMSO peak. This behavior has already been observed in the group in other NAMI-A analogue complexes.

UHPLC purity.

Ru1

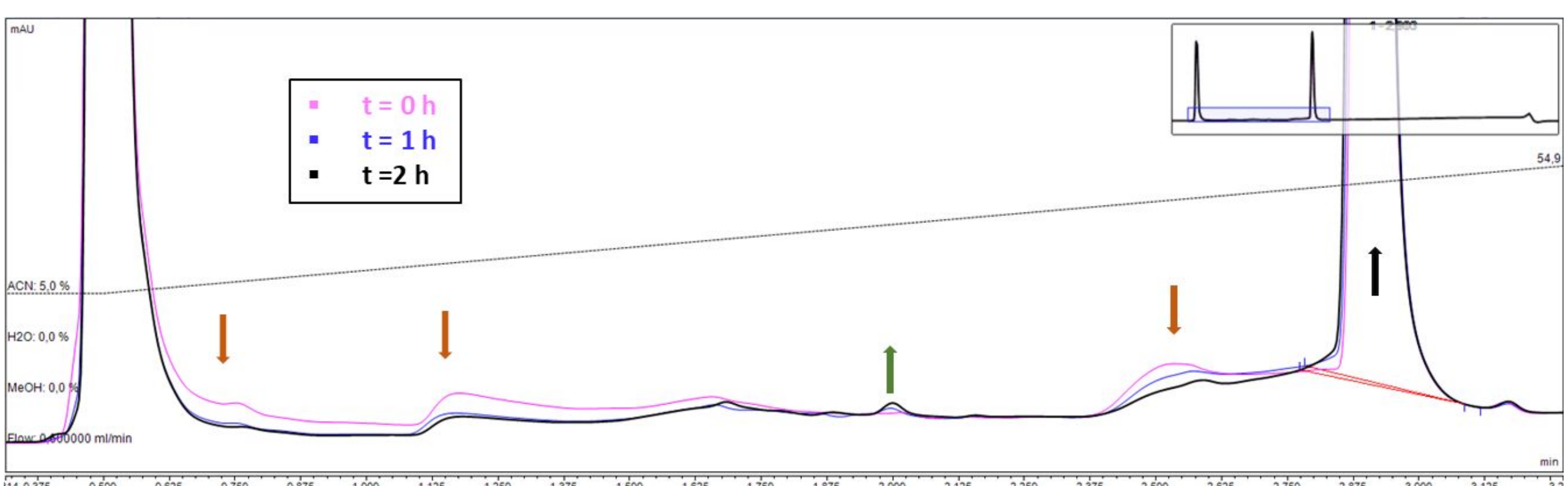

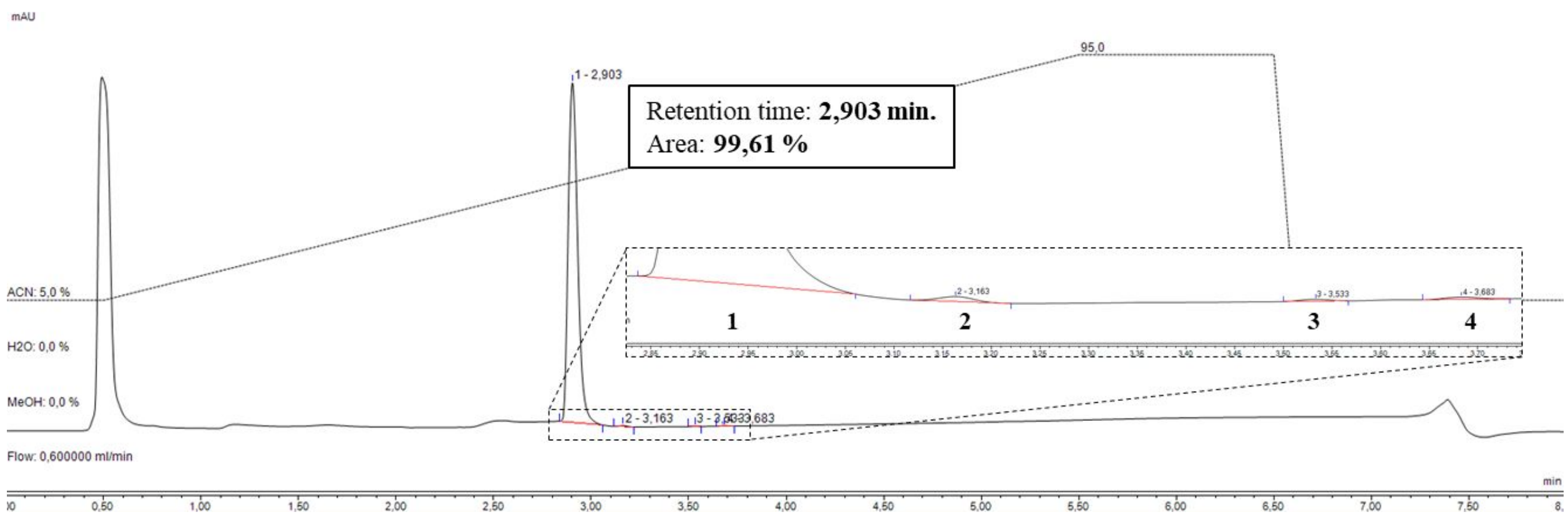

Peak No.	Peak Name	Ret.Time min	Amount n.a.	Rel.Area %	Area mAU*min	Height mAU	Type	Width (50%) min	Asym. EP	Resol. EP	Plates EP
1		2,903	n.a.	99,61	157,4125	2800,76	BMB*	0,052	1,34	3,34	17248
2		3,163	n.a.	0,22	0,3522	8,57	BMB*	0,040	1,00	6,04	34839
3		3,533	n.a.	0,07	0,1107	3,34	BMB*	0,032	0,99	2,52	65690
4		3,683	n.a.	0,10	0,1533	3,91	BMB*	0,038	1,07	n.a.	52694
Maximum			0,0000	99,61	157,4125	2800,76		0,052	1,34	6,04	65690
Minimum			0,0000	0,07	0,1107	3,34		0,032	0,99	2,52	17248
Sum			0,0000	100,00	158,0288	2816,57					

Ru2

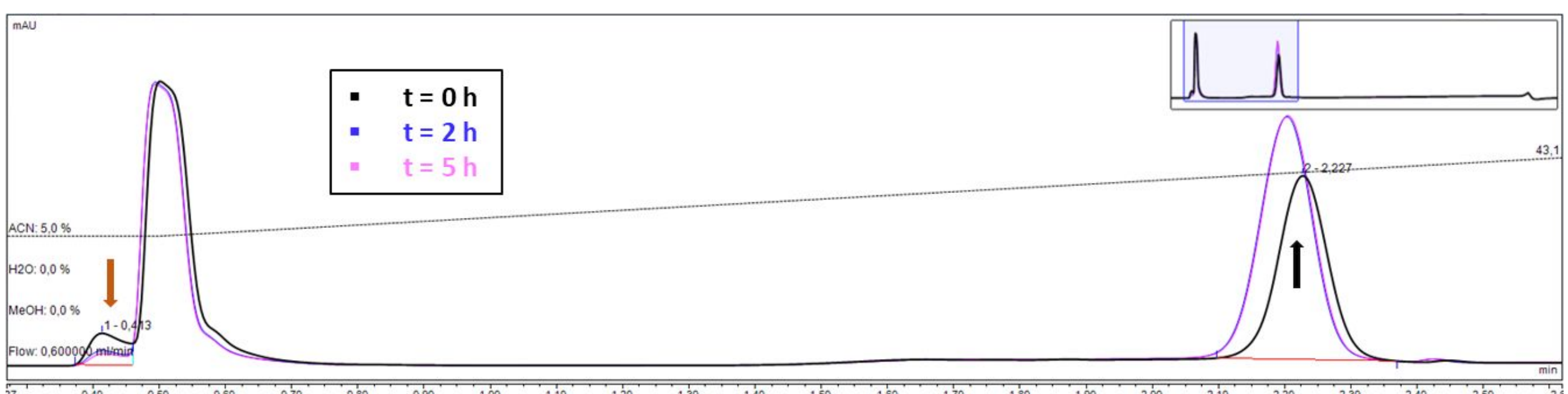

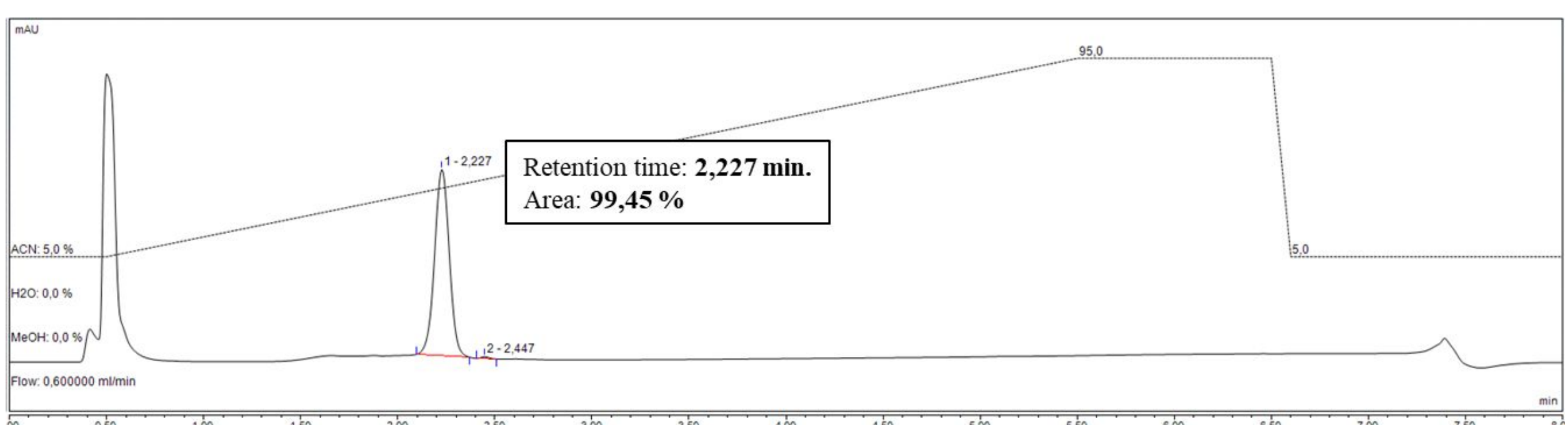

Peak No.	Peak Name	Ret.Time min	Amount n.a.	Rel.Area %	Area mAU*min	Height mAU	Type	Width (50%) min	Asym. EP	Resol. EP	Plates EP
1		2,227	n.a.	99,45	166,5383	1901,24	BMB*	0,081	1,01	2,09	4189
2		2,447	n.a.	0,55	0,9127	20,47	BMB*	0,043	1,25	n.a.	17966
Maximum			0,0000	99,45	166,5383	1901,24		0,081	1,25	2,09	17966
Minimum			0,0000	0,55	0,9127	20,47		0,043	1,01	2,09	4189
Sum			0,0000	100,00	167,4510	1921,70					

Ru3

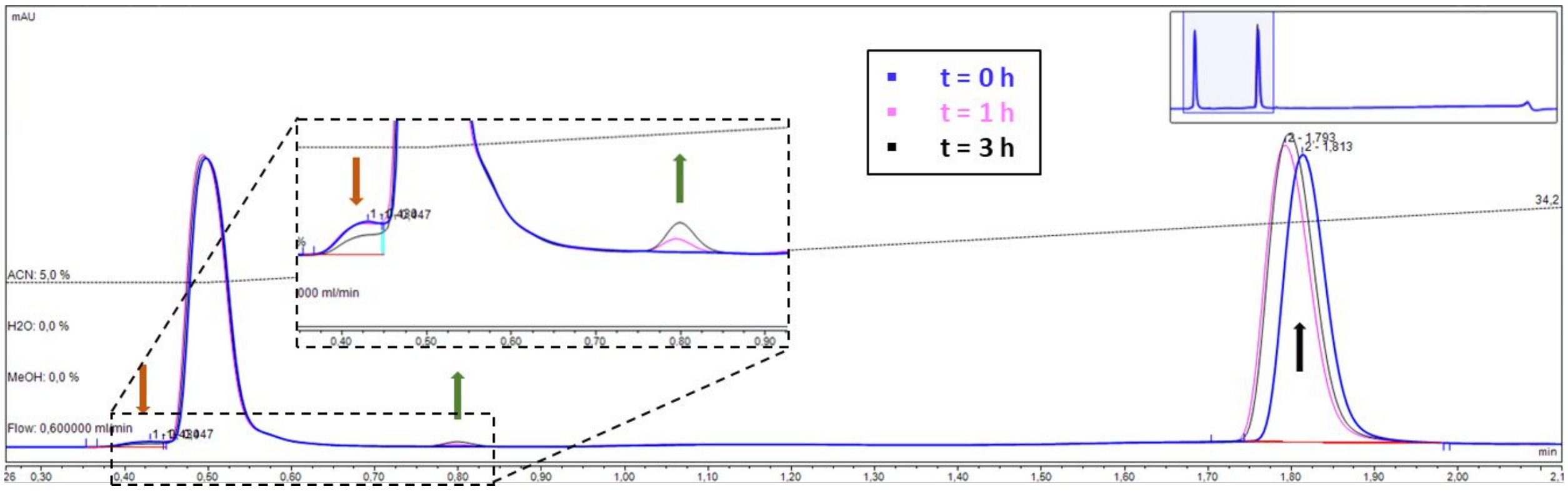

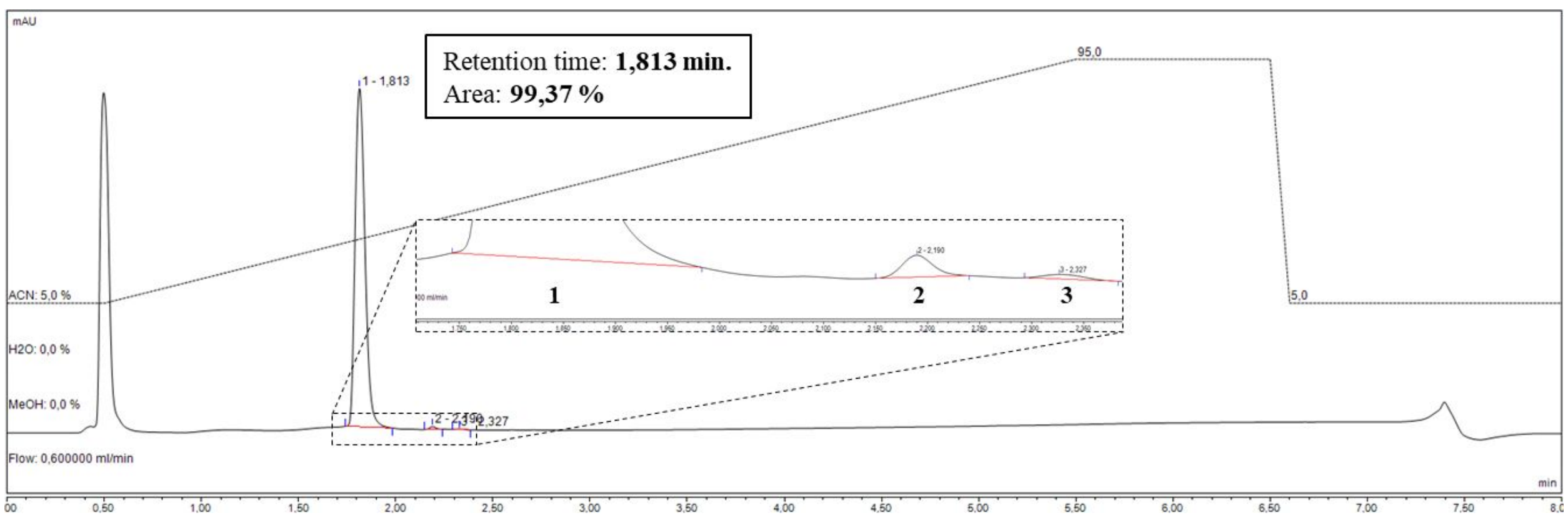

Peak No.	Peak Name	Ret.Time min	Amount n.a.	Rel.Area %	Area mAU*min	Height mAU	Type	Width (50%) min	Asym. EP	Resol. EP	Plates EP
1		1,813	n.a.	99,37	165,1747	2808,02	BMB*	0,055	1,28	5,12	6025
2		2,190	n.a.	0,49	0,8172	23,79	BMB*	0,032	1,14	2,11	26175
3		2,327	n.a.	0,14	0,2244	4,95	BMB*	0,045	1,24	n.a.	14987
Maximum			0,0000	99,37	165,1747	2808,02		0,055	1,28	5,12	26175
Minimum			0,0000	0,14	0,2244	4,95		0,032	1,14	2,11	6025
Sum			0,0000	100,00	166,2163	2836,76					

Ru4

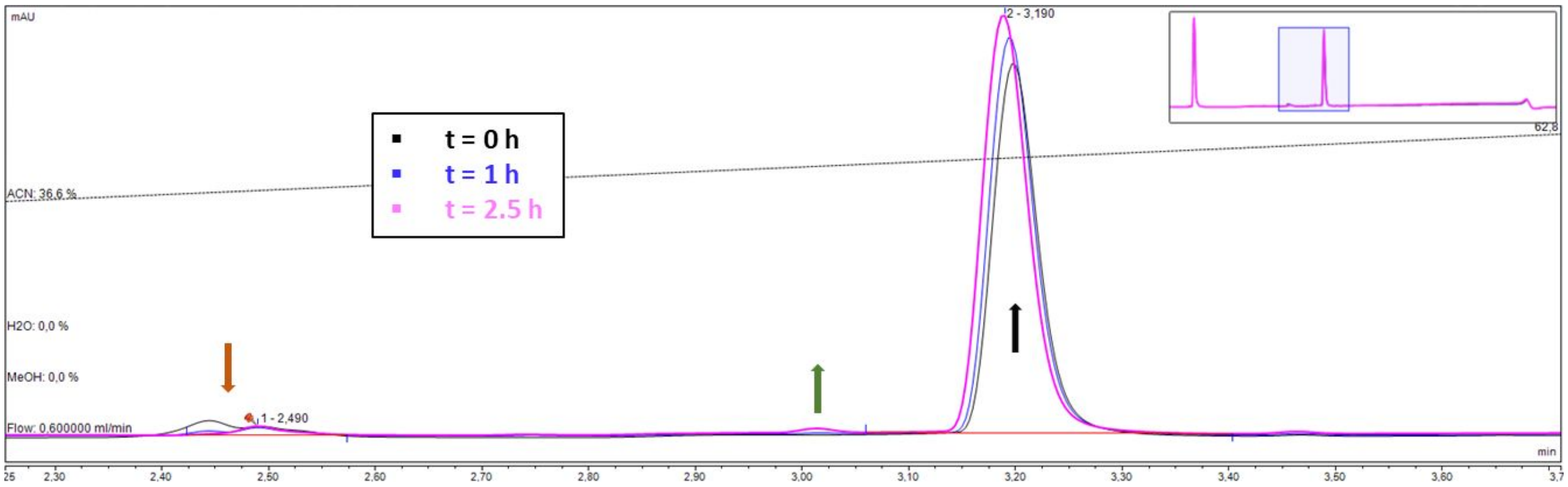

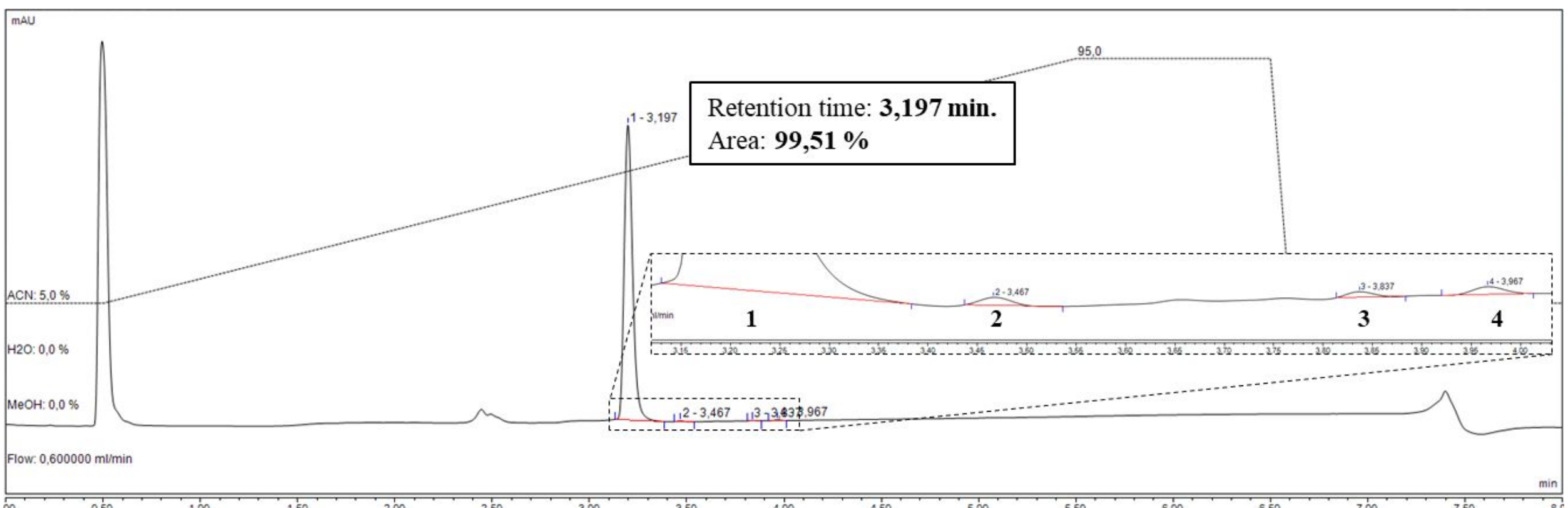

Peak No.	Peak Name	Ret.Time min	Amount n.a.	Rel.Area %	Area mAU*min	Height mAU	Type	Width (50%) min	Asym. EP	Resol. EP	Plates EP
1		3,197	n.a.	99,51	106,9084	2134,62	BMB*	0,046	1,32	3,94	26912
2		3,467	n.a.	0,19	0,2010	5,81	BMB*	0,035	1,11	6,75	54087
3		3,837	n.a.	0,11	0,1171	4,00	BMB*	0,030	1,15	2,35	93347
4		3,967	n.a.	0,19	0,2082	5,63	BMB*	0,036	1,11	n.a.	68776
Maximum			0,0000	99,51	106,9084	2134,62		0,046	1,32	6,75	93347
Minimum			0,0000	0,11	0,1171	4,00		0,030	1,11	2,35	26912
Sum			0,0000	100,00	107,4347	2150,07					

Ru5

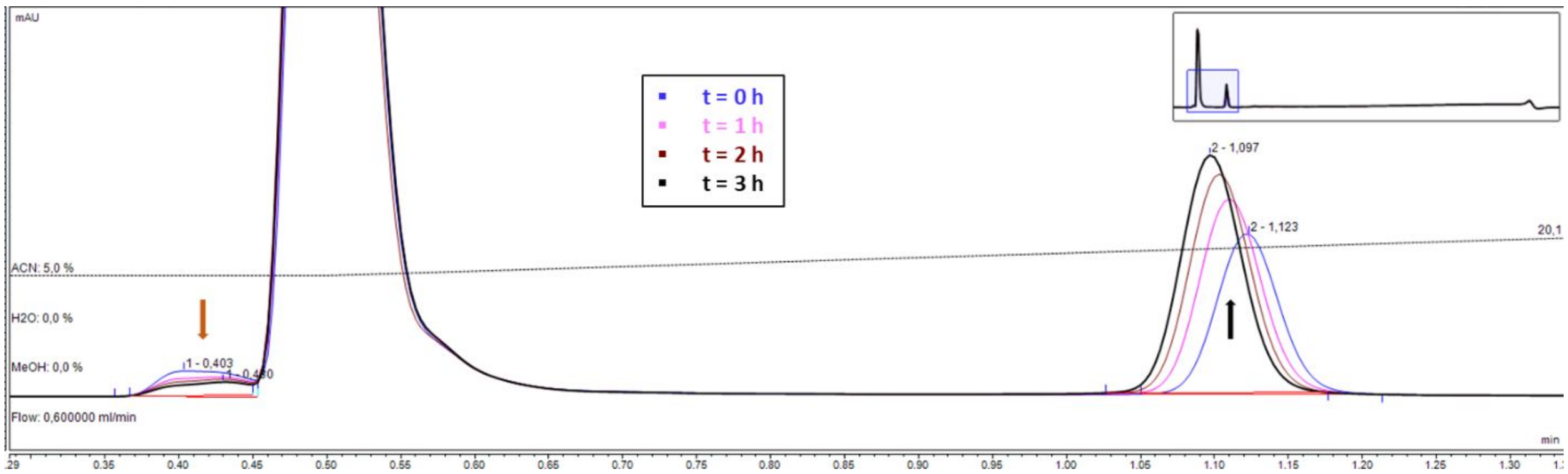

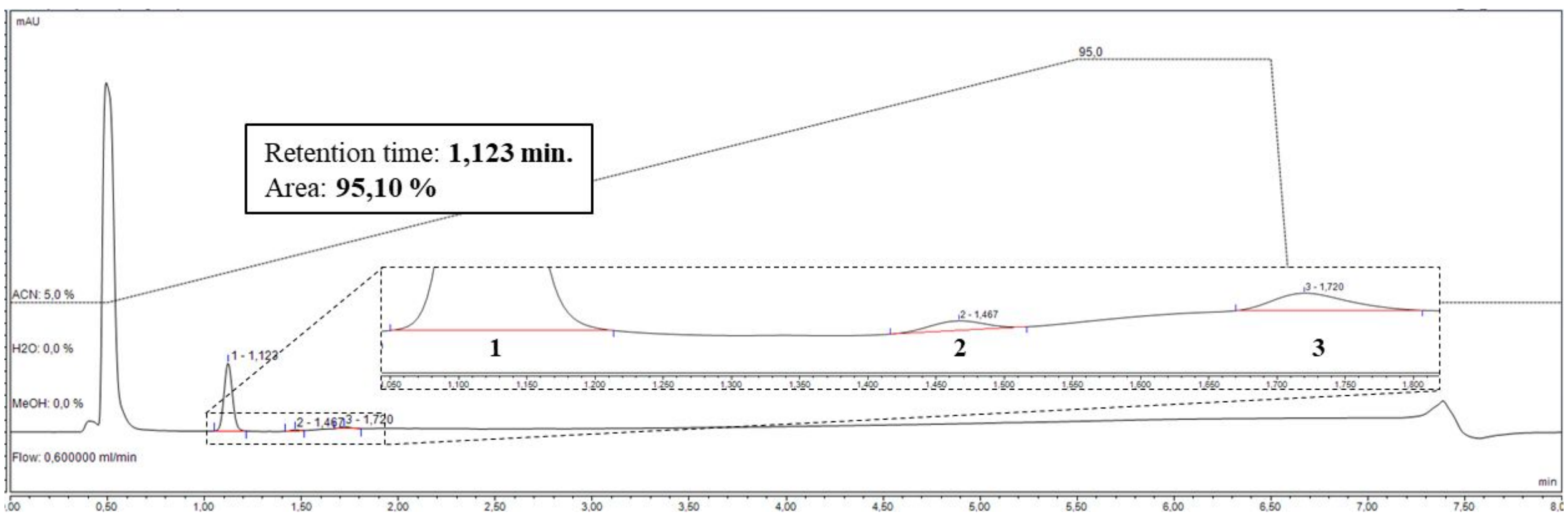

Peak No.	Peak Name	Ret.Time min	Amount n.a.	Rel.Area %	Area mAU*min	Height mAU	Type	Width (50%) min	Asym. EP	Resol. EP	Plates EP
1		1,123	n.a.	95,10	28,2461	564,86	BMB*	0,047	1,04	4,45	3210
2		1,467	n.a.	1,39	0,4137	9,04	BMB*	0,044	0,96	2,86	6044
3		1,720	n.a.	3,51	1,0411	16,46	BMB*	0,060	1,33	n.a.	4529
Maximum			0,0000	95,10	28,2461	564,86		0,060	1,33	4,45	6044
Minimum			0,0000	1,39	0,4137	9,04		0,044	0,96	2,86	3210
Sum			0,0000	100,00	29,7009	590,36					

Ru6

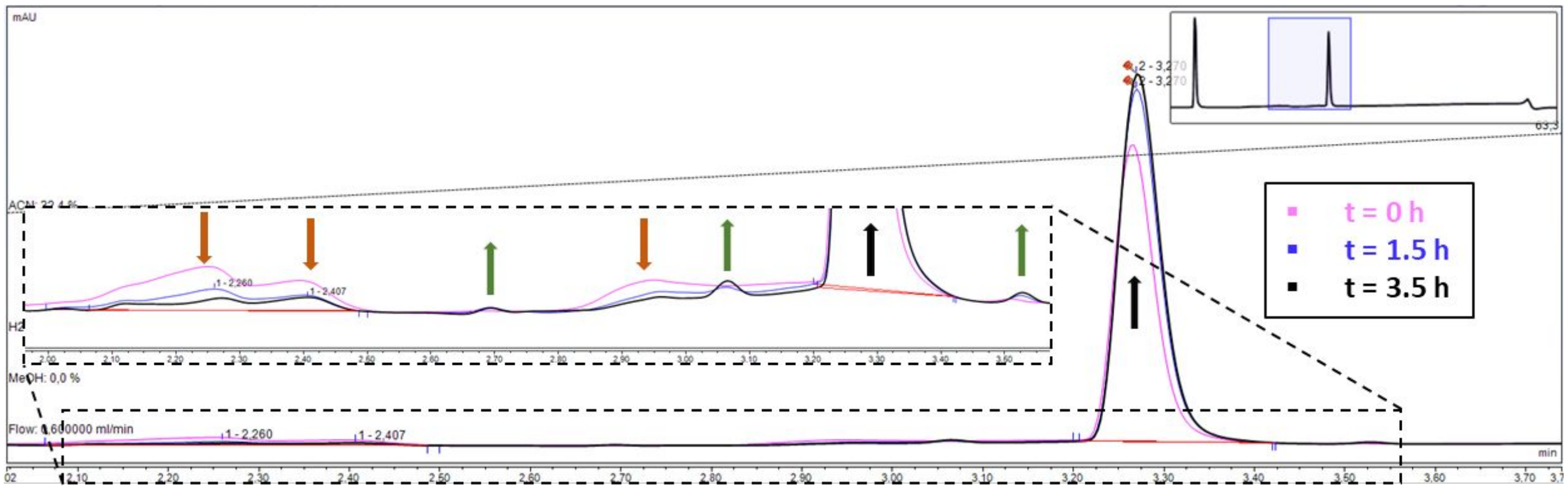

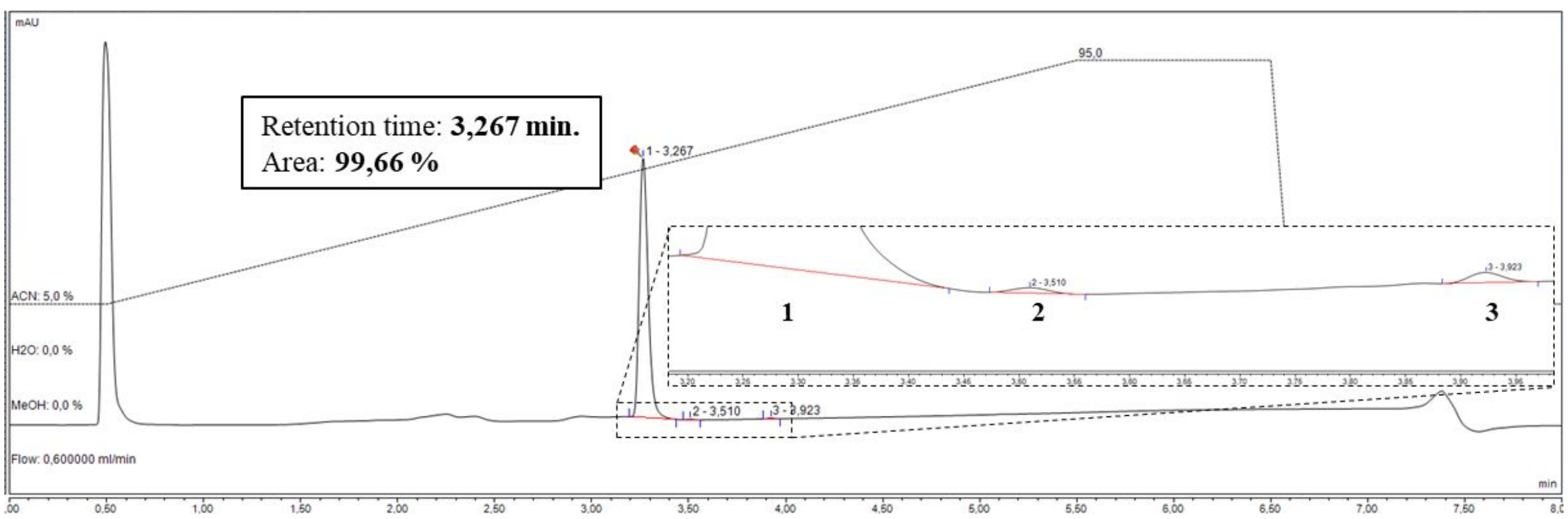

Peak No.	Peak Name	Ret.Time min	Amount n.a.	Rel.Area %	Area mAU*min	Height mAU	Type	Width (50%) min	Asym. EP	Resol. EP	Plates EP
1		3,267	n.a.	99,66	99,4222	1881,61	BMB*^	0,049	1,26	3,39	25073
2		3,510	n.a.	0,12	0,1211	3,28	BMB*	0,036	1,01	6,95	51981
3		3,923	n.a.	0,22	0,2160	6,12	BMB*	0,034	1,06	n.a.	74226
Maximum			0,0000	99,66	99,4222	1881,61		0,049	1,26	6,95	74226
Minimum			0,0000	0,12	0,1211	3,28		0,034	1,01	3,39	25073
Sum			0,0000	100,00	99,7593	1891,01					

Ru6

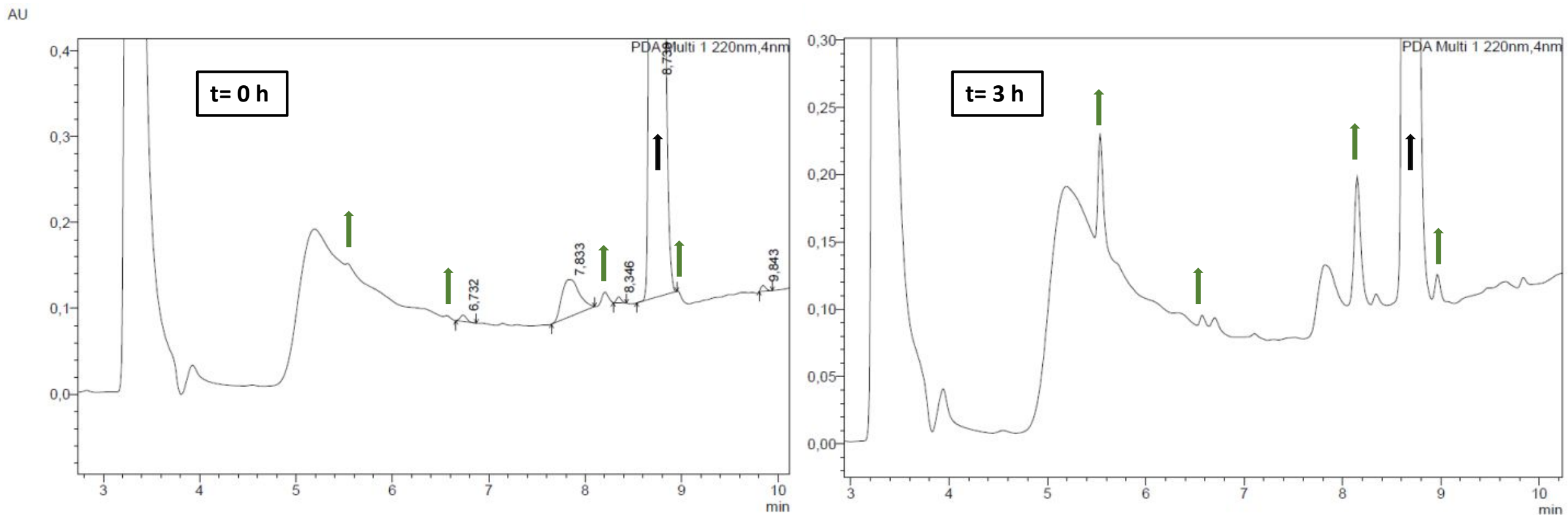

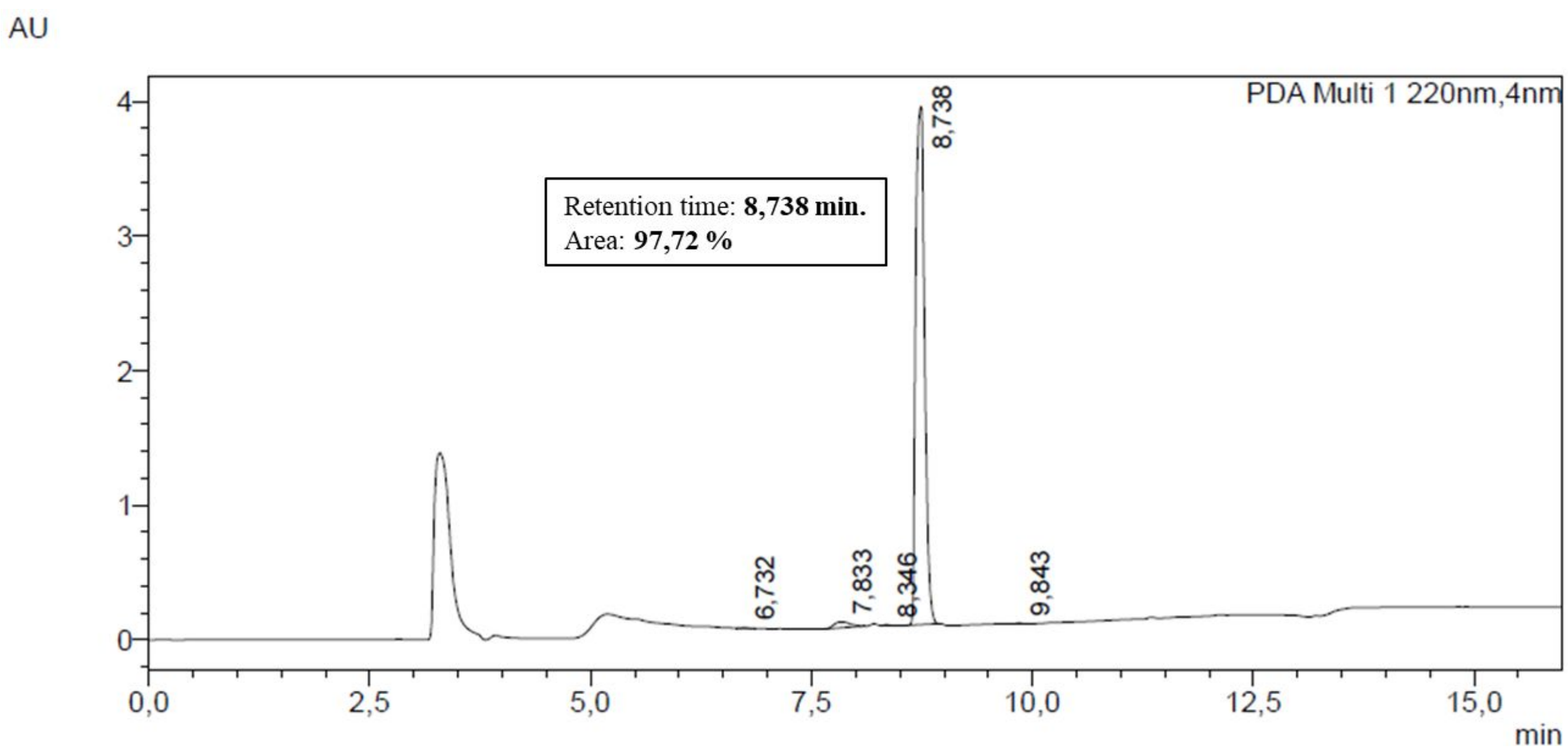

<Peak Table>

PDA Ch1 220nm

Peak#	Ret. Time	Area	Height	Area%
1	6,732	32563	7538	0,117
2	7,833	555285	43713	2,000
3	8,346	22801	6805	0,082
4	8,738	27125392	3847033	97,716
5	9,843	23372	6694	0,084
Total		27759414	3911782	100,000

Ru7

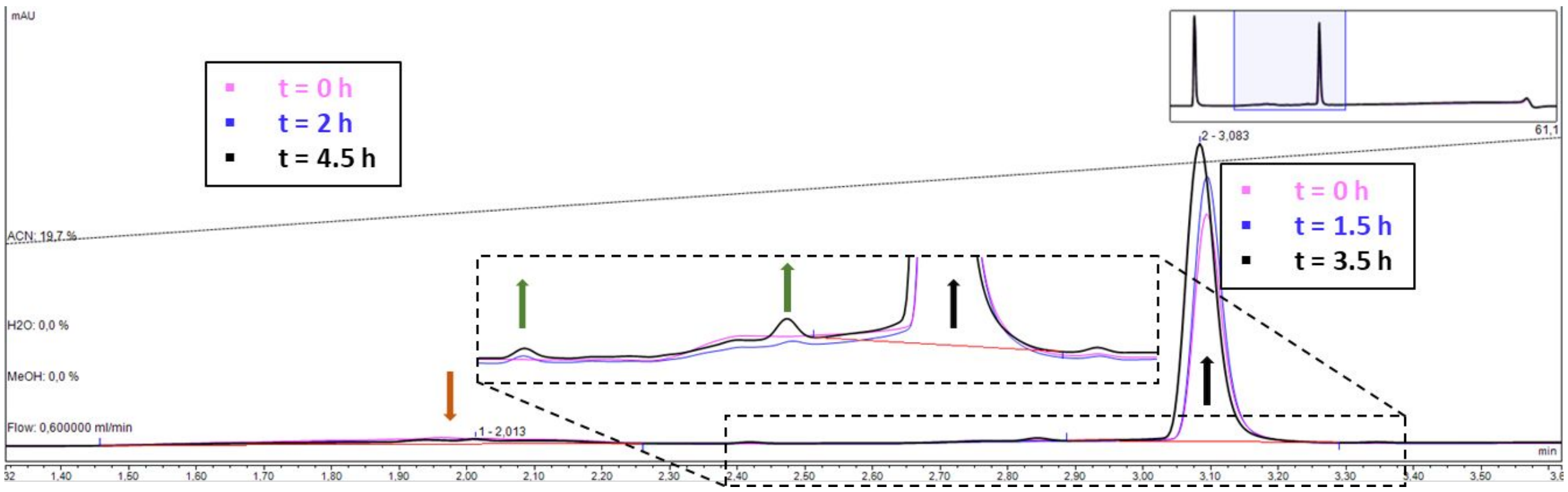

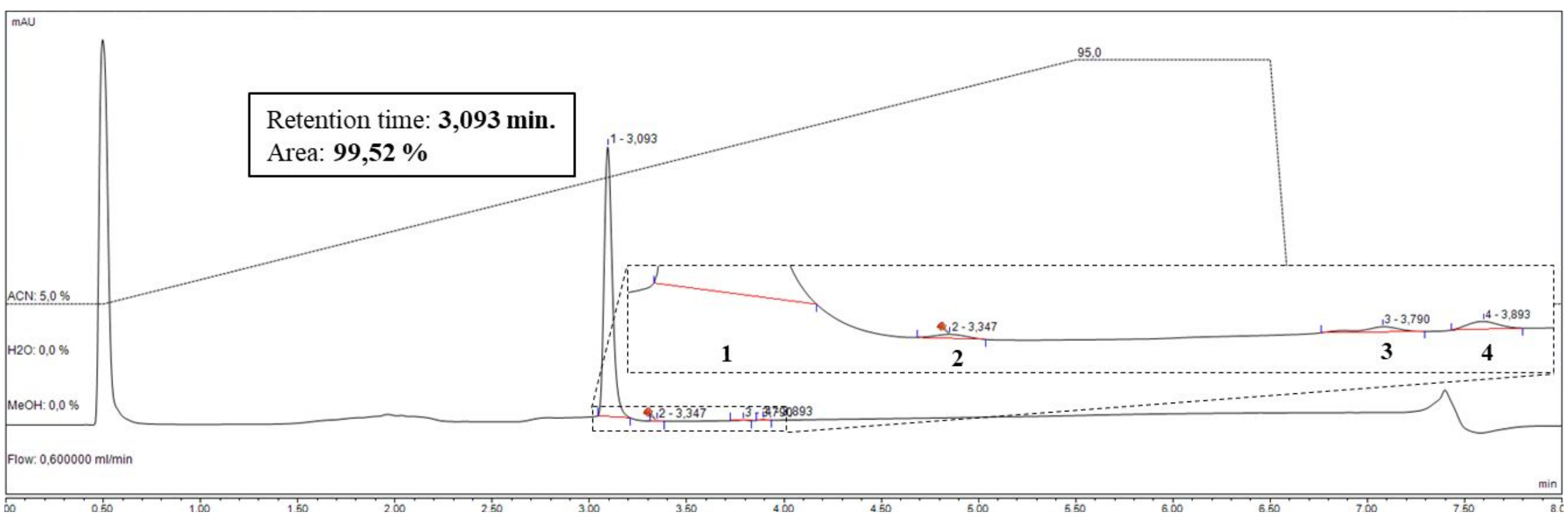

Peak No.	Peak Name	Ret.Time min	Amount n.a.	Rel.Area %	Area mAU*min	Height mAU	Type	Width (50%) min	Asym. EP	Resol. EP	Plates EP
1		3,093	n.a.	99,52	95,2872	1956,32	BMB*	0,044	1,34	3,74	26830
2		3,347	n.a.	0,10	0,0991	2,75	BMB*^	0,035	1,05	7,48	49246
3		3,790	n.a.	0,17	0,1636	3,57	BMB*	0,034	0,79	1,73	67037
4		3,893	n.a.	0,20	0,1957	5,31	BMB*	0,036	1,05	n.a.	64334
Maximum			0,0000	99,52	95,2872	1956,32		0,044	1,34	7,48	67037
Minimum			0,0000	0,10	0,0991	2,75		0,034	0,79	1,73	26830
Sum			0,0000	100,00	95,7457	1967,94					

Ru8

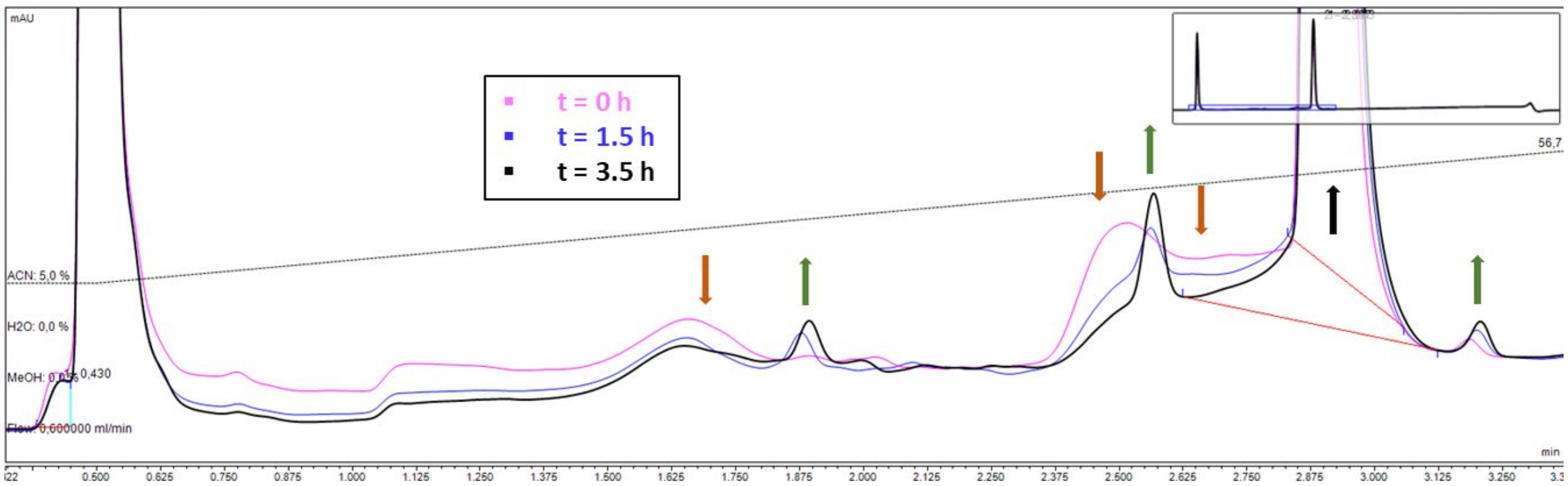

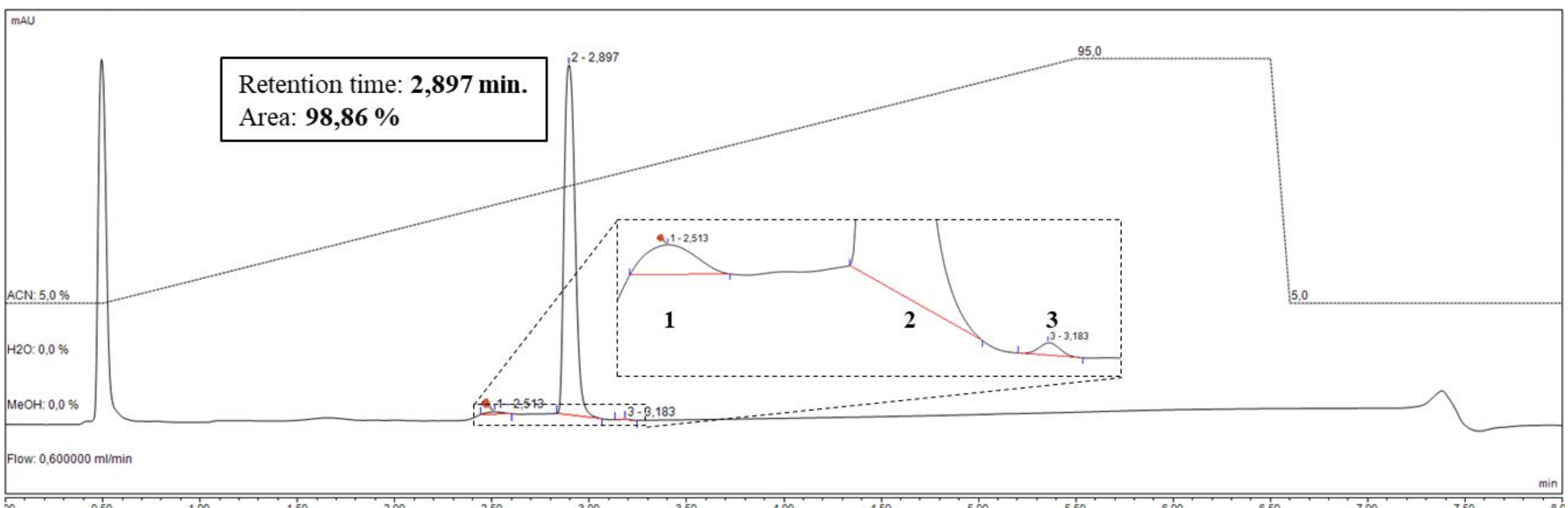

Peak No.	Peak Name	Ret.Time min	Amount n.a.	Rel.Area %	Area mAU*min	Height mAU	Type	Width (50%) min	Asym. EP	Resol. EP	Plates EP
1		2,513	n.a.	0,97	1,6091	15,76	BMB*^	0,105	1,25	2,71	3152
2		2,897	n.a.	98,86	164,2945	2531,88	BMB*	0,062	1,25	3,30	12165
3		3,183	n.a.	0,17	0,2821	6,61	BMB*	0,041	1,05	n.a.	34096
Maximum			0,0000	98,86	164,2945	2531,88		0,105	1,25	3,30	34096
Minimum			0,0000	0,17	0,2821	6,61		0,041	1,05	2,71	3152
Sum			0,0000	100,00	166,1857	2554,25					

Ru9

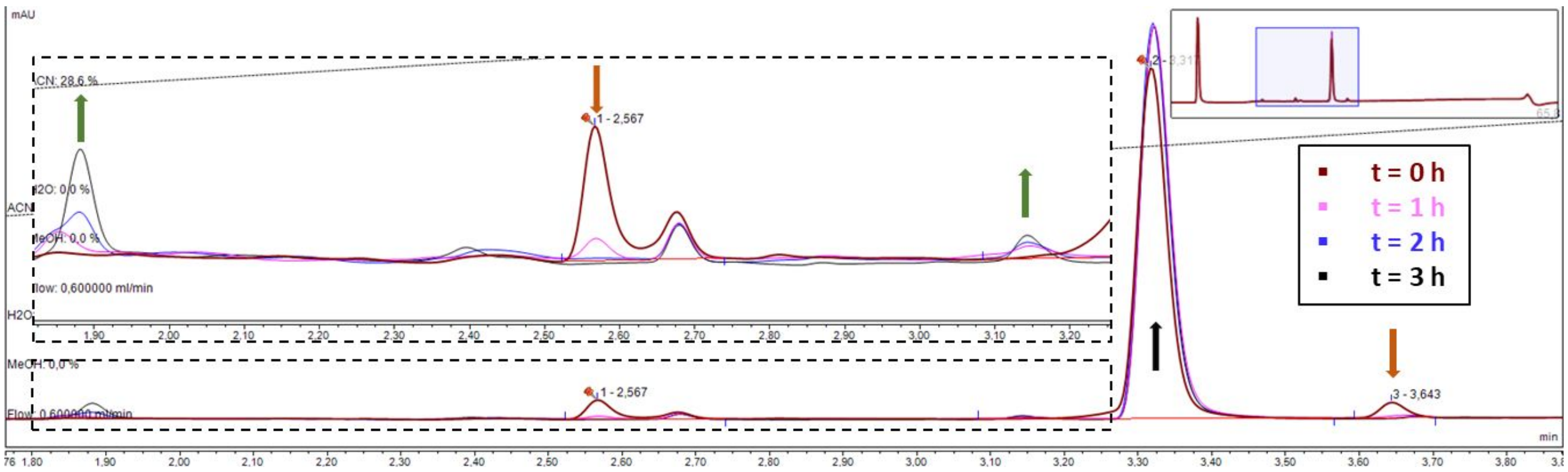

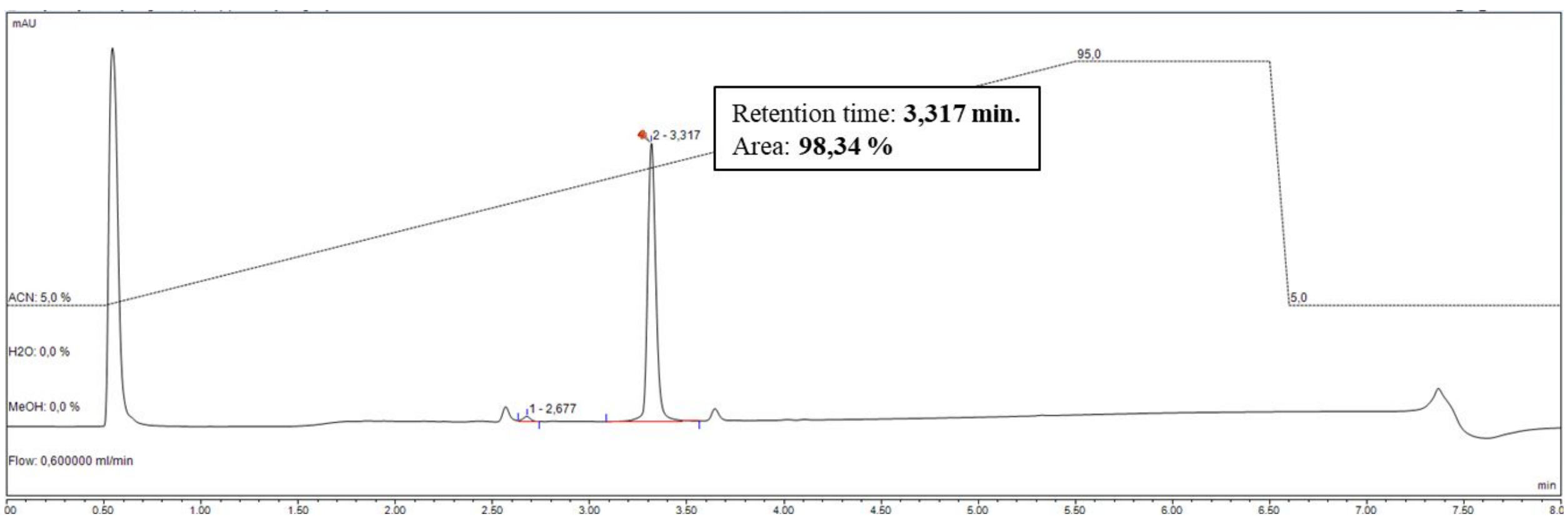

Peak No.	Peak Name	Ret.Time min	Amount n.a.	Rel.Area %	Area mAU*min	Height mAU	Type	Width (50%) min	Asym. EP	Resol. EP	Plates EP
1		2,677	n.a.	1,66	1,6735	37,50	MB*	0,039	n.a.	9,07	25692
2		3,317	n.a.	98,34	99,0507	2018,32	BMB^	0,044	1,23	n.a.	31516
Maximum			0,0000	98,34	99,0507	2018,32		0,044	1,23	9,07	31516
Minimum			0,0000	1,66	1,6735	37,50		0,039	1,23	9,07	25692
Sum			0,0000	100,00	100,7242	2055,82					

Ru10

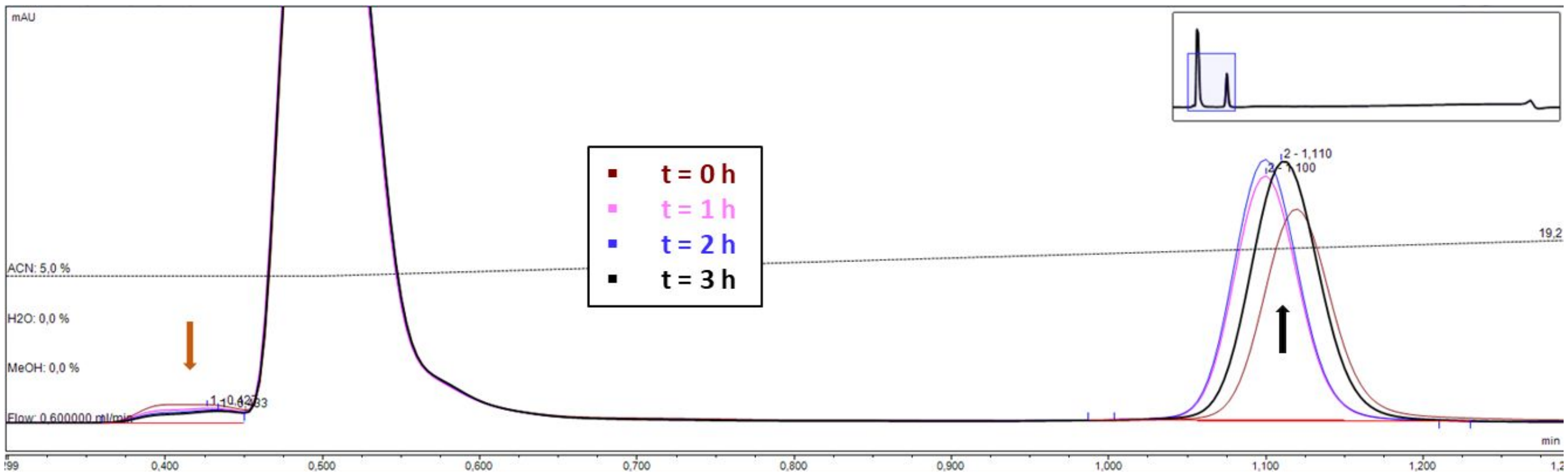

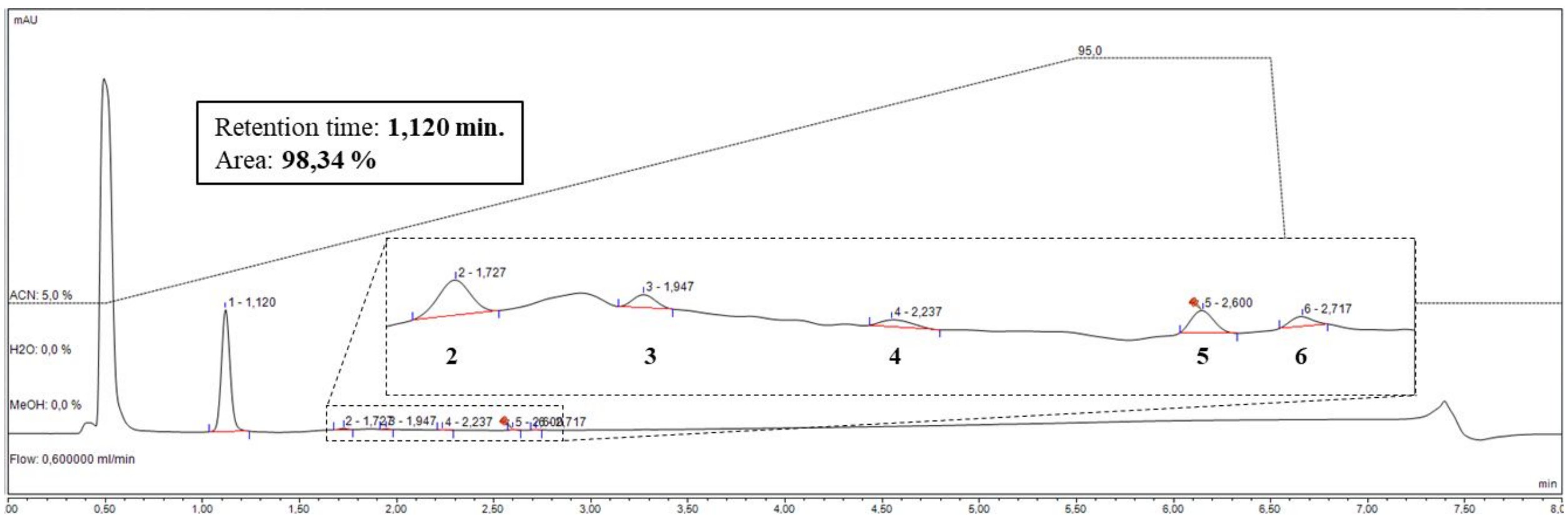

Peak No.	Peak Name	Ret.Time min	Amount n.a.	Rel.Area %	Area mAU*min	Height mAU	Type	Width (50%) min	Asym. EP	Resol. EP	Plates EP
1		1,120	n.a.	98,34	52,2118	1005,49	BMB*	0,048	1,09	8,02	3068
2		1,727	n.a.	0,80	0,4221	9,62	BMB*	0,042	0,94	3,63	9491
3		1,947	n.a.	0,20	0,1082	3,57	BMB*	0,030	1,07	4,64	23725
4		2,237	n.a.	0,16	0,0829	1,88	BMB*	0,044	1,43	5,82	14359
5		2,600	n.a.	0,35	0,1868	6,14	BMB*^	0,030	1,10	2,32	42538
6		2,717	n.a.	0,15	0,0806	2,65	BMB*	0,030	1,05	n.a.	46544
Maximum			0,0000	98,34	52,2118	1005,49		0,048	1,43	8,02	46544
Minimum			0,0000	0,15	0,0806	1,88		0,030	0,94	2,32	3068
Sum			0,0000	100,00	53,0923	1029,35					

Ru11

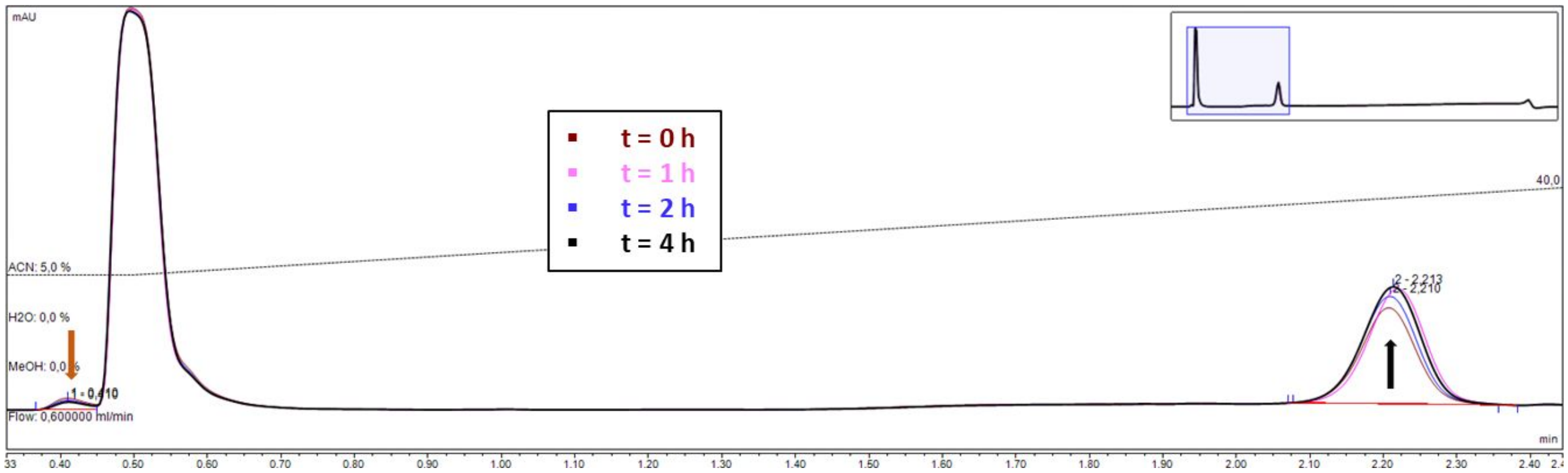

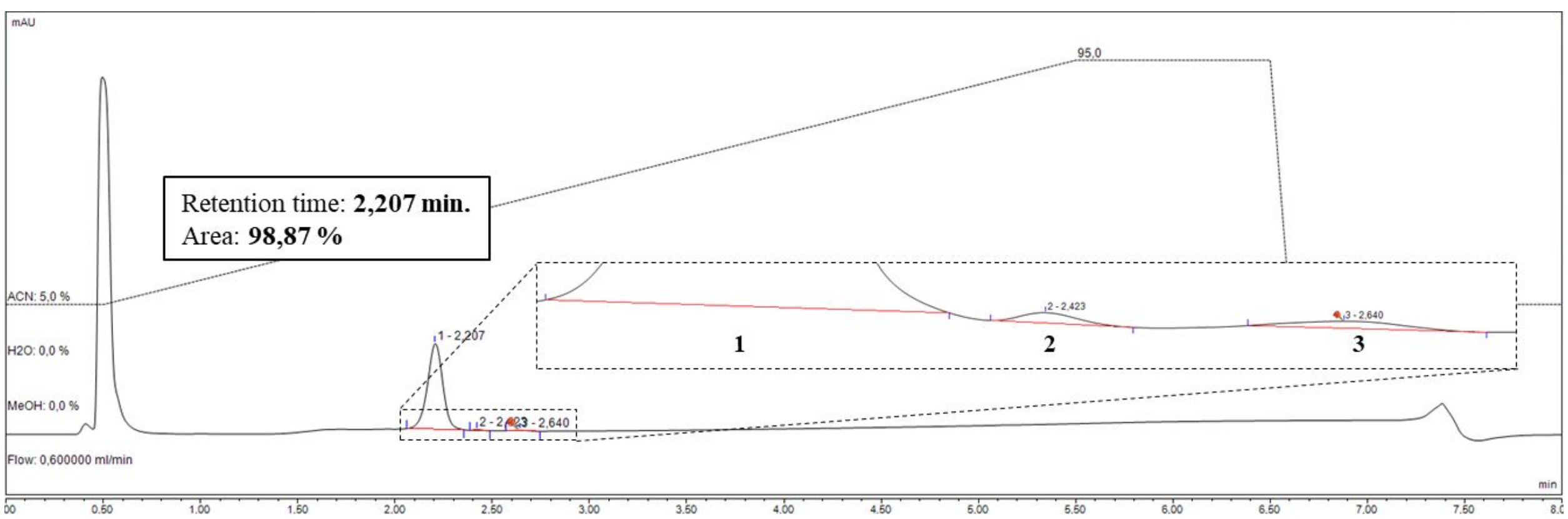

Peak No.	Peak Name	Ret.Time min	Amount n.a.	Rel.Area %	Area mAU*min	Height mAU	Type	Width (50%) min	Asym. EP	Resol. EP	Plates EP
1		2,207	n.a.	98,87	64,3929	710,40	BMB*	0,082	0,96	2,01	3992
2		2,423	n.a.	0,49	0,3185	6,82	BMB*	0,045	1,23	1,86	15852
3		2,640	n.a.	0,64	0,4190	4,58	BMB*^	0,092	1,11	n.a.	4531
Maximum			0,0000	98,87	64,3929	710,40		0,092	1,23	2,01	15852
Minimum			0,0000	0,49	0,3185	4,58		0,045	0,96	1,86	3992
Sum			0,0000	100,00	65,1304	721,80					

Ru12

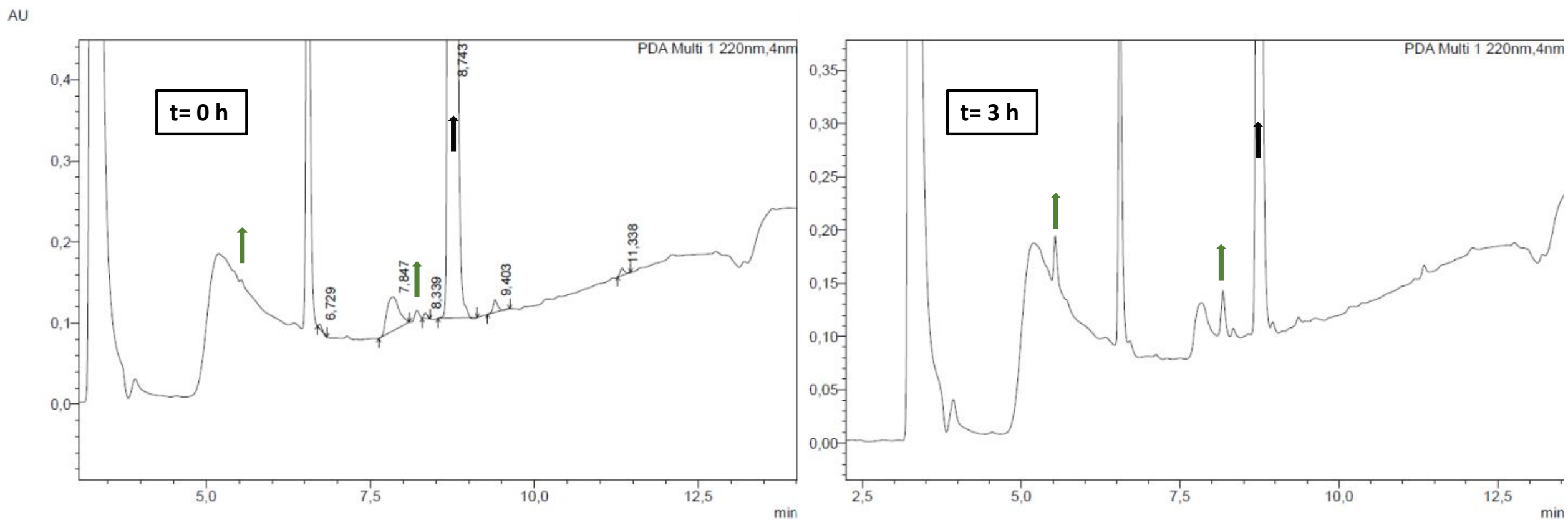

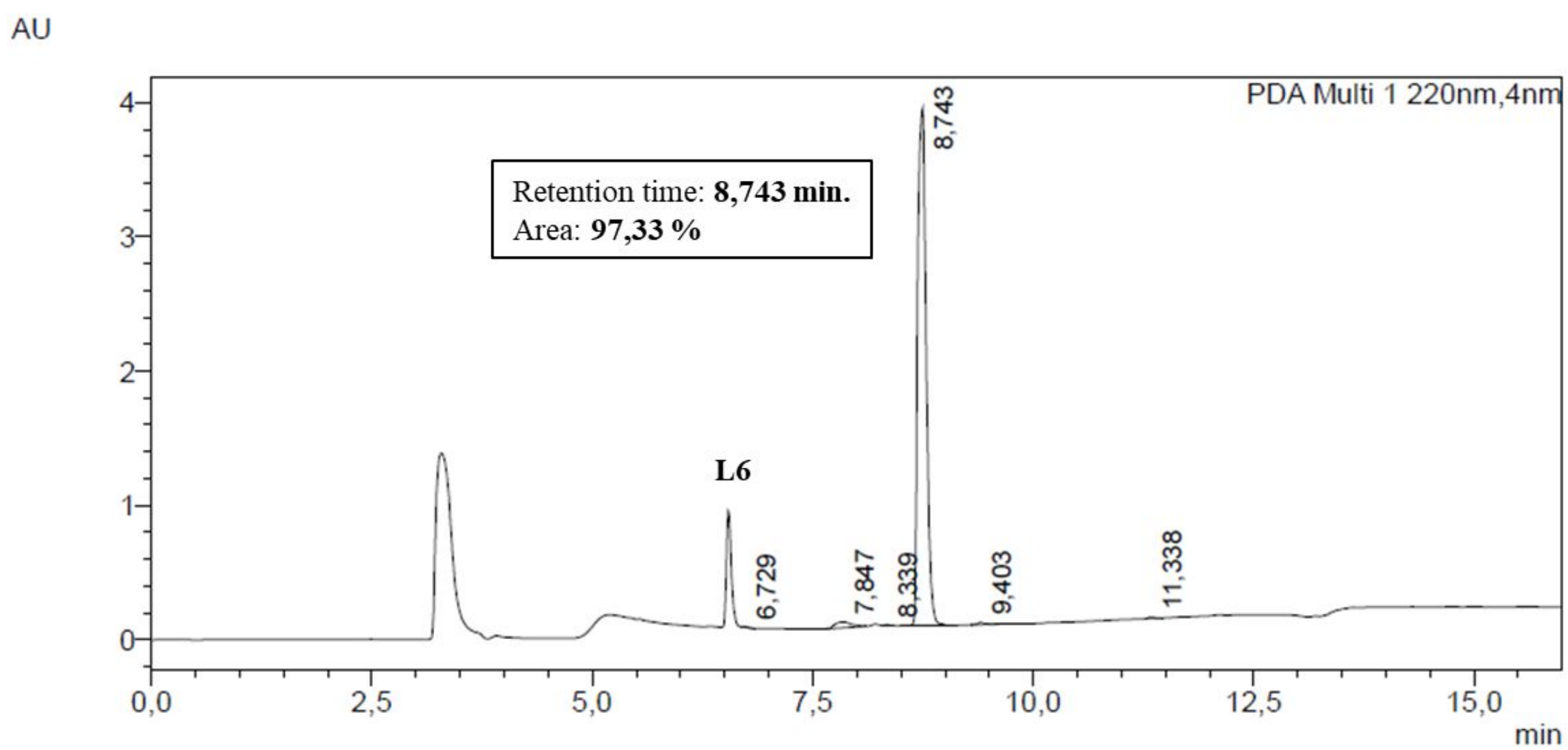

<Peak Table>

PDA Ch1 220nm

Peak#	Ret. Time	Area	Height	Area%
1	6,729	14906	5825	0,056
2	7,847	548855	41501	2,080
3	8,339	22083	6746	0,084
4	8,743	25679909	3854285	97,334
5	9,403	75675	15143	0,287
6	11,338	41904	9222	0,159
Total		26383333	3932722	100,000

(1) Van Der Sluis, P.; Spek, A. L. BYPASS: an effective method for the refinement of crystal structures containing disordered solvent regions. *Acta Crystallographica Section A* **1990**, *46* (3), 194-201. DOI: https://doi.org/10.1107/S0108767389011189.
(2) Trott, O.; Olson, A. J. AutoDock Vina: improving the speed and accuracy of docking with a new scoring function, efficient optimization, and multithreading. *J Comput Chem* **2010**, *31* (2), 455-461. DOI: 10.1002/jcc.21334.
(3) Morris, G. M.; Huey, R.; Lindstrom, W.; Sanner, M. F.; Belew, R. K.; Goodsell, D. S.; Olson, A. J. AutoDock4 and AutoDockTools4: Automated docking with selective receptor flexibility. *J Comput Chem* **2009**, *30* (16), 2785-2791. DOI: 10.1002/jcc.21256.
(4) Berman, H. M.; Westbrook, J.; Feng, Z.; Gilliland, G.; Bhat, T. N.; Weissig, H.; Shindyalov, I. N.; Bourne, P. E. The Protein Data Bank. *Nucleic Acids Research* **2000**, *28* (1), 235-242. DOI: 10.1093/nar/28.1.235.
(5) Morgan, C. A.; Hurley, T. D. Characterization of two distinct structural classes of selective aldehyde dehydrogenase 1A1 inhibitors. *J Med Chem* **2015**, *58* (4), 1964-1975. DOI: 10.1021/jm501900s.
(6) Morgan, C. A.; Hurley, T. D. *Structure of human ALDH1A1 with inhibitor CM053*. 2014.
(7) Moretti, A.; Li, J.; Donini, S.; Sobol, R. W.; Rizzi, M.; Garavaglia, S. Crystal structure of human aldehyde dehydrogenase 1A3 complexed with NAD(+) and retinoic acid. *Sci Rep* **2016**, *6*, 35710. DOI: 10.1038/srep35710.
(8) Moretti, A.; Rizzi, M.; Garavaglia, S. *Human aldehyde dehydrogenase 1A3 complexed with NAD(+) and retinoic acid*. 2015.
(9) Hurley, T. D.; Parajuli, B. *Crystal structure of human ALDH3A1 with its isozyme selective inhibitor - N-[4-(4-methylsulfonyl-2-nitroanilino)phenyl]acetamide*. 2012. (accessed.
(10) *Gaussian 09*; Wallingford, CT, 2009.
(11) Becke, A. D. Density-functional thermochemistry. III. The role of exact exchange. *The Journal of Chemical Physics* **1993**, *98* (7), 5648-5652. DOI: 10.1063/1.464913.
(12) Lee, C.; Yang, W.; Parr, R. G. Development of the Colle-Salvetti correlation-energy formula into a functional of the electron density. *Phys Rev B Condens Matter* **1988**, *37* (2), 785-789. DOI: 10.1103/physrevb.37.785.
(13) Dunning, T. H., Jr. Gaussian basis sets for use in correlated molecular calculations. I. The atoms boron through neon and hydrogen. *The Journal of Chemical Physics* **1989**, *90* (2), 1007-1023. DOI: 10.1063/1.456153.
(14) *Discovery studio modeling environment*; Release San Diego, CA: San Diego: Dassault Systèmes, 2017.
(15) Rebollido-Rios, R.; Venton, G.; Sánchez-Redondo, S.; Iglesias, I. F. C.; Fournet, G.; González, E.; Romero Fernández, W.; Borroto Escuela, D. O.; Di Stefano, B.; Penarroche-Díaz, R.; et al. Dual disruption of aldehyde dehydrogenases 1 and 3 promotes functional changes in the glutathione redox system and enhances chemosensitivity in nonsmall cell lung cancer. *Oncogene* **2020**, *39* (13), 2756-2771. DOI: 10.1038/s41388-020-1184-9.
(16) Ibrahim, A. I. M.; Ikhmais, B.; Batlle, E.; AbuHarb, W. K.; Jha, V.; Jaradat, K. T.; Jiménez, R.; Pequerul, R.; Parés, X.; Farrés, J.; Pors, K. Design, Synthesis, Biological Evaluation and In

Silico Study of Benzyloxybenzaldehyde Derivatives as Selective ALDH1A3 Inhibitors. *Molecules* 2021, *26* (19). DOI: 10.3390/molecules26195770.
(17) Park, J. W.; Jung, K. H.; Lee, J. H.; Moon, S. H.; Cho, Y. S.; Lee, K. H. Inhibition of aldehyde dehydrogenase 1 enhances the cytotoxic effect of retinaldehyde on A549 cancer cells. *Oncotarget* 2017, *8* (59), 99382-99393. DOI: 10.18632/oncotarget.19544.
(18) Batlle, E.; Pequerul, R.; Farrés, J.; Eriksson, L. A.; Pors, K.; Jha, V. Discovery of a reversible ALDH1A3 inhibitor throwugh a consensus docking-based virtual screening study. *J Comput Aided Mol Des* 2025, *39* (1), 72. DOI: 10.1007/s10822-025-00622-3.
(19) Ogunwa, T. Computer-Aided Modeling of Interaction between Aldehyde Dehydrogenase and Garcinia Biflavonoids. *International Journal of Computer Applications* 2018, *179*, 18-25. DOI: 10.5120/ijca2018916788.
(20) Dinavahi, S. S.; Gowda, R.; Gowda, K.; Bazewicz, C. G.; Chirasani, V. R.; Battu, M. B.; Berg, A.; Dokholyan, N. V.; Amin, S.; Robertson, G. P. Development of a Novel Multi-Isoform ALDH Inhibitor Effective as an Antimelanoma Agent. *Mol Cancer Ther* 2020, *19* (2), 447-459. DOI: 10.1158/1535-7163.Mct-19-0360.
